# Inotuzumab ozogamicin as single agent in pediatric patients with relapsed and refractory acute lymphoblastic leukemia: results from a phase II trial

**DOI:** 10.1038/s41375-022-01576-3

**Published:** 2022-04-25

**Authors:** Edoardo Pennesi, Naomi Michels, Erica Brivio, Vincent H. J. van der Velden, Yilin Jiang, Adriana Thano, Anneke J. C. Ammerlaan, Judith M. Boer, H. Berna Beverloo, Barbara Sleight, Ying Chen, Britta Vormoor-Bürger, Susana Rives, Bella Bielorai, Claudia Rössig, Arnaud Petit, Carmelo Rizzari, Gernot Engstler, Jan Starý, Francisco J. Bautista Sirvent, Christiane Chen-Santel, Benedicte Bruno, Yves Bertrand, Fanny Rialland, Geneviève Plat, Dirk Reinhardt, Luciana Vinti, Arend Von Stackelberg, Franco Locatelli, Christian M. Zwaan

**Affiliations:** 1 Princess Máxima Center for Pediatric Oncology, Utrecht, the Netherlands; 2 Department of Pediatric Oncology, Erasmus MC-Sophia Children’s Hospital, Rotterdam, the Netherlands; 3 Oncode Institute, Utrecht, the Netherlands; 4 Department of Immunology, Erasmus MC, University Medical Center Rotterdam, Rotterdam, the Netherlands; 5 Department of Clinical Genetics, Erasmus MC, University Medical Center Rotterdam, Rotterdam, the Netherlands; 6 Pfizer Inc, Groton, CT USA; 7 Pediatric Oncology and Hematology Department, Hospital Sant Joan de Déu de Barcelona, Barcelona, Spain; 8 Institut de Recerca Sant Joan de Déu, Barcelona, Spain; 9 Division of Pediatric Hematology and Oncology, Sheba Medical Center, Ramat-Gan, Israel; 10 Pediatric Hematology and Oncology, University Children’s Hospital Muenster, Münster, Germany; 11 Department of pediatric Hematology and Oncology, Hopital Armand Trousseau, APHP, Sorbonne Université, Paris, France; 12 Pediatric Hematology-Oncology Unit, Department of Pediatrics, MBBM Foundation, ASST Monza, University of Milano-Bicocca, Monza, Italy; 13 St Anna Children’s Hospital, Medical University of Vienna, Vienna, Austria; 14 Department of Pediatric Hematology and Oncology, University Hospital Motol, Prague, Czech Republic; 15 Department of Pediatric Oncology and Hematology, Hospital Niño Jesús, Madrid, Spain; 16 Department of Pediatrics, Division of Oncology and Hematology, Charité – Universitätsmedizin Berlin, Berlin, Germany; 17 Department of Pediatrics, Rostock University Medical Centre, Rostock, Germany; 18 Pediatric Hematology, Hôpital Jeanne de Flandre, CHRU de Lille, Lille, France; 19 Institute of Pediatric Hematology and Oncology, Civil Hospital of Lyon, Claude Bernard University, Lyon, France; 20 Service Onco-Hématologie Pédiatrique, Hôpital Mère-Enfant, Nantes University Hospital, Nantes, France; 21 Service d’Hématologie-Immunologie-Oncologie, Hôpital des Enfants, CHU Toulouse, Toulouse, France; 22 Department of Pediatric Oncology, Essen University Hospital, Essen, Germany; 23 Department of Hematology, Oncology and of Cell and Gene Therapy, IRCCS Ospedale Pediatrico Bambino Gesú, Sapienza, University of Rome, Rome, Italy; 24 IntReALL study group, Berlin, Germany

**Keywords:** Drug development, Acute lymphocytic leukaemia

## Abstract

Inotuzumab Ozogamicin is a CD22-directed antibody conjugated to calicheamicin, approved in adults with relapsed or refractory (R/R) B cell acute lymphoblastic leukemia (BCP-ALL). Patients aged 1–18 years, with R/R CD22 + BCP-ALL were treated at the RP2D of 1.8 mg/m^2^. Using a single-stage design, with an overall response rate (ORR) ≤ 30% defined as not promissing and ORR > 55% as expected, 25 patients needed to be recruited to achieve 80% power at 0.05 significance level. Thirty-two patients were enrolled, 28 were treated, 27 were evaluable for response. The estimated ORR was 81.5% (95%CI: 61.9–93.7%), and 81.8% (18/22) of the responding subjects were minimal residual disease (MRD) negative. The study met its primary endpoint. Median follow up of survivors was 16 months (IQR: 14.49–20.07). One year Event Free Survival was 36.7% (95% CI: 22.2–60.4%), and Overall Survival was 55.1% (95% CI: 39.1−77.7%). Eighteen patients received consolidation (with HSCT and/or CAR T-cells therapy). Sinusoidal obstructive syndrome (SOS) occurred in seven patients. MRD negativity seemed correlated to calicheamicin sensitivity in vitro, but not to CD22 surface expression, saturation, or internalization. InO was effective in this population. The most relevant risk was the occurrence of SOS, particularly when InO treatment was followed by HSCT.

## Introduction

In pediatric patients with acute lymphoblastic leukemia (ALL), relapse still occurs in 10–15% of the subjects [1, 2]. Overall survival (OS) after relapse plateaus at 50–60%, while event free survival (EFS) after second and third relapse are approximately 25% and 15%, respectively [3–5]. Novel therapies are changing the treatment of children with relapsed/refractory (R/R) B-cell precursor ALL (BCP-ALL), with the approval of blinatumomab, a CD19-antigen directed T-cell engager, and Tisagenlecleucel, a CD19 chimeric antigen receptors (CAR) T-cell therapy. However, cases in which these agents are no longer effective were reported [6, 7]. Inotuzumab Ozogamicin (InO) consists of an anti-CD22 monoclonal antibody linked to the cytotoxic agent calicheamicin [8, 9]. InO is approved for adults with CD22-positive R/R BCP-ALL, based on the INO-VATE ALL trial [10]. CD22 is an antigen on the cell surface of most normal B-cells (60–90%) [11], and is expressed on the leukemic blasts in more than 90% of childhood BCP-ALL [12, 13]. Two retrospective studies of InO in pediatric BCP-ALL patients showed a remission rate of approximately 67% [14, 15]. The results of the phase I pediatric study from our group reported an Overall Response Rate (ORR) of 80% (95% CI: 59–93%) and, among the responders, 84% (95% CI: 60–97%) achieved minimal residual disease (MRD) negativity [16]. Recently, the Children’s Oncology Group (COG) reported a remission rate of 58.3% (90% CI: 46.5–69.3%), from 48 patients enrolled in a phase II trial using 1.8 mg/m^2^ [17]. A correlation between clinical response and CD22 alternative splicing and expression has been hypothesized. A study in Acute Myeloid Leukemia (AML) patients treated with gemtuzumab ozogamicin, a CD33 conjugate to calicheamicin, showed a link between alternative splicing of the CD33 antigen and clinical response [18], while an increased expression of a CD19 isoform with intraexonic splicing of exon 2 was found associated with treatment failure on blinatumomab [19]. Moreover, a trial in adults treated with the combination of chemotherapy and InO, with or without blinatumomab, identified baseline CD22 expression level <70% as predictor of poor outcome [20]. In this paper, we report the clinical results from the phase II InO single-agent ITCC-059 clinical trial and elaborate on the pharmacodynamic (PD) investigations on potential causes of intrinsic resistance to InO.

## Materials/Subjects and methods

ITCC-059 (EUDRACT nr 2016-000227-71; NTR5736) is a phase I-II, multicenter, international, single-arm, open-label study conducted in accordance with the International Ethical Guidelines for Biomedical Research Involving Human Subjects, International Council for Harmonization Guidelines for Good Clinical Practice, and the Declaration of Helsinki. The protocol received Ethics Committee review and approval at all participating centers. Patients were treated under protocol version 2 and 3 following an amendment unrelated to this cohort. Informed consent was obtained from all patients or their parents (as applicable) before enrollment. The study was sponsored by the Erasmus MC and funded by Pfizer inc. in the context of a Pediatric Investigational Plan.

### Patients and treatment

Criteria for enrollment (supplementary table 1) included age ≥1 to <18 years, diagnosis of CD22-positive R/R BCP-ALL with an M2 or M3 bone marrow (BM) status and refractory disease or ≥2^nd^ relapse, or any relapse post-hematopoietic stem cell transplantation (HSCT). Exclusion criteria included isolated extramedullary disease, active infections, and any history of prior or ongoing hepatic sinusoidal obstruction syndrome (SOS). Subjects started InO at the recommended phase II dose (RP2D) of 1.8 mg/m^2^/cycle fractionated in three weekly administrations, or 1.5 mg/m^2^/cycle once remission was achieved. Intrathecal prophylaxis was administered depending on the central nervus system (CNS) status. A maximum of six cycles were allowed, except for patients proceeding to HSCT for which the recommended number of cycles was two, or three if still MRD positive. Patients attaining an M1 BM with absolute neutrophil count (ANC) ≥ 0.5 × 10^9^/L and platelets count ≥30 × 10^9^/L, and those with M3 BM at study entry attaining an M2 BM irrespective of hematological criteria, could proceed to the subsequent cycles.

### Endpoints and statistical design

The primary objective was to establish the preliminary activity of InO. Secondary objectives included safety, other measures of antileukemic activity, PD analysis and pharmacokinetic (PK) parameters. PD analysis was performed on patients from phase I and II for whom material was available. The primary endpoint of the study was the ORR, defined as the combined Complete Remission (CR), CR with insufficient platelet recovery (CRp) and without recovery of counts (CRi) rate (supplementary table 2), and measured as best response during the entire treatment. Secondary endpoints included ORR after cycle one, EFS, OS, duration of response (DOR), MRD negativity rate and safety (supplementary table 3). MRD negativity was defined as either a PCR result below 10^−4^, or a flow cytometry result below 0.01% when the PCR was negative, but the quantitative range (QR) was above 10^−4^ (supplementary text 2) [21, 22]. PD parameters included the relationship between clinical response (MRD-negativity rate) and CD22 expression, saturation kinetics, CD22 clonal evolution, alternative splicing of the CD22 transcript, calicheamicin sensitivity, and the percentage of patients who exhibited anti-drug antibodies (ADA). The statistical design consisted of a single-stage design, based on exact binomial distribution. An ORR of ≤30% was considered not promising (null hypothesis, H_0_) and an ORR of >55% was expected (H_1_); 25 patients evaluable for response provided 80% power at a significance level of 0.05 (one-sided).

### CD22 expression levels

Flow cytometry was used to evaluate CD22 expression levels, by measuring both the mean fluorescent intensity (MFI) of leukemic blasts and the percentage of CD22-positivity at diagnosis on peripheral blood (PB) and BM samples at the Erasmus MC central Immunology laboratory in Rotterdam. Leukemic blast cells were gated based on expression of CD45, CD10, CD20, CD19, CD38, CD81, and CD34. The CD22 antibody RFB4® MHCD2204 (Thermo Fisher, Waltham, Massachusetts) was used for flow cytometry. In addition, CD22 saturation (Eq. 1 supplementary) and internalization (Eq. 2 supplementary) were measured on PB samples taken at day one and day eight of cycle one. The methods for the analysis of CD22 saturation and internalization of InO have been described previously [9].

### In-vitro drug response

In vitro drug response to calicheamicin (MedChemExpress, Monmouth Junction, New Jersey) was assessed with MTT assays in U-bottom 96-well plates. Patient samples from BM or PB were enriched to at least 80% leukemic blasts, based on morphology with a May-Grunwald-Giemsa staining, using a negative magnetic bead enrichment. The concentrations of calicheamicin on the MTT assay plates were tested in duplicates and ranged from 0.4 ng/ml to 400 ng/ml. MTT assays were performed over four days at 1.6 million cells/ml density using medium containing RPMI 1640 Dutch Modified with 20% Fetal calf serum, Penicilline, Streptavidine, and Fungizone. After four days >70% leukemic blast had to be present in the no-drug control wells to construct dose-response curves. The absorbance was read on a spectrophotometer at wavelengths of 562 nm and 720 nm and analyzed with Softmax Pro software. Optical density (OD) values after correction for blank wells of >50 were required. Metabolic activity was calculated at each drug concentration relative to control wells after correction for the background OD values of the blank wells. IC50 values represent the concentration of the drug which inhibits 50% of the leukemic cells [23].

### RNA sequencing, CD22 splice variants

The GENCODE reference annotations version 29 for GRCh38 and the GRCh38.p12 compliant Ensembl human genome reference were provided by the CTAT resource bundle (release: 27th of March 2019). Paired-end RNA-sequencing reads were aligned to this human genome reference and, subsequently, read counts per gene were calculated using STAR 2.6.0c. Split-reads were used to evaluate alternative splicing of the CD22 transcript. Only splice variants with at least 10 split reads were considered. Differential gene expression was assessed using TMM normalized counts and the generalized linear model from the EdgeR package in R statistics. Anti- and pro-apoptotic genes were selected based on the hallmark geneset of the GeneSet Enrichement Analysis software.

### Anti-drug antibody (ADA) analysis

Blood samples were collected during the screening, prior to each course of treatment, and at the end of treatment study. Samples were tested for ADA using a validated, electro chemiluminescent bridging assay.

### Statistical analysis

The response analysis set included all enrolled patients who received at least one dose of InO and completed at least one baseline and one post-baseline disease assessment. The full analysis set consisted of all enrolled patients who received at least one dose of study therapy and was used for the safety analysis. Detailed definitions of outcome measures are provided in supplementary table 4. EFS and OS were estimated using the Kaplan–Meier method. Events defined as non-response (not achieving CR, CRi or CRp, considered as event at day 0), relapse, death or second malignancy. For the analysis of PD parameters, patients were categorized into three groups: CR and MRD negative; CR and MRD positive; and no CR. For RNA sequencing analysis and calicheamicin sensitivity, subject not in CR and subject with CR MRD positive were grouped together, due to limited sample size. The Kruskal-Wallis test was used to test the association between the three response groups and the following PD parameters: CD22 surface expression (as MFI and percentage positive cells), CD22 saturation and InO internalization. The Wilcoxon rank-sum test was used to test calicheamicin sensitivity. As a post-hoc analysis, Fisher’s exact test and Mann-Whitney U test were used to test the association between clinical characteristics (eg. sex and age) and MRD response and between potential risk factors (eg. number of InO cycles received, time to HSCT) and SOS occurrence in post-InO transplanted patients. For all hypothesis tested, *p* values ≤0.05 were considered statistically significant. Statistical analyses were performed using R statistical software, version 4.1.3 (the code is available on request).

## Results

Results are based on a data cut-off date of 12 October 2021.

### Patients and treatment

Overall, 32 patients consented and were screened for inclusion from 03 June 2019 to 24 April 2020 at 16 sites of the ITCC consortium. In total, 30 patients were enrolled (two screening failures, both with inadequate liver function), 28 started treatment (two patients did not start treatment due to rapidly progressive disease), and 27 were evaluable (disease response not assessed in one patient, who discontinued due to SOS). Patient characteristics are reported in Table 1. A total of 147 doses of InO were given to 28 patients (median six doses/patient, range: 1–12). Thirteen (46.4%) subjects received one cycle, nine (32.1%) received two cycles, five (17.9%) received three cycles and one (3.6%) received four cycles.

**Table 1:** Patients’ characteristics.

Patients’ characteristics	Total (*n* = 28)
Male	19 (67.9%)
Female	9 (32.1%)
Median age in years at enrollment (IQR)	7.5 (4–13)
Age at enrollment breakdown	
>1 & ≤ 2 years	2 (7.1%)
>2 & ≤ 6 years	10 (35.7%)
>6 years	16 (57.1%)
**Extramedullary Disease (at screening)**^a^	
CNS1	21 (75%)
CNS2	4 (14.3%)
CNS3	2 (7.1%)
Testicular involvment	0
Lymph nodes enlarged	1 (3.6%)
Other locations (excluding spleen and liver)	0
**Diagnosis**	
first relapsed BCP-ALL post allogeneic HSCT	6 (21.4%)
second or greater relapsed BCP-ALL	16 (57.1%)
refractory BCP-ALL	6 (21.4%)
first HSCT prior to study treatment	14 (50.0%)
second HSCT prior to study treatment	1 (3.6%)
WBC (10^9^/L) at screening, median (IQR)	3.1 (2.3–9)
CD22 Peripheral blood blasts percentage, median (IQR)	96.7 (86.7–99.9)
Mean Fluoroscence Intensity - CD22 + expression, median (IQR)	2296.9 (1025.5–3709.2)
**Prior antibody therapy**	
Blinatumomab	7 (25%)
**Karyotype abnormalities**	
Normal	4 (14.3%)
Not Assessed/Available	9 (32.1%)
Hypodiploid (40–45 chromosomes)	2 (7.1%)
Low Hypodiploid (<40 chromosomes)	2 (7.1%)
Hyperdiploid (47–50 chromosomes)	2 (7.1%)
High hyperdiploid (51–65 chromosomes)	2 (7.1%)
Pseudodiploid	7 (25.0%)
t(9;22)(q34;q11.2) and variants	0
t(4;11)(q21;q23)	0
t(12;21)(p13;q22)	1 (3.6%)
t(11;v)(q23;v)	1 (3.6%)
t(1;19)(q23;p13)	1 (3.6%)
dic(9,20)(p11;q11)	1 (3.6%)
Down syndrome	0

^a^in one patient the sample was not evaluable due to red blood cells contamination.

*IQR*: Interquartile range.

### Efficacy

**Fig. 1:**
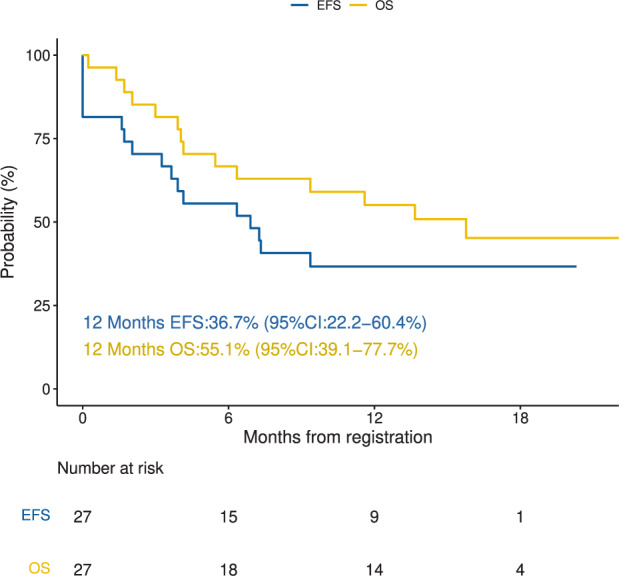
Event Free Survival and Overall Survival of patients treated in phase II. Blue line: Event Free Survival (EFS); Yellow Line: Overall Survival (OS). CI Confidence Interval.

Twenty-two patients achieved response (ORR 81.5%; 95%CI: 61.9–93.7%), in all cases after the first cycle; 14 were in CR, one in CRp and seven in CRi. MRD negativity, as best response, was achieved by 18 out of 22 (81.8%) responding subjects; after the first cycle by 13 (59.1%) patients, and after the second cycle by the other five. All patients were CNS negative at the end of cycle 1 and maintained the response at end of treatment. A total of 18 patients (66.7%) proceeded to consolidation therapy, 14 with HSCT (one after subsequent therapy with blinatumomab due to loss of response), two with CAR T-cell therapy (supplementary fig. 1), and two with CAR T-cell therapy followed by HSCT. Three patients received blinatumomab as bridging therapy before HSCT; one patient received chemotherapy; two received CAR-T as mentioned above; and the others did not receive additional treatment between the last InO administration and transplant. Median time between last InO dose and HSCT was 45 days (IQR:26.5–70.5). Median time between last InO dose and CAR-T therapy was 53.5 days (IQR:46.5–260.75). In two cases lymphocyte apheresis for CAR T-cell therapy was performed before initiating InO and in the other two cases after. Of the other four responding subjects, one proceeded to blinatumomab (in continuous CR after seven months), one received maintenance therapy with 6-mercaptopurine and intrathecaltriple therapy (relapsed after seven months), one relapsed within one month after end of treatment, and one patient died while in CR due to neurological deterioration attributable to previous therapy and CNS leukemic involvement. The median follow for survival was 16 months (IQR: 14.49–20.07). At six months, EFS probability was 55.6% (95% CI: 39.6–77.8%) and OS was 66.7% (95% CI: 51.1–87.0). At 12 months, EFS probability was 36.7% (95% CI: 22.2–60.4%) and OS probability was 55.1% (95% CI: 39.1–77.7%) (Fig. 1). Median DOR was 7.74 months (95% CI: 5.65-not reached). The cumulative incidence of non-response or relapse was 29.63% (95% CI 13.77–47.42%) at six months and 40.74% (95% CI 22.03–58.69%) at 12 months. Combining phase I (*n* = 25) and II (*n* = 27) results, 52 patients received InO, of which 40 were treated at the RP2D (13 in phase I and 27 in phase II) [16]. Considering the combined response data from patients treated at the RP2D, 33 achieved response (ORR 82.5%; 95%CI: 67.2–92.7%), and 27 (81.8%) of the responders were MRD negative. EFS at 12 months was 41.3% (95%CI: 28.3–60.1%); and OS at 12 months was 56.3% (95%CI: 42.6–74.3%) (supplementary fig. 2). In patients treated at RP2D (*N* = 40), age, gender, previous treatments, WBC, and CD22 MFI were not found to impact the ORR, MRD-negative response (supplementary table 5) or EFS (Table 2). In total, 17 patients relapsed (11 in phase I and 6 in phase II) and for 10 of them CD22 expression data were available. CD22 expression turned negative in three cases, and partially negative in two.

**Table 2:** Univariable Cox model for EFS of all patients treated at RP2D (*N* = 40 patients, phase I and II combined, 25 events).

Variable name	Events	Number patients	HR	Lower 95% CI	Upper 95% CI	Level P	*p* value
**Age at enrollment (in year)**	25	40	1.191	0.542	2.617	0.6643	0.66
**Sex**							0.47
male	18	29	1				
female	7	11	1.377	0.574	3.305	0.4735	
**Diagnosis**							0.17
first relapse post HSCT	6	10	1				
2nd or greater relapsed	13	23	1.052	0.399	2.775	0.9179	
refractory	6	7	2.452	0.783	7.681	0.1236	
**Prior HSCT**							0.19
no	15	20	1				
yes	10	20	0.589	0.264	1.314	0.1964	
**Prior antibody therapy (blinatumomab)**							0.22
no	18	31	1				
yes	7	9	1.713	0.714	4.108	0.2279	
**Peripheral Blood WBC at screening (10**^**9**^**/L)**							0.62
	25	40	0.821	0.374	1.806	0.6246	
**Mean Fluoroscence Intensity - CD22** + **expression**							0.35
	24	38	0.681	0.304	1.526	0.3505	

### Safety

All patients (*n* = 28) had at least one adverse event (AE), 20 (71.4%) at least one grade 3–4 AE. The most common AE was fever (*n* = 16, 57.1%). Five (17.6%) patients had infection of grade ≥3, and 6 (21.4%) had febrile neutropenia. All patients had at least one grade 3–4 hematologic laboratory test abnormality (neutropenia being the most common, *n* = 26, 92.9%). Only four patients stll had thrombocytopenia grade 3/4 at C1D22, of which one after day 42. Details are provided in supplementary Table 7–9. A total of 26 serious adverse events were observed in 17 (60.71%), patients (supplementary table 10). Seven (25%, *n* = 28) cases of SOS were reported; one grade 2, four grade 3 and two grade 4 (the latter six classified as “severe” according to the EBMT criteria) [24]. Six cases occurred after HSCT post-treatment with InO, four resolved after treatment with defibrotide, and two might have contributed to death due to multi organ failure and infection. Another case of SOS occurred after one dose of InO in a patient treated due to relapse three months after HSCT. SOS resolved completely following administration of defibrotide. Including the phase I part of the study, nine SOS cases occurred in 52 treated patients (17.3%), of which six in the 23 transplanted patients (26.1%) (supplementary table 6). In a post-hoc analysis, when considering patients who developed SOS subsequently to transplant post-InO and those who did not, the median time interval between last InO dose and HSCT was shorter in patients developing SOS (24.5 (*n* = 6) vs 54.5 days (*n* = 17), *p* = 0.01). The number of InO cycles received, previous HSCT, defibrotide prophylaxis, conditioning regimen with total body irradiation and dose level, were not statistically significant (supplementary table 6). No cases of toxic death considered related to InO were observed during study treatment. One patient died of encephalopathy considered attributable to prolonged intrathecal chemotherapy due to CNS leukemic involvement. Five additional non-relapse deaths occurred after HSCT due to multiple complications. The cumulative incidence of relapse was 29.63% (95% CI 13.77–47.42%) at six months, and 40.74% (95% CI 22.03–58.69%) at 12 months. The cumulative incidence of non-relapse death was 14.81% (95% CI 4.47–30.94%) at six months, and 22.59% (95% CI 8.8–40.23%) at 12 months, including post-HSCT follow-up (supplementary fig. 3).

### Pharmacodynamics

**Fig. 2:**
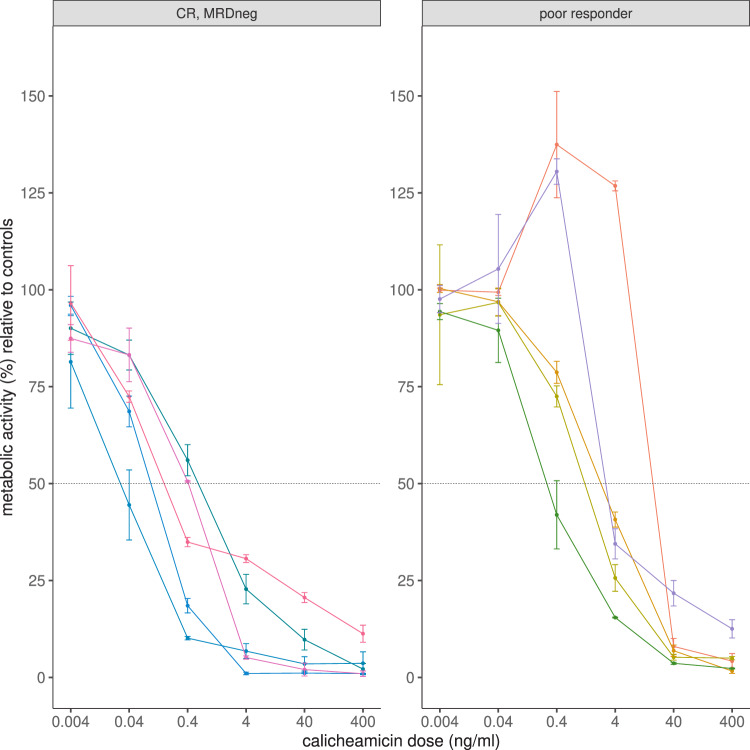
Dose-response curves for calicheamicin based on MTT assays. Each color represents a different patient. The intersection with the black line at 50% represents the IC50 value. IC50: concentration of drug required for 50% inhibition; MRD neg: minimal residual disease <10^−4^; CR: complete response; poor responders: no CR and/or MRD ≥ 10^−4^. The median IC50 value for all patients was 0.75 ng/ml (range 0.035–27.27; *n* = 10), median 0.26 ng/ml (range 0.035–1.05; *n* = 5) in the good responders (left panel) and median 3.12 ng/ml (range 0.34–27.27; *n* = 5) in the poor responders (right panel) (*p* = 0.032). From the literature, the median calicheamicin sensitivity in AML cells was 4.8 ng/ml, ranging between 0.1–1000 ng/ml (de Vries JF, et al.; 2012).

**Fig. 3:**
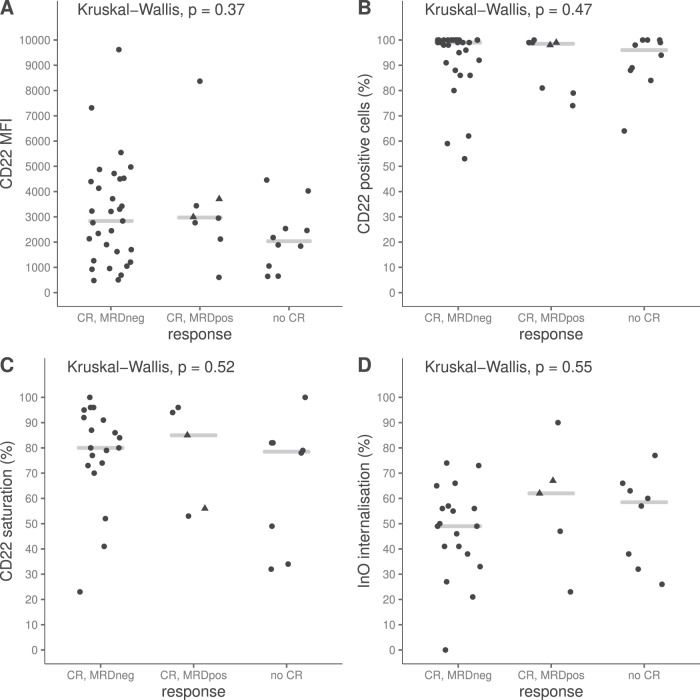
CD22 expression on BM blasts at baseline, saturation and InO internalization on leukemic PB blasts post infusion on day one. Presented as Mean Fluorescence Intensity (MFI) (**A**), percentage CD22-positive cells (**B**), saturation (**C**) and internalization (**D**). Grey horizontal lines represent the median value per group. In all four parameters, there were no statistically significant differences between the response groups as defined in the statistical methods for the PD analysis. Triangles represent patients with PCR-MRD quantitative range >10^−4^.

In vitro sensitivity to calicheamicin was available for 11 patients, of which 10 had MRD data available. In the latter group, MTT assays were performed, nine from BM and one from PB. IC_50_ values for calicheamicin ranged from 0.035 to 27.27 ng/ml. The median was twelve times higher in the five MRD-positive patients compared to MRD-negative patients (3.12 ng/ml vs 0.26 ng/ml, *p* = 0.032, *n* = 10; Fig. 2). Furthermore, patients with IC_50_ values above the median seemed to have a poorer EFS (supplementary fig. 4), despite not statistically significant (*n* = 5, *p* = 0.19). Nevertheless, four of the five poor responders were treated at 1.4 mg/m^2^/cycle in phase I (*p* = 0.048). The association of CD22 expression on leukemic blasts in BM samples obtained at baseline with response to InO was not statistically significant, neither the mean fluorescence index (MFI, range = 479–9619, *p* = 0.37, *n* = 49, Fig. 3A), nor the percentage of CD22-positive cells (range = 53–100%, *p* = 0.47, *n* = 49, Fig. 3B). Additionally, neither the level of saturation of CD22 antigens on PB leukemic blasts after the InO dose (samples taken prior and after infusion at day one) (range = 23–100, *p* = 0.52, *n* = 32, Fig. 3C), nor the level of internalization of InO after the first InO dose (range = 0–90, *p* = 0.55, *n* = 32, Fig. 3D) were associated to response. Alternative splicing was assessed by the number of split reads that included or excluded particular exons. The most prevalent alternative splicing variants of CD22, observed in our samples, were further analyzed. Multiple splicing isoforms involving the skipping of at least exon 2, which contains the start-codon for CD22 translation, were observed in all patients (supplementary fig. 5). The splice variant of the CD22 transcript with exclusion of exons two to six (Δex2to6-CD22), encoding part of the extracellular domain, was seen most frequent with variable expression levels among patients. No correlation between alternative splicing of exon 2 and response was seen. Additionally, we did not find any association between the skipping or inclusion of exon 2 in the CD22 transcript and the expression of CD22 antigen on leukemic blasts, the saturation levels of CD22 on leukemic blasts with InO, or the internalization levels of InO (supplementary fig. 6). Skipping of exon 5 and 6 was seen in all patients (supplementary fig. 7), but did not influence the expression of CD22 antigen on leukemic blasts, the saturation levels of CD22 on leukemic blasts with InO, or the internalization levels of InO (supplementary fig. 8). Skipping of exon 12, suggested to negatively affect internalization of InO [25], was found in all patients (*n* = 9, supplementary fig. 9), but did not seem to affect internalization levels (supplementary fig. 10). RNA sequencing of leukemic cells was performed on nine patients with available material. Since calicheamicin acts by causing DNA double-strand breaks, leading to apoptosis of the cells, we looked at the expression of various anti- and pro-apoptotic genes, including *BCL2* gene family members [9]. No significantly different expression of apoptotic genes was seen in the leukemic cells of patients MRD negativity and those MRD positive (supplementary fig. 11). Among the 52 patients treated in phase I and II, one (1.9%) patient had positive ADA (titer ≥2.30) against InO at baseline. The patient was treated at DL1 in Phase I and did not respond to InO. The presence of positive ADA at baseline was likely due to pre-existing host antibodies that were cross-reactive with InO and seemed not to impact on the PK. No treatment-boosted ADA responses were identified.

## Discussion

This phase II study provides further evidence for the activity of InO in R/R BCP-ALL pediatric patients. No clinical characteristic were found related to ORR or EFS (supplementary 5). InO was generally well tolerated, with a low incidence of infections during treatment (17.8%). SOS remains the most serious AE (25%, *n* = 7), although mostly occurring after subsequent HSCT and only occasionally while on treatment. Most SOS cases (5/7) resolved completely and no cases of toxic death considered related to InO or deaths in CR due to infections were observed during study treatment. Combining data from phase I (all dose levels) and II cohorts, SOS occurred in 26.1% of the patients transplanted post-InO, which is significantly lower than reported by Bhojwani et al. (52%, 11/21) but in line with data from O’Brien et al. (28.6%, 6/21), all treating patients at 1.8 mg/m^2^ [14, 17]. Median time since the last InO dose appeared to be statistically significantly shorter in patients who developed SOS post HSCT. A possible explanation might be the long half-life of InO (12 days), and the inverse relationship between tumor load and the time dependent component of its clearance, generally longer after few weeks of treatment [26]. Therefore, InO might still be circulating during the first month after treatment, particularly in patient achieving CR early on treatment. The use of prophylactic defibrotide was left at investigators’ discretion, therefore not uniformly performed. This makes difficult to assess its impact as a protective factor for SOS in this small cohort. Taken together, the relationship between risk factors and SOS occurrence should be investigated in larger series. As suggested in other studies, the sensitivity of ALL cells to calicheamicin might contribute to the achievement of MRD negativity [9]. When considering all patients from phase I and II, including those treated at 1.4 mg/m^2^, MRD negativity was found to correlate to calicheamicin sensitivity in vitro but not to CD22 surface expression, saturation, or internalization. Although CD22 expression is needed for binding of InO, high levels of CD22 expression on leukemic blasts, saturation of CD22 with InO and internalization of InO do not appear to be crucial for clinical response to InO. Our findings are in line with previous in vitro studies from our group on BCP-ALL cells which showed that, although CD22 expression was essential for InO binding, efficacy was not dependent on CD22 expression levels, while a clear correlation with calicheamicin sensitivity was noticed [9]. In contrast, COG reported lower baseline CD22 density (measured as antibody bound per cell), and a reduction of CD22 percentage over time in the poor responders [17]. Nevertheless, they did not find CD22 percentage at baseline as significant, but only four subjects had a CD22 expression <90%. Instead, CD33 expression on leukemic blasts does affect clinical response of AML patients to gemtuzumab ozogamicin, [18, 27, 28]. This may be due to AML cells being less sensitive to calicheamicin, higher levels of CD33 on leukemic blasts, higher levels of CD33 saturation, and a continuous loop of internalization and renewed expression of CD33 antigens might be required for a sufficient accumulation of calicheamicin inside AML cells; whereas in ALL cells lower levels of accumulated calicheamicin might be sufficient to cause apoptosis [9]. In adults treated with chemotherapy and InO, baseline CD22 expression <70% was independently associated with worse survival, while ORR and MRD negativity rate could not be identified as significantly different in the two groups [20]. Moreover, a significant correlation between higher median baseline CD22 levels and achievement of MRD-negative CR was found in patients treated with anti-CD22 CAR T-cells [29]. In our cohort, no correlation between CD22 expression and ORR and MRD was found, but only a limited number of patients (*n* = 4) had a CD22 expression <70%. CD22 exon 2 seems to be crucial for both initiating RNA translation into a protein product and for the binding of CD22-directed antibody [30]. One patient with only the Δex2 CD22 splice isoform has been reported resistant to InO [30]. In our study, alternative splicing of CD22 was observed in all patients, especially the Δex2–6 variant, but did not correlate with response, probably because all patients had at least some normal CD22 expression. Skipping of exon 5–6 (binding region of RFB4 antibody) was observed only in a limited percentage which might explain why it did not correlated to CD22 MFI. Similarly, skipping of CD22 exon 12, previously reported as potentially reducing InO internalization [25], occurred in all patients samples in our cohort (*n* = 9) at various extent, but did not correlate to lower internalization levels. These findings underscore that high levels of full-length CD22 might not be necessary to respond to InO. Nevertheless it is worth noting that CD22 surface expression was tested before study inclusion by using anti-CD22 RFB4 antibody and only CD22-positive cases were included. Our findings confirmed evidence of CD22 negative/dim relapses after treatment with anti-CD22 CAR T-cells, suggesting CD22 downmodulation as possible mechanism of acquired resistance [29]. A RNA sequening analysis pre- and post-relapse, which could better highlight the mechanism of CD22 downmodulation, was not performed. Previous studies suggested gene expression as possible cause of resinstance to calicheamicin, but we did not find significant differences between the response groups in the expression of known genes of the apoptotic pathway such as BCL2, albeit in a small sample size [31, 32]. InO is currently tested in front-line treatment by the COG in a phase III randomized trial for high-risk CD22-positive BCP-ALL (NCT03959085), and in the ALLTogether1 protocol, for patients stratified to the intermediate-high risk group (NCT03911128). The ITCC-059 study is ongoing, testing InO in combination with chemotherapy in R/R pediatric ALL and as single agent in very high risk first relapse ALL.

## Supplementary information

Supplementary Material

Study ITCC-059 Protocol
