# Supplementary material for: Inotuzumab ozogamicin as single agent in pediatric patients with relapsed and refractory acute lymphoblastic leukemia: results from a phase II trial
Source: Leukemia. 2022 Apr 25;36(6):1516–24. doi: 10.1038/s41375-022-01576-3 (PMC9162924; doi:10.1038/s41375-022-01576-3)
Supplement: Supplementary file 1 — Supplementary Material [file 41375_2022_1576_MOESM1_ESM.pdf]

SUPPLEMENTARY MATERIAL

Supplementary Table 1. Inclusion and Exclusion Criteria

Inclusion Criteria	
**Age**	• ≥1 and <18 years at time of enrollment<br>• The first three patients on dose level 1 must be ≥6 and <18 years<br>• Then ≥2 additional patients ≥1 year and <6 years at the same dose level
**Diagnosis**	• First relapse of BCP-ALL post allogeneic HSCT<br>• Second or greater R/R BCP-ALL<br>• Refractory disease (newly diagnosed patients who had induction failures after ≥2 previous regimens without attainment of remission, or patients with refractory first relapse after one previous reinduction regimen without attainment of remission) **AND**:<br>• M2 or M3 marrow status (≥5% blasts by morphology)<br>• Malignant clone CD22 surface antigen positive (in either bone marrow or peripheral blood) by institutional standards and measured by the routine diagnostic method of the local laboratory and reported as positive or negative according to the local interpretation of the data (no specific cut-off was used).<br>• The first six patients must have M3 marrow status (≥25% blasts by morphology)
**Performance level and life expectancy**	• Karnofsky >60% (>16 years) or Lansky >60% (≤16 years)<br>• Life expectancy of ≥6 weeks
**Prior therapy**	Patients must have recovered from the acute toxic effects of all prior therapy, defined as resolution of non-hematologic toxicities to ≤Grade 2 per the CTCAE 4.03 prior to entering the study<br><br>a. <u>Chemotherapy</u><br>≥7 days since the completion of cytotoxic therapy (exceptions: hydroxyurea, 6-mercaptopurine and steroids which are permitted up until 48 hours prior to initiating protocol therapy)<br><br>b. <u>Radiotherapy</u><br>≥28 days since any prior radiation therapy<br><br>c. <u>Hematopoietic stem cell transplant</u><br>≥90 days since previous allo-HSCT<br>No evidence of active graft vs host disease No GVHD prophylaxis or treatment<br><br>d. <u>Hematopoietic growth factors</u><br>≥7 days since the completion of therapy with GCSF or other growth factors, or ≥14 days since completion of therapy with pegfilgrastim (Neulasta®)<br><br>e. <u>Immunotherapy</u><br>≥42 days after the completion of any type of immunotherapy, e.g. CART therapy. Patients may not have received prior CD22-targeted therapy (immunotoxin or CART therapy)<br><br>f. <u>Monoclonal antibodies</u><br>≥3 half-lives of the antibody must have elapsed after the last dose of a monoclonal antibody (rituximab = 66 days, epratuzumab = 69 days) Exclusion of blinatumomab: patients must have been off<br>blinatumomab infusion for ≥14 days and all drug-related toxicity must have resolved to ≤Grade 2<br><br>g. <u>Investigational drugs</u><br>≥7 days or five drug half-lives (whichever is longer) since prior treatment with any experimental drug (with the exception of monoclonal antibodies) under investigation. No residual toxicities should be observed following previous treatment<br><br>h. <u>Prior calicheamicin exposure</u><br>Patient has not received prior treatment with a calicheamicin conjugated antibody (e.g. gemtuzumab ozogamicin)

**Renal and hepatic function**	• Serum creatinine ≤1.5 x institutional ULN according to age<br>• AST and ALT ≤2.5 x institutional ULN<br>• Total bilirubin ≤1.5 x institutional ULN unless the patient has documented Gilbert syndrome
**Cardiac function**	• Shortening fraction ≥30% by echocardiogram or an ejection fraction >50% by MUGA.
**Reproductive function**	• Female patients of childbearing potential: negative urine or serum pregnancy test confirmed prior to enrollment<br>• Female patients with infants must agree not to breastfeed on study<br>• Male and female patients of child-bearing potential must agree to use a *highly effective* method of contraception (≥8 months for females and for ≥5 months for males after the last dose of InO)

**Exclusion Criteria**	
**Isolated extramedullary relapse**	• Patients with isolated extramedullary disease are excluded
**VOD/SOS**	• Any history of prior or ongoing VOD/SOS as per modified Seattle criteria, or prior liver-failure [defined as severe acute liver injury with encephalopathy and impaired synthetic function (international normalized ratio of ≥1.5)]
**Infection**	• Systemic fungal, bacterial, viral or other infection that is exhibiting ongoing signs/symptoms<br>• The patient may not have:<br>• A requirement for vasopressors<br>• Positive blood culture within 48 hours of study enrollment<br>• Fever above 38.2 degrees Celsius within 48 hours of study enrollment with clinical signs of infection. Fever that is determined to be due to tumor burden is allowed, with documented negative blood cultures for ≥48 hours prior to enrollment and no concurrent signs or symptoms of active infection or hemodynamic instability<br>• A positive fungal culture within 30 days of study enrollment<br>• Active fungal, viral, bacterial, or protozoal infection requiring intravenous or oral treatment. Chronic prophylaxis therapy to prevent infections is allowed
**Other anti-cancer therapy**	• Patients will be excluded if there is a plan to administer non-protocol anti-cancer therapy during the study period
**Allergic reaction**	• Patients with prior Grade 3/4 allergic reaction to a monoclonal antibody are excluded
**Concurrent disease**	• Significant concurrent disease, illness, psychiatric disorder or social issue that would compromise patient safety or compliance with protocol therapy, interfere with consent, study participation, followup, or interpretation of study results<br>• Children with Down syndrome are excluded from participation in the dose finding parts of the study

ALT, alanine aminotransferase; AST, aspartate aminotransferase; BCP-ALL, B-cell precursor acute lymphoblastic leukemia; CART, chimeric antigen receptor T cell; GCSF, granulocyte-colony stimulating factor; GVHD, graft versus host disease; HSCT, hematopoietic stem cell transplant; InO, inotuzumab ozogamicin; MUGA, multiple gated acquisition scan; R/R, relapsed/refractory; SOS, sinusoidal obstruction syndrome; ULN, upper limit of normal; VOD, veno-occlusive disease.

Supplementary Table 2. Definitions of Response

Response Category	Criteria*
**Complete Response**	No evidence of circulating blasts or extramedullary disease; including CNS-1 status; absence of splenomegaly, lymphadenopathy, skin/gum infiltration, testicular mass<br>A bone marrow with <5% blasts (M1 marrow)<br>Recovery of peripheral counts (platelets >50,000/µL and transfusion independent, and ANC >500/µL)
**Complete Response with Insufficient Platelet Recovery**	No evidence of circulating blasts or extramedullary disease<br>A bone marrow with <5% blasts (M1 marrow)<br>An ANC > 500/µL but Platelets ≤ 50,000/µL
**Complete Response without Recovery of Counts**	No evidence of circulating blasts or extramedullary disease<br>A bone marrow with <5% blasts (M1 marrow)<br>An ANC ≤ 500/µL and / or Platelets ≤50,000/µL
**Partial Response**	Greater than 50% relative reduction (with a minimum of 10% absolute reduction) in the bone marrow aspirate leukemic cell count, irrespective of recovery of the peripheral blood counts
**Stable Disease/ No Response**	Stable disease is present when the patient fails to qualify for CR, CRi, PR, or PD
**Progressive Disease**	Progressive disease is defined as an increase of at least 25% of the absolute number of bone marrow or circulating leukemic blasts, development of extramedullary disease, or other laboratory or clinical evidence of PD, with or without recovery of ANC or platelets
**Relapse**	After documentation of remission, a bone marrow aspirate and/or biopsy showing ≥5% leukemic blasts using morphology with flow cytometric confirmation, and/or pathological/radiological evidence of extramedullary disease, including development of CNS3 status or clinical CNS-involvement with radiological confirmation (MRI).
**Refractory**	Any patient not achieving CR, CPp or CRi after induction therapy (cycle 1 and/or cycle 2).

ANC: Absolute Neutrophile Count; CNS 1/2/3: Central Nervous System Disease Status; CR: Complete Response; CRi: Complete Response without Recovery of Counts; CRp: Complete Response with Insufficient Platelet Recovery; PD: Progressive Disease; SD: Stable Disease.

* All criteria must be present to define each response category

Supplementary Table 3. Study Endpoints

**Primary endpoints**
ORR, defined as the percentage of patients with CR, CRi, CRp, measured as best response during InO treatment
**Secondary endpoints**
**1. Safety:**<br>• AEs, as characterized by type, frequency, severity (as graded using CTCAE v4.03), timing, seriousness, and relation to study therapy, during the first and subsequent cycles of therapy.<br>• Occurrence of toxic death; i.e., death attributable to InO therapy.<br>• Occurrence of VOD/SOS during or after therapy with InO.<br>• Laboratory abnormalities as characterized by type, frequency, severity and timing.<br>• The cumulative incidence of non-relapse mortality, defined as the cumulative probability of non-relapse mortality, with time calculated between start of study treatment and death due to other causes than relapsed or refractory leukemia or lymphoma, accounting for competing events.<br><br>**2. Other measures of anti-leukemic activity:**<br>• ORR after cycle 1.<br>• Minimal residual disease levels, including the percentage of patients who become MRD-negative (complete MRD response defined as an MRD-level < $1x10^{-4}$), after cycle 1, as well as the best response (MRD-negativity) over multiple cycles.<br>• Duration of response, defined as the time between achieving response (CR, CRi or CRp) after starting study treatment and documented relapse or death.<br>• Number and percentage of patients being transplanted and those receiving CAR T-cell therapy after treatment with InO.<br>• EFS, defined as the time between start of study treatment and first event including failure to achieve CR/CRp/CRi (calculated as an event on day 0), relapse, death of any cause and second malignancies.<br>• Survival, defined as time to death following start of study treatment.<br>• The cumulative incidence of non-response or relapse, defined as the cumulative probability of non-response or relapse, with time calculated between start of study treatment and relapse and with non-responders included as an event on day 0. Non-relapse death is considered a competing event.<br><br>**3. Serum pharmacokinetic parameters of InO and unconjugated calicheamicin.**<br><br>**4. Pharmacodynamics parameters***<br>• Relationship between response (ORR) and CD22 expression levels and WBC.<br>• Relationship between response (ORR) and CD22 saturation kinetics.<br>• Relationship between response (ORR) and calicheamicin sensitivity.<br>• Clonal evolution (CD22-negativity) and relation to loss of response.
**Other endpoints**
The percentage of patients responding to InO (ORR) without adequate recovery of CD19-positive B-cells (below LLN for age) or immunoglobulins (below LLN for age) following 4 weeks, 10 weeks, 3, 6 and 12 months after treatment with InO, excluding patients who have been transplanted from the date of HSCT or have received CAR-T cells therapy.<br><br>Percentage of patients who exhibit ADA

LLN: lower limit for normality; ADA: anti-drug antibodies; WBC: with blood cells; ORR: overall response rate; MRD: Minimal residual disease; SOS/VOD: Sinusoidal occlusive syndrome/veno-occlusive disease; EFS: event free survival; HSCT: Hematopoietic stem cell transplantation. * in post hoc analysis we also investigated the relationship between PD parameters and achievement of MRD negativity as defined in the main paper.

Supplementary Table 4: Expanded definitions of outcome measurements

Term	Definition
**Overall response rate (ORR)**	defined as complete remission (CR), CR with incomplete hematologic recovery (CRi), or CR with incomplete platelet recovery (CRp; response criteria followed standard procedures in leukemia, as designed by the National Comprehensive Cancer Network, with slight modifications in relation to bone marrow regeneration [CR = platelets >30,000/µL rather than >100,000/µL; and ANC >500/µL rather than >1000/µL]).
**Minimal Residual Disease (MRD) status:**	bone marrow negative if MRD $<1x10^{-4}$ with real-time quantitative-PCR or <0.01% with multi-parameter flow cytometry according to EuroFlow protocols when PCR was negative but the QR was $> 10^{-4}$.[1,2]
**Event-free survival (EFS)**	defined as time from start of treatment to first event including failure to achieve CR/CRp/CRi, relapse, death, and second malignancies.
**Cumulative incidence of non-response or relapse**	defined as the cumulative probability of non-response or relapse, with time calculated between start of study treatment and relapse and with non-responders included as an event on Day 0. Non-relapse death is considered a competing event.
**Cumulative incidence of non-relapse mortality**	defined as the cumulative probability of non-relapse mortality, with time calculated between start of study treatment and death due to other causes than relapsed or refractory leukemia or lymphoma, accounting for competing events.
**Duration of response**	defined as the time between achieving response (CR, CRi or CRp) after starting study treatment and documented relapse or death
**Overall survival (OS)**	defined as time to death following start of study treatment.

ADDITIONAL SPECIFICATIONS ON THE METHODOLOGY

Supplementary text 1. CD22 saturation and internalization

Saturation was defined as (Eq. 1)

$$\frac{(\text{specific fluorescence intensity of bound Ino})}{(\text{specific fluorescence intensity maximal Ino binding})} \cdot 100\%$$

Internalisation was defined as (Eq. 2)

$$1 - \left(\frac{\text{specific fluorescence intensity of bound Ino}_{\tau+1}}{specific\ fluorescence\ intensity\ maximal\ Ino\ binding_{\tau}}\right) \cdot 100\%$$

Where τ stands for time. Data were acquired on a FACSCanto flow cytometer (BD Biosciences) using EuroFlow instrument settings and analyzed using DIVA (BD Biosciences) and Infinicyt (Cytognos) software.

Supplementary text 2: Determination of MRD levels by PCR and Flowcytometry

Molecular MRD levels were centrally determined by RQ-PCR of leukemia-specific rearranged immunoglobulin (IG) and T-cell receptor (TR) genes (van der Velden and Van Dongen, 2009). Quality control and standardized interpretation of RQ-PCR data were achieved following the guidelines of the European Study Group on MRD detection in ALL (EuroMRD) (Van der Velden et al, Leukemia 2007). For flowcytometric MRD analysis, also centrally performed, bone marrow samples were bulk-lysed and subsequently stained using 8 color stainings according to EuroFlow protocols (Theunissen et al, Blood 2017; Kalina et al, Leukemia 2012). Four million cells (if available) were acquired and MRD positivity was defined if at least 20 ALL cells could be detected. Flow MRD negativity was defined as MRD < 0,01% using an assay with a sensitivity of at least 0,01%. MRD negativity was defined as PCR below 10^{-4} or flow cytometry below 0.01% when PCR was negative but the Quantitative Range was above 10^{-4}.

Supplementary Table 5: Risk factors analysis for MRD response of all patients treated at RP2D (N= 40 patients, phase I and II combined)

		MRD neg	MRD pos	P value
**Age at enrollment**				0.73
	<10 years	13	7	
	>=10 years	14	5	
**Sex**				0.26
	male	21	7	
	female	6	5	
**Diagnosis**				0.27
	first relapse post allogeneic HSCT	7	2	
	second or greater relapsed	17	6	
	refractory	3	4	
**Prior HSCT**				0.73
	no	13	7	
	yes	14	5	
**Prior antibody therapy (blinatumomab)**				0.42
	no	22	8	
	yes	5	4	
**PB WBC at screening**				0.73
	<median	13	7	
	>=median	14	5	
**IC50 Calicheamicin (N=10)**				0.21
	<median	4	1	
	>=median	1	4	

HSCT: Hematopoietic Stem cell transplant; PB WBC: Peripheral Blood With Blood Count; IC50: values represent the concentration of the drug which inhibits 50% of the leukemic cell

Supplementary Table 6. SOS cases in patients receiving transplantation after InO for combined cohort of phase I and phase II

Age at enrollment	HSCT Conditioning regimen	Defibrotide prophylaxis	Previous HSCT	Days since last InO dose	Number of InO courses	Dose level* (mg/m^2)	SOS	Grade	Outcome SOS
6	Etoposide; TBI	no	no	125	1	1.8	no		
4	Fludarabine; Thiotepa; treosulfan	no	no	83	1	1.4	no		
14	Fludarabine; busulfan; Thiotepa	na	no	99	1	1.8	no		
12	Etoposide; TBI	no	no	26	2	1.4	no		
11	Fludarabine; treosulfan; Thiotepa	no	no	23	2	1.4	no		
15	Etoposide; TBI	na	no	39	2	1.8	no		
16	Fludarabine; melphalan; Thioptepa	na	yes	51	1	1.8	no		
4	Etoposide; TBI	yes	yes	55	2	1.8	no		
13	Etoposide; TBI	yes	no	47	3	1.8	yes	3	Resolved
6	Fludarabine; Thiotepa; TBI	no	no	72	4	1.8	no		
12	Fludarabine; busulfan; Thiotepa	no	yes	54	3	1.8	no		
2	Busulfan; fludarabine; Thiotepa	yes	no	27	2	1.8	yes	3	Resolved
1	Fludarabine;treosulfan; Thiotepa	yes	no	35	2	1.8	no		
8	Treosulfan;fludarabine; Thiotepa	no	yes	20	1	1.8	yes	3	Ongoing at time of death (due to MOF)
14	Fludarabine; TBI	yes	no	22	2	1.8	no	3	Resolved
14	Fludarabine; TBI	yes	no	22	2	1.8	yes	2	Resolved
13	Etoposide; TBI	yes	yes	110	1	1.8	no		
5	Etoposide; TBI	no	no	25	3	1.8	no		
7	Thiotepa; fludarabine	no	no	70	2	1.8	no		

Age at enrollment	HSCT Conditioning regimen	Defibrotide prophylaxis	Previous HSCT	Days since last InO dose	Number of InO courses	Dose level* (mg/m^2)	SOS	Grade	Outcome SOS
7	Etoposide; TBI	no	no	182	2	1.8	no		
13	Etoposide; TBI	no	yes	78	2	1.8	no		
4	Etoposide; TBI	yes	yes	35	1	1.8	yes	4	Resolved
8	Etoposide; TBI	yes	yes	43	1	1.8	no		
17	Etoposide; TBI	yes	no	22	3	1.8	yes	4	Ongoing at time of death (due to MOF)

To identify the risk factors for SOS, Fisher's exact test (for categorical variables) and Mann-Whitney U test (for continuous variables) were used to compare the patients who received transplantation after Inotuzumab Ozogamicin (InO) (6 who later developed SOS vs 18 who did not). Days from last InO dose was found to be statistically significant with a p value of 0.014. The median value of days from last InO dose in patients with SOS was 24.5 (IQR: 21.5-38) while the median in patients with no SOS was 54.5 (IQR: 30.5-75). Dose level 1.4 mg/m2 refers to phase I only. Conditioning regimen with TBI (p=1.0), age at enrollment (p=0.84), defibrotide prophylaxis (p=0.06), previous HSCT (p=1.0), and number of InO courses received (p=0.69) were not statistically significant. SOS: sinusoidal obstruction syndrome; HSCT hematopoietic stem cell transplantation; TBI: total body irradiation; MOF: multi organ failure. SOS was diagnosed based on Seattle criteria and was graded according to the CTCAE v. 4.03 under hepatobiliary disorders, and AE term: Other, specify.

Supplementary Table 7: AEs listing Grade 3 and 4 during phase 2 (N=28) reported by the local investigators as clinically significant (worst grade per term per patient)

AE term	Grade	3	4	Total
Alkaline phosphatase increased		1	0	1
Anal mucositis		1	0	1
Anal pain		1	0	1
Arthralgia		1	0	1
Aspartate aminotransferase increased		5	0	5
Blood bilirubin increased		1	1	2
Cardiac disorders - Other specify: Hypotension		1	0	1
Catheter related infection		1	0	1
Constipation		1	0	1
Cytokine release syndrome		1	0	1
Depressed level of consciousness		0	1	1
Febrile neutropenia		6	0	6
Fever		2	0	2
gamma-glutamyl transferase increased		1	0	1
Hematoma		4	2	6
Hepatobiliary disorders - sinusoidal obstruction syndrome		1	0	1
Hypokalemia		2	0	2
Hypotension		2	0	2
Immune system disorders - Other specify: engraftment syndrome		1	0	1
Infections and infestations - Other specify: infection CMV		1	0	1
Infections and infestations - Other specify: Septicemiae from Pseudomonas plecoglossicida		1	0	1
Left ventricular systolic dysfunction		0	1	1
Lung infection		2	0	2
Mucositis oral		2	0	2
Rash maculo-papular		1	0	1
Sepsis		2	0	2
Sinus tachycardia		0	1	1
Tumor lysis syndrome		3	0	3
Upper gastrointestinal hemorrhage		1	0	1
Vascular disorders - Other specify: hematoma legs		1	0	1
Vomiting		1	0	1
Weight gain		1	0	1

A total of 28 patients had at least one adverse event (AE) during the study, of which 20 patients had at least one grade 3/4 AE.

Supplementary Table 8. List of chemistry laboratory abnormalities and grade (highest toxicity grade per patient) as compared to local normal ranges.

Grades	0	1	2	3	4	NA	Total
Albumin (low)	5	13	9	0	0	1	28
Alkaline phosphatase (high)	22	6	0	0	0	0	28
ALT (high)	2	16	5	5	0	0	28
Amylase (high)	23	2	2	0	0	1	28
AST (high)	3	16	5	4	0	0	28
Calcium (low)	12	12	3	0	1	0	28
Creatinine	21	7	0	0	0	0	28
GGT (high)	6	8	8	3	0	1	26
Lipase (high)	20	2	4	2	0	0	28
Phosphate (low)	19	3	3	3	0	0	28
Potassium (low)	16	9	0	3	0	0	28
Sodium (low)	15	12	0	1	0	0	28
Total Bilirubin (high)	22	4	1	1	0	0	28
Uric acid (high)	19	7	0	0	0	2	28

The highest toxicity grade per patient was counted only once per patient. This table includes also abnormalities not reported by the local investigator as AEs.

Supplementary Table 9. List of Hematologic laboratory abnormalities and grade (highest toxicity grade per patient) as compared to local normal ranges.

Grade	0	1	2	3	4	Total
Absolute neutrophil count decrease	1	1	0	2	24	28
Hemoglobin decrease	1	3	16	8	0	28
Platelet count decrease	0	3	3	5	17	28
White blood cell count decrease	1	1	1	7	18	28

The highest toxicity grade per patient was counted only once per patient. This table includes also abnormalities not reported by the local investigator as AEs.

Supplementary Table 10. Serious Adverse Events (SAE) observed in the phase II

SAE Term	SAE Category	Grade	Outcome
Intestinal massive hemorrhage	Life threatening	4	Ongoing
Febrile neutropenia	Prolongation of hospitalization	3	Resolved
Sepsis	Prolongation of hospitalization	3	Resolved
Febrile neutropenia	Prolongation of hospitalization	3	Resolved
Febrile neutropenia	Prolongation of hospitalization	3	Resolved
Febrile neutropenia	Prolongation of hospitalization	3	Resolved
Catheter related infection	Prolongation of hospitalization	3	Resolved
Hematuria	Prolongation of hospitalization	2	Resolved
Engraftment syndrome	Life threatening	3	Resolved
Mycosis pneumopathy	Life threatening	4	Death
Sinusoidal obstruction syndrome	Prolongation of hospitalization	3	Resolved
Encephalopathy	Death	5	Death
Maculo-papular rash	Prolongation of hospitalization	2	Resolved
Upper respiratory tract infection	Prolongation of hospitalization	2	Resolved
Sepsis	Prolongation of hospitalization	4	Resolved
Sinusoidal obstruction syndrome	Life threatening	4	Resolved
Pain	Prolongation of hospitalization	3	Resolved
Sinusoidal obstruction syndrome	Life threatening	4	Ongoing
Febrile neutropenia	Prolongation of hospitalization	2	Resolved
Bacteremia	Other medically important condition	2	Ongoing
Sinusoidal obstruction syndrome	Other medically important condition	3	Resolved
Multi organ failure	Death	5	Death
Sinusoidal obstruction syndrome	Other medically important condition	3	Ongoing
Sinusoidal obstruction syndrome	Other medically important condition	2	Resolved
Disease progression	Death	5	Death
Sinusoidal obstruction syndrome	Other medically important condition	3	Resolved

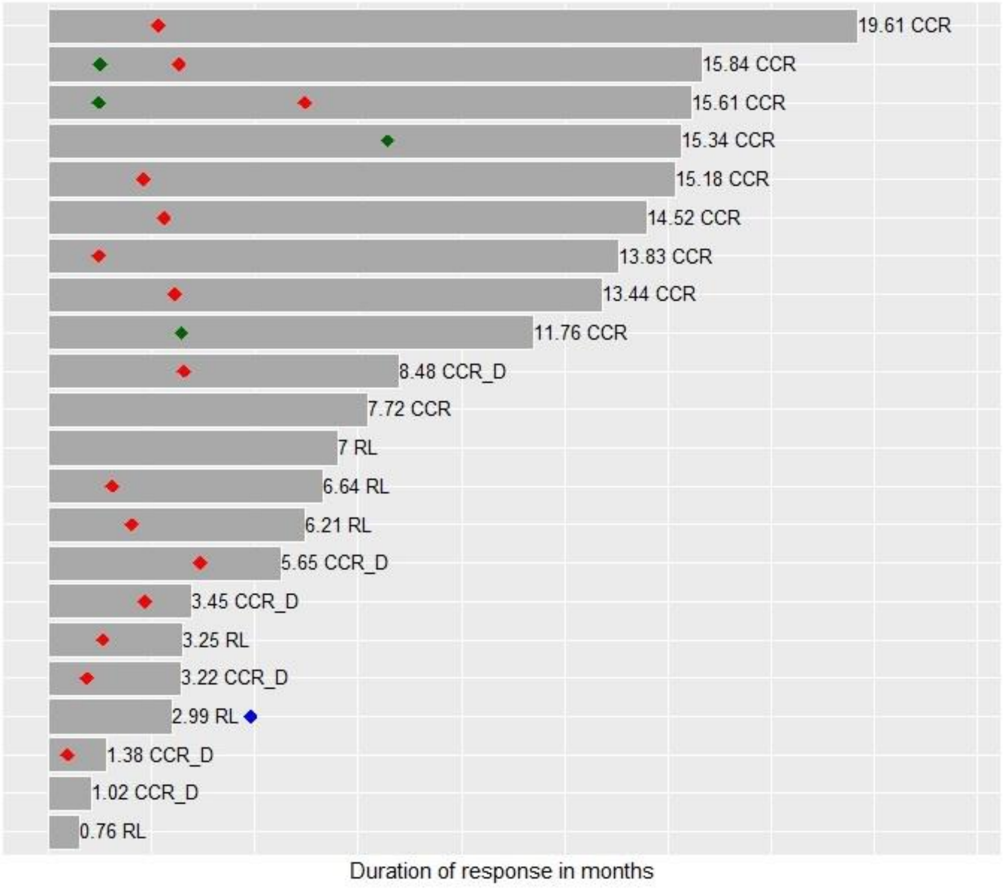

Supplementary Figure 1. Duration of response bar plot (n=22).
CCR: Continuous Complete Remission; RL: Relapse; CCR_Death: Death while in Continuous Complete Remission. Red squares: HSCT treatment; Blue square: in this patient HSCT was performed after additional treatment with blinatumomab due to loss response; Green squares: CAR-T treatment. Numbers at the end of each bar represent the duration of response in months. Four patients were non-responders (not reported here).

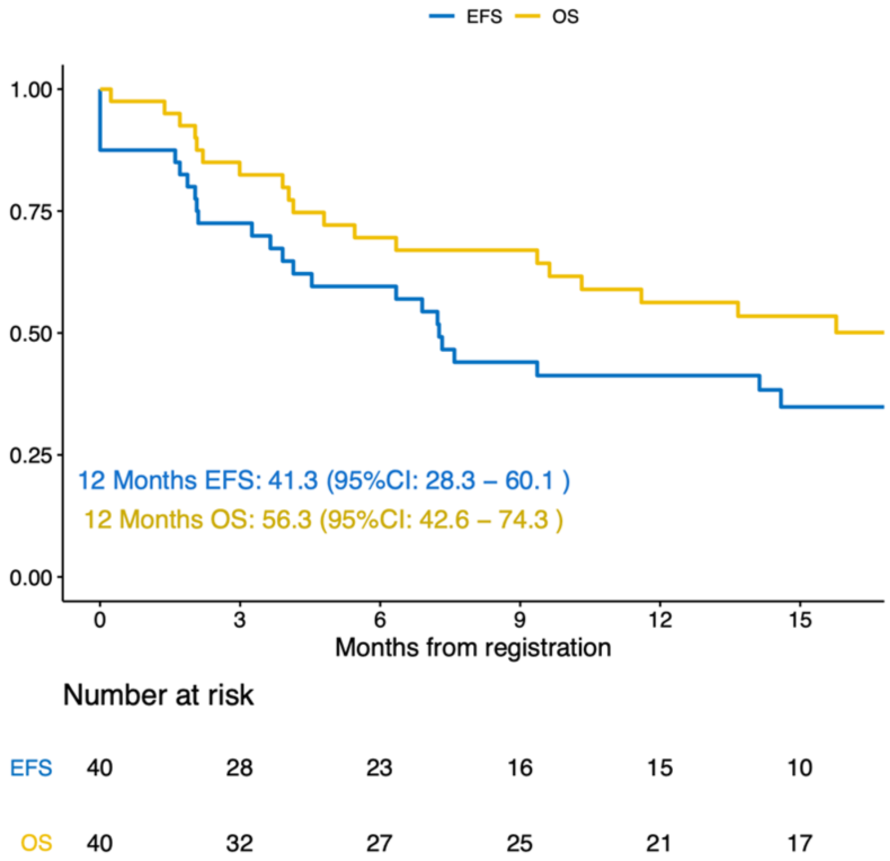

Supplementary Figure 2. EFS and OS for all patients from phase I treated at the RP2D, and phase II patients (n=40). Number at risk is presented below the graph. Blue line: Event Free Survival. Yellow line: Overall Survival. Non responders are added as event on day 0. RP2D: Recommended Phase II dose = 1.8 $mg.m^2$

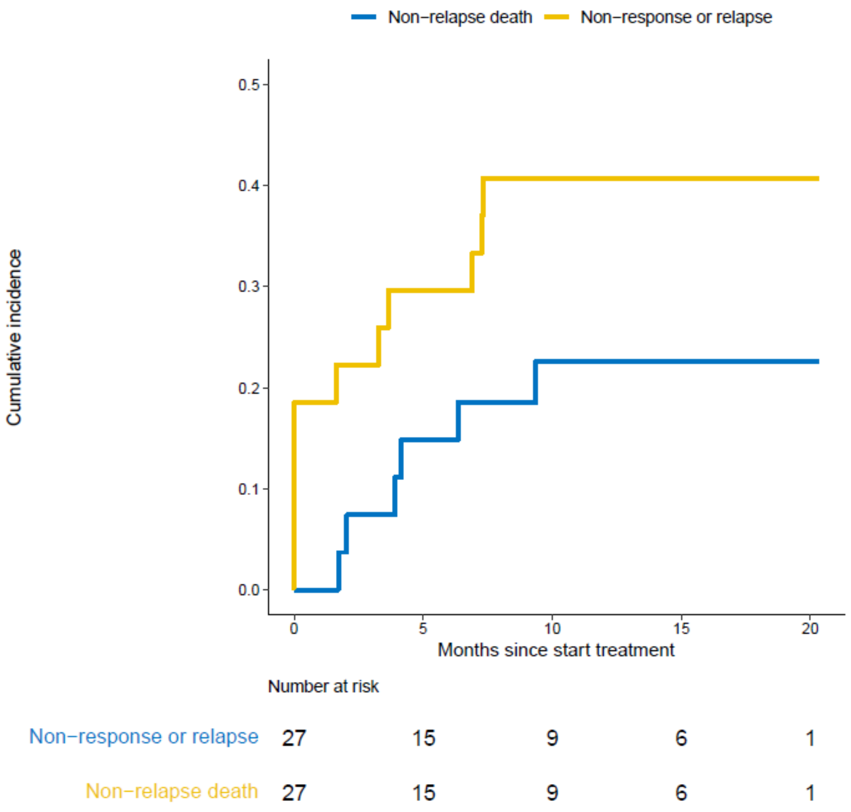

Supplementary Figure 3. Cumulative incidence of refractory/relapse and non-relapse death. The number at risk is presented below the graph. Blue line: refractory/relapse cumulative incidence. Yellow line: non-relapse death cumulative incidence. Non responders are added as event on day 0. Five non-relapse death occurred after hematopoietic stem cell transplant procedure.

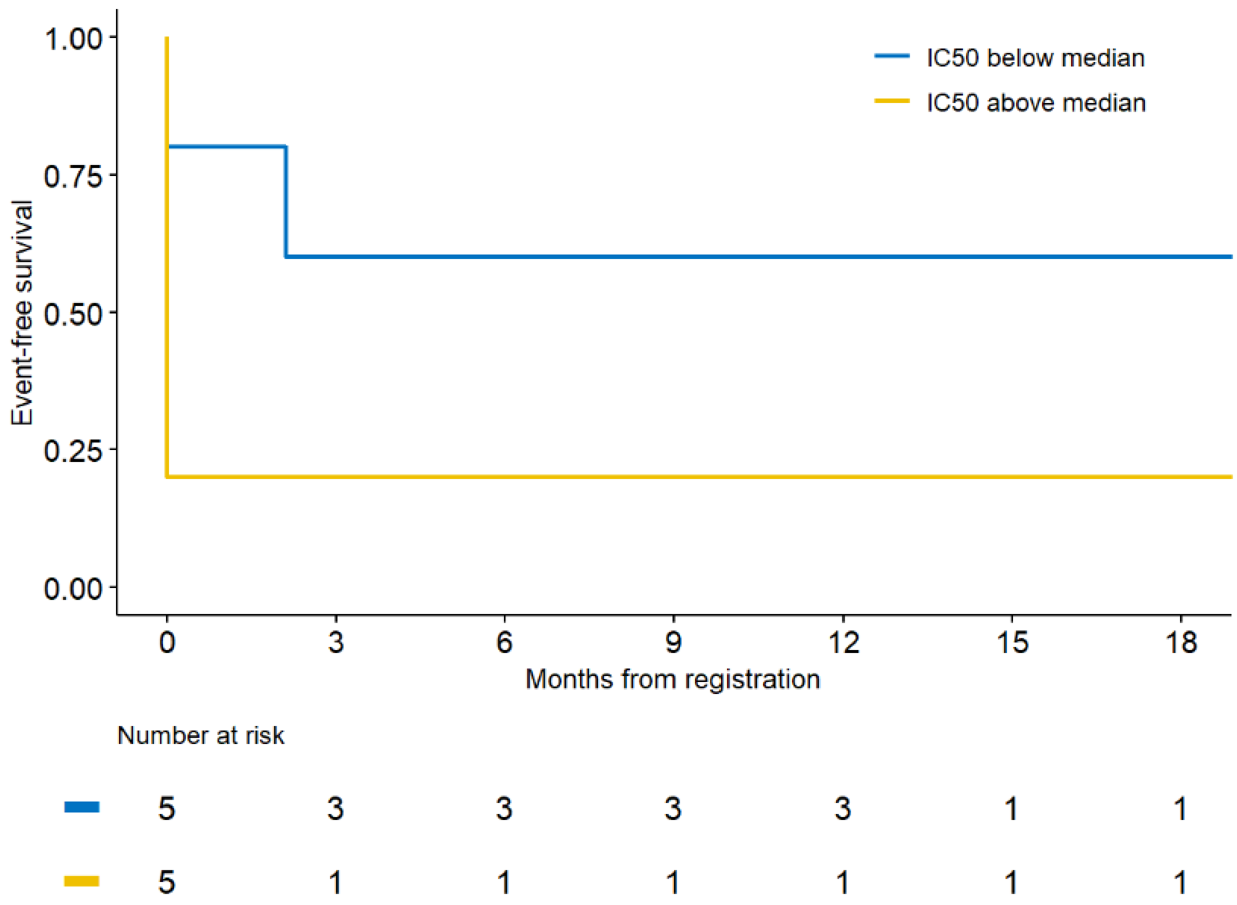

Supplementary Figure 4. EFS in patients with calicheamicin IC_{50} above the median (yellow line) and in those with calicheamicin IC_{50} below the median (blue line), n=10. The event definition is this graph differs in that not achieving MRD neg CR is calculated as an event at day 0. Number at risk is presented below the graph. Patients with *in vitro* calicheamicin sensitivity have a better one year EFS than patients with an IC50 above the median (23% (95%CI 29.3-100%) versus 20% (95%CI 3.46-100%)), although the difference is not statistically significant (p=0.19).

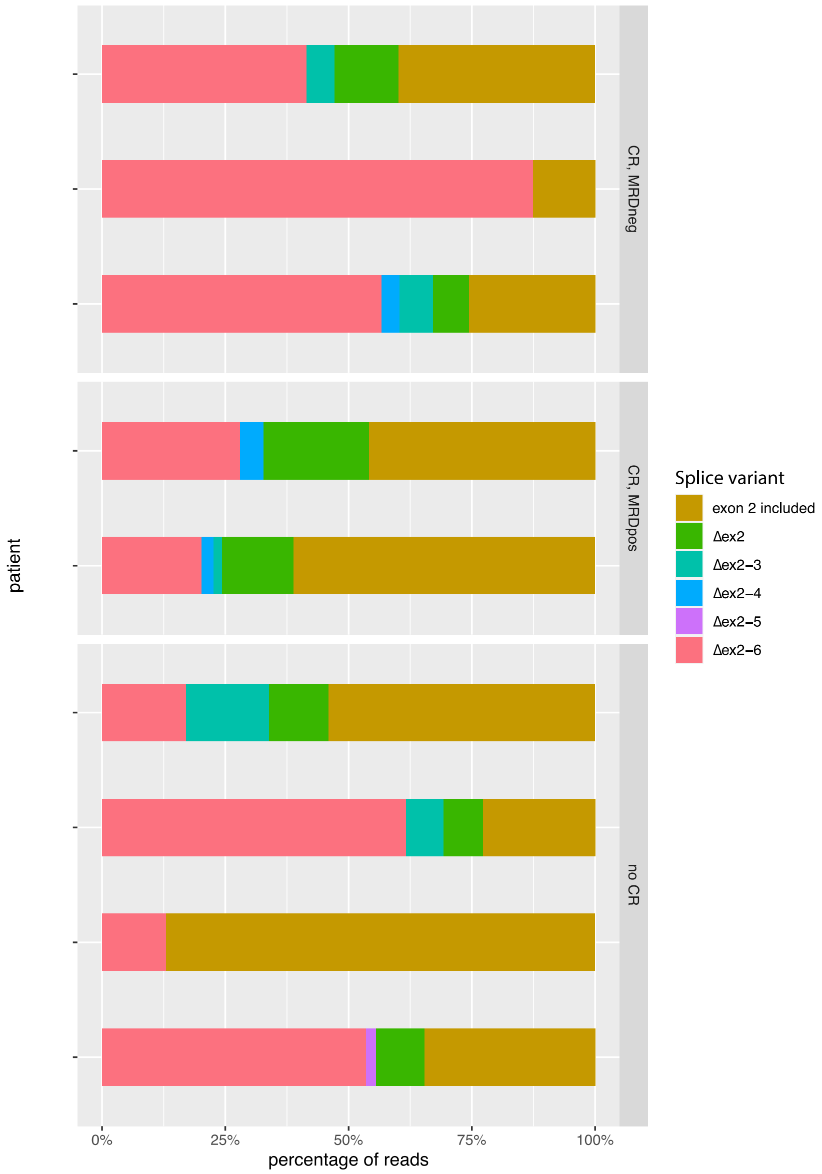

Supplementary Figure 5. Splice variants concerning exon 2 of the CD22 transcript in the 9 patients with RNA sequencing data available.
Percentages represent the number of split reads either in- or excluding the exons as percentage of the total number of split reads. All patients had at least some RNA copies including exon 2. The variant delta ex2-6 seems the most prevalent in this sample.

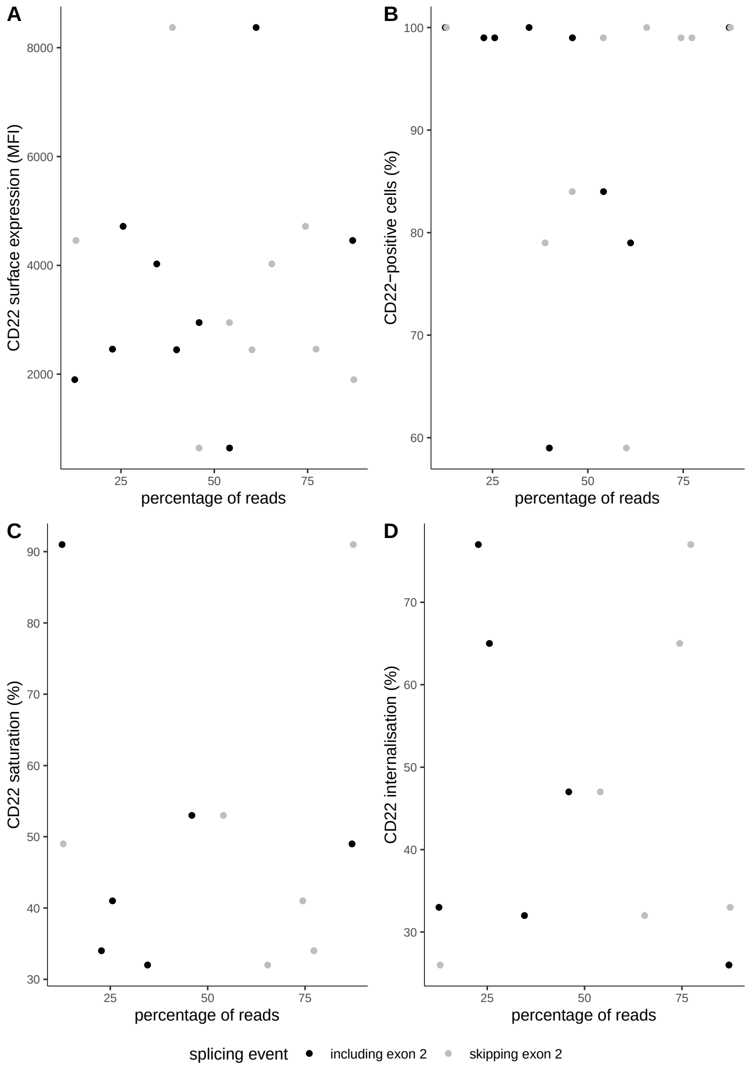

Supplementary Figure 6. Correlation between all splice variants skipping or including exon 2 and CD22 on leukemic blasts as MFI (A), and percentage CD22-positive cells (B), saturation (C) and internalization (D). No trend of association between the in- and exclusion of exon 2 and the expression of CD22 on leukemic blasts, the saturation levels of CD22 on leukemic blasts with InO, or the internalization levels of InO was found.

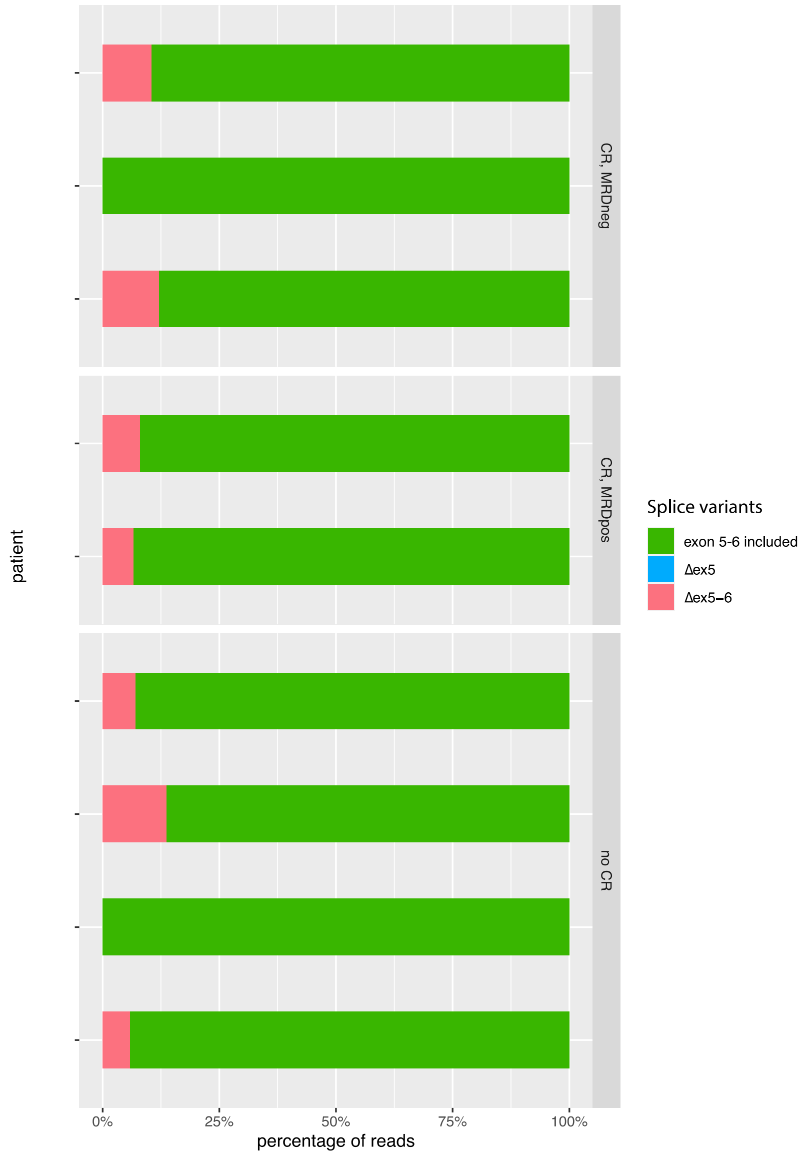

Supplementary Figure 7. Splicing variants concerning exon 5 and 6 of the CD22 transcript in the 9 patients with RNA sequencing data available. Percentages represent the number of split reads either in- or excluding the exons as percentage of the total number of split reads. All patients had at least some RNA copies including exon 5 and 6.

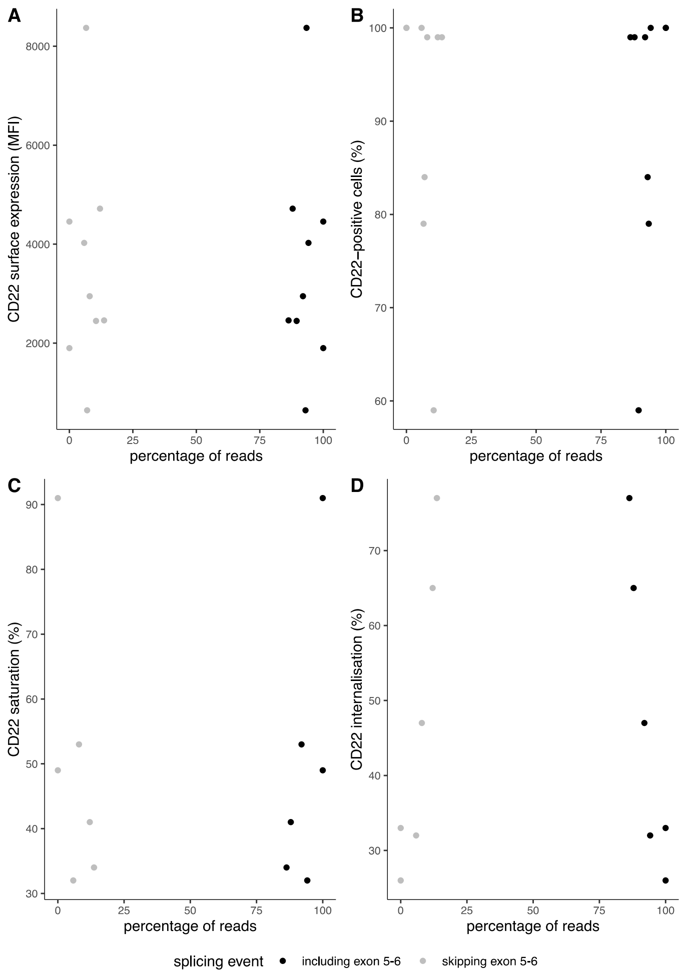

Supplementary Figure 8. Correlation between splicing variants skipping or including exon 5-6 and CD22 on leukemic blasts as MFI (A), and percentage CD22-positive cells (B), saturation (C) and internalization (D). No trend of association between the in- and exclusion of exon 5-6 and the expression of CD22 on leukemic blasts, the saturation levels of CD22 on leukemic blasts with InO, or the internalization levels of InO was found.

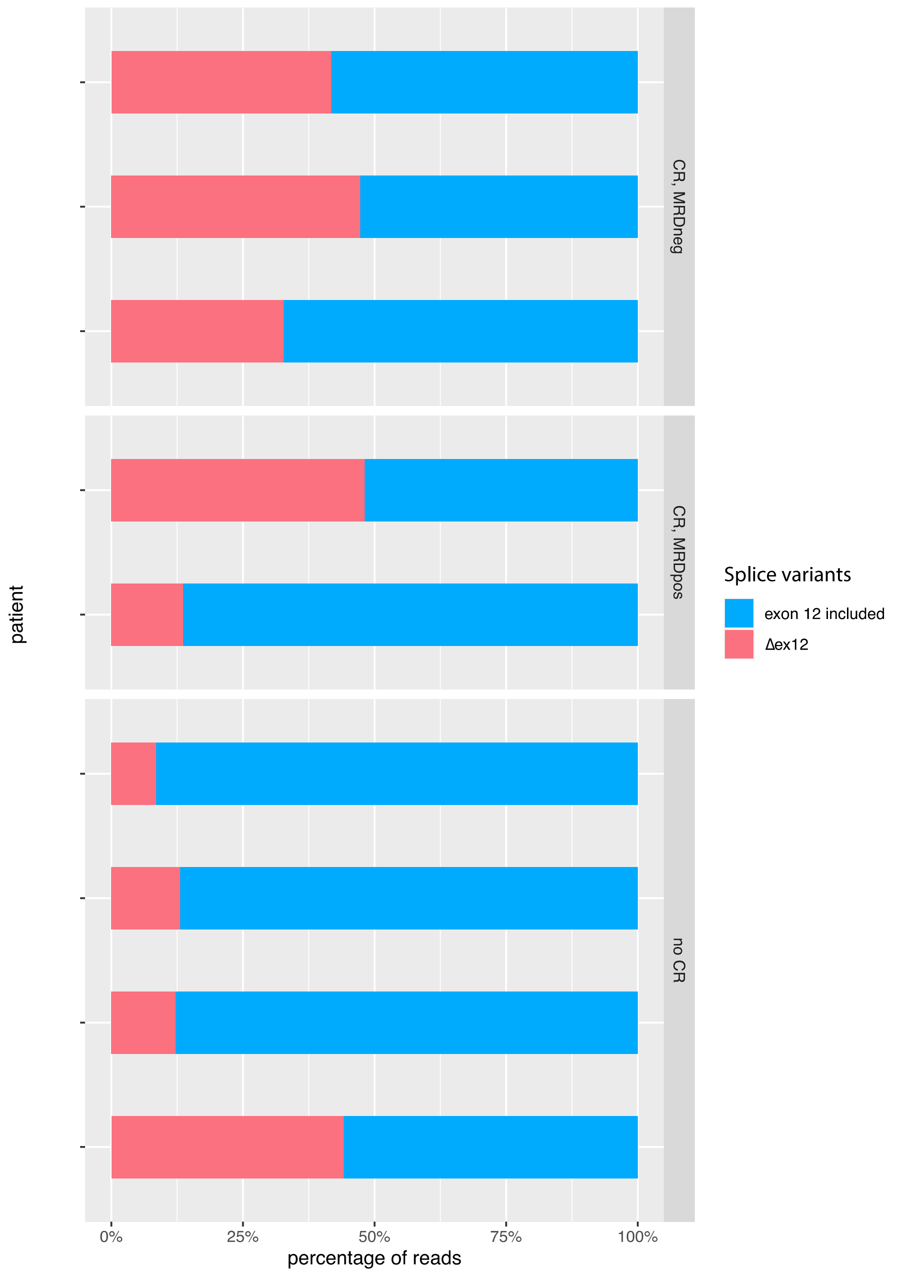

Supplementary Figure 9. Splicing variants concerning exon 12 of the CD22 transcript in the 9 patients with RNA sequencing data available. Percentages represent the number of split reads either in- or excluding the exons as percentage of the total number of split reads. All patients had at least some RNA copies including exon 12.

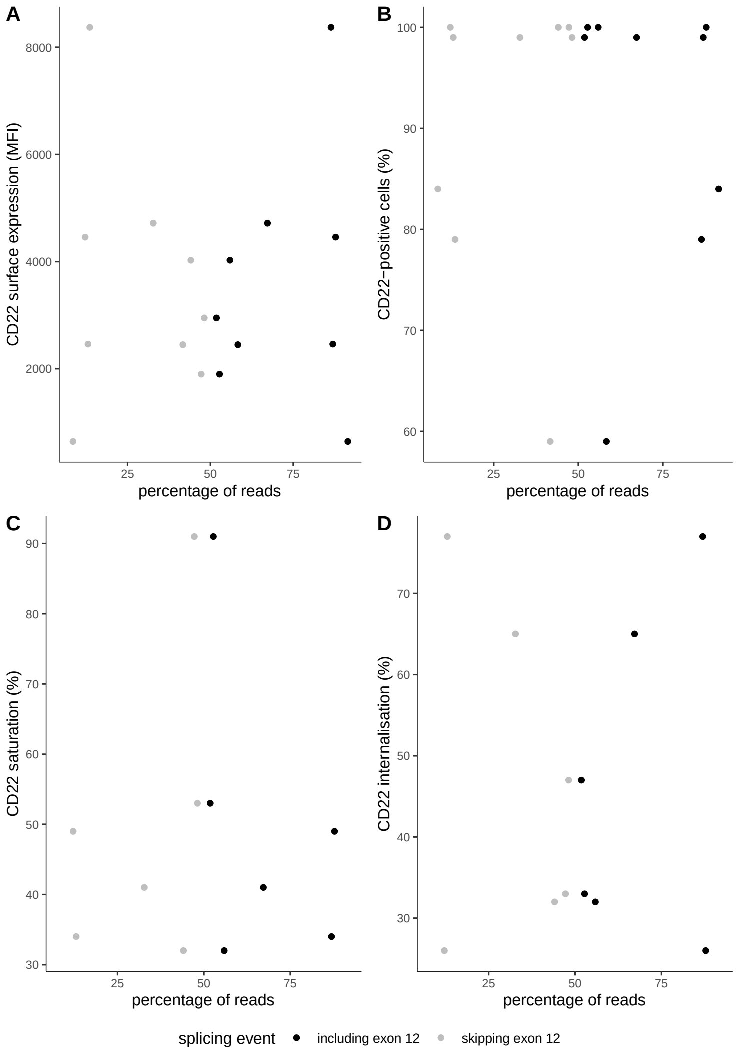

Supplementary Figure 10. Correlation between the splice variant skipping or including exon 12 and CD22 on leukemic blasts as MFI (A), and percentage CD22-positive cells (B), saturation (C) and internalization (D). No trend of association between the in- and exclusion of exon 12 and the expression of CD22 on leukemic blasts, the saturation levels of CD22 on leukemic blasts with InO, or the internalization levels of InO was found.

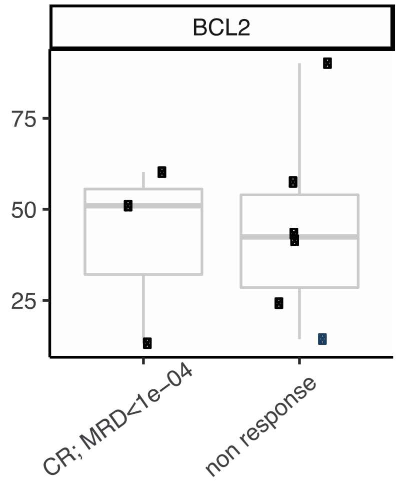

Figure 10. *BCL2* gene expression per response group. Presented as fragments per kilobase per million (FPKM), which correct for library size and gene length. No difference in *BCL2* gene expression between response groups was observed.

REFERENCES (for supplementary files)

1. Theunissen P, Mejstrikova E, Sedek L, et al. Standardized flow cytometry for highly sensitive MRD measurements in B-cell acute lymphoblastic leukemia. *Blood*. 2017;129(3):347-357. doi:10.1182/blood-2016-07-726307

2. van der Velden VH, Panzer-Grümayer ER, Cazzaniga G, et al. Optimization of PCR-based minimal residual disease diagnostics for childhood acute lymphoblastic leukemia in a multi-center setting. *Leukemia*. 2007;21(4):706-713. doi:10.1038/sj.leu.2404535

3. Kalina T, Flores-Montero J, van der Velden VH, Martin-Ayuso M, Bottcher S, Ritgen M, et al. EuroFlow standardization of flow cytometer instrument settings and immunophenotyping protocols. Leukemia 2012; 26:1986-2010.

4. Theunissen P, Mejstrikova E, Sedek L, et al. Standardized flow cytometry for highly sensitive MRD measurements in B-cell acute lymphoblastic leukemia. Blood. 2017;129(3):347-357.

5. van der Velden VH, van Dongen JJ. MRD detection in acute lymphoblastic leukemia patients using Ig/TCR gene rearrangements as targets for real-time quantitative PCR. Methods Mol Biol. 2009;538:115-150.

6. van der Velden VH, Panzer-Grümayer ER, Cazzaniga G, et al. Optimization of PCR-based minimal residual disease diagnostics for childhood acute lymphoblastic leukemia in a multi-center setting. Leukemia. 2007;21(4):706-713.
