# Supplementary material for: Inotuzumab ozogamicin as single agent in pediatric patients with relapsed and refractory acute lymphoblastic leukemia: results from a phase II trial
Source: Leukemia. 2022 Apr 25;36(6):1516–24. doi: 10.1038/s41375-022-01576-3 (PMC9162924; doi:10.1038/s41375-022-01576-3)

**Protocol: ITCC-059**

# A phase I/II study of Inotuzumab Ozogamicin as a single agent and in combination with chemotherapy for pediatric CD22-positive relapsed/refractory Acute Lymphoblastic Leukemia - *Study ITCC-059*

| | |
|---|---|
| Protocol version | v4.1 |
| Sponsor | Erasmus MC |
| EUDRA-CT nr | 2016-000227-71 |
| Dutch Trial Registry nr. | NTR5736 |
| Date: | 22-MAR-2021 |

**SPONSOR**

Erasmus MC – Sophia Children's Hospital
Department of Pediatrics
Wytemaweg 80, 3015 GN Rotterdam
The Netherlands

**DCOG-ECTC Trial Office**

Princess Máxima Center for Pediatric Oncology
PO Box 85090, 3508 AB Utrecht
Phone: +31-6-5000 6679 / +31-88-972 5711
Fax: +31-88-972 9009
DCOG-ECTC Web Site: https:\\www.dcog-ectc.nl

**TRIAL STEERING COMMITTEE**

**International Coordinating Investigator**

**C. Michel Zwaan, MD, PhD**
**DCOG-ECTC Chair**
Dept of Pediatric Oncology/Hematology
Erasmus MC – Sophia Children's Hospital,
PO Box 2060, 3000 CB Rotterdam, Netherlands
& Princess Máxima Center for Pediatric Oncology, Utrecht
Phone: +31-10-7036691 (Erasmus MC) or +31-88-972 9011 (Máxima)
Email: c.m.zwaan@erasmusmc.nl or c.m.zwaan@prinsesmaximacentrum.nl

**Other members of the Trial Steering Committee**

**James Whitlock, MD**
The Hospital for Sick Children
555 University Avenue
Toronto, ON M5G 1XG Canada
Phone: 416-813-8885
E-mail: jim.whitlock@SickKids.ca

**David Ziegler, MBBS, MD**
Sydney Children's Hospital
High St, Randwick, NSW 2031 Australia
Phone: +61-2-9382-1730
Email: d.ziegler@unsw.edu.au

**Inge M. van der Sluis, MD, PhD**
Princess Máxima Center for Pediatric Oncology, Utrecht
Phone: +31-88-9729519
Email: i.m.vandersluis@prinsesmaximacentrum.nl

**Barbara Sleight, MD**
Medical Director
Pfizer Oncology
Mailstop MS 8260-2135
Groton, Connecticut 06349
Mobile Phone: +1-203-687-9781
Email: Barbara.sleight@pfizer.com

**Other members of the Trial Steering Committee for Stratum 3 (VHR BCP-ALL cohort)**

**Arend von Stackelberg, MD**
IntReALL Study group leadership
Charité - Universitätsmedizin
Charitéplatz 1, 10117 Berlin, Germany
Phone: + 49-30-450 666 833
Email: Arend.Stackelberg@charite.de

**Franco Locatelli, MD**
IntReALL Study group leadership
Department of Pediatric Hematology-Oncology
Opsedale Pediatrico Bambino Gesu
Piazza S. Onofrio, 4 – 00165, Roma, Italy
Phone: 06.68592129
Email: franco.locatelli@opbg.net

**Peter Hoogerbrugge, MD**
IntReALL Study group leadership
Princess Máxima Center for Pediatric Oncology, Utrecht
Phone: +31 88-9727272
Email: p.m.hoogerbrugge@prinsesmaximacentrum.nl

**Pierre Rohrlich, MD**
IntReALL Study group leadership
Universitair medisch centrum,
30 Voie Romaine, 06000 Nice, Frankrijk
Phone: +33 04 92 03 58 44
Email: rohrlich.ps@chu-nice.fr

**Study Statistician**

**Adriana Thano**
Princess Máxima Center for Pediatric Oncology
Postbus 113 .3720 AC Bilthoven
Heidelberglaan 25, 3584 CS Utrecht
Phone: +31 88 97 25 206
Email: A.Thano@prinsesmaximacentrum.nl

**Pharmacokinetics**

**Alwin Huitema, PhD**
Hospital Pharmacist/Clinical Pharmacologist
Netherlands Cancer Institute and Princess Máxima Center for Pediatric Oncology
Postbus 113 .3720 AC Bilthoven
Heidelberglaan 25, 3584 CS Utrecht
The Netherlands
E-Mail: a.huitema@nki.nl; A.D.R.Huitema-6@prinsesmaximacentrum.nl

**Ying Chen, Ph.D**
Director, Clinical Pharmacology
Pfizer Oncology
+1 (858) 622-7910
Ying.Chen@pfizer.com

**Laboratories**

**1. Central laboratory coordination of PD studies and calicheamicin sensitivity assay**
Monique den Boer, Prof. PhD.
Central laboratory for Early Clinical Trials
Princess Máxima Center for Pediatric Oncology
Heidelberglaan 25
3584 CS Utrecht, the Netherlands
Phone: +31-88-972 5071
Email: m.l.denboer@prinsesmaximacentrum.nl

**2. Central laboratory for CD22 expression and saturation analysis and MRD studies**
Vincent van der Velden, PhD
Laboratory for Medical Immunology
Wytemaweg 80
3015 CN Rotterdam
Phone: +31-10-7044253
Email: v.h.j.vandervelden@erasmusmc.nl

**3. Central Review of cytogenetic data**
Berna Beverloo, PhD
Department of Clinical Genetics, ErasmusMC
Wytemaweg 80
3015 CN Rotterdam, the Netherlands
Phone: +31-10-7043196
Email: b.beverloo@erasmusmc.nl

**4. Central laboratory coordinating Pharmacokinetics and Immunogenicity studies**
QPS Netherlands B.V.
Phone: +31-50-3681048
Email: sampleteam@qps.com

Sample Delivery address: QPS Netherlands B.V.
Professor Rankestraat 42
9713 GG Groningen
The Netherlands
**Samples will be provided from QPS Netherlands to the following laboratories:**

**5. Central laboratory for Immunogenicity Assay**
QPS Pharma
One Innovation Way, Suite 200
Delaware Technology Park
Newark, DE 19711, USA
Attn: Xun Wang

**6. Central laboratory for Pharmacokinetics of InO and unconjugated calichaemicin**
PPD
2244 Dabney Road
Richmond, VA 23230, USA

**DCOG-ECTC Trial Office**

Princess Máxima Center for Pediatric Oncology,
PO Box 85090,
3508 AB Utrecht
Phone: +31-6-5000 6679 / +31-88-972 5711
Fax: +31-88-972 9009
Email: dc-ectc@prinsesmaximacentrum.nl; trialmanagement@prinsesmaximacentrum.nl

**Disclaimer**

Erasmus MC and ITCC protocols and their content are for research purposes only and should not be copied, redistributed or used for any other purpose. The procedures in this protocol are intended only for use at ITCC member institutions under the direction of Erasmus MC / ITCC physicians.

## SIGNATURE PAGE

## A phase I/II study of Inotuzumab Ozogamicin as a single agent and in combination with chemotherapy for pediatric CD22-positive relapsed/refractory Acute Lymphoblastic Leukemia - *Study ITCC-059*

**This protocol has been approved by:**

| | | | |
|---|---|---|---|
| Name: | C.M. Zwaan, MD PhD, Professor of Pediatric Oncology Erasmus MC | **Trial role**: | Coordinating principal Investigator (PI) |
| Signature: | ____________________________ | Date: DD/MON/YYYY | ____________________ |
| Name: | E.H.H.M Rings, Professor of Pediatrics and Head of the Dept. of Pediatrics, Erasmus MC | **Trial role**: | On behalf of Erasmus MC as sponsor |
| Signature: | ____________________________ | Date: DD/MON/YYYY | _____________________ |

**InO trial - *Study ITCC-059***

**INVESTIGATOR DECLARATION AND SIGNATURE**

- I have read the current protocol version, and agree to abide by all provisions set forth therein.
- I agree to conduct this clinical study in accordance with the design and specific provisions of this protocol.
- I agree to personally conduct or supervise this investigation and to ensure that all associates, colleagues, and employees assisting in the conduct of this study are informed about their obligations in meeting these commitments.
- I agree to maintain adequate and accurate records and to make those records available for audit and inspection in accordance with relevant regulatory requirements.
- I will ensure that the investigational drug supplied by the sponsor will be used only for administration to subjects included in this clinical research study.

| | | | |
|---|---|---|---|
| Name: | ____________________________ | Hospital: | __________________________ |
| Signature: | ____________________________ | Date: DD/MON/YYYY | ___________________________ |

**AMENDMENT HISTORY**

The following amendments and/or administrative changes have been made to this protocol since the implementation of the first approved version.

| Amendment number | Date of amendment | Protocol version number | Type of amendment | Summary of amendment |
|---|---|---|---|---|
| Version 2.0 | 31-AUG- 2018 | 2 | Substantial amendment | • Phase I study design was amended to better define the recommended phase 2 dose (see section amendment phase 1 study, starting at page 7)<br>• Literature section was updated with recently published data (Section 2)<br>• InO was approved by EMA and FDA for BCP-ALL in adults (Section 2)<br>• Safety information was updated based on the most recent IB (May 2018) and the SPC (Section 2)<br>• Inclusion/exclusion criteria were modified to allow patients to be enrolled 90 days after HSCT, and to exclude patients who have already been treated with anti-tumor vaccines (Section 3)<br>• The number of cycles of InO were limited for patients who are MRD-negative and will subsequently be transplanted, in line with the SPC (Section 4)<br>• Dose delay guidelines were added for transaminase or total bilirubin elevation during the 1st cycle and at the start of subsequent cycles (Section 4)<br>• The DLT definition was modified: grade 4 injection site reactions are no longer excluded from the DLT definition, whereas transaminases related to leukemic liver infiltration was excluded from the DLT definition (Section 4.6)<br>• Added guidance for infusion of low volume doses to very small children (Section 6.1.5.)<br>• Volume of blood samples collected in younger children was modified to comply with EMA blood volume regulations for children (Section 7)<br>• PK sampling time-points were reduced for younger children (Section 7.8)<br>• Time-lines of the study were adapted to the changes of the design (Section 9)<br>• In the decision on the recommended phase 2 dose, cumulative toxicity will be taken into account (Section 9)<br>• The design for the phase 2 study was modified to a single-stage design rather than |

| | | | | |
|---|---|---|---|---|
| | | | | a 2-stage design, and the sample size was reduced (Section 9)<br>• Contraception measures were added to implement *highly* effective contraception and for a longer period after treatment following CTFG recommendations (Section 10.2)<br>• SUSAR definitions were added (Section 12.9) |
| Version 3.0 | 31-AUG-2019 | 3 | Substantial amendment | • Stratum 1B (InO combined with chemotherapy) was added (Section 1)<br>• Literature section was updated with recently published data (Section 2)<br>• The rationale for this study was updated for the combination treatment (Section 2.7)<br>• Additional exclusion criteria for Stratum 1B (Section 3.4) were added<br>• Treatment program for Stratum 1B was added (Section 4.2)<br>• Information about non-IMPDs used for Stratum 1B were added (Section 6)<br>• Revised DLT criteria were added for stratum 1B (Section 4.6)<br>• PK sampling time-points were adapted according to preliminary pediatric patients PK analysis (Section 7.8)<br>• The platelet count criteria to proceed to subsequent cycles were updated for Phase 2 and Stratum 1B BCP-ALL patients<br>• Hematologic DLT definition was updated<br>• General spelling, grammar, repetitions and formatting errors corrected. |
| Version 4.0 | 02-FEB-2021 | 4 | Substantial amendment | • The dose of Dexamethasone administered to patients enrolled in Stratum 1B was modified (Section 4.3.2), based on toxicity findings in the first few patients treated in Stratum 1B<br>• Rationale and details for re-escalation to dose level -1 (and subsequent levels) InO with reduced dose Dexamethasone for Stratum 1B/1B-ASP.<br>• *Table 14* was updated to include the dose modification/reduction of intrathecal Methotrexate in case of toxicity in Stratum 1B/1B-ASP (Section 4.4)<br>• Criteria for dose modification/interruption in stratum 1B/1B-ASP in case of ALT/AST increment have been modified consistently with Section ***4.3.5.2***<br>• Criteria to start cycle 2 (and subsequent cycles) in stratum 1B/1B-ASP in case of total bilirubin and/or ALT/AST elevation have been modified<br>• The background and rationale for this study were updated to include first relapse ALL patients with very high risk (VHR) disease |

| | | | | |
|---|---|---|---|---|
| | | | | characteristics and to present recently published data regarding Stratum 3 (Section 2)<br>• A new cohort was added (Stratum 3) including 1st relapse ALL VHR patients, to test InO using a Simon 2-stage design (phase 2 study using single-agent InO)<br>• Inclusion/Exclusion criteria for the “Very High-Risk” cohort were added (Section 3.4)<br>• Schedule of event was updated to account for the VHR cohort (see **Table 25** and<br>• ***Table 34***)<br>• ADAs (anti-drug antibody) sampling will be stopped from amendment 4 onwards.<br>• Assessing the impact of InO reinduction on the delivery of CAR-T cells was added to the objectives of the study for Stratum 3 only.<br>• IntReALL Study group leadership was added to the Steering Committee for the VHR cohort<br>• General spelling, grammar, repetitions and formatting errors were corrected |
| Version 4.1 | 22-MAR-2021 | 4 | NOT substantial | NA – not substantial changes |

**AMENDMENT VERSION 4**

**Rationale Substantial Amendment (Version 4.0 and including not-substantial version 4.1)**

The amendment has two main purposes:

- The first is to reduce the dose of dexamethasone administered to patients enrolled in stratum 1B and 1B-ASP, from 20 $mg/m^2$/day to 10 $mg/m^2$/day, and subsequently re-escalate InO to DL-1 if considered safe.
- The second is to add the details for an additional cohort (Stratum 3) including patients with "very high risk" (VHR) first relapse of B-cell precursor ALL, according to the criteria proposed by the IntReALL consortium.

The rationale for these modifications is explained in the following paragraphs with the same order.

**Ad1: Modification of dexamethasone dose**
Preliminary data from the patients enrolled in Stratum 1B and treated with InO at 1.1 $mg/m^2$ (dose level -1, DL-1) in combination with a modified version of UKALL-R3 re-induction chemotherapy during Cycle 1, highlighted potential hepatic toxicity putatively attributable to the cumulative effect of InO and chemotherapy (vincristine, dexamethasone and intrathecal methotrexate).

Patients were treated during Cycle 1 at DL-1 with InO at 1.1 $mg/m^2$ (0.5 $mg/m^2$ on day 1, and 0.3 $mg/m^2$ on days 8 and 15) combined with the UKALL-R3 modified regimen (no asparaginase). The latter consisted of intravenous (IV) Vincristine 1.5 $mg/m^2$ on days 3, 10, 17 and 24, oral dexamethasone 20 $mg/m^2$/day on days 1-5 and 15-19 divided into 2 daily doses, and Methotrexate i.t. on day 1 and on day 8.

One patient was diagnosed with VOD after receiving two doses of InO, and one patient suffered Grade 3 hepatic transaminase elevation lasting more than 7 days (both fulfilling the criteria for DLTs). Another patient experienced an increase in hepatic transaminases without fulfilling the criteria of DLT as specified in section 4.6. Finally, the fourth patient had a neutropenic fever diagnosed at day 13 of Cycle 1 during cycle 1, without hepatic toxicity. Consequently, the Steering Committee agreed upon de-escalating the dose of InO to 0.8 $mg/m^2$, as per protocol.

**Patient narratives: Dose level -1 (1.1 $mg/m^2$) Stratum 1B, Cohort 1, Cycle I.**

- **Patient 63-07**.
  The first DLT (VOD) occurred in a 7 year old male patient who was enrolled after failing three prior lines of ALL therapy, including allo HSCT and CAR-T cells therapy. Grade 3 VOD occurred after the second dose of InO during cycle 1.

  The patient achieved CR with negative MRD after the first induction with chemotherapy (SEHOP - PETHEMA 2013) and suffered the 1st BM relapse in 2018, then underwent a haploidentical HSCT on 21st January 2019. After four months, the patient suffered a second relapse (May 2019) and was treated with CAR-T cells therapy. Nine months later, he experienced a third isolated CD22+ bone marrow relapse and enterd the ITCC-059 study.

  Two days before receiving the third dose of InO, the patient lamented pain in the right upper quadrant associated with hepatomegaly that increased from 1 cm (screening visit) to 3 cm. Compared to the baseline, it was also reported a weight increment higher than 5%, but no ascites. A moderate transaminase increment was observed (ALT 91 UI/L and AST 73 UI/L; normal ranges < 31 U/L and < 50 U/L, respectively). Total bilirubin levels remained normal at 7.3 UMOL/L (normal range 3.4 – 17 UMOL/L). In the following seven days, total bilirubin levels increased at 20 Umol/L (Grade 1). On 29th May, abdominal ultrasound/Doppler revealed hepatomegaly with gallbladder edema and hepatopetal flow of 36cm/second.

On the same day (15th cycle 1), the patient was diagnosed with VOD based on the modified Seattle criteria (painful hepatomegaly, increased body weight >5%). Study treatment was immediately discontinued and defibrotide was initiated (800 mg IV daily for 22 days). This grade 3 AE fulfilled the criteria of DLT. The event completely resolved after treatment.

- **Patient 66-31.**
  The patient, a 17 year old male, had a history of diabetes mellitus type 1 diagnosed at age of 10 years, and was diagnosed with BCP ALL, CNS1, in 2017. He completed first line NOPHO ALL2008 IR chemotherapy on 23 January 2020. In May 2020, he suffered a first relapse which was refractory to IntReALL re-induction, therefore, he was eligible for this study.

  At study baseline, this patient had Grade 1 AST/ALT increased of unknown etiology (Table 1)

  **DLT #2:** Grade 3 AST/ALT increased lasting more than 7 days.
  After receiving the first dose of InO, the patient experienced Grade 3 ALT elevation that persisted until the end of study treatment, while AST increased to Grade 3 starting at day 10. No total bilirubin elevation was noted during study therapy. The patient immediately went off protocol.

  Notwithstanding, the patient was in good clinical conditions in spite of these laboratory abnormalities and the hepatic transaminases were in the lower third of the Grade 3 toxicity range (CTCAE v4.03).

  After the patient went off protocol, the treating physician considered the patient was benefitting from InO, with improvements of bone pain related to leukemic infiltration, therefore further treatment with InO was performed off protocol.

***Table 1. Hepatic Transaminases Trend in Patient 66-31. Alanine aminotransferase (AST) and aspartate aminotransferase (AST)***

| | BL | D1 InO + Dex | D2 Dex | D3 Dex | D4 Dex | D5 Dex | D6 | D7 | D8 | D9 | D10 | D11 | D12 |
|---|---|---|---|---|---|---|---|---|---|---|---|---|---|
| Date | 01.07.20 | 02.07.20 | 03.07.20 | 04.07.20 | 05.07.20 | 06.07.20 | 07.07.20 | 08.07.20 | 09.07.20 | 10.07.20 | 11.07.20 | 12.07.20 | 13.07.20 |
| AST* | 1.35 Gr. 1 | 1.16 Gr. 1 | 1.52 Gr. 1 | 0.82 Gr. 1 | 1.24 Gr. 1 | 1.15 Gr. 1 | 2.33 Gr. 1 | 2.10 Gr. 1 | 0.64 Normal | 0.55 Normal | 4.92 Gr. 3 | 4.51 Gr. 3 | 5.66 Gr. 3 |
| ALT** | 0.74 Gr. 1 | 0.59 Gr. 1 | 0.60 Gr. 1 | 0.73 Gr. 1 | 01.47 Gr. 1 | 2.87 Gr. 3 | 4.05 Gr. 3 | 5.26 Gr. 3 | 3.64 Gr. 3 | 2.67 Gr. 3 | 7.72 Gr. 3 | 9.39 Gr. 3 | 10.6 Gr. 4 |

* Upper limit of normal (ULN) = 0.8 mg/dl
** ULN = 0.52 mg/dl
Toxicity Grade (Gr.) is reported based on CTCAE v4.03
BL=baseline

- **Patient 65-07.**
  This patient was a 17 year old male with three prior lines of ALL therapy including a prior allo HSCT. He displayed an increase in hepatic transaminases without fulfilling the criteria of DLT. No total bilirubin elevation was noted. Despite overall conditions were good and no signs of VOD were present, the day 15 dose of InO was delayed due to transaminases >2.5 x ULN. On day 15, the

patient only received IT methotrexate; the second pulse of dexamethasone was not administered. The patient received the third dose of InO on day 17 at a reduced dose of 0.2 mg/m$^2$ i.o. 0.3 mg/m$^2$. After completing the first cycle, achieving, the patient withdrew from this study and was referred for CAR-T cells therapy as consolidation.

High dosage of dexamethasone at 20 mg/m$^2$/day administered in 5-days blocks at day 1-5 and day 15-19, in combination with InO, might have had the highest impact on the observed liver toxicity. In general, in most ALL induction protocols, low dose steroids are given for a 4-week block; however at relapse, the higher dosages in 5-day blocks are administered to avoid resistance and to control for undesirable side-effects with long-term administration of steroids, mainly infections.

In summary, out of four patients enrolled, two experienced a DLT at DL-1 and, for this reason, the next patients enrolled in Stratum 1B started the treatment with a de-escalated dose of InO at DL-2 (total dose of InO at 0.8 mg/m$^2$ during Cycle 1), as required by the protocol.

Nevertheless, in consideration of the high response rate (RR) observed in patients treated with single agent InO at 1.8 mg/m$^2$ in Cycle 1, and at 1.5 mg/m$^2$ in Cycle 2 to 6 (Stratum 1A and Phase II cohorts), the Steering Committee was concerned that a low dose of InO (0.8 mg/m$^2$ during Cycle 1) might not achieve the same CR rates, even when combined with chemotherapy.[1] Hence, we argue that the dose of InO should be increased given its outstanding activity as single agent observed in this trial; while the dose of Dexamethasone can be reduced, as patients have previously been treated with significant dexamethasone and/or other corticosteroids, and 20 mg/m2 is a very high dose compared to regular ALL protocols mainly using 6 mg/m2. We therefore have reduced the dexamethasone dose to 10 mg/m$^2$/day, to begin immediately once this amendment is approved by each local regulatory agency and IRB/EC, and InO will be re-escalated to DL-1.

In our phase I study (Stratum 1A), in total, during Cycle 1, 16% of patients reported AST grade 3-4 elevation (four Grade 3, no Grade 4), and 16% patients reported grade 3-4 ALT elevation (two Grade 3, and two Grade 4). Blood bilirubin level increased in three patients, (two Grade 3 and one Grade 4). In total six VOD cases were reported in Stratum 1A and Phase 2 (N=25+27), but only one event occurred during InO treatment. The other cases occurred after consecutive alloHSCT or after additional chemotherapy post-InO treatment (analysis updated at the time of amendment writing). Combining data from Stratum 1A at RP2D and from Phase 2, 33 out of 40 (82, 5%) reached complete remission, with 94% of MRD-negativity rate in responding patients; confirming the noteworthy activity of InO single agent at 1.8 mg/m$^2$ in patients with chemotherapy resistant ALL.

A Phase 2 trial in adults tested the combination of InO with mini-hyper-CVD, Cyclophosphamide (150 mg/m2 every 12 h on days 1-3) and dexamethasone (20 mg on days 1-4 and days 11-14), vincristine (2 mg flat dose on days 1 and 8), no anthracycline, Methotrexate (250 mg/m2 at day 1), Cytarabine at 0.5 g/m$^2$ x 4 doses, in elderly (median 68 years) patients with ALL. The dose of InO, initially dosed at 1.8 mg/m$^2$ during the induction phase and at 1.3 mg/m$^2$ during the consolidation phase, was decreased after concerns regarding the risk of developing VOD. Patients enrolled from the 50$^{th}$ onwards received InO at 0.6 mg/m$^2$ on day 2 and 0.3 mg/m$^2$ on day 8 during the induction phase while during consolidations 2 through 4, they received 0.3 mg/m$^2$ on both days 2 and 8, respectively. The dose of dexamethasone in the study was 20 mg/day in 4 day blocks, on days 1 through 4 and 11 through 14; therefore for a shorter period of time and at a fraction of the dose used in DL-1 of Stratum 1B of this trial (20 mg/m$^2$/day on Days 1-5 and Days 15-19).[2]

Furthermore, a Phase I adult trial of InO in combination with CVP (Cyclophosphamide 750 mg/m$^2$, Vincristine 1.4 mg/m$^2$ and max 2 mg, Prednisone 100 mg orally days 1-5) for relapsed/refractory CD22+ ALL also tested the safety and effectiveness of InO in combination with chemotherapy.[3] InO was started at 0.8 mg/m$^2$ and then escalated up to 1.8 mg/m$^2$. The study concluded that the combination was relatively well tolerated with high response rates and low toxicity also in heavily pre-treated patients[3]. Considering a rough equivalence of prednisone 100 mg as equal to approximately 16 mg of dexamethasone[4], also in this

case, InO was combined with lower dosages of glucocorticoids compared to the regimen adopted in Stratum 1B, and it was judged safe and effective at 1.2 mg/m$^2$.

These results support an adjustment of the dose of dexamethasone in the modified UK-ALLR3 regimen to 10 mg/m$^2$/day administered in 5-days blocks from days 1-5 and days 15-19 of Cycle 1, as recommended by the Steering Committee. As explained above, this could reduce the hepatic toxicity and allow patients to receive a more effective dose of InO in combination with this modified UK ALLR3 regimen. We therefore propose a new dose-escalation schema with 10 mg/m$^2$/day dexamethasone in combination with InO, vincristine and IT Methotrexate to provide at least 1.1 mg/m$^2$ during Cycle 1 of InO, yet preferably even a higher dose of InO if this regimen is tolerated.

**Ad2: Inclusion of a "Very High-risk" cohort as Stratum 3**

The second part of this amendment concerns the addition of a third stratum for a new cohort of patients defined as "very high-risk" first relapse (VHR) of BCP-ALL. These children suffer from very-early bone marrow or combined relapse (within 18 months after initial diagnosis, during aggressive chemotherapy), or relapse with high-risk cytogenetic characteristics (independent from the timing), namely KTM2A/AF4, E2A/TCF3-PBX1 t(1;19) or E2A/TCF3-HLF t(17;19), hypodiploidy (<40 chromosomes), and TP53 mutation and/or deletion. This category of VHR patients was recently identified based on the pooled data from ALL-R3 and ALL-REZ BFM 2002 (manuscript submitted), by the IntReALL (International Relapsed ALL Study Group) consortium as a subgroup with dismal outcomes in the prior studies, despite intensive re-induction chemotherapy and HSCT. Therefore, this risk group was spliced out of the traditional HR group in a revised risk-group stratification schema (***Table 2***).[5-7] Given the dismal outcomes with traditional very intensive reinduction modalities, this subgroup is considered a candidate for experimental treatment.

***Table 2. Previous classification from IntReALL 2010 and revised risk-group stratification schema from IntReALL 2020 for BCP-ALL***

A. Definition of IntReALL 2010 Risk Group

| | **Immunophenotype B-cell precursor ALL** | | |
|---|---|---|---|
| **Time point vs Site of relapse** | **Isolated EM** | **Combined BM/EM** | **Isolated BM** |
| **Very early** < 18 months after initial diagnosis | HR | HR | HR |
| **Early** ≥ 18 months after initial diagnosis and < 6 months after completion of 1L therapy | SR | SR | HR |
| **Late** ≥ 6 months after completion of 1L therapy | SR | SR | SR |

B. Definition of IntReALL 2020 Risk Group

| | **Immunophenotype B-cell precursor ALL** | | |
|---|---|---|---|
| **Time point and cytogenetic characteristics vs Site of relapse** | **Isolated EM relapse** | **Combined BM/EM** | **Bone marrow isolated** |
| **Very early** < 18 months after initial diagnosis | HR | **VHR** | **VHR** |
| **Early** ≥ 18 months after initial diagnosis and < 6 months after completion of initial therapy | SR | SR | HR |
| **Late** ≥ 6 months after completion of initial therapy | SR | SR | SR |
| Presence of<br>• **TP53 mutation and/or deletion**<br>• **Hypodiploidy(< 40 chromosomes)**<br>• **t(1;19) TCF3-PBX1 or (17;19) TCF3-HLF**<br>• **KTM2A/AF4** | Refer to time point of relapse | **VHR Independently from timing** | **VHR Independently from timing** |

**Abbreviations**: HR: High-Risk; SR: Standard-Risk; VHR: Very-High-Risk; EM=extramedullary; BM=bone marrow; 1L=first line therapy

C. Subgroup analysis of VHR patients obtained from pooled data of ALL-R3 and ALL-REZ BFM 2002

| EFS at 10 years | PFS at 10 years | OS at 10 years | Induction failure* |
|---|---|---|---|
| 20.6% (SE ±3.4%) | 32.6% (SE ±5.0%) | 25.4% (SE ±3.7%) | 36.9% |

Abbreviations: SE: standard error.
* ≥ 5 blasts after induction treatment

This group of VHR patients has a high rate of induction-failure and induction death between 20% and 60%, while the 10 years EFS is 20.6%.[5,6] Such prognosis is significantly worse than "standard risk" relapse patients that have an EFS of 70%, but also than traditionally defined "high risk" relapse patients with an EFS at 10 years of 30-35%.[8-10] Current standard therapy for very early relapse patients is represented by repeated blocks of intensive chemotherapy, followed by HSCT or CAR-T cells therapy when available. Recent experience with InO in patients with multiple relapse or refractory ALL, as well as trials with other monoclonal antibodies, such as 2 (in consolidation), demonstrated the outstanding activity of these latter strategies. In our InO Phase I study we reported a response rate of 80% in R/R ALL patients treated with InO with MRD-negativity in approximately 80%, and an improved toxicity profile than intensive chemotherapy.[1] Moreover, some of these patients had the characteristics of VHR patients. In a retrospective post-hoc analysis of the phase I cohort, seven "VHR" patients were identified (presenting ≥1 of the following characteristics: hypodiploidy (less than 40 chromosomes), n=2; KTM2A/AF4, n=1; t(1;19)(q23;p13), n=2; relapse <18 months post-diagnosis, n=6); of these, only two were non-responders to InO (one with hypodiploidy, and one with t[1;19] and very early relapse). No specific subgroups of patients with decreased efficacy were identified (manuscript accepted for publication).

Similar considerations were confirmed also by clinical trials comparing blinatumomab with standard chemotherapy as post-reinduction consolidation regimen in first relapse patients. Following the first block of the re-induction chemotherapy of UKALLR3/mitoxantrone regimen, patients were randomized to receive either three intensive chemotherapy blocks of the UKALLR3; or two intensive chemotherapy blocks of the UKALLR3 plus one block of blinatumomab; both strategies were followed by HSCT. Both, activity and safety profile, were significantly better for patients receiving blinatumomab. The intention to treat 2-year OS was 59.2 ± 6.0% for patients treated with chemotherapy versus 79.4 ± 4.5% for patients treated with blinatumomab. Grade 3 to 4 febrile neutropenia, infection, sepsis and mucositis were higher in patients receiving the second and third block of UKALLR3/mitoxantrone regimen, and post-induction deaths occurred in 4 patients on intensive chemotherapy whilst none on blinatumomab. Such results highlight the need of optimizing bridging therapy for relapsing patients who are candidates for consolidation therapy with HSCT or CAR-T cells therapy.[11]

Therefore, in the proposed stratum 3, InO will be studied as reinduction therapy in 1st relapse BCP-ALL VHR-relapsed patients, followed by subsequent consolidation when needed, with CAR-T cells therapy or TBI-based allo-HSCT as definitive treatment. The study aims at establishing the preliminary efficacy activity with respect to ORR using InO as reinduction based on a Simon 2-stage design. Consolidation treatment is left to investigator discretion but will be captured in the CRF.

**AMENDMENT VERSION 3**

**Rationale substantial amendment (Version 3.0)**

In compliance with the InO Pediatric Investigational Plan (PIP) commitment to the PDCO/EMA, the main purpose for this amendment is to add the details for Stratum 1B cohort which will determine the recommended Phase 2 dose (RP2D) of InO in combination with a modified version of UKALL-R3 re-induction chemotherapy, using a Rolling-6 design. The final cohort in which pegylated-asparaginase is added is referred to as Stratum 1B-ASP.

The platelet count criteria required to proceed to subsequent cycles of study therapy and the hematologic DLT definition were updated for Phase 2 and Stratum 1B BCP-ALL patients since during Stratum 1A, several patients failed to recover their platelet count to 50,000/ul yet benefited from study therapy as evidenced by 1) achievement of complete remission after 1 cycle of InO and 2) platelet transfusion independence. These heavily pretreated patients, in particular after an allogenic HSCT, may have a particular impaired hematopoietic reservoir that interfered with hematologic recovery. Under the prior amendment, such patients were not allowed to receive a second cycle of InO while on study.

After the initial analysis of PK data, an optimization of PK sample collection schedule in the protocol amendment 3 was planned. The PK sample collected 4 hours post-InO infusion did not provide additional information and therefore was removed in amendment 3 to reduce patient burden. The samples collected at 2 hours after the start of infusion will now be collected at the end of the infusion in order to determine the maximum concentration of InO. This will also allow a direct comparison of peak concentrations between pediatric patients in this study and adult patients in previously conducted studies.

**AMENDMENT VERSION 2**

**Rationale substantial amendment (Version 2.0)**

As of June 6th 2018, 13 patients were enrolled in stratum 1 in the phase I study.

At DL1 one patient had a dose limiting toxicity (DLT) out of 6 DLT-evaluable patients, and hence DL1 was cleared.

At dose-level 2 (DL2), 2 DLTs were encountered out of 5 evaluable patients (another 2 patients were not evaluable for DLTs as they did not receive at least 2 dosages of InO). The first DLT occurred in a patient who did not regenerate WBC counts at Cycle 1 Day 42 (patient 9-20; see narrative below) despite being in complete remission (CR) after 1 cycle of InO; and a different patient experienced persisting Grade 4 transaminase elevation (patient 15-04), despite also being in CR after 1 cycle of InO. Both patients with DLTs received all 3 dosages of InO during cycle 1 without being clinically ill, and hence the steering committee does not consider these toxicities as truly dose-limiting.

Moreover, these DLTs were not consistent with the pattern that was observed in the adult phase 1 study, nor were they in line with the pediatric compassionate use experience described by Bhojwani et al (ASCO 2017; manuscript submitted). The table below shows the toxicities during cycle 1 in the 51 children treated with InO on compassionate use basis as reported by Bhojwani et al (ASCO 2017). The typical pattern for transaminases is to rise at day 8, potentially as a result of leukemic cell death, but this is not consistent with the late rise we observed in patient 15-04 (see case narratives below).

**Table 3*****: Toxicities during cycle 1, including transaminase elevations, in 51 children treated with InO on compassionate use basis, reported by Bhojwani et al, ASCO 2017.***

| Toxicity | Grade 1-2 | Grade 3* | Grade 4 | Unknown grade | Total |
|---|---|---|---|---|---|
| ALT increase | 6 (11.8%) | 3 (5.9%) | | | 9 (17.6%) |
| AST increase | 8 (15.7%) | 2 (3.9%) | | | 10 (19.6%) |
| GGT increase | 2 (3.9%) | 1 (2.0%) | 1 (2.0%) | | 4 (7.8%) |
| Hyperbilirubinemia | | 1 (2.0%) | | | 1 (2.0%) |
| Fever | 9 (17.6%) | | | | 9 (17.6%) |
| Febrile neutropenia | 2 (3.9%) | 6 (11.8%) | | | 8 (15.7%) |
| Infection | 4 (7.8%) | 8 (15.7%) | 2 (3.9%) | 1 (2.0%) | 15 (29.4%) |
| Bone pain | 3 (5.9%) | 1 (2.0%) | | | 4 (7.8%) |
| Infusion reaction | | 2 (3.9%) | | | 2 (3.9%) |
| Vomiting | 2 (3.9%) | | | 1 (2.0%) | 3 (5.9%) |
| Diarrhea | 1 (2.0%) | | | 1 (2.0%) | 2 (3.9%) |
| Tumor lysis syndrome | | 2 (3.9%) | | | 2 (3.9%) |
| Bleeding | 1 (2.0%) | 1 (2.0%) | | | 2 (3.9%) |
| Electrolyte disturbances | 7 (13.7%) | 3 (5.9%)^ | | | 10 (19.6%) |

*Additional grade 3 toxicities noted in one patient each: anorexia, hypertension, hypertriglyceridemia, paroxysmal atrial tachycardia

^Grade 3 electrolyte disturbances: hypokalemia (2), hypocalcemia (1)

In addition, the Children's Oncology Group (COG) is conducting a phase 2 study with InO (COG AALL1621, NCT02981628) for pediatric patients with relapsed/refractory (R/R) ALL (second relapse or later) using the approved adult dose of 1.8 mg/m$^2$ during Cycle 1 (without a prior dose-escalation study), and have not

observed the degree of transaminase elevation we observed in patient 15-04 in the 24 patients they treated. However, COG did observe 4 cases of hepatic veno-oclusive disease/sinusoidal obstruction syndrome (VOD/SOS) after allogeneic stem cell transplant (allo SCT), which is in line with the current safety information available for InO (personal communication, Dr M O'Brien, PI for COG AALL1621, safety teleconference June7th 2018; and COG AALL1621 Fall 2018 Study Progress Report – quoted with permission). Three of these cases of VOD/SOS resolved with defibrotide therapy. COG also observed a few patients with insufficient platelet regeneration, in line with the toxicity we observed in patient 9-20.

**Patient narratives**

**Dose-level 1**

**Patient 10-20**. This 4 year-old patient with 2nd relapse of ALL (WBC 5.3 x$10^9$/l, no prior SCT) received only 1 dose of InO on April 6 2018, and rapidly developed grade 4 transaminase elevation during Cycle 1, which lasted longer than 7 days, and therefore was considered a DLT as defined in the protocol.

Concomitant medications consisted of the following:

- Regular Infusion (NaCl 4,5% / G2,5%, Heparin 400E 3000ml/m²
- Onandsetron 4 mg IV x1
- Methylprednisolone 17mg IV
- Furosemide 8 mg IV x3
- Pantoprazole 20 mg PO x1
- Movicol x 1 pack PO x2
- Allopurinol 80 mg PO x3

No pre-existing liver disease was known. HA/B/C-Virus serologically negative. CMV/HSV diagnostics were negative.

The patient was taken off study, despite the fact that response evaluation showed that the patient was in CR with a low level of minimal residual disease (MRD) following 1 dose of InO.

***Table 4. Liver functions of patient 10-20. Liver functions increased after one infusion with InO. There were no liver function abnormalities present at screening.***

| **Visit**<br>*Normal range for a 4 year old female* | **Date** | **ALT (U/L)**<br>*<31* | **AST (U/L)**<br>*<53* | **Total Bilirubin (mg/dL)**<br>*<1.2* |
|---|---|---|---|---|
| | 21-03-2018 | 39 | 31 | 0.28 |
| | 25-03-2018 | 26 | 20 | 0.24 |
| Screening | 04-04-2018 | 32 | 26 | |
| | 05-04-2018 | 27 | 21 | |
| C1D1 (Pre-dose) | 06-04-2018 | 69 | 47 | 0.43 |
| | 07-04-2018 | 441 | 233 | |
| | 08-04-2018 | 1155 | 488 | |
| | 09-04-2018 | 1419 | 568 | 0.68 |
| | 10-04-2018 | 2318 | 997 (peak) | |
| | 11-04-2018 | 2520 (peak) | 912 | |
| | 12-04-2018 | 2004 | 403 | 1.0 |
| C1D8 – InO not given due to DLT | 13-04-2018 | 1361 | 158 | |
| | 14-04-2018 | 949 (declining) | 76 (declining) | |
| | 15-04-2018 | 696 | 53 | |
| | 17-04-2018 | 427 | 38 | 0.64 |

**Dose-level 2 patients not evaluable for DLTs**

**Patient 08-30.** This 7 year-old patient with 2nd relapse of ALL post allo-SCT (WBC $7.1x10^9/l$) received only 1 dose of InO on March 2nd 2018. Next, the patient suffered from a suspected fungal infection and gastrointestinal bleeding. Biopsy showed graft vs. host disease (GVHD). It was concluded that these side-effects were not related to InO but due to prior treatment, but as they were clinically severe, the patient could not proceed with InO treatment. This patient therefore was considered unevaluable for DLTs.

**Patient 12-06.** This 14 year-old patient with 2nd relapse of ALL post allo-SCT (WBC $0.4x10^9/l$) received only 1 dose of InO on April 17th 2018. Two days (April 19, 2018) after the first InO infusion, the patient suffered from sepsis (clinically behaved as Gram-negative sepsis with hypotension requiring inotropic pressure support, despite negative blood cultures). This was associated with a sharp rise in bilirubin values which normalized rapidly, and hence was considered a consequence of sepsis and not consistent with a diagnosis of VOD/SOS. Hepatic transaminases were not elevated. The patient was started on defibrotide initially because the diagnosis of VOD was considered with the sharp rise in bilirubin plus an enlarged liver and some upper quadrant pain, but this did not persist, and this clinical scenario was considered most consistent with Gram-negative sepsis.

***Table 5. Bilirubin values in patient 12-06. Only 1 dose of InO was infused.***

| **Visit** | **Date** | **Total Bilirubin (mg/dL)** *normal range <1.2* |
|---|---|---|
| Screening | 06-04-2018 | 0.45 |
| C1D1 InO | 17-04-2018 | 0.74 |
| | 19-04-2018 | 3.22 |
| | 20-04-2018 | 9.88 (peak) |
| | 22-04-2018 | 2.05 |
| | 26-04-2018 | 1.98 |
| EOT | 02-05-2018 | 1.05 |
| | 04-05-2018 | 0.85 |

The patient was not re-exposed to InO after sepsis, and hence was considered non-evaluable for DLTs.

**Dose-level 2 patients with DLTs**

**Patient 09-20.** This 9 year-old patient was enrolled with a 1st refractory relapse of E2A/PBX1+ ALL (WBC $0.2x10^9/l$), and received all 3 dosages of InO in the first cycle. There was no preceding SCT. During the screening period, the patient suffered from severe disease-related radicular pain requiring Morphine and Ketamine treatment. She responded rapidly to InO and became pain-free. This patient failed to recover blood counts at Cycle 1 Day 42, although the marrow was only slightly cytopenic and M1 (CR). MRD at April 26th was $2x10^{-3}$ (day 38). The peripheral blood values at day 42 were: Leucocytes 0.8/nl, Granulocytes 0.15/nl, Platelets 47/nl (post transfusion). The reason for this lack of regeneration was not clear. Disease-related marrow damage or cytokine activation within the marrow stroma was considered. No hemophagocytosis was visible in the marrow. Lack of hematopoietic regeneration at day 42 in the absence of leukemia is defined in the protocol as a DLT.

**Patient 15-04.** This 14 year-old patient was enrolled with 2nd relapse of ALL (WBC $2.8\ x10^9/l$). Hepatomegaly was noted at baseline. The patient was treated with InO on April 26th, May 3rd and May 9th, 2018. At day 8 there was a sharp rise in transaminases, without any increase in bilirubin. The lab result only came available after the 2nd dose was already infused (there was no language in the protocol withholding InO in case of transaminitis on day 8). The transaminases then dropped at day 15 and 22, but increased again later (see table below). This initial rise may have been related to leukemia, although there was no other evidence of tumor-lysis syndrome. There was no clear explanation for the late rise in transaminases which prevented continuation of InO treatment. The patients was receiving Ambisome prophylaxis. No azoles or other co-medication could be identified to explain this rise; no infectious

complications were found and viral tests were negative. Clinically the patient was in a very good condition and did achieve CR after 1 cycle of InO. Because the transaminitis persisted and was not clearly attributable to causes other than InO, this event was considered a DLT.

***Table 6. Liver functions in patient 15-04 in relation to the 3 InO infusions of the first cycle***

| **InO Dose** | **Date** | **ALT (IU/L)** | **AST (IU/L)** | **Total Bilirubin (µmol/L)** |
|---|---|---|---|---|
| *Normal ranges* | | *<47* | *<47* | *<25* |
| Screening | 20-04-2018 | 13 | 24 | 5 |
| C1D1 InO | 26-04-2018 | 81 | 32 | 10 |
| C1D8 InO | 03-05-2018 | 937 | 484 | 6 |
| | 07-05-2018 | 515 (declined despite recent InO dose) | 93 | 8 |
| C1D14 InO | 09-05-2018 | 368 | 78 | 5 |
| | 14-05-2018 | 454 | | 5 |
| C1D22 | 17-05-2018 | 384 | 164 | 5 |
| | 22-05-2018 | 1140 | 574 | 3 |
| EOT | 07-06-2018 | 200 | 110 | 4 |

Steroids were not continued during study therapy. Patient achieved CR after cycle 1 with a low MRD rate (flow <0.001 in BM) and was physically well and asymptomatic.

**Adapted design for stratum 1A (dose-finding cohort)**

Given the observed DLTs at DL2, of which at least the one with transaminase elevation was considered atypical (and the patient tolerated the dose clinically very well and achieved CR3), the dose was temporarily de-escalated to DL1 while awaiting approval of this amendment. Six additional DLT-evaluable patients will be recruited in DL1 to better characterize the safety of InO in the pediatric population. This dose level will again be considered safe if a maximum of 1 out of these additional 6 evaluable patients experience a DLT, in line with the Rolling-6 principle (i.e., ≤3 DLTs out of the total of 12 DLT-evaluable patients at this dose level, thus less than 33%.

When DL1 is once more considered safe, the dose will be re-escalated to DL2, the approved adult dose, and again up to 6 DLT-evaluable patients will be recruited to confirm safety in this dose-level. We will consider this a new cohort of 3-6 patients, and apply the Rolling-6 design characteristics as described in Section 9.4.1. Prior to re-escalation to DL2, IRB aproval for this amendment needs to be obtained.
Additional changes were made to align protocol with Clinical Trial Agreements with participating countries and local institutional review boards/ethics committees, and to resolve issues raised to date during trial conduct.

**ABBREVIATIONS**

| | |
|---|---|
| ADA | ANTI-DRUG ANTIBODY |
| AE | ADVERSE EVENT |
| AESI | ADVERSE EVENT OF SPECIAL INTEREST |
| ALL | ACUTE LYMPHOBLASTIC LEUKEMIA |
| ALLO | ALLOGENEIC |
| ALT | ALANINE AMINOTRANSFERASE |
| ANC | ABSOLUTE NEUTROPHIL COUNT |
| ASP | ASPARAGINASE |
| AST | ASPARTATE AMINOTRANSFERASE |
| AUC | AREA UNDER THE CURVE |
| AR | ADVERSE REACTION |
| BCP | B-CELL PRECURSOR |
| BFM | BERLIN-FRANKFURT-MÜNSTER COLLABORATIVE STUDY GROUP |
| BM | BONE MARROW |
| BP | BLOOD PRESSURE |
| BSA | BODY SURFACE AREA |
| CBA | CHROMOSOME BANDING ANALYSIS |
| CI | CHIEF INVESTIGATOR |
| CIs | CONFIDENCE INTERVALS |
| CNS | CENTRAL NERVOUS SYSTEM |
| CNS 1/2/3 | CENTRAL NERVOUS SYSTEM DISEASE STATUS |
| COG | CHILDREN'S ONCOLOGY GROUP |
| CR | COMPLETE REMISSION |
| CRi | COMPLETE REMISSION WITH INCOMPLETE HEMATOLOGIC RECOVERY |
| CRp | COMPLETE REMISSION WITH INCOMPLETE PLATELET RECOVERY |
| CRF | CASE REPORT FORM |
| CSF | CEREBROSPINAL FLUID |
| CT | COMPUTERISED TOMOGRAPHY |
| CTCAE | COMMON TERMINOLOGY CRITERIA FOR ADVERSE EVENTS |
| CTFG | CLINICAN TRIAL FACILITATION GROUP |
| DFS | DISEASE FREE SURVIVAL |
| DLBCL | DIFFUSE LARGE B-CELL LYMPHOMA |
| DLT | DOSE LIMITING TOXICITY |
| DMC | DATA MONITORING COMMITTEE |
| DSUR | DEVELOPMENT SAFETY UPDATE REPORT |
| ECHO | ECHOCARDIOGRAM |
| EM | EXTRAMEDULLARY |
| EMA | EUROPEAN MEDICINES AGENCY |
| EFS | EVENT FREE SURVIVAL |
| FDA | FOOD AND DRUG ADMINISTRATION |
| GCP | GOOD CLINICAL PRACTICE |
| GFR | GLOMERULAR FILTRATION RATE |
| GVHD | GRAFT VERSUS HOST DISEASE |
| HB | HEMOGLOBIN |
| HR | HEART RATE |
| HSV | HERPES SIMPLEX VIRUS |
| HSCT | HEMATOPOIETIC STEM CELL TRANSPLANT |
| HTD | HIGHEST TREATMENT DOSE |
| IB | INVESTIGATOR BROCHURE |
| ICF | INFORMED CONSENT FORM |
| ICH | INTERNATIONAL CONFERENCE ON HARMONISATION |
| IM | INTRAMUSCULAR |
| IMP | INVESTIGATIONAL MEDICINAL PRODUCT |
| InO | INOTUZUMAB OZOGAMICIN |

| | |
|---|---|
| IntReALL | INTERNATIONAL STUDY FOR TREATMENT OF CHILDHOOD RELAPSED ALL |
| ISF | INVESTIGATOR SITE FILE |
| IT | INTRATHECAL |
| ITCC | INNOVATIVE THERAPIES IN CHILDREN WITH CANCER |
| IV | INTRAVENOUS |
| LLN | LOWER LIMIT OF NORMAL |
| LP | LUMBAR PUNCTURE |
| MedDRA | MEDICAL DICTIONARY FOR REGULATORY ACTIVITIES |
| MHRA | MEDICINES AND HEALTHCARE PRODUCTS REGULATORY AGENCY |
| MRD | MINIMAL RESIDUAL DISEASE |
| MRI | MAGNETIC RESONANCE IMAGING |
| MTD | MAXIMUM TOLERATED DOSE |
| NCC | NATIONAL COORDINATING CENTRE |
| NCI | NATIONAL COORDINATING INVESTIGATOR |
| ORR | OVERALL RESPONSE RATE |
| OS | OVERALL SURVIVAL |
| OTC | OVER THE COUNTER |
| PD | PROGRESSIVE DISEASE |
| PEG-ASP | PEGYLATED-ASPARAGINASE |
| PFS | PROGRESSION FREE SURVIVAL |
| PI | PRINCIPAL INVESTIGATOR |
| PIS | PATIENT INFORMATION SHEET |
| PK | PHARMACOKINETICS |
| PO | ORALLY |
| PR | PARTIAL RESPONSE |
| REC | RESEARCH ETHICS COMMITTEE |
| RR | RESPONSE RATE |
| RP2D | RECOMMENDED PHASE 2 DOSE |
| SAE | SERIOUS ADVERSE EVENT |
| SAR | SERIOUS ADVERSE REACTION |
| SCT | STEM CELL TRANSPLANT |
| SD | STABLE DISEASE |
| SOS | SINUSOIDAL OBSTRUCTION SYNDROME |
| SPC | SUMMARY OF PRODUCT CHARACTERISTICS |
| SUSAR | SUSPECTED UNEXPECTED SEVERE ADVERSE REACTION |
| TMG | TRIAL MANAGEMENT GROUP |
| TSC | TRIAL STEERING COMMITTEE |
| UKALL | UNITED KINGDOM ACUTE LYMPHOBLASTIC LEUKEMIA PROTOCOL |
| UKALL-R3 | A COLLABORATIVE UKALL TRIAL FOR RELAPSED AND REFRACTORY ALL |
| ULN | UPPER LIMIT OF NORMAL |
| VOD | VENO-OCCLUSIVE DISEASE (LIVER) |
| WBC | WHITE BLOOD CELLS |

## TRIAL SYNOPSIS

### Title

A phase I/II study of Inotuzumab Ozogamicin (InO) as a single agent and in combination with chemotherapy for pediatric CD22-positive relapsed/refractory Acute Lymphoblastic Leukemia. *Study ITCC-059*

### Trial Design

This is a Phase 1-2, multicenter, international, single-arm, open-label study designed to identify a recommended dose of InO administered IV either as monotherapy or in combination with chemotherapy (Stratum 1B) to pediatric patients with relapsed/refractory CD22 positive acute lymphoblastic leukemia (ALL), and to estimate the efficacy, safety, and tolerability of the selected InO dose and evaluate PK and PD in this patient population. In addition, an explorative cohort of other CD22-positive B-cell malignancies will be evaluated.

### Patient Population

This protocol contains 3 disease strata:

- **Stratum 1**: relapsed (2$^{nd}$ or greater relapse or first relapse after transplant)/refractory (R/R) CD22 positive B-cell precursor (BCP)-ALL

  **In stratum 1**, the BCP-ALL stratum, three different cohorts of patients are planned:

  - **Stratum 1A**: in this cohort we will determine the maximum tolerated dose (MTD) or the Recommended Phase 2 Dose (RP2D) of single-agent InO using a Rolling-6 design
  - **Phase 2 Cohort**: in this cohort we will assess the preliminary activity [overall response rate, (ORR) defined as the rate of patients who achieve complete remission (CR), CR with incomplete hematologic recovery (CRi), or CR with incomplete platelet recovery (CRp)] after single-agent InO using a single-stage design
  - **Stratum 1B**: in this cohort we will determine the RP2D of InO in combination with a modified version of UKALL-R3 re-induction chemotherapy, using a Rolling-6 design.
    - Note that the final cohort in which pegylated-asparaginase (PEG-ASP) is added is referred to as Stratum 1B-ASP

- **Stratum 2**: other CD22 positive B-cell malignancies

- **Stratum 3:** VHR first relapse CD22-positive BCP-ALL patients (defined as isolated bone marrow or combined relapse < 18 months from initial diagnosis excluding patients transplanted in 1$^{st}$ CR, and/or cytogenetic-high risk characteristics: KTM2A/AF4, E2A/TCF3-PBX1 t(1;19) or E2A/TCF3-HLF t(17;19), hypodiploidy (less than 40 chromosomes), TP53 mutation and/or deletion (see table below). In this cohort we will assess the preliminary activity (ORR defined as CR, CRi, CRp) in VHR first relapse ALL, after single-agent InO, using a Simon 2-stage design.

***Definition of IntReALL 2020 Risk Group (see Table 2B).***

| | **Immunophenotype B-cell precursor ALL** | | |
|---|---|---|---|
| **Time point and cytogenetic characteristics vs Site of relapse** | **Isolated EM relapse** | **Combined BM/EM** | **Bone marrow isolated** |
| **Very early** < 18 months after initial diagnosis | HR | **VHR** | **VHR** |
| **Early** ≥ 18 months after initial diagnosis and < 6 months after completion of initial therapy | SR | SR | HR |
| **Late** ≥ 6 months after completion of initial therapy | SR | SR | SR |
| Presence of<br>• **TP53 mutation and/or deletion**<br>• **Hypodiploidy(< 40 chromosomes)**<br>• **E2A/TCF3-PBX1 t(1;19) or E2A/TCF3-HLF t(17;19)**<br>• **KTM2A/AF4** | Refer to time point of relapse | **VHR Independently from timing** | **VHR Independently from timing** |

**Abbreviations**: HR: High-Risk; SR: Standard-Risk; VHR: Very-High-Risk; EM=extramedullary; BM=bone marrow;

## Objectives

### Primary Objectives

Stratum 1

- Stratum 1A:
  To establish the MTD or the RP2D of single agent InO when administered in children with CD22-positive relapsed/refractory BCP-ALL.
  Note that:
    - if the MTD is not reached at the highest tested dose, no further dose-escalation will be performed.
    - to establish the RP2D for further study in the Phase 2 cohort, additional modelling will be performed regarding cumulative toxicity (as described in Section 9)

- Phase 2 Cohort:
  To establish the preliminary clinical activity (efficacy) activity with respect to ORR of single agent InO when administered in children with CD22-positive relapsed/refractory BCP-ALL.
- Stratum 1B (and 1B-ASP):
  To determine the RP2D of InO in children with CD22-positive relapsed/refractory BCP-ALL in combination with a modified UKALL-R3 based re-induction regimen.

Stratum 2 (EXPLORATORY)

- To explore the safety and tolerability of InO as a single agent in children with relapsed/refractory other CD22 positive B-cell malignancies.

Stratum 3

- To establish the preliminary clinical activity (efficacy) with respect to ORR of single agent InO when administered in children with CD22-positive VHR 1st relapse BCP-ALL, excluding patients transplanted in 1$^{st}$ CR.

### Secondary Objectives

Stratum 1A and Phase 2 Cohort (BCP- ALL patients)

- To determine the safety and tolerability of InO as a single agent during cycle 1; the cumulative toxicities in patients receiving multiple cycles of InO; as well as after subsequent allogeneic hematopoietic stem cell transplant (allo-HSCT) or CAR-T cells therapy.
- To determine the overall hematological response rate in these patients:
  - after cycle 1
  - as well as the overall best response (Stratum 1A only; this is the primary objective for Phase 2 cohort).
- To determine minimal residual disease (MRD) levels in responding patients, including the percentage of patients with a complete MRD response (see Appendix 2.1)
  - after cycle 1
  - as well as the best ORR.

- To describe the durability of response and long-term follow-up, including the number of patients that undergo HSCT or CAR-T cells therapy after treatment with InO as consolidation, the cumulative incidence of non-response or relapse, the cumulative incidence of non-relapse mortality, the event-free survival (EFS) and overall survival (OS).
- To determine the serum pharmacokinetic parameters of unconjugated calicheamicin and InO in the pediatric population.
- To assess the relationship between CD22 receptor density, white blood cell count (WBC) at start of treatment, CD22 saturation kinetics, cytogenetics, and in-vitro calicheamicin resistance to clinical response to InO.
- To assess for the persistence of B-Cell aplasia and hypogammaglobulinemia in responding patients following treatment with InO.
- To assess the number of patients developing anti-drug antibodies (ADAs; immunogenicity).

<u>Stratum 1B and Stratum 1B-ASP (BCP ALL patients)</u>

- To determine the safety and tolerability of InO in combination with a modified UKALL-R3 re-induction chemotherapy regimen (1 or 2 cycles), and the cumulative toxicities in patients receiving multiple InO-based cycles, as well as after subsequent allogeneic HSCT.
- To determine the overall hematological response rate in these patients:
  - after cycle 1
  - as well as the overall best response
- To determine MRD levels in responding patients, including the percentage of patients with a complete MRD response (see appendix 2.1 for definition):
  - after cycle 1
  - as well as the best ORR.
- To describe the durability of response and long-term follow-up, including the number of patients that undergo HSCT or CAR-T cells therapy after study treatment as consolidation, the cumulative incidence of non-response or relapse, the cumulative incidence of non-relapse mortality, the EFS and OS.
- To determine the serum pharmacokinetic parameters of unconjugated calicheamicin and InO when added to a modified reinduction UKALL-R3 chemotherapy regimen without (stratum 1B), then with ASP (stratum 1B-ASP).
- To assess the relationship between CD22 expression and clinical response to InO
- To assess the number of patients developing CD22-negative relapse

<u>Stratum 2 (Other B-cell malignancies)</u>

- To determine the response rate in these patients:
  - after cycle 1
  - as well as the overall best response.
- To describe the durability of response and long-term follow-up, including the number of patients that undergo stem-cell transplant after treatment with InO, the cumulative incidence of non-response or relapse, the cumulative incidence of non-relapse mortality, the EFS and OS.
- To determine the serum pharmacokinetic parameters of unconjugated calicheamicin and InO in the pediatric population.

- To assess for the persistence of B-Cell aplasia and hypogammaglobulinemia in responding patients following treatment with InO.

  To assess the number of patients developing ADAs (NOT valid for patients enrolled after Amendment 4).

<u>Stratum 3 (VHR)</u>

- To determine the safety and tolerability of InO as a single agent during cycle 1; the cumulative toxicities in patients receiving multiple cycles of InO; as well as after subsequent allogeneic hematopoietic stem cell transplant (allo-HSCT) or CAR-T cells therapy.
- To determine the overall hematological response rate in these patients after cycle 1
- To determine minimal residual disease (MRD) levels in responding patients, including the percentage of patients with a complete MRD response (see Appendix 2.1)
  - after cycle 1
  - as well as the best ORR.
- To describe the durability of response and long-term follow-up, including the number of patients that undergo HSCT or CAR-T cells therapy after treatment with InO as consolidation, the cumulative incidence of non-response or relapse, the cumulative incidence of non-relapse mortality, the EFS and overall survival (OS).
- To assess the relationship between CD22 receptor density, white blood cell count (WBC) at start of treatment, cytogenetics, and in-vitro calicheamicin resistance to clinical response to InO.
- To assess the persistence of B-Cell aplasia and MRD negativity in responding patients following the treatment with InO and the implications for CAR-T cells therapy.

## Outcome Measures

### Stratum 1A

**Primary endpoints for patients enrolled in Stratum 1A**

Dose-limiting toxicities (DLTs) during the first cycle of therapy.

**Secondary endpoints for patients enrolled in Stratum 1A**

1. Safety and tolerability:
   - AEs, as characterized by type, frequency, severity (as graded using CTCAE, v4.03), timing, seriousness, and relation to study therapy, during the first and subsequent cycles of therapy.
   - Occurrence of toxic death; i.e., death attributable to InO therapy.
   - Occurrence of hepatic veno-occlusive disease (VOD)/sinusoidal obstruction syndrome (SOS) during or after therapy with InO.
   - Laboratory abnormalities as characterized by type, frequency, severity and timing.
   - The cumulative incidence of non-relapse mortality, defined as the cumulative probability of non-relapse mortality, with time calculated between start of study treatment and death due to other causes than relapsed or refractory leukemia or lymphoma, accounting for competing events.

2. Measures of anti-leukemic activity:
   - ORR, defined as CR, CRi, or CRp both after cycle 1 as well as the best response over multiple cycles of InO therapy (see Appendix 2 for definitions).
   - MRD levels, including the percentage of patients who become MRD-negative (complete MRD response defined as an MRD-level < $1x10^{-4}$), after cycle 1, as well as the overall best response (MRD-negativity) over multiple cycles.
   - Duration of response, defined as the time between achieving response (CR, CRi or CRp) after starting study treatment and documented relapse or death.
   - Number and percentage of patients being transplanted and those receiving CAR T-cell therapy after treatment with InO.
   - EFS, defined as the time between start of study treatment and first event including failure to achieve CR/CRp/CRi (calculated as an event on day 0), relapse, death of any cause and second malignancies.
   - Overall survival, defined as time to death following start of study treatment.
   - The cumulative incidence of non-response or relapse, defined as the cumulative probability of non-response or relapse, with time calculated between start of study treatment and relapse and with non-responders included as an event on day 0. Non-relapse death is considered a competing event.

3. Serum pharmacokinetic parameters of InO and unconjugated calicheamicin.

4. Pharmacodynamics parameters
   - Relationship between response (ORR) and CD22 expression levels and WBC.
   - Relationship between response (ORR) and CD22 saturation kinetics.
   - Relationship between response (ORR) and calicheamicin sensitivity.
   - Clonal evolution (CD22-negativity) and relation to loss of response.

5. Other endpoints
    - The percentage of patients responding to InO (ORR) without adequate recovery of CD19-positive B-cells (below lower limit of normal (LLN) for age) or immunoglobulins (below LLN for age). Following 4 weeks, 10 weeks, 3, 6 and 12 months after treatment with InO, excluding patients who have been transplanted from the date of HSCT or have received CAR-T cells therapy.
    - Percentage of patients who exhibit anti-drug antibodies (ADA) (NOT valid for patients enrolled after Amendment 4).

__Phase 2 cohort__

**Primary endpoints for patients enrolled in Phase 2 cohort**

ORR, defined as the percentage of patients with CR, CRi, CRp, measured as best response during InO treatment (see Appendix 2 for definitions).

**Secondary endpoints for patients enrolled in Phase 2 cohort**

1. Safety:
    - AEs, as characterized by type, frequency, severity (as graded using CTCAE v4.03), timing, seriousness, and relation to study therapy, during the first and subsequent cycles of therapy.
    - Occurrence of toxic death; i.e., death attributable to InO therapy.
    - Occurrence of VOD/SOS during or after therapy with InO.
    - Laboratory abnormalities as characterized by type, frequency, severity and timing.
    - The cumulative incidence of non-relapse mortality, defined as the cumulative probability of non-relapse mortality, with time calculated between start of study treatment and death due to other causes than relapsed or refractory leukemia or lymphoma, accounting for competing events.

2. Other measures of anti-leukemic activity:
    - ORR after cycle 1.
    - Minimal residual disease levels, including the percentage of patients who become MRD-negative (complete MRD response defined as an MRD-level < $1x10^{-4}$), after cycle 1, as well as the best response (MRD-negativity) over multiple cycles.
    - Duration of response, defined as the time between achieving response (CR, CRi or CRp) after starting study treatment and documented relapse or death.
    - Number and percentage of patients being transplanted and those receiving CAR T-cell therapy after treatment with InO.
    - EFS, defined as the time between start of study treatment and first event including failure to achieve CR/CRp/CRi (calculated as an event on day 0), relapse, death of any cause and second malignancies.
    - Survival, defined as time to death following start of study treatment.
    - The cumulative incidence of non-response or relapse, defined as the cumulative probability of non-response or relapse, with time calculated between start of study treatment and relapse and with non-responders included as an event on day 0. Non-relapse death is considered a competing event.

3. Serum pharmacokinetic parameters of InO and unconjugated calicheamicin.

4. Pharmacodynamics parameters
    - Relationship between response (ORR) and CD22 expression levels and WBC.
    - Relationship between response (ORR) and CD22 saturation kinetics.
    - Relationship between response (ORR) and calicheamicin sensitivity.
    - Clonal evolution (CD22-negativity) and relation to loss of response.

5. Other endpoints
    - The percentage of patients responding to InO (ORR) without adequate recovery of CD19-positive B-cells (below LLN for age) or immunoglobulins (below LLN for age) following 4 weeks, 10 weeks, 3, 6 and 12 months after treatment with InO, excluding patients who have been transplanted from the date of HSCT or have received CAR-T cells therapy.

      Percentage of patients who exhibit ADA (NOT valid for patients enrolled after Amendment 4).

**<u>Stratum 1B and 1B-ASP</u>**

**Primary endpoint**

Dose-limiting toxicities (DLTs) during the first cycle of InO when added to a modified UKALL-R3 re-induction chemotherapy regimen without or with ASP.

**Secondary endpoints (Same definitions as above for Stratum 1A)**

1. Safety:
    - AEs
    - Occurrence of toxic death; i.e., death attributable to InO therapy.
    - Occurrence of hepatic VOD/SOS during or after therapy with InO.
    - Laboratory abnormalities
    - Cumulative incidence of non-relapse mortality

2. Other measures of anti-leukemic activity:
    - ORR
    - MRD levels
    - Duration of response
    - Number and percentage of patients being transplanted and those receiving CAR T-cell therapy after treatment with InO.
    - EFS
    - Overall survival
    - Cumulative incidence of non-response or relapse

3. Serum pharmacokinetic parameters of InO and unconjugated calicheamicin during treatment combined with modified UKALL-R3 re-induction regimen both with and without pegylated asparaginase.

4. Pharmacodynamics parameters
    - Relationship between response (ORR) and CD22 expression levels

- Clonal evolution (CD22-negativity) and relation to loss of response.

**Stratum 2**

**Primary endpoint for patients enrolled in Stratum 2**

Safety and tolerability:

- AEs, as characterized by type, frequency, severity (as graded using CTCAE v4.03, timing, seriousness, and relation to study therapy, during the first and subsequent cycles of therapy.
- Occurrence of toxic death; i.e., death attributable to InO therapy.
- Occurrence of hepatic VOD/SOS during or after therapy with InO.
- Laboratory abnormalities as characterized by type, frequency, severity and timing.
- The cumulative incidence of non-relapse mortality, defined as the cumulative probability of non-relapse mortality, with time calculated between start of study treatment and death due to other causes than relapsed or refractory leukemia or lymphoma, accounting for competing events.

**Secondary endpoints for patients enrolled in Stratum 2**

1. Measures of anti-tumor activity:
   - Overall remission rate (CR and PR) both after cycle 1 as well as overall best response in patients receiving multiple cycles of InO therapy (see Appendix 2 for definitions).
   - Duration of response, defined as the time between achieving response (CR and PR) after starting study treatment and documented relapse or death.
   - Number and percentage of patients being transplanted and those receiving CAR T-cell therapy after treatment with InO.
   - EFS, defined as the time between start of study treatment and first event including failure to achieve CR/PR (calculated as an event on day 0), relapse, death of any cause and second malignancies.
   - Overall survival, defined as time to death following start of study treatment.
   - The cumulative incidence of non-response or relapse, defined as the cumulative probability of non-response or relapse, with time calculated between start of study treatment and relapse and with non-responders included as an event on day 0. Non-relapse death is considered a competing event.
2. Serum pharmacokinetic parameters of InO and unconjugated calicheamicin.
3. Other endpoints:
   - The percentage of patients responding to InO (ORR) without adequate recovery of CD19-positive B-cells (below LLN for age) or immunoglobulins (below LLN for age) following 4 weeks, 10 weeks, 3, 6 and 12 months after treatment with InO, excluding patients who have been transplanted from the date of HSCT or have received CAR-T cells therapy.
   - Percentage of patients who exhibit ADA (NOT valid for patients enrolled after Amendment 4).

**Stratum 3**

**Primary endpoints for patients enrolled in the VHR cohort**

ORR, defined as the percentage of patients with CR, CRi, CRp, measured as best response of InO treatment (see Appendix 2 for definitions) as a single agent in CD22-positive VHR 1st relapse BCP ALL patients.

**Secondary endpoints for patients enrolled in the VHR cohort**

1. Safety:
   - AEs, as characterized by type, frequency, severity (as graded using CTCAE v4.03), timing, seriousness, and relation to study therapy, during the first and subsequent cycles of therapy.
   - Occurrence of any induction death and/or toxic death attributable to InO therapy.
   - Occurrence of VOD/SOS during or after therapy with InO.
   - Laboratory abnormalities as characterized by type, frequency, severity and timing.
   - The cumulative incidence of non-relapse mortality, defined as the cumulative probability of non-relapse mortality, with time calculated between start of study treatment and death due to other causes than relapsed or refractory leukemia or lymphoma, accounting for competing events.

2. Other measures of anti-leukemic activity:
   - ORR after cycle 1
   - Minimal residual disease levels, including the percentage of patients who become MRD-negative (complete MRD response defined as an MRD-level < $1x10^{-4}$) after cycle 1, as well as the best response (MRD-negativity) over multiple cycles.
   - Duration of response, defined as the time between achieving response (CR, CRi or CRp) after starting study treatment and documented relapse or death.
   - Number and percentage of patients being transplanted and those receiving CAR T-cell therapy after treatment with InO.
   - EFS, defined as the time between start of study treatment and first event including failure to achieve CR/CRp/CRi (calculated as an event on day 0), relapse, death of any cause and second malignancies.
   - Survival, defined as time to death following start of study treatment.
   - The cumulative incidence of non-response or relapse, defined as the cumulative probability of non-response or relapse, with time calculated between start of study treatment and relapse and with non-responders included as an event on day 0. Non-relapse death is considered a competing event.
   - To study the interval between InO re-induction and CAR-T cells therapy based on MRD negativity and B cell aplasia after InO re-induction.

3. Pharmacodynamics parameters
   - Relationship between response (ORR) and CD22 expression levels and WBC.
   - Relationship between response (ORR) and calicheamicin sensitivity.
   - Clonal evolution (CD22-negativity) and relation to loss of response.

4. Other endpoints

- The percentage of patients responding to InO (ORR) without adequate recovery of CD19-positive B-cells and CD4+/CD8+ T-cells(below LLN for age) or immunoglobulins (below LLN for age) following 4,6,8 and 10 weeks, 3, 6 and 12 months after treatment with InO, excluding patients who have been transplanted from the date of HSCT or CAR T-cell infusion.

## Sample Size

### Stratum 1A

A Rolling-6 escalation design will be used. Escalation/de-escalation decisions will be based on the DLTs that occur during the first cycle of treatment for patients in Stratum 1A. The proposed single agent, dose escalation study stratum 1A will accrue a minimum of 6 evaluable patients. The maximum number of patients is estimated to be approximately 36 evaluable patients.

### Phase 2

The statistical design for the phase 2 cohort will consist of a single-stage design assuming binomial distribution using exact method. Assuming that an ORR of 30% or less ($H_0$) is not promising and an ORR of over 55% (Ha) is expected, a total of 25 patients evaluable for response will provide 80% power to reject $H_0$ at a significance level of 0.05 (1-sided) when the true ORR is ≥55% (Ha). The drug will be considered promising if there are ≥12 responders out of 25 response evaluable patients.

### Stratum 1B and 1B-ASP

A rolling-6 design will be used. InO will be given in combination with a modified UKALL-R3 (including dexamethasone, vincristine and asparaginase) re-induction chemotherapy, with the InO dose starting at 60% (i.e. 2 dose levels lower) of the RP2D of InO established in Stratum 1A, and which will not exceed the MTD/RP2D for single-agent InO as determined in stratum 1A. Escalation/de-escalation decisions will be based on the DLTs that occur during the first cycle of treatment for patients in Stratum 1B. The proposed dose escalation stratum 1B will accrue a minimum of 6 evaluable patients. The starting DL for the new cohort with the reduced dexamethasone dose will be DL-1, once the combination of InO at DL-2 with 20 mg/m$^2$/day of dexamethasone is considered safe. If the latter appears unsafe, dosing of patients will start from InO DL-2 with 10 mg/m$^2$/day dexamethasone. The Steering Committee will review the risk-benefit after each cohort before deciding to escalate to the next cohort. The Steering Committee could decide not to escalate to 1.8 mg/m$^2$ or to the MTD, based on the most recent risk-benefit analysis. Therefore, the maximum dose in stratum 1B/1B-ASP might be capped for example at 1.4 mg/m$^2$ in combination with chemotherapy.
Initially, asparaginase will not be given during the dose escalation phase of Stratum 1B. Safety permitting, in the latter cohort, asparaginase will be included in the re-induction combination regimen, followed by an expansion cohort to gain sufficient safety information for future studies. The part where asparaginase is added will be referred to as ‘Stratum 1B-ASP’.

The maximum number of patients in Stratum 1B/1B-ASP with the addition of the dose-escalation cohort treated with the lower dose of dexamethasone is estimated to be 34 evaluable patients for the dose-finding part (4 patients were treated at DL-1; 2 DLTs occurred). Recruitment in DL-2 is ongoing with the expectation of recruiting 6 patients. It is assumed that 2 or 3 dose levels will be evaluated in Stratum 1B (see above) after protocol amendment v4.0 with lower dexamethasone dose. When the recommended dose of InO in combination with vincristine and dexamethasone 10 mg/m$^2$ is reached, 6 patients will be treated in the 1B-ASP cohort. Once the RP2D is determined for Stratum 1B-ASP, 4 additional patients will be enrolled as an expansion cohort (10 patients to be treated in total in Stratum 1B-ASP), if the treatment is considered safe after the first 6 patients are treated. In addition, up to 4 patients who are not eligible to receive ASP may be enrolled in Stratum 1B after initiation of Stratum 1B-ASP. Non-evaluable patients will be replaced. Therefore, the maximum number of evaluable patients is expected to be 42 (34 in the dose finding part, with 4 additional patients in the expansion cohort at the RP2D of 1B-ASP and up to 4 additional patients who are not eligible to receive ASP enrolled in Stratum 1B after initiation of 1B-ASP).

### Stratum 2

Since this is a separate, exploratory cohort, patients participating in Stratum 2 (other CD22-positive hematological malignancies) will not take part in either dose-finding cohort or the Phase 2 part of the study. These patients are rare and heterogeneous and numbers preclude designing a formal dose-finding study

for this stratum. Stratum 2 may remain open for the duration of the study, and it is expected that approximately 5-25 patients may be enrolled.

**Stratum 3**

The VHR cohort is designed as an optimal Simon 2-stage design based on the exact binomial distribution. Assume that an ORR of 55% or less is not promising (based on historical data from IntReALL), ie. the null hypothesis $H_0$ is ORR $\leq$ 55%, and an ORR of ≥75% is expected with InO treatment, a total of 43 patients will provide 80% power to reject $H_0$ at a significance level of 0.05 (1-sided) when the true ORR is ≥75% (Ha).

In the first stage, 15 patients will be enrolled: if 10 patients or more have clinical response (CR, CRp or CRi), the results will appear as promising and the enrollment will continue. A total of 43 patients will be enrolled and the results will be considered promising if at least 29 patients achieve a CR, CRp or CRi.

43 evaluable patients are expected to be enrolled over a 2-year period.

## Inclusion and Exclusion Criteria

The inclusion criteria are similar for the 3 strata apart from the underlying diagnosis (stratum 1: $CD22^+$ relapsed/refractory BCP-ALL; stratum 2: other $CD22^+$ B-cell malignancies; stratum 3: VHR first relapse CD22 + BCP-ALL excluding patients transplanted in 1st CR).

In addition, the inclusion criteria are similar for Stratum 1A, 1B, the Phase 2 cohort and the VHR cohort of Stratum 3, with the exception that Down syndrome patients are excluded in stratum 1A and 1B/1B-ASP, but not in the phase 2 cohort and Stratum 3.

The eligibility criteria listed below cannot be waived.

**INCLUSION CRITERIA**

**Age (for all patients)**

- Patients must be ≥ 1 and < 18 years of age at the time of enrollment.

*Additional criteria for limited to Stratum 1A and 1B only:*

- The first 3 BCP-ALL patients on dose level 1 must be aged 6 years to less than 18 years.
- Then at least 2 additional patients must be enrolled from age 1 year to less than 6 years at the same dose level.
- After this requirement is met, subsequent dose levels may enroll patients aged 1 year to less than 18 years.
- In case 2 younger patients are not yet recruited, patients aged 6 years up to less than 18 years may continue to be enrolled at dose level 1 until a maximum of 6 patients are enrolled.

**Stratum 1A, Phase 2 and Stratum 1B/1B-ASP: Diagnosis**

Patients must have either

- First relapse of BCP-ALL post allogeneic HSCT
- Second or greater relapsed or refractory BCP-ALL
- Refractory disease, defined as newly diagnosed patients who are induction failures after at least 2 previous regimens without attainment of remission, or patients with refractory first relapse after 1 previous reinduction regimen without attainment of remission.

**AND** must meet the following criteria:

- Patients must have M2 or M3 marrow status (≥ 5% blasts by morphology)
- The malignant clone needs to be CD22 surface antigen positive (in either the bone marrow or peripheral blood) by institutional standards as determined by the local immunophenotyping laboratory.
- The first 6 patients (Stratum 1A only) must have M3 marrow status (≥ 25% blasts by morphology).

**Stratum 2: Diagnosis**

Patients must have second or greater relapsed or refractory CD22-positive B-cell malignancy including but not limited to diffuse large B-cell lymphoma (DLBCL), primary mediastinal large B-cell lymphoma (PMBCL), Burkitt lymphoma, Burkitt leukemia or B-cell precursor lymphoblastic lymphoma:

- There must be histologic verification of disease at original diagnosis or subsequent relapse.
- Patient must have evaluable or measurable disease documented by radiographic criteria or bone marrow disease present at study entry.

- The malignant cells need to be CD22 surface antigen positive (in either biopsy material, the bone marrow or peripheral blood) by institutional standards as determined by the local immunophenotyping laboratory.

**Stratum 3: Diagnosis**

- First BM or combined relapse of CD22+ VHR BCP-ALL defined as any relapse <18 months from initial diagnosis and/or cytogenetic-high risk characteristics: KTM2A/AF4, E2A/TCF3-PBX1 t(1;19) or E2A/TCF3-HLF t(17;19), hypodiploidy (less than 40 chromosomes), TP53 mutation and/or deletion, as also shown in Table 2 (excluding patients who received a HSCT in 1st CR).

  **AND** must meet the following criteria:
  - Patients must have M2 or M3 marrow status (≥ 5% blasts by morphology)
  - The malignant clone needs to be CD22 surface antigen positive (in either the bone marrow or peripheral blood) by institutional standards as determined by the local immunophenotyping laboratory.
- Evidence of prior fusion gene abnormalities is acceptable as they tend to be stable during the course of the disease.
- Laboratory techniques acceptable to test the presence of the above mentioned cytogenetic-high risk characteristics are chromosome banding analysis (CBA), FISH, PCR and/or Next Generation Sequencing (inclusion is based on local laboratory results).

**For all patients**

**Performance Level and Life Expectancy**

- Karnofsky > 60% for patients > 16 years of age and Lansky > 60% for patients ≤ 16 years of age. (See Appendix I for Performance Scales).
- Patient must have a life expectancy of at least 6 weeks.

**Prior Therapy**

Patients must have fully recovered from the acute toxic effects of all prior chemotherapy, immunotherapy, or radiotherapy defined as resolution of all such non-hematologic toxicities to ≤ Grade 2 per the CTCAE 4.03 prior to entering this study, with the exception of the authorized laboratory abnormalities as defined in the inclusion/exclusion criteria.

a. Chemotherapy:
   At least 7 days must have elapsed since the completion of cytotoxic therapy, with the exception of hydroxyurea, 6-mercaptopurine and steroids which are permitted up until 48 hours prior to initiating protocol therapy. Patients may have received intrathecal therapy at any time prior to study entry. Patients who relapse while receiving maintenance chemotherapy will not be required to have a waiting period before enrollment onto this study.

b. Radiotherapy:
   At least 28 days must have elapsed since any prior radiation therapy.

c. Hematopoietic Stem Cell Transplant:
   At least 90 days must have elapsed since previous allo-HSCT. Patient must have no evidence of active graft vs. host disease. Patient must not be receiving GVHD prophylaxis or treatment.

d. Hematopoietic growth factors:
   At least 7 days must have elapsed since the completion of therapy with GCSF or other growth factors at the time of enrollment. At least 14 days must have elapsed since the completion of therapy with pegfilgrastim (Neulasta®).

e. Immunotherapy:
At least 42 days must have elapsed after the completion of any type of immunotherapy, e.g. chimeric antigen receptor T cell (CART) therapy. Patients may not have received prior CD22-targeted therapy (immunotoxin or CART therapy).

f. Monoclonal antibodies:
At least 3 half-lives of the antibody must have elapsed after the last dose of a monoclonal antibody (ie: Rituximab = 66 days, Epratuzumab = 69 days), with the exclusion of blinatumomab. Patients must have been off blinatumomab infusion for at least 14 days and all drug-related toxicity must have resolved to grade 2 or lower as outlined in the inclusion and exclusion criteria.

g. Investigational drugs:
At least 7 days or 5 drug half-lives (whichever is longer) must have elapsed since prior treatment with any experimental drug (with the exception of monoclonal antibodies) under investigation. No residual toxicities should be observed following previous treatment. An experimental drug is defined as any drug that is not approved and licensed for sale by the FDA for institutions in the United States, by the EMA for institutions in Europe, by Health Canada for institutions in Canada and by The Therapeutic Goods Administration for institutions in Australia.

h. Prior calicheamicin exposure:
Patient has not received prior treatment with a calicheamicin-conjugated antibody (e.g. gemtuzumab ozogamicin).

**Renal and Hepatic Function**

- Patient's serum creatinine must be ≤ 1.5 x institutional upper limit of normal (ULN) according to age. If the serum creatinine is greater than 1.5 x institutional ULN, the patient must have a GFR ≥ 70mL/min/1.73 $m^2$ estimated based on serum creatinine and/or cystatin C levels (e.g. Bedside Schwartz formula).

- Patient's AST and ALT must be ≤ 2.5 x institutional ULN.

- Patient's total total bilirubin must be ≤ 1.5 x institutional ULN unless the patient has documented Gilbert syndrome, in which case the AST and ALT must be ≤ 2.5 x ULN.

**Cardiac Function**

- Patient must have a shortening fraction ≥ 30% by echocardiogram or an ejection fraction > 50% by MUGA.

**Reproductive Function**

- Female patients of childbearing potential must have a negative urine or serum pregnancy test confirmed prior to enrollment.
- Female patients with infants must agree not to breastfeed their infants while on this study.
- Male and female patients of child-bearing potential must agree to use a *highly effective* method of contraception approved by the investigator during the study, following the CTFG recommendations, and for at least 8 months for females and for at least 5 months for males after the last dose of InO.
- Highly effective methods of contraception include (but not exclusively) the following contraceptive methods:
  - combined (estrogen- and progestogen-containing) hormonal contraception associated with inhibition of ovulation
  - progestogen-only hormonal contraception associated with inhibition of ovulation
  - intrauterine device (IUD)
  - intrauterine hormone-releasing system (IUS)
  - sexual abstinence.

**EXCLUSION ELIGIBILITY CRITERIA**

Patients will be excluded if they meet any of the following criteria:

**Isolated extramedullary relapse**

- Patients with isolated extramedullary disease are excluded (not applicable to lymphoma patients except for isolated CNS-relapse)

**VOD/SOS**

- Patients with any history of prior or ongoing VOD/SOS per the modified Seattle criteria are excluded, as specified in Appendix 3, or prior liver-failure [defined as severe acute liver injury with encephalopathy and impaired synthetic function (INR of ≥1.5)].

**Infection**

- Patients will be excluded if they have a systemic fungal, bacterial, viral or other infection that is exhibiting ongoing signs/symptoms related to the infection without improvement despite appropriate antibiotics or other treatment.
- The patient may not have:
  - A requirement for vasopressors;
  - Positive blood culture within 48 hours of study enrollment;
  - Fever above 38.2 degrees Celsius within 48 hours of study enrollment with clinical signs of infection. Fever that is determined to be due to tumor burden is allowed if patients have documented negative blood cultures for at least 48 hours prior to enrollment and no concurrent signs or symptoms of active infection or hemodynamic instability.
  - A positive fungal culture within 30 days of study enrollment.
  - Active fungal, viral, bacterial, or protozoal infection requiring IV or oral treatment. Chronic prophylaxis therapy to prevent infections is allowed.

**Other anti-cancer therapy**

- Patients will be excluded if there is a plan to administer non-protocol anti-cancer therapy including but not limited to chemotherapy, radiation therapy, or immunotherapy during the study period.
- Patients will be excluded if they have received prior treatment with anti-tumor vaccines.

**Allergic reaction**

- Patients with prior Grade 3/4 allergic reaction to a monoclonal antibody are excluded.

**Concurrent disease**

- Patients will be excluded if they have significant concurrent disease, illness, psychiatric disorder or social issue that would compromise patient safety or compliance with protocol therapy, interfere with consent, study participation, follow up, or interpretation of study results.
- Children with Down syndrome are excluded from participation in the dose finding parts (stratum 1A and 1B), but not in the single-agent phase 2 cohort or the VHR cohort.

**Additional exclusion criteria for Stratum 1B**

- Patients with grade 3-4 peripheral neuropathy (as defined in the Delphi consensus of acute toxic effects for childhood ALL by Schmiegelow et al.[12]).
- Patients with prior history of thrombosis during steroid and/or asparaginase are eligible provided they use adequate anti-coagulant prophylaxis, according to institutional guidelines.
- Patients in whom prior experience suggests that a timely delivery of therapy is unlikely or associated with an undue risk because of intolerance.

**Additional exclusion criteria for Stratum 1B-ASP cohort only**

- Patients with any history of PEG-asparaginase intolerance due to allergic reactions or silent inactivation during prior treatment.

- Patients with any history of prior asparaginase-associated acute pancreatitis (any grade as defined in the Delphi consensus[12].

Patients who are excluded from Stratum 1B-ASP may potentially be enrolled in Stratum 1B expansion cohort.

**Additional exclusion criteria for Stratum 3 (VHR cohort) only**

- Patients who are transplanted in CR1 (such patients are eligible for the phase 1B cohort).

**TABLE OF CONTENTS**

**SPONSOR** ..... **2**
**TRIAL STEERING COMMITTEE** ..... **2**
**INTERNATIONAL COORDINATING INVESTIGATOR** ..... **2**
**OTHER MEMBERS OF THE TRIAL STEERING COMMITTEE** ..... **2**
**STUDY STATISTICIAN** ..... **3**
**PHARMACOKINETICS** ..... **3**
**LABORATORIES** ..... **4**
**SIGNATURE PAGE** ..... **6**
**INVESTIGATOR DECLARATION AND SIGNATURE** ..... **6**
**AMENDMENT HISTORY** ..... **7**
**AMENDMENT VERSION 4** ..... **10**
Ad1: Modification of dexamethasone dose ..... 10
Ad2: Inclusion of a "Very High-risk" cohort as Stratum 3 ..... 13
**AMENDMENT VERSION 3** ..... **16**
**AMENDMENT VERSION 2** ..... **17**
**ABBREVIATIONS** ..... **21**
**TRIAL SYNOPSIS** ..... **23**
**TABLE OF CONTENTS** ..... **41**
**LIST OF TABLES** ..... **44**
**LIST OF FIGURES** ..... **46**
**PROTOCOL** ..... **47**
**1.0 AIMS AND OBJECTIVES** ..... **47**
1.1 Disease strata and cohorts ..... 47
1.2 Primary Objectives ..... 47
1.3 Secondary Objectives ..... 48
**2.0 BACKGROUND AND RATIONALE** ..... **51**
2.1 Current Results in Pediatric Acute Lymphoblastic Leukemia (ALL) ..... 51
2.2 CD22 and its expression in childhood ALL ..... 58
2.3 Other CD22 positive B-cell malignancies ..... 58
2.4 Inotuzumab Ozogamicin ..... 59
2.5 Clinical trials of Inotuzumab Ozogamicin ..... 61
2.6 Biomarker and PK studies ..... 65
2.7 Rationale for this study ..... 66
**3.0 PATIENT SCREENING, ELIGIBILITY AND ENROLLMENT** ..... **68**
3.1 Informed Consent ..... 68
3.2 Reservation and Enrollment ..... 68
3.3 Diagnostic work-up ..... 71
3.4 Eligibility criteria ..... 71
**4.0 TREATMENT PROGRAM** ..... **76**
4.1 Treatment program for Stratum 1A, the Phase 2 Cohort, Stratum 2 and Stratum 3 patients ..... 76
4.2 Treatment program for Stratum 1B and 1B-ASP ..... 77
4.3 Treatment Cycles ..... 78
4.4 Dose discontinuation/delay/modification ..... 86
4.5 Dose Escalation Schedule ..... 91
4.6 Dose-limiting Toxicity (DLT) ..... 93
**5.0 SUPPORTIVE CARE** ..... **96**
5.1 Blood Products ..... 96
5.2 Infection Control and Prophylaxis ..... 96
5.3 Anti-emetic Protection ..... 96
5.4 Tumor Lysis ..... 97
5.5 Concurrent Therapy ..... 97
5.6 Hepatic Toxicity ..... 97
**6.0 DRUG INFORMATION** ..... **101**

6.1 Inotuzumab Ozogamicin ........ 101
6.2 Methotrexate (MTX) ........ 105
6.3 Cytarabine (Ara-C) ........ 106
6.4 Prednisolone for IT use ........ 107
6.5 Hydrocortisone for IT use ........ 107
6.6 Dexamethasone for systemic administration ........ 108
6.7 Vincristine ........ 109
6.8 Pegylated (PEG-) asparaginase ........ 110
**7.0 REQUIRED OBSERVATIONS/MATERIAL AND DATA TO BE ACCESSIONED ........ 112**
7.1 Schedule of events for BCP-ALL patients in Stratum 1A and Phase 2 cohort ........ 112
7.2 Schedule of events for BCP-ALL in Stratum 1B/1B-ASP patients – combination therapy cycle 1 and 2 ........ 116
*7.3 Schedule of Events for Stratum 1B/1B-ASP, cycle 2 and/or beyond – for patients continuing therapy with single-agent InO.* ........ 119
7.4 Schedule of events for BCP-ALL patients in Stratum 3 ........ 121
7.5 Schedule of Events for Stratum 2 patients with other CD22-positive B-cell malignancies. ........ 125
7.6 Samples for Minimal Residual Disease (MRD) testing (BCP-ALL patients only) ........ 129
7.7 Biology samples: CD22 Expression and Saturation Kinetics ........ 130
7.8 Pharmacokinetic Samples (Stratum 3 patients excluded) ........ 132
7.9 Immunogenicity (for patients treated with single-agent InO only) ........ 135
7.10 In-vitro Calicheamicin Resistance ........ 136
7.11 End of Treatment and Week 10 post last InO dose safety visit ........ 136
7.12 Follow-up for all patients ........ 137
**8.0 CRITERIA FOR REMOVAL FROM PROTOCOL THERAPY, OFF STUDY CRITERIA AND STUDY TERMINATION ........ 140**
8.1 Criteria for removal of subjects from protocol therapy (End of treatment) ........ 140
8.2 Off study criteria (End of study) ........ 140
8.3 Termination of the Study by ITCC ........ 141
**9.0 STATISTICAL CONSIDERATIONS ........ 142**
9.1 Stratum 1A ........ 142
9.2 Phase 2 cohort ........ 143
9.3 Stratum 1B and 1B-ASP ........ 144
9.4 Stratum 2 ........ 145
9.5 Stratum 3 ........ 146
9.6 Statistical design for dose escalation phase (Stratum 1A) ........ 147
9.7 Statistical design for dose escalation phase (Stratum 1B/1B-ASP) ........ 148
9.8 Statistical design for the Phase 2 cohort ........ 149
9.9 Statistical design for Stratum 2 ........ 150
9.10 Statistical design for Stratum 3 ........ 150
9.11 Patient Accrual and Study Duration ........ 150
9.12 Interim Monitoring of Toxic Death ........ 151
9.13 Analysis of primary endpoints ........ 151
9.14 Analysis of secondary toxicity, efficacy and correlative endpoints. ........ 152
9.15 Definitions ........ 152
**10.0 ASSESSMENTS ........ 153**
10.1 Safety Assessments ........ 153
10.2 Pregnancy Testing and Contraception Check ........ 153
10.3 Adverse Events ........ 153
10.4 Laboratory Safety Assessments ........ 153
10.5 Vital signs and Physical Examination ........ 154
10.6 Echocardiograms ........ 154
10.7 Menarchal Status Assessments ........ 154
10.8 Disease Response Assessments ........ 154
**11.0 RESPONSE CRITERIA ........ 155**
11.1 Response criteria in BCP-ALL patients ........ 155
11.2 Response criteria in patients with other CD22-positive malignancies ........ 155

**12.0 ADVERSE EVENTS ..... 156**
12.1 Definition of an Adverse Event ..... 156
12.2 Definition of Serious Adverse Events ..... 156
12.3 Adverse Event Reporting ..... 157
12.4 Serious Adverse Event Reporting ..... 157
12.5 AEs of Special Interest ..... 158
12.6 Sponsor Reporting Requirements to Regulatory Authorities ..... 159
12.7 Reporting to Pfizer ..... 159
12.8 Safety Oversight ..... 159
12.9 SUSARs ..... 159
12.10 Development Safety Update Reports ..... 160
**13.0 DATA HANDLING AND RECORD KEEPING ..... 161**
13.1 Case Report Forms / Electronic Data Record ..... 161
13.2 Record retention / Archiving ..... 161
**14.0 QUALITY MANAGEMENT ..... 162**
14.1 Site Set-up and Initiation ..... 162
14.2 On-site Monitoring ..... 162
14.3 Central Monitoring ..... 162
14.4 Audit and Inspection ..... 163
14.5 Notification of Serious Breaches (deviations/violations) ..... 163
**15.0 ETHICAL CONSIDERATIONS ..... 164**
15.1 Institutional Review Board (IRB)/Ethics Committee (EC) ..... 164
15.2 Patient Information Sheet and Consent Form ..... 164
15.3 Patient Recruitment ..... 164
15.4 Reporting of Safety Issues and Serious Breaches of the Protocol or ICH GCP ..... 164
15.5 Sponsor discontinuation criteria ..... 164
15.6 Risk-benefit analysis ..... 165
**16.0 CONFIDENTIALITY AND DATA PROTECTION ..... 166**
**17.0 INSURANCE AND INDEMNITY ..... 166**
**18.0 PUBLICATION POLICY ..... 166**
**19.0 REFERENCES ..... 167**
**APPENDIX 1: PERFORMANCE STATUS SCALES / SCORES ..... 173**
**APPENDIX 2: RESPONSE CRITERIA ..... 174**
**APPENDIX 2.1. Definitions and Response Criteria for BCP-ALL patients** ..... 174
**APPENDIX 2.2 Response criteria for pediatric NHL patients [112] ..... 176**
**APPENDIX 3: MODIFIED SEATTLE CRITERIA FOR THE DIAGNOSIS OF VOD/SOS ..... 180**
**APPENDIX 4: FORMULAE ..... 182**
**APPENDIX 5: REINDUCTION UKALL-R3 THERAPY COMBINED WITH INO ..... 183**
**APPENDIX 6: DRUG ADMINISTRATION SETUP AND PROCESS FOR LOW VOLUME DOSES ..... 184**

**LIST OF TABLES**

*Table 1. Hepatic Transaminases Trend in Patient 66-31. Alanine aminotransferase (AST) and aspartate aminotransferase (AST)* ........ 11
*Table 2. Previous classification from IntReALL 2010 and revised risk-group stratification schema from IntReALL 2020 for BCP-ALL* ........ 13
**Table 3***: Toxicities during cycle 1, including transaminase elevations, in 51 children treated with InO on compassionate use basis, reported by Bhojwani et al, ASCO 2017.* ........ 17
*Table 4. Liver functions of patient 10-20. Liver functions increased after one infusion with InO. There were no liver function abnormalities present at screening.* ........ 18
*Table 5. Bilirubin values in patient 12-06. Only 1 dose of InO was infused.* ........ 19
*Table 6. Liver functions in patient 15-04 in relation to the 3 InO infusions of the first cycle* ........ 20
*Table 7: Outcomes of 1st relapses, BM isolated & BM combined, by genetic mutation* ........ 54
*Table 8. Incidence of NHL in pediatric population* ........ 58
*Table 9. Single-agent InO schedule for cycle 1* ........ 78
*Table 10. Stratum 1B/1B-ASP combination InO schedule for cycle 1 (See Appendix 5)* ........ 79
*Table 11. Dosages of IT medication for patients with CNS1 status* ........ 80
*Table 12: Dosages of IT medication for CNS 2 or 3 status* ........ 80
*Table 13. InO schedule for cycle 2-6 of Single Agent InO* ........ 84
*Table 14. Dose Modification/Reduction for Toxicity in Stratum 1B/1B-ASP* ........ 88
*Table 15. InO dose-levels for Stratum 1A and 1B Patients (Note that Dose Level 2 is the RP2D for InO single agent only and to be used in Stratum 3)* ........ 91
*Table 16. Side-effects related to single agent InO in other studies* ........ 101
*Table 17. Toxicities of intrathecal methotrexate* ........ 105
*Table 18. Toxicities of intrathecal cytarabine* ........ 106
*Table 19. Toxicities for systemically administered dexamethasone* ........ 108
*Table 20. Toxicities of Vincristine* ........ 109
*Table 21. Toxicities of PEG-asparaginase* ........ 111
**Table 22** *Schedule of events for BCP-ALL patients in Stratum 1A and Phase 2 cohort. Further follow-up is described in Table 33* ........ 112
*Table 23. Schedule of events for BCP-ALL in Stratum 1B/1B-ASP patients – combination therapy cycle 1* ........ 116
*Table 24: Schedule of Events for Stratum 1B/1B-ASP, cycle 2 and/or beyond for 1B/1B-ASP patients continuing therapy with single-agent InO.* ........ 119
**Table 25.** *Schedule of events for BCP-ALL patients in Stratum 3 (VHR). Further follow-up is described in Table 33 and Table 34.* ........ 121
*Table 26: Schedule of Events for Stratum 2 patients with other CD22-positive B-cell malignancies.* ........ 125

***Table 27: Instructions to perform MRD monitoring in BCP-ALL patients*** ........ 129
***Table 28: Instructions to perform CD22 saturation analysis*** ........ 131
***Table 29. Cycle 1. Sampling schedule for PK of InO*** ........ 133
***Table 30. Cycle 2. Sampling schedule for PK of InO*** ........ 134
***Table 31. Cycle 3. Sampling schedule for PK of InO*** ........ 134
***Table 32. In-vitro Calicheamicin Resistance*** ........ 136
***Table 33. Follow up for all patients except patients in Stratum 3 (see Table 34 )*** ........ 138
***Table 34 Specific follow up for patients in Stratum 3*** ........ 139

**LIST OF FIGURES**

**Figure 1: EFS probability for risk group S1-4 within the ALL-REZ BFM 2002** .................................... 52
**Figure 2: Kaplan Meier estimates of progression-free survival (A) and overall (B) survival by randomized treatment 3-year estimated survival percentages within the UKALL-R3.[29]** .................. 53
**Figure 3. Disease-free Survival and Overall Survival of Blinatumomab (Arm B) Vs. Chemotherapy (Arm A) As Post-Reinduction Therapy in High and Intermediate Risk (HR/IR) First Relapse of B-Acute Lymphoblastic Leukemia (B-ALL) in Children and Adolescents/Young Adults (AYAs).[11]** ..... 54
***Figure 4. Combined analysis of HR relapses ALL R3/ALL-REZ BFM 2002. Event Free Survival at 10 years (EFS) in early and very early relapse showing the VHR of induction failure after very early relapse (37%) represented by the blue line (Courtesy Dr. A. Von Stackelberg).*** ............................... 55
***Figure 5: Cumulative incidence of disease (progression, relapse and disease related death) and treatment related events (treatment-related deaths and 2nd malignancies) by induction in UKALL-R3.[29]*** ........................................................................................................ 56
***Figure 6. NHL subtypes in pediatric and adult patients[51]*** .................................................................... 59

**PROTOCOL**

## 1.0 AIMS AND OBJECTIVES

### 1.1 Disease strata and cohorts

**This protocol contains 3 disease strata:**

- **Stratum 1**: relapsed (2nd or greater relapse or first relapse after transplant)/refractory (R/R) CD22 positive B-cell precursor (BCP)-ALL

  In stratum 1, three different cohorts of patients are planned:

  - **Stratum 1A**: in this cohort we will determine the maximum tolerated dose (MTD) or the Recommended Phase 2 Dose (RP2D) of single-agent InO using a Rolling-6 design

  - **Phase 2 Cohort**: in this cohort we will assess the preliminary activity [overall response rate, (ORR) defined as the rate of patients who achieve complete remission (CR), CR with incomplete hematologic recovery (CRi), or CR with incomplete platelet recovery (CRp)] after single-agent InO using a single-stage design

  - **Stratum 1B**: in this cohort we will determine the RP2D of InO in combination with a modified version of UKALL-R3 re-induction chemotherapy, using a Rolling-6 design.
    - Note that the final cohort, in which pegylated-asparaginase (PEG-ASP) is added, is referred to as Stratum **1B-ASP**

- **Stratum 2**: other CD22 positive B-cell malignancies

- **Stratum 3:** VHR first relapse CD22-positive BCP-ALL patients (defined as isolated bone marrow or combined relapse < 18 months from initial diagnosis, and/or cytogenetic-high risk characteristics: KTM2A/AF4, E2A/TCF3-PBX1 t(1;19) or E2A/TCF3-HLF t(17;19), hypodiploidy (less than 40 chromosomes), TP53 mutation and/or deletion) excluding patients transplanted in 1st CR. In this cohort we will assess the preliminary activity (ORR defined as CR, CRi, CRp) in VHR first relapse ALL, after single-agent InO (at the RP2D single agent dose established by Stratum 1a/Phase 2 cohort), using a Simon 2-stage design

The formal dose-finding part of this protocol will be limited to stratum 1A for single-agent InO. Data derived from stratum 2 will be explorative, and patients can enroll when they present and always at least one dose level below the current dose-escalation level in stratum 1A, hence at a dose that has provisionally been determined to be safe. The dose that will be taken forward in the Phase 2 cohort will be determined by the Steering Committee considering the safety and efficacy data generated in the dose-finding stratum 1A. The dose-finding part will be performed in Stratum 1B for the combination treatment.

## Objectives

### 1.2 Primary Objectives

Stratum 1

- Stratum 1A:
  To establish the MTD or the RP2D of single agent InO when administered in children with CD22-positive relapsed/refractory BCP-ALL.
  Note that:

  - if the MTD is not reached at the highest tested dose, no further dose-escalation will be performed.
  - to establish the RP2D for further study in the Phase 2 cohort, additional modelling will be performed regarding cumulative toxicity (as described in Section 9)

- Phase 2 Cohort:
  To establish the activity (ORR defined as the rate of patients with CR, CRi and CRp) of single agent InO when administered in children with CD22-positive relapsed/refractory BCP-ALL.

- Stratum 1B (and 1B-ASP):
  To determine the RP2D of InO in children with CD22-positive relapsed/refractory BCP-ALL in combination with a modified UKALL-R3 based re-induction regimen, initially without ASP.

Stratum 2 (EXPLORATORY)

- To explore the safety and tolerability of InO as a single agent in children with relapsed/refractory other CD22 positive B-cell malignancies.

Stratum 3

- To establish the preliminary clinical activity (efficacy) with respect to ORR of single agent InO when administered in children with CD22-positive VHR 1st relapse BCP-ALL (excluding patients transplanted in 1$^{st}$ CR).

### 1.3 Secondary Objectives

Stratum 1A and Phase 2 Cohort (BCP- ALL patients)

- To determine the safety and tolerability of InO as a single agent during cycle 1; the cumulative toxicities in patients receiving multiple cycles of InO; as well as after subsequent allogeneic hematopoietic stem cell transplant (allo-HSCT).
- To determine the overall hematological response rate in these patients:
  - after cycle 1
  - as well as the overall best response (Stratum 1A only; this is the primary objective for the Phase 2 cohort).
- To determine minimal residual disease (MRD) levels in responding patients, including the percentage of patients with a complete MRD response (see Appendix 2.1)
  - after cycle 1
  - as well as the best overall response.
- To describe the durability of response and long-term follow-up, including the number of patients that undergo HSCT or CAR-T cells therapy after treatment with InO as consolidation, the cumulative incidence of non-response or relapse, the cumulative incidence of non-relapse mortality, the EFS and overall survival (OS).
- To determine the serum pharmacokinetic parameters of unconjugated calicheamicin and InO in the pediatric population.

- To assess the relationship between CD22 receptor density, white blood cell count (WBC) at start of treatment, CD22 saturation kinetics, cytogenetics, and in-vitro calicheamicin resistance to clinical response to InO.
- To assess for the persistence of B-Cell aplasia and hypogammaglobulinemia in responding patients following treatment with InO.
- To assess the number of patients developing anti-drug antibodies (ADAs; immunogenicity).

<u>Stratum 1B and Stratum 1B-ASP (BCP ALL patients)</u>

- To determine the safety and tolerability of InO in combination with a modified UKALL-R3 re-induction chemotherapy regimen; and the cumulative toxicities in patients receiving multiple InO-based cycles, as well as after subsequent allogeneic HSCT.
- To determine the overall hematological response rate:
  - after cycle 1
  - as well as the overall best response
- To determine MRD levels in responding patients, including the percentage of patients with a complete MRD response (see Appendix 2.1 for definition):
  - after cycle 1
  - as well as the best overall response.
- To describe the durability of response and long-term follow-up, including the number of patients that undergo HSCT or CAR-T cells therapy after study treatment as consolidation, the cumulative incidence of non-response or relapse, the cumulative incidence of non-relapse mortality, the EFS and OS.
- To determine the serum pharmacokinetic parameters of unconjugated calicheamicin and InO when added to a modified reinduction UKALL-R3 chemotherapy regimen initially without followed by with ASP.
- To assess the relationship between CD22 expression and clinical response to InO
- To assess the number of patients developing CD22-negative relapse

<u>Stratum 2 (Other B-cell malignancies)</u>

- To determine the response rate in these patients:
  - after cycle 1
  - as well as the overall best response.
- To describe the durability of response and long-term follow-up, including the number of patients that undergo stem-cell transplant after treatment with InO, the cumulative incidence of non-response or relapse, the cumulative incidence of non-relapse mortality, the EFS and OS.
- To determine the serum pharmacokinetic parameters of unconjugated calicheamicin and InO in the pediatric population.
- To assess for the persistence of B-Cell aplasia and hypogammaglobulinemia in responding patients following treatment with InO.
- To assess the number of patients developing ADAs (NOT valid for patients enrolled after Amendment 4).

<u>Stratum 3 (VHR)</u>

- To determine the safety and tolerability of InO as a single agent during cycle 1; the cumulative toxicities in patients receiving multiple cycles of InO; as well as after subsequent allogeneic hematopoietic stem cell transplant (allo-HSCT) or CAR-T cells therapy.
- To determine the overall hematological response rate in these patients after cycle 1.
- To determine minimal residual disease (MRD) levels in responding patients, including the percentage of patients with a complete MRD response (see Appendix 2.1)
    - after cycle 1
    - as well as the best overall response.
- To describe the durability of response and long-term follow-up, including the number of patients that undergo HSCT or CAR-T cells therapy after treatment with InO as consolidation, the cumulative incidence of non-response or relapse, the cumulative incidence of non-relapse mortality, the EFS and overall survival (OS).
- To assess the relationship between CD22 receptor density, white blood cell count (WBC) at start of treatment, cytogenetics, and in-vitro calicheamicin resistance to clinical response to InO.
- To assess the persistence of B-Cell aplasia and MRD negativity in responding patients following the treatment with InO and the implications for CAR-T cells therapy.

# 2.0 BACKGROUND AND RATIONALE

## 2.1 Current Results in Pediatric Acute Lymphoblastic Leukemia (ALL)

Acute lymphoblastic leukemia (ALL) is the most frequent malignant disease in childhood with an incidence of about 4/100,000 children per year. In the past two decades significant improvement in outcome has been made in the treatment of childhood ALL, with current rates of EFS in first complete remission (CR) of 80-90% with the use of multi-drug front-line protocols.[14-16] However, the prognosis for those children who experience a relapse or who are refractory to front-line therapy (about 20%) remains poor. Relapse is the main cause of treatment failure and death for these patients.[17-20]

Following a relapse, only about 40% of children can be successfully salvaged with intensive chemotherapy treatment, followed by allo-HSCT in high-risk cases of early ALL relapse or late bone marrow relapse with persistence of MRD.

The common therapeutic elements of current successful strategies for salvage treatment following ALL relapse are induction of a second complete remission (CR2) with conventional intensive reinduction chemotherapy, followed by consolidation, re-intensification and maintenance therapy; for eligible patients, allo-HSCT or chimeric-antigen receptor T-cell therapy (CAR T) may be undertaken as further intensification of treatment.[21] Availability of the latter is still very limited in Europe to date.

Repeated intensive short treatment cycles have been successfully utilized for relapsed ALL by the Italian Study Group (AIEOP), the German/Austrian Berlin-Frankfurt-Münster Study Group (BFM), the French/Belgium/Luxembourg Cooperative Group (COPRALL), the Children's Oncology Group (COG) and the United-Kingdom ALL Relapse Study Group (MRC UKALL-R).[19,22-24]

Another common feature is risk group stratification based on prognostic factors such as time and site of ALL relapse, immunophenotype of leukemic blasts, and MRD response during early phases of treatment, in order to facilitate risk-adapted treatments, whereby children expected to have an acceptable EFS rate without allo-HSCT are treated with chemotherapy alone and those with high-risk factors undergo allo-HSCT in order to achieve an acceptable cure rate.[20,25-27] More recently, the IntReALL-consortium identified a subgroup of patients defined as "very high-risk". Patients relapsing earlier than 18 months from the diagnosis and/or presenting specific genetic mutations (KTM2A/AF4, E2A/TCF3-PBX1 t(1;19) or E2A/TCF3-HLF t(17;19), hypodiploidy (less than 40 chromosomes), TP53 mutation and/or deletion) account for the VHR group of relapsed ALL patients, and have a particularly poor prognosis, with EFS at 10 years of approximately 20%.[5-9]

**The ALL-REZ BFM 2002 Trial for 1$^{st}$ relapse of ALL**

The ALL relapse trial completed by the BFM Group was the ALL-REZ BFM 2002 protocol, which was based on preceding BFM ALL relapse trials which used a risk-adapted treatment approach of chemotherapy with or without allo-HSCT by stratifying patients based on the duration of initial response, immunophenotype, and involved site(s) at relapse. Since 1995, the ALL-REZ BFM group has used the S1-4 (defined below) stratification of patients to treat relapsed ALL patients with a risk-adapted strategy. Treatment consisted of induction cycles, followed by consolidation cycles and irradiation/maintenance therapy or consolidation by allo-HSCT, depending on the patient's risk profile and MRD response after the initial phases of treatment. The results of this experience showed a significant improvement of event-free-survival (EFS) and overall survival (OS) compared to preceding trials, particularly in patients in the intermediate risk group, even with poor MRD. The improvement in survival can be attributed particularly to the improved selection of patients eligible for HSCT and to the standardization of transplant procedure and management, leading to lower rates of treatment-related mortality. S1 patients (late isolated extramedullary relapse) had acceptable EFS rates with chemo/radiotherapy of 70%, whereas S3 (early isolated BM relapse of BCP immunophenotype) and S4 (very early bone marrow relapse and any T-lineage bone marrow relapse) patients achieved EFS rates below 5% with chemotherapy alone and were assigned to allo-HSCT since then. The large and heterogeneous S2 groups (very early/early isolated extramedullary relapse, early/late combined bone marrow and late isolated bone marrow relapse of BCP immunophenotype) achieved intermediate EFS rates of about 60%.[19,28] See **Figure 1**.

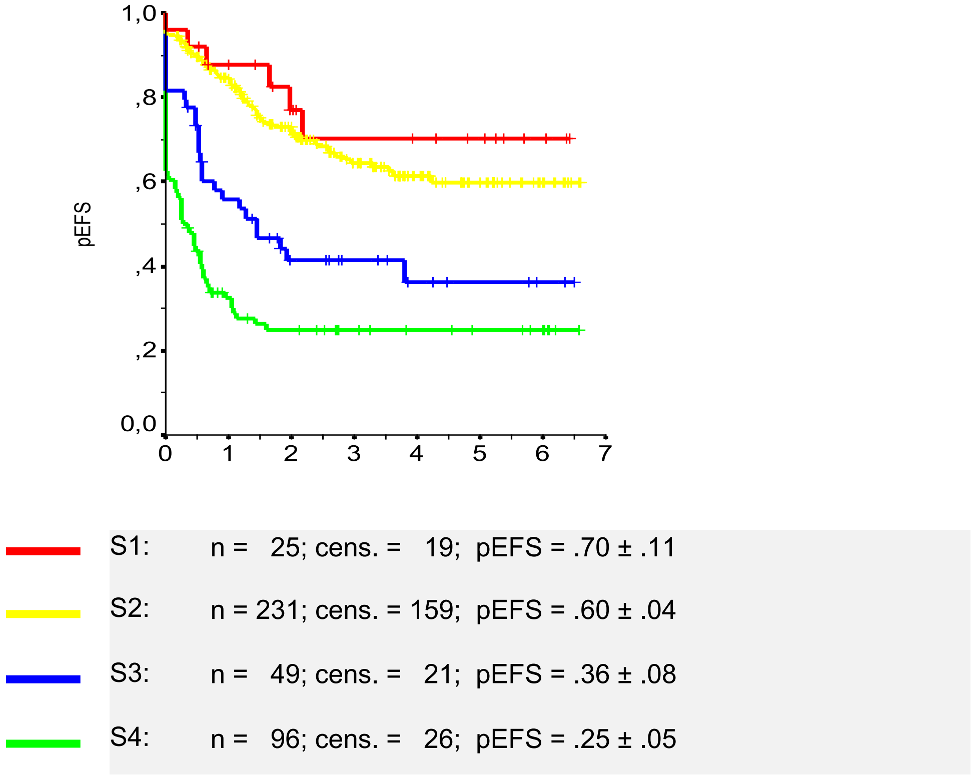


**Figure 1: EFS probability for risk group S1-4 within the ALL-REZ BFM 2002**

**The UKALL-R3 Trial for 1st relapse of ALL**

The design of the UKALL-R3 trial for ALL in 1st relapse was based on the experience of preceding ALL relapse trials such as the UKALL R1 and R2 protocols[22]. The treatment strategy consisted of a continuous schedule of repetitive chemotherapy elements, avoiding longer treatment-free intervals compared to the BFM strategy, and adopting a patient risk stratification system similar to the BFM. The MRC UKALL R group used a modified version of BFM-stratification, combining S1 and S2 patients into a standard risk group and S3 and S4 patients into a high risk group. Very early isolated extramedullary ALL relapses were also included in the high risk group. In intermediate risk patients with bone marrow relapse, MRD response post-induction was used to allocate patients to allo-HSCT or chemotherapy. High risk patients received the same induction/consolidation chemotherapy but all were eligible for allo-HSCT. The main question of UKALL-R3 was to evaluate the benefit of replacing idarubicin in a randomized manner with mitoxantrone during reinduction therapy. The mitoxantrone arm showed a significantly superior EFS, leading to early cessation of the trial[29](**Figure 2**). In a subgroup analysis, the improved outcome in the mitoxantrone arm was determined to be restricted to standard risk and intermediate risk patients, and to patients undergoing HSCT[29]. The UKALL-R3 trial showed the best overall outcomes to date for standard and intermediate risk patients and is regarded by many as the current best standard of care for relapsed ALL protocols. In contrast, the results in HR patients remained unsatisfactory, with poor remission and EFS rates, leading to a clear need to improve outcomes in this subgroup of children.

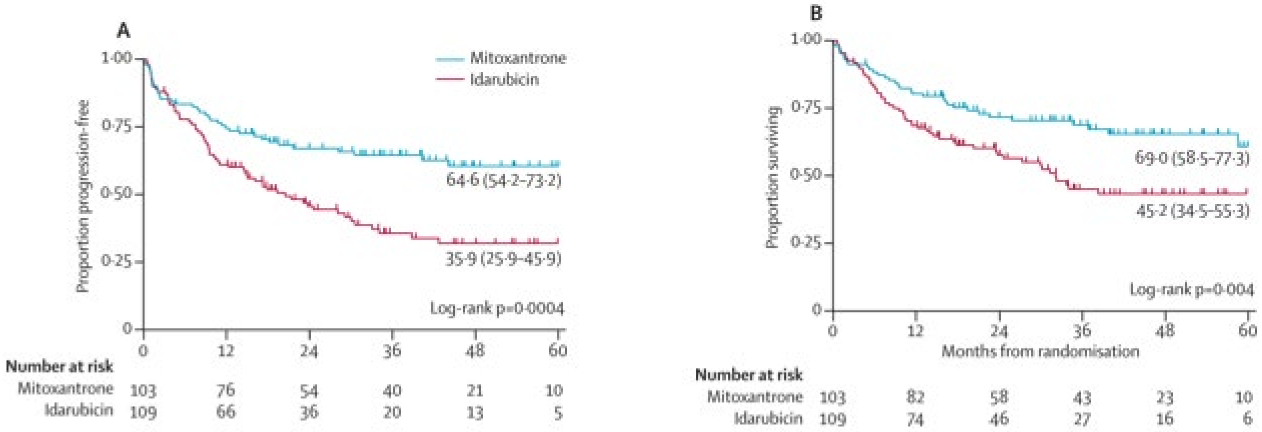


**Figure 2: Kaplan Meier estimates of progression-free survival (A) and overall (B) survival by randomized treatment 3-year estimated survival percentages within the UKALL-R3.[29]**

**The Children's Oncology Group Study AALL1331 for First Relapse of B-Acute Lymphoblastic Leukemia in Children and Adolescents/Young Adults**

A recent randomized trial compared the efficacy of blinatumomab in consolidation with UKALLR3 re-induction protocol. 208 patients, aged 1 to 30 years, were randomized to two arms after undergoing the first block of the UKALLR3 re-induction protocol. Arm A received the second and third block of the UKALLR3 re-induction protocol; Arm B received blinatumomab. After the first block of chemotherapy, patients were reassessed and risk stratified as "high risk" if early relapses and "low risk" if late relapses. The latter group was further divided into "true low risk" (with MRD below 0.1%) and "intermediate risk" (MRD at or above 0.1%). Patients with 25% or more marrow blasts and/or failure to clear extramedullary disease after the UKALL R3 block were defined as "treatment failure". Preliminary data show that after a median follow-up of 1.4 years, the intention to treat 2-year OS was 59.2% ± 6.0% (standard error) for patients treated with chemotherapy versus 79.4% ± 4.5% (standard error) for patients treated with blinatumomab; Grade 3 to 4 febrile neutropenia, infection, sepsis and mucositis were higher in patients receiving the second and third block of UKALLR3/mitoxantrone regimen, and post-induction deaths occurred in 4 patients on intensive chemotherapy versus none on blinatumomab. Notably, only 45% of patients that completed the UKALLR3 regimen proceeded to HSCT, while 73% proceeded to HSCT among those treated with blinatumomab. MRD negativity was reached by 79% patients after the first cycle of blinatumomab, while only 21% was MRD negative after the second block of UKALLR3. Combining the EFS rate and OS (**Figure 3**), treatment with blinatumomab was considered superior to UKALLR3 and with a better tolerability.

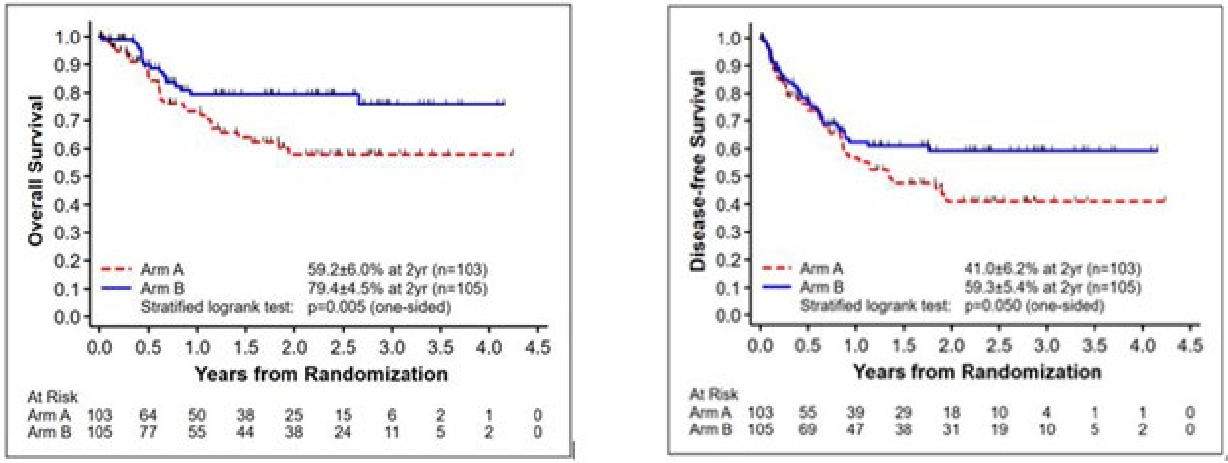


**Figure 3. Disease-free Survival and Overall Survival of Blinatumomab (Arm B) Vs. Chemotherapy (Arm A) As Post-Reinduction Therapy in High and Intermediate Risk (HR/IR) First Relapse of B-Acute Lymphoblastic Leukemia (B-ALL) in Children and Adolescents/Young Adults (AYAs).[11]**

**VHR Relapse B cell ALL**

Patients with very early relapse (<18 months after diagnosis) and patients with early (between 18 months after diagnosis and 6 months after cessation of frontline chemotherapy) isolated bone marrow relapse have been traditionally considered an homogenous group in terms of risk stratification (namely "high-risk"). Recently, within this cohort, a subgroup of patients, defined as "very high risk", have been identified because vexed by a particularly poor prognosis. An unpublished sub-group analysis of the data collected in the ALL R3 & ALL-REZ BFM 2002 trials showed that patients with a CR1 of less than 18 months have a 10-year EFS of approximately 20% (***Figure 4***). From the IntReALL2010 trial, it was also possible to analyse the impact of specific cytogenetic alterations. Patients carrying KTM2A/AF4, E2A/TCF3-PBX1 t(1;19) or E2A/TCF3-HLF t(17;19), hypodiploidy (less than 40 chromosomes), and TP53 mutation and/or deletion had a 10-year EFS approximately between 17% and 8% (***Table 7***), therefore poorer than the traditionally defined HR group, and independently from the time of first relapse. Overall, the EFS of patients defined as VHR is approximately 20%, compared to 30% of traditionally defined HR patients.[5,6,8,9] In addition to the dismal chances of survival, these patients experience the additional burden of long-term sequlae following high intensity chemotherapy. As alternatives to chemotherapy are being developed, and show encouraging results in adults and children, it becomes fundamental to understand whether these VHR patients could benefit as well from treatments with monoclonal antibodies such as InO. Results with blinatumomab as post-reinduction consolidation regimen have been encouraging and paved the way to further trials to generate additional evidence with other novel therapies. In light of the results shown in the present study, InO appears a suitable candidate for re-induction therapy to attempt improving the outcome of VHR patients and the tolerability of current treatments.

***Table 7: Outcomes of 1st relapses, BM isolated & BM combined, by genetic mutation***

| Genetic Mutation | Cohort (frequency) | EFS % (+/- SE) | OS % (+/- SE) |
|---|---|---|---|
| TP53 mutation and/or deletion | n=52/459 (11%) | 17.3 (±5.2) | 28.7 (±6.3) |
| Hypodiploidy (< 40 chromosomes) | n=11/413 (3%) | 9.0 (±9.0) | |
| TCF3-PBX1 t(1;19) or E2A/TCF3-HLF t(17;19) | n= 7/478 (1.5%) | 17.9 (±16.0) | |
| KTM2A/AF4 | n=13/530 (2.5%) | 7.7 (±7.4) | |

**Abbreviations**: EFS: Event Free Survival at 10 years; SE: Standard Error; OS: Overall Survival at 10 years; BM: Bone Marrow

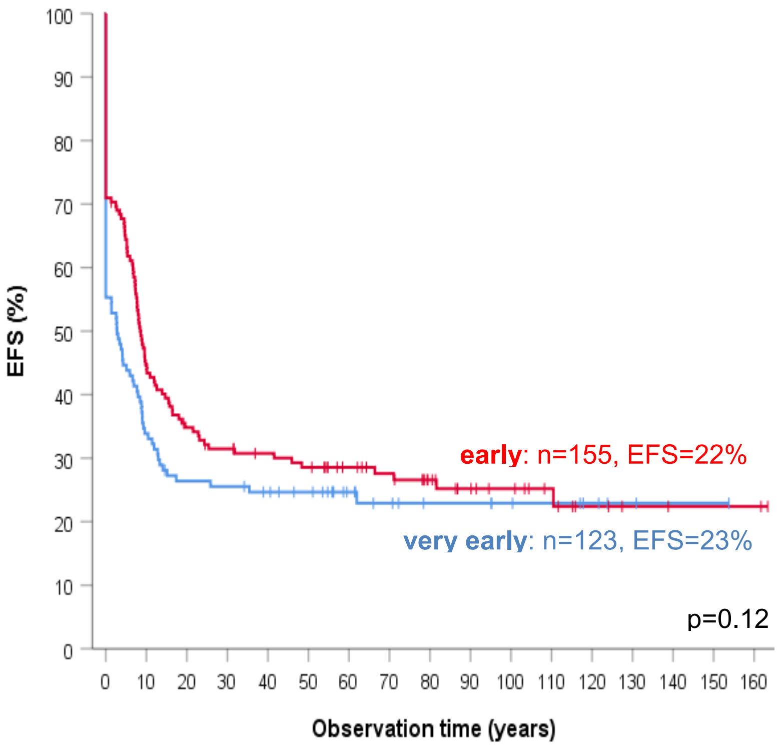


***Figure 4. Combined analysis of HR relapses ALL R3/ALL-REZ BFM 2002. Event Free Survival at 10 years (EFS) in early and very early relapse showing the VHR of induction failure after very early relapse (37%) represented by the blue line  (Courtesy Dr. A. Von Stackelberg).***

**Treatment-related mortality in relapsed ALL protocols**

Treatment-related mortality (TRM), including death during induction, does occur during ALL relapse therapy, and is mainly related to infection. In the UKALL-R3 protocol, TRM rates throughout study treatment in the idarubicin and mitoxantrone arm were 16.5% and 9.7%, respectively.[1629] These TRM rates are comparable to results reported by ALL-REZ BFM 90 trial, with 5% of deaths during induction, and 6% deaths in CCR.[20]

Overall, in UKALL-R3, treatment related events occurred mostly in the first induction period, as shown by the curves in ***Figure 5*** (disease-related events: progression, relapse, disease-related deaths and treatment-related events: treatment-related deaths, second malignancies). Regarding the specific toxicities such as grade 3 or higher liver toxicity, no events were reported by Parkers et al., although detailed data are not published.[29]

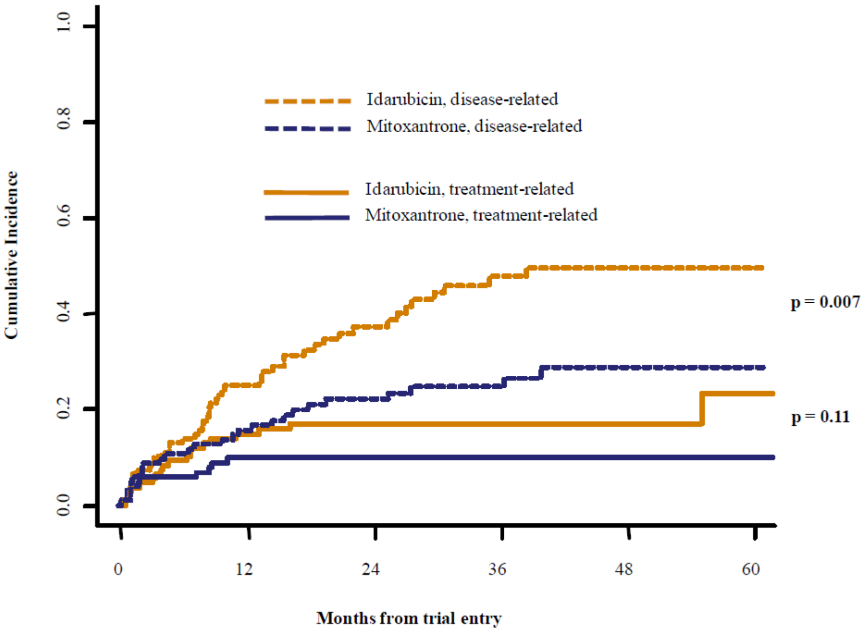


***Figure 5: Cumulative incidence of disease (progression, relapse and disease related death) and treatment related events (treatment-related deaths and 2nd malignancies) by induction in UKALL-R3.[29]***

In a retrospective study performed by the Therapeutic Advances in Childhood Leukemia & Lymphoma (TACL) consortium regarding their experience with the UKALL-R3 regimen (mitoxantrone arm only) in children with relapsed ALL, infections were the most common toxicity, with 90% of patients experiencing a grade 3 or higher infection. Other non-hematologic grade ≥3 events included electrolyte abnormalities and pain. Focusing on liver toxicity, only 2/59 patients reported grade 3 or 4 liver toxicity (increased level of bilirubin or GGT). The majority of patients had recovery of neutrophils and platelets within 4 weeks. Even though significant toxicity was observed in the majority of patients, the TRM rate was low (1.7%) and no significant difference was observed in the duration of myelosuppression, prevalence of infections or other AEs in patients treated for 1$^{st}$ versus ≥ 2$^{nd}$ relapse, or in patients with prior HSCT, which is of major interest for the cohort of patients included in the present study.[30] It is not clear why these rates were lower than reported in the original Parker publication on UKALL-R3.[29]

**Medical need**

These data document the urgent need to develop novel therapeutic strategies for high risk patients who do not adequately benefit from current chemotherapy and allo-HSCT treatments. Historically, most efforts to improve outcomes for children with relapsed ALL have been unsuccessful. Contemporary reinduction regimens have relied on further intensification of standard chemotherapeutic agents with higher doses and/or compacted drug schedules. However, these strategies fail to address one of the major challenges present in many high-risk ALL patients at relapse - intrinsic chemoresistance. Most current strategies have not only failed to improve remission rates, but have also reached tolerability limits, with toxic death rates generally ranging from 3%-8%[30,31]. Therefore, as further dose intensification is not tolerable, new therapeutic approaches which incorporate novel agents into conventional therapeutic strategies are urgently needed to overcome chemotherapy resistance and improve outcome in high risk patients.

In recent years, a number of new agents with novel mechanisms of activity have been evaluated and developed in preclinical studies and subsequently in clinical phase I- III studies.

Whereas European Groups focused on optimizing and standardizing Phase III conventional chemotherapy trials and on improving allo-HSCT management, the COG relapsed ALL group established a strategy for childhood relapsed ALL focused on the integration of new agents into the conventional ALL relapse platform. In a series of phase I and phase II studies conducted during the last 15 years, the safety,

tolerability and activities of new agents such as the antibody-drug conjugate epratuzumab (COG ADVL04P2; NCT00098839) and the proteosome inhibitor bortezomib (COG AALL07P1; NCT00873093) were explored, paving the way for future integration of new agents into relapsed ALL protocols in an international scenario[32,33].

The European Resistance Disease Committee, composed by representatives of the different European national study groups under the umbrella of the International BFM Study Group, is currently performing the IntReALL 2010 study, the first international Phase II/III trial for the treatment of childhood ALL in first relapse. The main objective for standard risk patients is to compare in a randomized way outcomes using the two most widely used conventional European protocols for relapsed ALL (ALL BFM REZ 2002 versus ALL UK R3) in order to optimize and standardize the treatment for standard risk patients. The IntReALL 2010 protocol for high risk patients will have the aim of exploring new agents and targeted therapies, such as bortezomib and blinatumomab, in addition to conventional chemotherapy and allo-HSCT, in order to improve the outcome of this specific subgroup of patients.

**ALL at 2nd or subsequent relapse**

ALL at 2nd relapse has very poor outcome rates, and remissions are often short-lived. Most groups consider consolidation with hematopoietic stem-cell transplantation (HSCT) required to potentially obtain cure[34].

There are only a few published reports regarding the outcome of children at second relapse of ALL. In a study by Chessels et al, the outcome of 439 patients who obtained a second CR (91.5%) after first relapse was reported. Of these 439 patients a group of 235 patients achieved a third CR after a second relapse. Outcome in this latter group of patients was very poor with 103 early failures and a high incidence of subsequent relapses, resulting in an OS of less than 10% for the patients treated with chemotherapy alone, and approximately 20% in transplanted patients. Patients who received HSCT in first or second remission had a dismal outcome at subsequent relapse.

Saarinen-Pihkala et al reported on 854 patients with a first and 274 patients with a 2nd relapse, and found that patients in CR3 had a 36% chance of OS for those receiving HSCT, and 15% for those receiving chemotherapy only[36].

In a retrospective cohort review of children between the ages of 0 and 21 years treated in North-America with relapsed and refractory ALL treated between 1995 and 2004 by Ko et al, CR rates (mean +/- SE) were 83% +/- 4% for early first marrow relapse, 93% +/- 3% for late first marrow relapse, 44% +/- 5% for second marrow relapse, and 27% +/- 6% for third marrow relapse. Five-year DFS rates in CR2 and CR3 were 27% +/- 4% and 15% +/- 7% respectively.[37] There is obvious medical need in this 2nd/3rd relapse population for better remission induction.

Most collaborative groups therefore regard patients with refractory ALL (after at least 2 induction regimens), as well as patients with early relapse on treatment or patients with second relapse eligible for phase II studies of experimental chemotherapy.

Several new-agent studies have recently been completed in this population:

- Single-agent Clofarabine resulted in approximately 20% response (CR/CRp) in the single-agent phase 2 study[38]. In a phase 2 combination study, clofarabine (40 mg/m$^2$ per day) was combined with cyclophosphamide (440 mg/m$^2$ per day) and etoposide (100 mg/m$^2$ per day) for five consecutive days. The ORR for the 25 patients with ALL was 44%[39].
- Blinatumomab showed a 32% response rate in the phase 2 part of the pediatric blinatumomab study in relapsed/refractory ALL patients[40].
- Bortezomib added to chemotherapy: this combination showed a favorable response rate of 73% in the (uncontrolled) TACL T2005-003 study of Bortezomib added to a vincristine, dexamethasone, asparaginase and doxorubicine backbone[33,41]. This response rate is remarkable and needs to be repeated in a controlled fashion. Such studies are currently underway.

## 2.2 CD22 and its expression in childhood ALL

CD22 (encoded on chromosome 19q13.1) is a B-cell adhesion molecule, a 140 kDa transmembrane immunoglobulin-like lectin which specifically binds sialic acid at its N-terminus. The presence of immunoglobulin domains makes CD22 a member of the immunoglobulin supergene family. It is characterized by an N-terminal IgV-like domain followed by 6 IgC-like domains in the extracellular region, a transmembrane region and a long cytoplasmic tail[42-44]. CD22 acts as an accessory co-receptor that modulates B-cell receptor (BCR) signalling upon ligation. Independently from ligation, the BCR associated kinase Lyn phosphorylates tyrosines of the cytoplasmic CD22 tail leading to immunoreceptor tyrosine-based inhibitory motifs. This mechanism inhibits BCR signaling, enhances calcium efflux and leads to endocytosis. Furthermore, CD22 is involved in the CD19/CD21and CD40 signaling regulation, peripheral B-cell homeostasis and survival and the promotion of BCR-induced cell cycle progression[45-47]. The CD22 antigen is considered to be an ideal target for conjugate delivery because of its expression profile as well as its intracellular trafficking. The normal function of CD22 is to regulate signal transduction initiated by antigen binding to the surface immunoglobulin receptors on B cells. The N-terminal immunogobulin domain of CD22 recognizes a 2,6-linked sialic acid residues on various surface glycoproteins expressed on the interacting cells. CD22 is also an attractive target for conjugate delivery due to the high level of internalization molecules among the B lymphoid lineage-specific surface antigens and due to the lack of shedding into the extracellular environment[45-47]. Its rapid endocytosis upon ligand binding qualifies it as an ideal target for immunoconjugated toxins whose cytotoxicity is mediated intracellularly against the target cell.

CD22 is expressed on both normal cells of the mature B-lymphocyte lineage and on the malignant cells of the majority of B-cell cancers. During normal B-cell development, CD22 is already expressed in the most immature B-cell stages, even before CD19 is expressed. Once expressed, CD22 persists on B cells until they differentiate into plasma cells. CD22 is not expressed on hematopoietic stem cells or other hematopoietic or non-hematopoietic lineages, with the exception of dendritic cells and basophils[48,49]. In line with its expression in normal B-cells, CD22 is highly expressed in more than 90% cases of childhood B-precursor ALL and in virtually all patients with newly diagnosed indolent, intermediate grade, or aggressive non-Hodgkin lymphoma (NHL). Interestingly, CD22 expression remains stable during the early phase of relapsed ALL treatment, rendering it a suitable target for therapeutic strategies in the post-induction phase of treatment as well as during induction. Furthermore, different from AML, ALL stem cells may be derived from more committed progenitors (such as CD34+CD19-, CD34+CD19+ and CD34-CD19+ cell fractions, which all show leukemia initiating potential in NOD/SCID mice), and therefore targeting CD22-expression may result in activity directed against ALL stem cells[48,49].

## 2.3 Other CD22 positive B-cell malignancies

Other pediatric CD22-positive malignancies include patients with lymphoblastic lymphoma, mature Burkitt ALL and NHL, diffuse large B-cell lymphoma (DLBCL) and primary mediastinal large B-cell lymphoma (PMBCL). As reported in the table below, these are variously distributed per age, the most common NHL subtypes in childhood.

***Table 8. Incidence of NHL in pediatric population***

| | Incidence of NHL per Million Person-Years | | | | | | | |
|---|---|---|---|---|---|---|---|---|
| | **Males** | | | | **Females** | | | |
| **Age (y)** | <5 | 5–9 | 10–14 | 15–19 | <5 | 5–9 | 10–14 | 15–19 |
| Burkitt | 3.2 | 6 | 6.1 | 2.8 | 0.8 | 1.1 | 0.8 | 1.2 |
| Lymphoblastic lymphoma | 1.6 | 2.2 | 2.8 | 2.2 | 0.9 | 1.0 | 0.7 | 0.9 |
| DLBCL | 0.5 | 1.2 | 2.5 | 6.1 | 0.6 | 0.7 | 1.4 | 4.9 |
| Other (mostly ALCL) | 2.3 | 3.3 | 4.3 | 7.8 | 1.5 | 1.6 | 2.8 | 3.4 |

From Childhood Non-Hodgkin Lymphoma Treatment (PDQ®): Health Professional Version. 2017 Jun 16[50]

Please notice that distribution of NHL subtypes and the spectrum of NHL occurring in children and adolescents differ significantly from adults, as illustrated in the figure below. Except for DLBCL, all the reported typical pediatric subtypes are rare in adults, where mainly indolent lymphomas predominate.

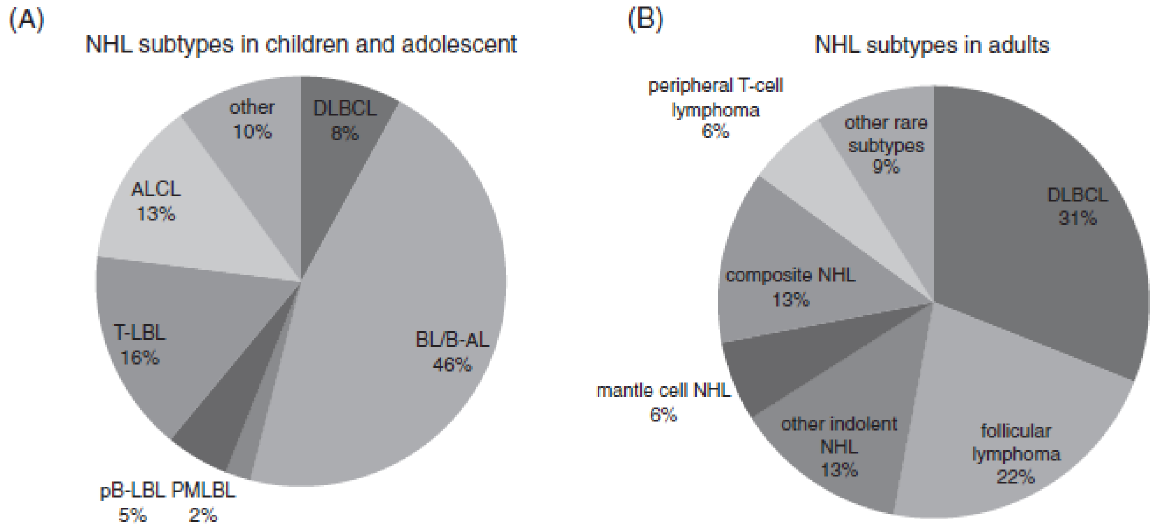


***Figure 6. NHL subtypes in pediatric and adult patients***[51]
T-LBL, lymphoblastic T-cell lymphoma; pB-LBL, lymphoblastic B-cell lymphoma; BL/B-AL, Burkitt lymphoma and Burkitt leukemia; DLBCL, diffuse large B-cell lymphoma; PMLBL, primary mediastinal large B-cell lymphoma; ALCL, anaplastic large cell lymphoma; other, rare subtypes ND NHL NOT FURTHER CLASSIFIED.

At first relapse, these specific pediatric NHL subtypes all have poor outcome[52]. For instance Burkitt ALL and NHL salvage strategies have been mostly unsuccessful, and many patients do not achieve a 2$^{nd}$ CR after relapse, despite excellent outcome rates in newly diagnosed disease (90% cure). Current treatment for relapse includes re-induction with R-ICE, a very intensive chemotherapy block combined with rituximab. DLBCL and PMBCL are rare subtypes of NHL, and newly diagnosed PMBCL is known to have a relatively poor outcome.

In lymphoblastic lymphoma outcome very much resembles the aforementioned results presented for BCP-ALL, as this is the NHL-counterpart of BCP-ALL. Usually at 1$^{st}$ relapse these patients are treated on relapse protocols for ALL, and 2$^{nd}$ relapse is considered an indication for experimental therapy, similar to BCP-ALL. Some of these patients may also be entered on trials with immune checkpoint inhibitors.

## 2.4 Inotuzumab Ozogamicin

InO (CMC-544) is classified as an antibody-drug conjugate (ADC) composed of a monoclonal CD22-targeted antibody linked to calicheamicin, a potent cytotoxic antitumor antibiotic. Antibody-targeted chemotherapy enables a cytotoxic agent to be delivered specifically to tumor cells by conjugating the cytotoxic agent with a monoclonal antibody that binds to a tumor-associated antigen. The targeting agent in InO is a humanized immunoglobulin type G, subtype 4 (IgG4) antibody, G544, which specifically recognizes the human CD22 antigen. Being an IgG4 isotype antibody, G544 is not expected to mediate effector functions such as complement-dependent cytotoxicity or antibody-dependent cellular cytotoxicity[53,54].

Antibody-targeted chemotherapy relies on the specific binding of the target: once the monoclonal antibody-drug conjugates to the tumor antigen, the antigen-antibody complex is internalized and the cytotoxic agent is delivered inside the targeted tumor cells. This strategy maximizes antitumor efficacy as it delivers the cytotoxic agent to targeted tumor cells, reducing exposure of calicheamicin to normal non- malignant cells which lack the targeted antigen, resulting in a significantly improved therapeutic index and less toxicity to the normal cell compartment[55,56].

Calicheamicins are DNA minor-groove-binding cytotoxic natural products that are significantly more potent than conventional cytotoxic chemotherapeutic agents *in vitro* and cause cell death by inducing double-strand DNA breaks[48,49,57,58]. The first antibody-targeted chemotherapy introduced into practice was gemtuzumab ozogamicin (Mylotarg), a CD33-targeted immunoconjugate of calicheamicin. Mylotarg was approved by the US Food and Drug Administration (FDA) for the treatment of patients with CD33-positive acute myeloid leukemia (AML) in first relapse aged 60 years or older and not eligible to other cytotoxic chemotherapy. Mylotarg was subsequently withdrawn from commercial availability in the US in October 2010 after failing to confirm clinical benefit in a phase III study where the experimental drug was added to conventional chemotherapy in previously untreated younger AML patients[59-62].

InO (Besponsa®) was approved by the European Medicines Agency (EMA) on June 27th, 2017 for the treatment of adults with relapsed/refractory (R/R) CD22+ BCP ALL, and by the Food and Drug Administration (FDA) on August 17th, 2017. InO is expected to have activity in other pediatric B-cell malignancies that express CD22 such as NHL (see below).

2.4.1 Inotuzumab Ozogamicin Preclinical Studies

Nonclinical pharmacodynamic studies indicate that InO can be an effective therapeutic agent against B-lymphoid malignancies and support its clinical evaluation as a targeted therapeutic option for B-cell NHL and ALL.

In preclinical studies, InO showed high affinity in binding CD22 antigen with a dissociation constant [Kd] of approximately 150 pM and exhibited a potent dose-dependent cytotoxicity against CD22 positive malignant B-cells. Particularly, InO was between 7- and 100-fold more potent than an isotype-matched nonbinding conjugate gemtuzumab ozogamicin (CMA-676) against CD22 positive B-lymphoma cells. Unconjugated anti-CD22 monoclonal antibody (the CD22-targeting antibody in InO) had no effect on the growth of various B- lymphoma cells in vitro and in vivo, while InO caused dose-dependent regression of B-lymphoma xenografts grown as subcutaneous solid tumors in athymic nude mice. The minimum efficacious dose of InO was 10 µg of conjugated calicheamicin/kg administered on days 1, 5, and 9 (Q4Dx3), which generated >55% inhibition of Ramos B lymphoma growth. In contrast, unconjugated NAc-gamma calicheamicin DMH derivative as well as unconjugated anti-CD22 monoclonal antibody had no effect on B lymphoma growth in vivo. InO administered at ≥ 120 µg of conjugated calicheamicin/kg Q4Dx3 caused complete regression of B lymphomas with treated mice remaining tumor-free for at least 100 days. The antitumor efficacy of InO was evident regardless of the size of the tumor before the initiation of therapy[63-65].

Interestingly, preclinical studies showed that CD22 expression was essential for uptake of InO, however, repetitive renewal of CD22 expression was not needed for efficacy of InO in BCP-ALL cells[66].

In preclinical studies of InO compared to conventional chemotherapy in the same RL B-cell lymphoma model, the antitumor effect of InO was longer lasting than that of the combination regimen of cyclophosphamide, doxorubicin, vincristine, and prednisone (CHOP) combination chemotherapy. Studies of InO in association with rituximab showed a synergistic or additive antitumor therapeutic effect of combination therapy. Further preclinical studies explored the efficacy of InO in combination with cyclophosphamide, vincristine, prednisone (CVP) or CHOP by either sequential or concurrent dosing regimens: sequential administration of CHOP plus InO once every 4 days in the Ramos xenograft model showed complete tumor regression which was maintained for more than 100 days while concurrent administration of InO and CVP or CHOP in Ramos xenografts in nude mice demonstrated regression of established xenografts with significantly improvement of anti-tumor activity, whereas lethality was seen in the mice after co-injection of InO and CHOP[67-69]. The effect of InO on relapsed B cell lymphoma after CHOP or CVP therapy was also studied: relapsed Ramos xenografts were poorly responsive to re-treatment with CVP or CHOP, while regression was seen with further treatment with InO[63,67,68].

A single IV dose of InO caused increases in blood pressure in cynomolgus monkeys. InO had no effect on either the CNS or respiratory function when evaluated in rats. In an in vitro human hERG current inhibition study, no significant hERG current inhibition was observed at any concentration tested. In repeat-dose toxicity studies in rats and monkeys up to 26 weeks in duration, primary target organs included the liver (liver enzyme elevations, sinusoidal dilation with hepatocyte atrophy, hepatocyte hypertrophy and

karyomegaly, and angiectasis), bone marrow and lymphoid organs (hypocellularity), and hematologic changes (reduced platelets, red blood cell mass, and lymphocytes). Other toxicity including peripheral and central axonal degeneration and renal nephropathy were observed in rats only, and glomerulonephritis with multisystemic vasculitis was identified in 1 monkey. The reversibility of axonal degeneration and liver findings were not established following a 4-week nondosing period in rats whereas partial to full reversal of effects on the hematolymphopoietic system and kidney were observed in rats and monkeys and in liver in monkeys. InO is a clastogen, and the released cytotoxin (calicheamicin) is a clastogen and mutagen. After 4 or 26 weeks of dosing, rats developed oval cell hyperplasia, altered cell foci, and hepatocellular adenomas in the liver. In 1 monkey, a focus of hepatocellular alteration was detected at the end of the 26-week dosing period. The relevance of these animal findings to humans is uncertain. InO caused testicular degeneration in and atrophy of ovaries, uterus, and vagina in male and female rats, respectively.

There were no events of hepatic pre-malignant or malignant lesions in patients with relapsed or refractory B-cell ALL or relapsed or refractory B-cell NHL treated with single-agent InO or those treated with InO plus chemotherapy. There were no InO related effects on respiratory rate, tidal volume or minute volume in rats.

Non-clinical pharmacokinetics indicated that coadministration of InO with inhibitors or inducers of CYP or UGT drug metabolizing enzymes are unlikely to alter exposure to N-Ac-γ-calicheamicin DMH. In addition, clinical metabolic DDI resulting from the inhibition or induction by inotuzumab Ozogamicin or N-Ac-γ-calicheamicin DMH of the metabolic clearance of concomitant drugs that are substrates for CYP1A2, CYP2B6, CYP2C8, CYP2C9, CYP2C19, CYP2D6, or CYP3A4/5 (inhibition) or CYP1A2, CYP2B6, or CYP3A4 (induction) are not likely to occur. The potential for N-Ac-γ-calicheamicin DMH to inhibit selected UGT enzymes (UGT1A1, UGT1A4, UGT1A6, UGT1A9, and UGT2B7) and transporters (P-gp, BCRP, OATP1B1, OATP1B3, OAT1, OAT3, and OCT2) was low at clinically relevant concentrations.

## 2.5 Clinical trials of Inotuzumab Ozogamicin

Based on promising preclinical results, InO was subsequently studied in phase I-III trials as single-agent as well as in combination with rituximab ± conventional chemotherapy in adult patients with relapsed/refractory B-NHL, and as a single agent for patients with B-cell ALL. (Please note that Pfizer-sponsored studies are denoted by prefix B193)

### 2.5.1 Studies in Adults with CD22 positive Non-Hodgkin's B-cell Lymphomas (DLBCL, follicular lymphoma, mantle-cell lymphoma, marginal zone or small lymphocytic lymphoma)

A phase I single-agent InO dose-escalation trial (B1931002) explored the safety and tolerability of the drug in adult patients with CD22-positive NHL (79 patients) with definition of the MTD of single-agent InO at 1.8 $mg/m^2$ administered every 28 days, based on Grade 4 hematologic toxicity (thrombocytopenia and neutropenia lasting more than 7 days) reported following the evaluation of the highest dose. Preliminary data on efficacy showed a 41% ORR.

A similar phase I single-agent study (B1931016) was conducted in Japan, treating 13 patients with B-cell NHL with confirmation of the MTD and preliminary identification of 80% of ORR in the subgroup of patients treated with the MTD.

These two studies paved the way for a Phase II trial of single-agent InO (B1931007): 81 subjects with indolent NHL (defined as follicular, marginal zone, or small lymphocytic lymphoma), who were refractory to or had relapsed within 6 months of rituximab in combination with chemotherapy or within 12 months of radioimmunotherapy, were treated with InO RP2D. The ORR was 67%, with InO administrated IV at the dose of 1.8 $mg/m^2$ on a 28-day cycle for 4–8 cycles.[70] Further trials explored InO in combination with rituxumab, defining the safe and tolerable profile of this combination in CD22 positive relapsed/refractory NHL.

Subsequently, two simultaneous phase I/II trials (B1931004 and B1931005) combined InO with rituximab in 119 patients with follicular NHL or aggressive NHL, predominantly diffuse large B-cell lymphoma and 10 relapsed CD20/CD22-positive NHL, defining the MTD of InO in combination with rituximab (1.8 $mg/m^2$ InO plus rituximab 375 $mg/m^2$) and showing an 87% ORR in follicular NHL, 74% ORR in relapsed DLBCL, and 20% in refractory aggressive NHL.[71] Another Phase II trial of InO (B1931001) combined with rituximab,

evaluated the safety and efficacy of treatment followed by autologous SCT in patients with R/R DLBCL, showing modest activity in this subgroup of patients with an ORR of 28.6% after 3 cycles.[72]

A phase I/II trial (B1931003) combined InO with conventional chemotherapy for CD22-positive NHL such as R-CVP (rituximab in combination with cyclophosphamide, vincristine, and prednisone) or R-GDP (rituximab in combination with gemcitabine, dexamethasone, and cisplatinum); the tolerability, safety and MTD of these combinations were determined.[73,74]

A trial combining InO plus rituximab, cyclophosphamide, vincristine and prednisolone in chemotherapy-naïve patients with DLBCL who are not candidates for anthracycline-based treatment is ongoing (NCT01679119).

A 2–arm randomized, Phase III trial (B1931008) compared the efficacy and safety of InO plus rituximab (R-InO) versus chemotherapy (investigator's choice: gemcitabine or bendamustine) plus rituximab (R) for R/R aggressive B-cell NHL (about 90% DLBCL) in patients who were not candidates for high-dose chemotherapy. The majority of patients in the control arm received R-bendamustine. Overall survival was not significantly different in the two arms and the study was prematurely terminated for futility.

Hematologic toxicity (thrombocytopenia and neutropenia) was the most frequent adverse drug reaction observed; however, only a small fraction of the events were reported as serious adverse events. In general, hematologic toxicity was described more frequently in patients who received InO in combination with rituximab plus standard cytotoxic chemotherapy than in patients who received InO as a single-agent or in combination with rituximab. Other common adverse events reported following treatment with InO were hepatic events such as low grade liver function test abnormalities. Nevertheless sometimes serious and, occasionally, fatal InO-related hepatic events also occurred. Infections, particularly pneumonia and upper respiratory infections associated with severe neutropenia, were present in patients treated with InO. Finally, nausea, vomiting, abdominal pain, constipation, diarrhea and decreased appetite were other common adverse events described, but these were generally low grade.

Please note that the immunophenotypes of NHL patients included in these adult clinical trials differ from the pediatric NHL cases that we aim to enroll in this study. In particular, indolent lymphomas such as follicular lymphoma, marginal zone lymphoma or small lymphocytic lymphoma included in adult studies are not included in the spectrum of pediatric NHL. Adult data regarding treatment with InO in typical pediatric NHL such as lymphoblastic lymphoma is minimal, and for Burkit's lymphoma is nil. In Study B1931022, among 11 adults treated with single agent InO for B-cell lymphoblastic lymphoma, the CR/CRi rate was 54.5%.

2.5.2 Studies in Predominantly Adults with Acute Lymphoblastic Leukemia (ALL)

The MD Anderson Cancer Center (MDACC) conducted a single institution, phase 2, investigator-initiated trial exploring the safety and efficacy of InO in predominantly adult relapsed/refractory ALL. The first part of this trial treated patients at the RP2D of 1.8 mg/m$^2$ every 3-4 weeks; subsequently the trial was amended with a change in InO dose schedule to fractionated weekly administration (0.8 mg/m$^2$ on day 1 and 0.5 mg/m$^2$ on day 8 and 15 every 3-4 weeks for the same total dose of InO of 1.8 mg/m$^2$ per cycle). Nineteen percent of patients achieved a CR, 27 patients (30%) had a CR without platelet recovery, and 8 patients (9%) had a CRi, with an ORR of 58%. Response rates with weekly and single-dose InO were similar. Single-dose InO therapy was associated with transient febrile and hypotensive episodes shortly after the first InO infusion, a higher incidence of liver function abnormalities and more frequent and severe hepatic veno-occlusive disease (VOD) after allo-HSCT. Adverse events were less frequent with the weekly schedule of InO and were probably related to the peak levels of InO. Peak InO levels were not associated with differences in the response rate for the weekly versus single-dose clinical experience, supported by pharmacokinetic studies, indicating that weekly InO is as effective as single-dose InO per cycle, but may be less toxic.[76,77]

Results describing 5 children (4-15 years of age) enrolled on this MDACC study were published by Rytting et al. Three of the five children received 1.3 mg/m$^2$ InO given once every 3 weeks; one of these patients had a dose escalation to 1.8 mg/m$^2$ for the second cycle. Two patients received the weekly schedule of InO

as described above. All children received at least 2 cycles of InO. One patient achieved a CR, two patients achieved bone marrow morphologic remission with incomplete platelets count recovery (CRp) while two patients had no response. Toxicities consisted of fever, sepsis and liver enzyme elevation. Three patients proceeded to transplant following InO therapy, and one of these transplanted patients developed VOD following an unrelated donor SCT. The episode of VOD in this patient resolved after therapy with defibrotide. This experience showed a safe and tolerable profile, as well as demonstrating promising activity of InO in pediatric patients with relapsed and refractory ALL[78].

Next, a phase I/II multi-institutional, single-agent InO dose-escalation trial (B1931010) was conducted in adult patients with relapsed/refractory ALL in order to test the safety and tolerability of the drug: a phase I dose-finding, dose-escalation part (n=37) [total dose 1.2 mg/m$^2$ (n=3), 1.6 mg/m$^2$ (n=12), or 1.8 mg/m$^2$ (n=22)] was followed by an expansion Phase II cohort (n=35 patients who had failed at least 2 prior treatment regimens). Overall, the median age was 45 years (range 20-79); 76.4% were Salvage status ≥ 2; 31.9% had received a prior HSCT and 22.2% were Ph The phase I dose-escalation component was performed to optimize the InO dose and schedule with weekly doses. The trial was developed after preclinical studies suggested that lower dose and more frequent schedules of the drug, compared to single-cycle dose, may improve anti-ALL activity and reduce toxicities[66]. The study showed a single DLT characterized by grade 4 elevated lipase. The most frequent (≥10% of patients) treatment-related adverse events were thrombocytopenia (31%, all grade 3/4), neutropenia (15%), and elevated transaminases (15%). The RP2D with this weekly schedule was determined to be 0.8 mg/m$^2$ on day 1, and 0.5 mg/m$^2$ on days 8 and 15. CR or CR with incomplete counts recovery (CRi) was achieved across all InO dose levels with a hematological remission rate of 74%[80]. The MTD was not reached but the RP2D was determined to be 1.8 mg/m$^2$/cycle administered every 3-4 weeks. In conclusion, InO was generally well tolerated in these 72 adult patients, and efficacy data showed an ORR of 68% after treatment with single-agent InO.

This experience paved the way for an open-label, pivotal randomized phase III trial (B1931022) of weekly InO (0.8 mg/m$^2$ on day 1, and 0.5 mg/m$^2$ on day 8 and 15 every 3-4 weeks) versus intensive chemotherapy (either fludarabine/cytarabine/granulocyte colony-stimulating factor [FLAG], or Cytarabine (ara-C) plus mitoxantrone, or high-dose ara-C) in adult patients with refractory and relapsed ALL as first or second salvage therapy, with the aim of evaluating whether InO has superior efficacy (CR/CRi and overall survival) compared to investigator's choice of chemotherapies in this patient population. Results, with 218 of 326 randomized patients included in the efficacy analysis, showed a CR/CRi rate significantly higher in the InO group than in the standard-therapy group (80.7% versus 29.4%, respectively). Also duration of remission and MRD negativity rates were significantly greater with InO versus intensive chemotherapies. More patients proceeded to allo-HSCT after InO treatment compared to intensive chemotherapy[81,82] Among all 326 randomised patients, the median OS was 7.7 (6.0 to 9.2) months in the InO arm versus 6.7 [4.9 to 8.3] months in the control arm. The survival probability at 24 months was 22.6% in the InO arm and 9.6% in the control arm with a hazard ratio of 0.77 (97.5% CI, 0.58 to 1.03) and 2-sided p-value = 0.0407. Safety results among the 164 patients treated with InO: The most common (≥ 20%) adverse reactions were thrombocytopenia (51%), neutropenia (49%), infection (48%), anaemia (36%), leukopenia (35%), fatigue (35%), haemorrhage (33%), pyrexia (32%), nausea (31%), headache (28%), febrile neutropenia (26%), increased transaminases (26%), abdominal pain (23%), increased gamma-glutamyltransferase (21%), and hyperbilirubinaemia (21%). Most common grade ≥3 adverse events were hematologic cytopenias[83]. The most common (≥ 2%) serious adverse reactions were infection (23%), febrile neutropenia (11%), haemorrhage (5%), abdominal pain (3%), pyrexia (3%), VOD/SOS (2%), and fatigue (2%). A recent update on liver toxicity in these patients was published by Kantarjian et al. Treatment-emergent hepatotoxicities of all grades were more frequent in the InO group than in the standard care group (51% versus 34% of patients). The frequency of SOS was higher in the InO group (overall 13% versus <1%). Of the 77 patients who received InO and proceeded to HSCT, 17 (22%) had SOS; five events after follow-up HSCT were fatal. Of 32 patients who received standard care and proceeded to HSCT, one (3%) had (non-fatal) SOS that was ongoing at the time of death due to septic shock.[83]

Results of 51 heavily pretreated children and young adults [aged 2.2-21.3 years (median 11.5)] with multiply (1$^{st}$-5$^{th}$ relapse) R/R B-ALL, treated with single agent InO via a compassionate access program, were published in September 2018 by Bhojwani et al. The majority of patients (N = 41, 80%) were refractory to their preceding regimen. Twenty-two patients (43%) had undergone one or more prior HSCT, 40 (78%) had

received prior CD19-directed therapy (CD19 CAR T cells and/or blinatumomab), while 10 (20%) had received prior CD22-directed therapy (CD22 CAR T cells and/or moxetumomab). 67% of the 42 patients with overt marrow disease achieved complete remission and the majority (71%) of responders were negative for minimal residual disease. All patients received the FDA approved fractionated dosing schedule of InO 3 weekly doses, one cycle consisted of 0.8 mg/m$^2$ on week 1 followed by 0.5 mg/m$^2$ on weeks 2 and 3. One patient with MRD-only disease received 0.5 mg/m$^2$/dose for all three doses. In second and subsequent cycles, patients received 0.5 mg/m$^2$/dose on days 1, 8, and 15. The median number of doses of InO received was 5 (range 1–15). Six (12%) patients developed grade 3 hepatic transaminitis or hyperbilirubinemia and 11 (22%) patients developed grade 3/4 infections. No patient developed VOD/SOS during InO therapy; however, 11 of 21 (52%) patients who underwent HSCT following InO developed SOS, with 2 fatal events. Patients who had received one or more HSCT prior to InO had a greater risk compared to patients with no prior HSCT: 6/11 (55%) vs. 3/10 (30%) respectively; and in patients whose conditioning regimens contained busulfan or clofarabine. [73,84]

As reported above, the most common toxicities associated with InO in clinical trials included thrombocytopenia and liver injury. The mechanism of these clinical toxicities is poorly understood. The mechanism of these adverse reactions were analyzed in preclinical studies in monkeys treated intravenously once every 3 weeks with a nonbinding antibody-calicheamicin conjugated (with the same linker-payload as GO and InO). The results of these studies were recently published. Acute induced thrombocytopenia (with the nadir of days 3-4) seems to be explained by the microscopic findings in liver from animals necropsied on day 3, with the loss of sinusoidal cells and marked platelets accumulation in hepatic sinusoids. The effect on megakaryocytes in bone marrow seemed less relevant, as shown in the monkeys bone marrow analysis, and also less consistent with the rapid decrease of platelets in the first 48-72 hours. Regarding liver histopathology, it showed a combination of sinusoidal capillarization and dilation/hepatocellulary atrophy, consistent with early SOS.

### 2.5.3 InO combined with chemotherapy regimens

The efficacy and feasibility of InO combined with chemotherapy regimens have been explored in some published or ongoing NHL studies.[73,74] Hematologic toxicity (thrombocytopenia and neutropenia) was the most frequent adverse event observed, and in general was described more frequently in patients who received InO in combination with rituximab plus standard cytotoxic chemotherapy than in patients who received InO as a single-agent or combined solely with rituximab. In these regimens, the MTD of InO when combined with standard-dose R-CVP (rituximab plus cyclophosphamide at 750 mg/m$^2$ day 1, vincristine at 1.4 mg/m$^2$ on day 1, and prednisone at 40 mg/m$^2$ on days1-5), was determined to be InO at 0.8 mg/m$^2$ as a single dose every 3 weeks The combination of InO (0.8 mg/m$^2$ as a single dose every 3 weeks) with R-GDP was feasible only with reduced doses of gemcitabine and cisplatin, due to hematologic toxicity.[74]

Another Phase II published trial tested the combination of InO with mini-hyper-CVD (cyclophosphamide and dexamethasone at 50% dose reduction, no anthracycline, methotrexate at 75% dose reduction, cytarabine at 0.5 g/m$^2$ x 4 doses) in older patients with ALL, and the survival results appeared to be better compared to historical results achieved with hyper-CVAD with or without rituximab (3-years OS 52% versus 36%, respectively). The first 6 patients received InO 1.3 mg/m$^2$ for cycle 1 followed by 0.8 mg/m$^2$ for subsequent cycles; patients 7 onwards received 1.8 mg/m$^2$ for Cycle 1 followed by 1.3 mg/m$^2$ for subsequent cycles. After the occurrence of VOD/SOS in 4 patients (3 during study therapy and 1 after follow-up HSCT), the dose of InO was modified to 1.3 mg/m$^2$ for Cycle 1 followed by 1.0 mg/m$^2$ for subsequent cycles.[86,87]

Other studies combining InO with chemotherapy for ALL are ongoing in adults: the NCT01925131 trial is testing the combination of InO with CVP in R/R ALL; while the NCT03150693 is a randomized Phase III trial focused on evaluation of the efficacy of InO plus standard frontline therapy for young adults with newly diagnosed BCP-ALL.

Importantly, no prior clinical trials have investigated the combination of InO with an asparaginase-containing regimen.

## 2.6 Biomarker and PK studies

Efficient killing of ALL cells by InO depends on binding of the drug to the CD22 antigen on the ALL cells, followed by internalization of the antibody-antigen complex and subsequent activation of calicheamicin. Although most ALL cells do express CD22, the level of positivity may vary and this may impact the efficacy of InO. However, in Study B1931022, remission rates were significantly higher with InO than with standard therapy among patients with both higher (≥90%) and lower (<90%) levels of CD22 positivity. Nevertheless, previous studies using Mylotarg did not find a clear relation between CD33 expression levels and response to Mylotarg[88,89,90] and also in vitro studies using InO did not show a relation between CD22 expression levels and response. This in part may be explained by the major contribution of drug efflux mechanisms and/or the high intrinsic sensitivity of ALL cells to calicheamicin. Based on the exposure response analyses for CR/CRi in clinical studies in adults, the CD22 postivity was a covariate indicating that patients with higher CD22 positivty had a greater probability of response; however it was shown that even those with lower CD22 positivity could achieve comparable level of response at later cycles when higher serum drug concentrations are reached.

After infusion of InO, the drug binds to CD22 antigens present on the ALL cells. Van der Velden, et al have previously shown that for Mylotarg near complete saturation is necessary for efficient induction of cell kill[91,92]. Therefore it is of importance to evaluate the CD22 saturation after infusion of the drug. Binding of InO to the ALL cells can be evaluated using a flow cytometric assay applying a biotinylated anti-human IgG4 antibody, comparable to the one used for Mylotarg[92]. Since InO will be administered intravenously, the drug may first bind to CD22 expressed by circulating ALL cells. Depending on the applied dose and "peripheral CD22 antigenic load", remaining InO may be delivered in the bone marrow and there bind to the ALL cells. As the leukemic stem cells are assumed to reside in the bone marrow, optimal delivery of InO to the bone marrow seems crucial. Previous studies using Mylotarg have shown that saturation levels in bone marrow were non-optimal in a substantial number of patients, which may have contributed to the suboptimal clinical response[91].

InO pharmacokinetics were well characterized by a 2-compartment model with linear and time-dependent clearance component. In 234 patients with relapsed or refractory ALL, the clearance of InO at steady-state was 0.0333 L/hr and the mean terminal elimination half-life ($t_{1/2}$) was 12.3 days. Following administration of multiple doses, a 5.3-fold accumulation of InO was observed between Cycles 1 and 4. In the clinical setting, unconjugated calicheamicin could be detected in some patients, but serum levels were typically below the limit of quantitation of 50 pg/mL.

The present trial includes a weekly infusion of InO, which has been demonstrated to be well tolerated in single-agent dose-escalation trials in adult patients with R/R ALL.[75,76,78,82] Moreover preclinical studies suggested that lower dose and fractionated schedules of the drug, compared to single-cycle high dose, may improve anti-ALL activity and reduce toxicities, probably related to the peak levels of InO.[66]

In Study B1931010 (US only) and B1931022 (global study), VOD was reported in 2.6-3.6% of patients treated with InO, while 8% (n=2/24) and 21% (n=10/48) of patients who proceeded to SCT after InO treatment developed VOD, respectively. In a preliminary analysis of VOD risk factors after treatment with InO, the use of dual alkylating conditioning regimens and/or proceeding to a second SCT seemed to be most predictive.

## 2.7 Rationale for this study

### 2.7.1 BCP-ALL

This study is part of an approved 'Pediatric Investigational Plan' (PIP) by the European Medicines Agency (EMA).

Given the activity of InO in adult ALL and the medical need in pediatric relapsed/refractory ALL, development of InO in pediatric ALL seems highly warranted. At first we will establish the MTD as single agent (in a fractionated schedule), followed by a phase 2 cohort to determine the ORR of single-agent InO and to collect additional safety and pharmacokinetic data at the MTD. Blocks of single agent InO may subsequently be studied incorporated into existing ALL regimens.

However, pediatric ALL is usually not treated with single-agent chemotherapy due to the rapid induction of resistance, certainly not when bulky disease is present. Moreover, in high-risk R/R ALL, re-induction remission rates are highly unsatisfactory with currently available chemotherapy regimens. More effective reinduction blocks are urgently needed. Therefore, in Stratum 1B, InO will be incorporated into a modified version of an existing ALL re-induction regimen (UK-ALLR3) to determine its safety, first without and then with pegylated asparaginase. If this modified re-induction regimen can be successfully developed, a randomized study for high-risk 1$^{st}$ relapse of pediatric BCP ALL will follow. Patients will be randomized against the best re-induction regimen currently available (UK-ALLR3), with the aim to improve outcome by increasing response rates and reducing transplant-related mortality (TRM) by providing targeted delivery to ALL-cells rather than non-targeted and toxic anthracycline therapy. Exclusion of anthracycline(s) during relapse ALL therapy will hopefully result in reduced myelosuppression in the short term, and reduced long-term cardiotoxicity for long-term survivors.

The UKALL-R3 protocol is a relatively toxic re-induction regimen. Mitoxantrone is considered to represent the most toxic component of this regimen. Since InO also contains an anti-tumor antibiotic (calicheamicin) and as the combination of mitoxantrone and InO is considered too toxic due to overlapping hematological toxicity profiles, we aim to develop a re-induction block based on the UKALL-R3-regimen by substituting weekly doses of InO in place of mitoxantrone.

The UKALL-R3 protocol also contains asparaginase, which is well recognized for its potential liver toxicity. Asparaginase is directly toxic to hepatocytes resulting in inhibition of protein synthesis and export of lipoproteins and lipids, with resultant steatosis and hepatic dysfunction. Some abnormal liver test results occur in almost all patients treated with asparaginase: most patients have a rise in alkaline phosphatase levels, and a lower proportion have increases in serum aminotransferase levels and bilirubin (generally mild-to-moderate in severity and self-limiting). Most typical asparaginase impairs hepatic protein synthetic function resulting in a decrease in serum albumin and clotting factors (including II, V, VII, VIII, IX, prothrombin and fibrinogen). The inhibition of clotting factor synthesis and thrombolytic activity rarely results in excess bleeding, but paradoxically may cause a hypercoagulable state. These abnormalities typically arise after 2 to 3 weeks of therapy and resolve within 2 to 4 weeks of stopping asparaginase. The hepatic dysfunction may be accompanied by hepatic steatosis, sometimes severe, and by jaundice, hepatomegaly and evidence of hepatic failure (somnolence, coma, ascites). Deaths due to asparaginase hepatotoxicity have been reported, but the rate of injury has been less frequent in recent years with the common use of pegylated asparaginase.[93,94]

Therefore, Stratum 1B will initially dose-escalate InO without asparaginase, to assess the toxicity profile of InO combined with vincristine and dexamethasone. Finally, PEG-asparaginase will be added. No mitoxantrone will be given during this modified re-induction regimen with InO.

Within the population of pediatric patients with relapse ALL, the standard risk stratification used to choose the therapeutic regimen has traditionally distinguished "standard risk" and 'high risk' patients, based on duration of first complete remission, immunophenotype (T- versus B-cell ALL) and site of relapse. More recently, the inclusion of specific genetic abnormalities was incorporated in the risk stratification of relapsed patients. The IntReALL-consortium has reported within this "high risk" relapse group a so-called "very-high risk" group. VHR is defined as isolated BM first relapse ALL or a combined first relapse with a duration of

CR 1 < 18 months with no prior HSCT during CR1, and/or cytogenetic-high risk characteristics: KTM2A/AF4, E2A/TCF3-PBX1 t(1;19) or E2A/TCF3-HLF t(17;19), hypodiploidy (less than 40 chromosomes), TP53 mutation and/or deletion. This group of patients represents an unmet medical need, that might benefit from the treatment with single agent InO induction based on the encouraging results of our phase I study and the preliminary data from the Phase II, both including R/R heavily pretreated patients. A VHR cohort will assess the efficacy and the safety profile in this specific population.

Furthermore, while data regarding the safety profile of HSCT after InO have been investigated both in adults and children, the impact of prior therapy with InO on CAR-T cells efficacy and toxicity is unknown. The most common CAR-T cells products are CD19-directed, therefore not competing on the same target antigen of InO. However, a possible expected effect on CAR-T cells efficacy may be mediated by the reduction of CD19+ positive B cells, both leukemic and native, following InO treatment. It was previously reported that marrow CD19+ antigen load of less than 15% can be correlated with suboptimal expansion and persistence of CAR-T cells. Moreover, it is not known if harvesting T-cells after InO therapy may damage T-cells and hence reduces efficacy. Although consolidation and definitive treatment will be at investigator discretion data will be captured and the number of patients proceeding to CAR-T cells therapy and/or HSCT will be reported as well as their outcome following these procedures.

### 2.7.2 Non-Hodgkin Lymphoma (NHL)

Pediatric patients with other CD22-positive malignancies can be enrolled in this study as a separate stratum. This may include patients with lymphoblastic lymphoma, mature Burkitt-ALL and -NHL, DLBCL and PMBCL.

The immunophenotypes of NHL patients included in trials in adults differ from the pediatric NHL subtypes that we aim to enroll in this study. In particular indolent lymphomas such as follicular lymphoma, marginal zone lymphoma or small lymphocytic lymphoma mentioned in adult studies are not included in the spectrum of pediatric NHL; while data about treatment with InO in typical pediatric NHL, such as Burkitt or lymphoblastic lymphomas, are not yet available. Moreover, at relapse, these specific pediatric NHL subtypes have poor outcome rates apart from lymphoblastic lymphoma, which may be enrolled at 2$^{nd}$ relapse.

# 3.0 PATIENT SCREENING, ELIGIBILITY AND ENROLLMENT

## 3.1 Informed Consent

It is the responsibility of the Investigator to obtain written informed consent for each patient and/or his/her parents or legal guardians(s) according to local law and regulations, prior to performing any trial-related procedure(s). Age appropriate assent will be obtained per national/institutional guidelines. Any other person allowed to take consent should be delegated by the Investigator as captured on the Site Signature and Delegation Log.

A Patient Information Sheet is provided to facilitate this process. Investigators must ensure that they adequately explain the aim, trial treatment, anticipated benefits and potential hazards of taking part in the trial to the patient. The Investigator should also stress that the patient is completely free to refuse to take part or withdraw from the trial at any time. The patient should be given ample time to read the Patient Information Sheet and to discuss their participation with others outside of the site research team. The patient must be given an opportunity to ask questions which should be answered to their satisfaction. The right of the patient to refuse to participate in the trial without giving a reason must be respected.

If the parents/legal guardians/patient express an interest in their child participating in the trial they should be asked to sign and date the latest approved version of the Informed Consent Form. The Investigator or authorized designate must then sign and date the form. A copy of the Informed Consent Form should be given to the parents/legal guardians/patient, a copy (a scanned image or pdf of the original is also acceptable) should be filed in the hospital medical notes, and the original placed in the Investigator File (IF). Once the patient is entered into the trial the patient's study number should be entered on the Informed Consent Form maintained in the IF.

Details of the informed consent discussions should be recorded in the patient's medical notes, this should include date of, and information regarding, the initial discussion, the date consent was given, and that a copy of the Informed Consent was received by the patient/legal guardians.

Throughout the trial the patient should have the opportunity to ask questions about the trial and any new information that may be relevant to the patient's continued participation should be shared with them in a timely manner. On occasion it may be necessary to re-consent the patient in which case the process above should be followed and the patient's right to withdraw from the trial respected.

Electronic copies of the approved Patient Information Sheet and Informed Consent Form should be available from the Investigator's Trials Office.

Details of all patients approached about the trial should be recorded on the Patient Screening/ Enrolment Log.

## 3.2 Reservation and Enrollment

Patients must be enrolled prior to beginning study-related procedures or treatment on this study. Results from laboratory tests or assessments, performed as a standard of care, prior to the date of informed consent but within the allowed timeframe for screening procedures, can be used for determining the patient's eligibility and will be entered in the CRF. Use of pre-consent results to support inclusion/exclusion criteria must be clearly documented in the patient's source documents. Note that certain biospecimens need to be shipped to a central laboratory and may require repeating procedures.

It is allowable to enroll a patient who has received IT ARA-C, IT MTX or triple IT therapy within 72 hours of enrollment as part of their evaluation to diagnose disease relapse.

Harvesting CAR-T cells in patients that will be enrolled in stratum 3 prior to InO is allowed and is considered as standard of care procedure. It is suggested to harvest T-cells prior to InO infusion – as there are some reports suggesting that T-cells may be affected by prior InO infusion.[5] Indeed, a small study collected data from 9 patients that received InO as bridging therapy before CAR-T cells infusion, among 67 subjects that in total underwent CAR-T cells therapy. Suboptimal expansion was observed in the InO group, and similar

conclusions were obtained in the 15 patients that received prior Blimatumumab.[95] Other investigators did not report issues regarding harvesting T cells post InO treatment.[96]

Subjects will be recruited from a population of children and adolescents treated at or referred to the investigational centers (study site). The investigational centers will be initiated by the sponsor before any patient screening and or registration may commence.

The investigator site staff will **pre-announce** any potential subjects by email to the coordinating principal investigator and the trial manager to make sure a slot is available. Eligibility screening may start when a slot is available and after informed consent has been obtained. After pre-registration in ALEA the subject ID is provided that needs to be used for the screening samples and correspondence on the screening results. After screening the eligibility of the patient will be confirmed in ALEA.

The site staff will submit the completed Pre-Registration Forms and Final registration Forms to the designated Sponsor study team member(s).

For each patient registration, the completed and signed (Pre) Registration Forms accompanied by essential encoded source documents need to be emailed to:

DCOG – ECTC Data Center
E-mail: dc-ectc@prinsesmaximacentrum.nl ; trialmanagement@prinsesmaximacentrum.nl

In case any issues need to be discussed prior to sending these Forms, please send an e-mail to the coordinating PI, Trial Manager, and ECTC Data Center simultaneously for discussion.

Note that it is the responsibility of the Site Investigator to ensure that the subject is eligible for the study. No waivers on the eligibility criteria can be granted.

The required type of information on the forms include:

1. Sex, date of birth, age
2. Review of eligibility criteria
3. Source documents (encoded with the subject ID): results of local CD22 expression and bone-marrow morphology as obtained during screening
4. Expected date of trial treatment start.

After checking the provided information, the Sponsor will enroll the patient by assigning a unique study subject identification number. The study subject identification number will be used on all case report form (eCRF) pages and other study related documentation or correspondence (including fax and/or email) referencing that patient.

Dose level allocation will be performed centrally after patient's legal representative has provided informed consent, the patient has provided consent/assent, and the patient has completed the necessary screening assessments.

No patient shall receive study drug until the Investigator or designee has received the following information in writing from the Sponsor:

- Confirmation of the patient's enrolment, including the study subject ID number;
- Specification of the dose level for that patient; and
- Permission to proceed with dosing the patient.

The enrolment details will be sent by automatic email notification to the investigator site staff (team), the CRO, and the sponsor study team.

The first dose of study therapy should be administered within 3 days once the patient is considered eligible following the necessary screening assessments.

In case of emergency, or when the normal Registration Procedure as described is not working appropriately, please call the DCOG – ECTC Data Center for a Registration procedure by telephone.

- Phone: +31-88-972 7671 / +31-6-5000 6679

### 3.3 Diagnostic work-up

At baseline, all patients must undergo a full leukemia/lymphoma diagnostic work-up including peripheral blood, bone marrow and cerebrospinal fluid (CSF) examination. A bone marrow biopsy is required only in case of a dry tap. Appropriate imaging of extramedullary disease (EMD)/lymphomas which allows repeated imaging with the same modality during follow-up (i.e. sonography/CT-scan or MRI-scan) is required (no central radiological review is planned). PET-scans may be used when clinically indicated and/or as part of institutional standard of care for NHL patients. When clinically indicated the presence of EMD/lymphomas may need to be confirmed by cytological or histopathological or flow cytometry examination. Harvesting T-cells for a CAR-T cells procedure may be performed as standard of care procedure per investigator discretion for stratum 3 patients during this period.

To assess response (as per Appendix 2), again a full work-up is required, including peripheral blood, bone marrow and CSF evaluation. The same radiological technique needs to be applied as used at baseline to evaluate EMD/lymphomas. PET-scans may only be used in response determination when also performed at baseline. Note that per lymphoma response criteria (given in Appendix 2.2) a resection or biopsy of a residual mass may be considered (but is not required) in the response-evaluation.

### 3.4 Eligibility criteria

The inclusion criteria are similar for the 2 strata apart from the underlying diagnosis (Stratum 1: $CD22^+$ R/R BCP-ALL versus Stratum 2: other $CD22^+$ B-cell malignancies).

In addition, the inclusion criteria are similar for Stratum 1A, 1B and the Phase 2 cohort, with the exception that Down syndrome patients are only included in the Phase 2 cohort and Stratum 3 (VHR cohort).

The eligibility criteria listed below cannot be waived.

**INCLUSION CRITERIA**

**<u>Age (for all strata)</u>**

- Patients must be ≥ 1 and < 18 years of age at the time of enrollment.

*Additional criteria for Stratum 1A and 1B only:*

- The first 3 BCP-ALL patients on dose level 1 must be aged 6 years to less than 18 years.
- Then at least 2 additional patients must be enrolled from age 1 year to less than 6 years at the same dose level.
- After this requirement is met, subsequent dose levels may enroll patients aged 1 year to less than 18 years.
- In case 2 younger patients are not yet recruited, patients aged 6 years up to less than 18 years may continue to be enrolled at dose level 1 until a maximum of 6 patients are enrolled.

**<u>Stratum 1A, Phase 2 and Stratum 1B/1B-ASP: Diagnosis</u>**

Patients must have either

- First relapse of BCP-ALL post allogeneic HSCT
- Second or greater relapsed or refractory BCP-ALL
- Refractory disease, defined as newly diagnosed patients who are induction failures after at least 2 previous regimens without attainment of remission, or patients with refractory first relapse after 1 previous reinduction regimen without attainment of remission.

**<u>AND</u>** must meet the following criteria:

- Patients must have M2 or M3 marrow status (≥ 5% blasts by morphology)

- The malignant clone needs to be CD22 surface antigen positive (in either the bone marrow or peripheral blood) by institutional standards as determined by the local immunophenotyping laboratory.
- The first 6 patients (Stratum 1A only) must have M3 marrow status (≥ 25% blasts by morphology).

**Stratum 2: Diagnosis**

Patients must have second or greater relapsed or refractory CD22-positive B-cell malignancy including but not limited to diffuse large B-cell lymphoma (DLBCL), primary mediastinal large B-cell lymphoma (PMBCL), Burkitt lymphoma, Burkitt leukemia or B-cell precursor lymphoblastic lymphoma:

- There must be histologic verification of disease at original diagnosis or subsequent relapse.
- Patient must have evaluable or measurable disease documented by radiographic criteria or bone marrow disease present at study entry.
- The malignant cells need to be CD22 surface antigen positive (in either biopsy material, the bone marrow or peripheral blood) by institutional standards as determined by the local immunophenotyping laboratory.

**Stratum 3: Diagnosis**

- First BM or combined relapse of CD22+ VHR BCP-ALL defined as any relapse <18 months from initial diagnosis and/or cytogenetic-high risk characteristics: KTM2A/AF4, E2A/TCF3-PBX1 t(1;19) or E2A/TCF3-HLF t(17;19), hypodiploidy (less than 40 chromosomes), TP53 mutation and/or deletion, as also shown in Table 2 excluding patients transplanted in 1st CR.

  **AND** must meet the following criteria:
  - Patients must have M2 or M3 marrow status (≥ 5% blasts by morphology)
  - The malignant clone needs to be CD22 surface antigen positive (in either the bone marrow or peripheral blood) by institutional standards as determined by the local immunophenotyping laboratory.

- Evidence of prior fusion gene abnormalities is acceptable as they tend to be stable during the course of the disease.
- Laboratory techniques acceptable to test the presence of the above mentioned cytogenetic-high risk characteristics are chromosome banding analysis (CBA), FISH, PCR and/or Next Generation Sequencing (inclusion is based on local laboratory results).

**Performance Level and Life Expectancy**

- Karnofsky > 60% for patients > 16 years of age and Lansky > 60% for patients ≤ 16 years of age. (See Appendix I for Performance Scales).
- Patient must have a life expectancy of at least 6 weeks.

**Prior Therapy**

Patients must have fully recovered from the acute toxic effects of all prior chemotherapy, immunotherapy, or radiotherapy defined as resolution of all such non-hematologic toxicities to ≤ Grade 2 per the CTCAE 4.03 prior to entering this study, with the exception of the authorized laboratory abnormalities as defined in the inclusion/exclusion criteria.

i. Chemotherapy:
   At least 7 days must have elapsed since the completion of cytotoxic therapy, with the exception of hydroxyurea, 6-mercaptopurine and steroids which are permitted up until 48 hours prior to initiating

protocol therapy. Patients may have received intrathecal therapy at any time prior to study entry. Patients who relapse while receiving maintenance chemotherapy will not be required to have a waiting period before enrollment onto this study.

j. Radiotherapy:
At least 28 days must have elapsed since any prior radiation therapy.

k. Hematopoietic Stem Cell Transplant:
At least 90 days must have elapsed since previous allo-HSCT. Patient must have no evidence of active graft vs. host disease (GVHD). Patient must not be receiving GVHD prophylaxis or treatment.

l. Hematopoietic growth factors:
At least 7 days must have elapsed since the completion of therapy with GCSF or other growth factors at the time of enrollment. At least 14 days must have elapsed since the completion of therapy with pegfilgrastim (Neulasta®).

m. Immunotherapy:
At least 42 days must have elapsed after the completion of any type of immunotherapy, e.g. chimeric antigen receptor T cell (CAR T) therapy. Patients may not have received prior CD22-targeted therapy (immunotoxin or CAR T therapy).

n. Monoclonal antibodies:
At least 3 half-lives of the antibody must have elapsed after the last dose of a monoclonal antibody (ie: Rituximab = 66 days, Epratuzumab = 69 days), with the exclusion of blinatumomab. Patients must have been off blinatumomab infusion for at least 14 days and all drug-related toxicity must have resolved to grade 2 or lower as outlined in the inclusion and exclusion criteria.

o. Investigational drugs:
At least 7 days or 5 drug half-lives (whichever is longer) must have elapsed since prior treatment with any experimental drug (with the exception of monoclonal antibodies) under investigation. No residual toxicities should be observed following previous treatment. An experimental drug is defined as any drug that is not approved and licensed for sale by the FDA for institutions in the United States, by the EMA for institutions in Europe, by Health Canada for institutions in Canada and by The Therapeutic Goods Administration for institutions in Australia.

p. Prior calicheamicin exposure:
Patient has not received prior treatment with a calicheamicin-conjugated antibody (e.g. gemtuzumab ozogamicin).

**Renal and Hepatic Function**

- Patient's serum creatinine must be ≤ 1.5 x institutional upper limit of normal (ULN) according to age. If the serum creatinine is greater than 1.5 x institutional ULN, the patient must have a GFR ≥ 70mL/min/1.73 $m^2$ estimated based on serum creatinine and/or cystatin C levels (e.g. Bedside Schwartz formula).

- Patient's AST and ALT must be ≤ 2.5 x institutional ULN.

- Patient's total bilirubin must be ≤ 1.5 x institutional ULN unless the patient has documented Gilbert syndrome, in which case the AST and ALT must be ≤ 2.5 x ULN.

**Cardiac Function**

- Patient must have a shortening fraction ≥ 30% by echocardiogram or an ejection fraction > 50% by MUGA.

**Reproductive Function**

- Female patients of childbearing potential must have a negative urine or serum pregnancy test confirmed prior to enrollment.
- Female patients with infants must agree not to breastfeed their infants while on this study.
- Male and female patients of child-bearing potential must agree to use a *highly effective* method of contraception approved by the investigator during the study, following the CTFG recommendations, and for at least 8 months for females and for at least 5 months for males after the last dose of InO.
- Highly effective methods of contraception include (but not exclusively) the following contraceptive methods:
  - combined (estrogen- and progestogen-containing) hormonal contraception associated with inhibition of ovulation
  - progestogen-only hormonal contraception associated with inhibition of ovulation
  - intrauterine device (IUD)
  - intrauterine hormone-releasing system (IUS)
  - sexual abstinence.

**EXCLUSION ELIGIBILITY CRITERIA**

Patients will be excluded if they meet any of the following criteria:

**Isolated extramedullary relapse**

- Patients with isolated extramedullary disease are excluded (not applicable to lymphoma patients except for isolated CNS-relapse)

**VOD/SOS**

- Patients with any history of prior or ongoing VOD/SOS per the modified Seattle criteria are excluded, as specified in Appendix 3, or prior liver-failure [defined as severe acute liver injury with encephalopathy and impaired synthetic function (INR of ≥1.5)].

**Infection**

- Patients will be excluded if they have a systemic fungal, bacterial, viral or other infection that is exhibiting ongoing signs/symptoms related to the infection without improvement despite appropriate antibiotics or other treatment.
- The patient may not have:
  - A requirement for vasopressors;
  - Positive blood culture within 48 hours of study enrollment;
  - Fever above 38.2 degrees Celsius within 48 hours of study enrollment with clinical signs of infection. Fever that is determined to be due to tumor burden is allowed if patients have documented negative blood cultures for at least 48 hours prior to enrollment and no concurrent signs or symptoms of active infection or hemodynamic instability.
  - A positive fungal culture within 30 days of study enrollment.
  - Active fungal, viral, bacterial, or protozoal infection requiring IV or oral treatment. Chronic prophylaxis therapy to prevent infections is allowed.

**Other anti-cancer therapy**

- Patients will be excluded if there is a plan to administer non-protocol anti-cancer therapy including but not limited to chemotherapy, radiation therapy, or immunotherapy during the study period.
- Patients will be excluded if they have received prior treatment with anti-tumor vaccines.

**Allergic reaction**

- Patients with prior Grade 3/4 allergic reaction to a monoclonal antibody are excluded.

**Concurrent disease**

- Patients will be excluded if they have significant concurrent disease, illness, psychiatric disorder or social issue that would compromise patient safety or compliance with protocol therapy, interfere with consent, study participation, follow up, or interpretation of study results.
- Children with Down syndrome are excluded from participation in the dose finding parts (stratum 1A and 1B), but not in the single agent phase 2 cohort or the VHR cohort..

**Additional exclusion criteria for Stratum 1B**

- Patients with grade 3-4 peripheral neuropathy (as defined in the Delphi consensus of acute toxic effects for childhood ALL by Schmiegelow et al.[12] ). Patients with prior history of thrombosis during steroid and/or asparaginase are eligible provided they use adequate anti-coagulant prophylaxis, according to institutional guidelines.
- Patients in whom prior experience suggests that a timely delivery of therapy is unlikely or associated with an undue risk because of intolerance.

**Additional exclusion criteria for Stratum 1B-ASP cohort only**

- Patients with any history of PEG-asparaginase intolerance due to allergic reactions or silent inactivation during prior treatment.
- Patients with any history of prior asparaginase-associated acute pancreatitis (any grade as defined in the Delphi consensus.[12]).

Patients who are excluded from Stratum 1B-ASP may potentially be enrolled in Stratum 1B expansion cohort.

**Additional exclusion criteria for Stratum 3 (VHR cohort) only**

- Patients who are transplanted in CR1 (such patients are eligible for the phase 1B cohort).

## 4.0 TREATMENT PROGRAM

The following sections detail the treatment plan for each cycle of therapy. Please refer to the Drug Information in Section 6.0 for additional administration guidelines

### 4.1 Treatment program for Stratum 1A, the Phase 2 Cohort, Stratum 2 and Stratum 3 patients

Treatment should begin within 7 calendar days of confirmation of eligibility after screening. A maximum of 6 cycles of InO may be given in this study for patients not proceeding to HSCT. For patients proceeding to HSCT the recommended duration of study treatment is 2 cycles, up to a maximum of 3 cycles for patients who are not yet MRD-negative after 2 cycles. Each cycle consists of 3 dosages of InO given 1 week apart, and doses should not be administered less than 6 days apart.

There is no specific recommendation for the number of cycles that may be given prior to CAR-T cells therapy. InO is mainly used in such patients as bridging therapy to control disease and cytoreduce patients prior to CAR-T cells therapy. Please note that patients may become MRD negative with InO and that in such patients we recommend to perform a very close watch and wait strategy and to delay CAR-T cells infusion until regeneration of healthy B-cells or minimal residual leukemia, although there are no published guidelines for this, apart from anecdotal evidence.[95]

It is suggested to harvest T-cells prior to InO infusion – as there are some reports suggesting that T-cells may be affected by prior InO infusion.[5] Indeed, a small study collected data from 9 patients that received InO as bridging therapy before CAR-T cells infusion, among 67 subjects that in total underwent CAR-T cells therapy. Suboptimal expansion was observed in the InO group, and similar conclusions were obtained in the 15 patients that received prior Blimatumumab.[95] Other investigators did not report issues regarding harvesting T cells post InO treatment.[96]
Timing of protocol therapy administration, laboratory evaluations and response assessment studies are based on schedules derived from the experimental design or on established standards of care. Schedule of events, laboratory evaluations and response assessments are detailed in **Table 22** (red table) for Stratum 1A and Phase 2 cohort, **Table 25** (red table) for Stratum 3 and in ***Table 26*** (green table) for Stratum 2 patients.

#### 4.1.1 Definition of Investigational Medicinal Product
The defined Investigational Medicinal Product (IMP) in this protocol is Inotuzumab Ozogamicin (InO).

#### 4.1.2 Definition of non-Investigational Medicinal Product
The defined non-IMPs in this protocol are IT methotrexate, cytarabine and prednisolone or hydrocortisone (depending on the steroid approved for IT use in your country). These drugs will be obtained from commercial source.

#### 4.1.3 Treatment Cycle
All patients should receive 1 cycle of induction therapy in the absence of progressive disease, dose limiting toxicity, or investigator/subject preference to withdraw.

A cycle of therapy is defined as 3 doses of InO administered weekly on days 1, 8 and 15. Cycle 1 will last 22 days (with delays allowed up to 42 days, depending on response and recovery from toxicity), and all subsequent cycles will last 28 days, again with delays up to 42 days.

CNS-prophylaxis in patients with CNS1 status is recommended. In case of CNS2 or CNS3, patients may receive intensified IT therapy with triple IT agents (methotrexate, cytarabine and prednisolone or hydrocortisone, depending on the steroid approved for IT use in your country) according to local practice.

## 4.2 Treatment program for Stratum 1B and 1B-ASP

Treatment should begin within 7 calendar days of confirmation of eligibility/enrollment. The treatment consists of a modified UKALL-R3 re-induction regimen, without mitoxantrone and initially without asparaginase, with the addition of 3 weekly doses of InO, as detailed in ***Table 10***. Furthermore, the dose of dexamethasone has been reduced to 10 mg/m$^2$/day with protocol amendment v4.0 instead of the 20 mg/m$^2$/day normally used in UKALL-R3.

Following the first re-induction cycle, either 1 additional cycle of the modified UKALL-R3 regimen may be given, or patients may be then be treated with single-agent InO at RP2D established in Stratum 1A (please discuss this with the Sponsor), per the discretion of the investigator.

Patients NOT proceeding to HSCT may receive a maximum of 2 combination cycles (cycle 1 and 2), followed by a maximum of 4 or 5 cycles of single-agent InO (depends on if 1 or 2 combination cycles were administered). A maximum of 6 cycles of InO may be administered for a given patient.

For patients who will proceed to HSCT, the recommended duration of InO treatment is 2 cycles, but 3 cycles (maximum of 2 as combination therapy) are allowed if the patient is not yet MRD-negative after the 2$^{nd}$ cycle.

Schedule of events, laboratory evaluations and response assessment for Stratum 1B are reported in Tables 23 and 24 (blue tables) for combination therapy and single-agent therapy, respectively.

4.2.1 Definition of Investigational Medicinal Product

The defined Investigational Medicinal Product (IMP) in this protocol is inotuzumab ozogamicin (InO).

4.2.2 Definition of non-Investigational Medicinal Product

The defined non-IMPs in Stratum 1B are dexamethasone, vincristine, and pegylated asparaginase (Stratum 1B-ASP only) administered systemically, and intrathecal (IT) methotrexate, cytarabine and prednisolone or hydrocortisone (depending on the steroid approved for IT use as standard of care in the participating country). These drugs will be obtained from commercial source.

4.2.3 Treatment Cycle

All patients will receive 1 cycle of re-induction therapy, in the absence of refractory disease, dose-limiting toxicity, or investigator/subject preference to withdraw consent for further therapy.

A cycle of therapy is defined as 3 doses of InO administered weekly (on days 1, 8 and 15) combined with the UKALL-R3 modified regimen (IV vincristine 1.5 mg/m$^2$ on days 3, 10, 17 and 24, oral dexamethasone 10 mg/m$^2$/day on days 1-5 and 15-19, divided in 2 daily doses, and IT methotrexate prophylaxis at the start of the block and at day 8.

In case of CNS 2 or 3, patients will receive triple IT agents (methotrexate, cytarabine, and prednisolone or hydrocortisone depending on the steroid approved for IT use in the country).

All cycles will last 28 days, with delays allowed up to 42 days to recover from toxicity.

Initially no pegylated asparaginase will be administered (Stratum 1B), but once a safe dose of InO in combination with dexamethasone and vincristine is established, a subsequent cohort will be added (referred to as Stratum 1B-ASP) using pegylated asparaginase 1000 UI/m$^2$ IV on days 3 and 17 of re-induction cycle 1, and possibly during cycle 2 if combination therapy is continued per the investigator's discretion.

## 4.3 Treatment Cycles

### 4.3.1 Cycle 1 (all patients treated with single-agent InO)

**Premedication**
To avoid infusion-related side-effects it is strongly recommended to pre-treat patients, directly prior to each infusion of InO with methylprednisolone 1 mg/kg IV (max 50 mg). If methylprednisolone is given, there is no need for pre-treatment with acetaminophen (paracetamol) or anti-histamine drugs such as clemastine.

In case of an infusion reaction related to InO, the infusion of InO should be discontinued and appropriate medical treatment, as needed, should be instituted according to local standards (eg, glucocorticoids, epinephrine, bronchodilators, or oxygen). Depending on the severity of the infusion reaction and interventions required, the investigator could consider restarting the infusion at a reduced rate.

**Inotuzumab Ozogamicin**

- The InO dose level is assigned at study entry (dose-levels are described in ***Table 15***), and is adjusted to BSA using the Mosteller formula as given in Appendix 4;
- For patients in stratum 3 the RP2D as established in the Stratum 1A/phase 2 cohort will be used (1.8 mg/m$^2$ in 3 weekly fractions: 0.8 mg/m$^2$ at day 1, followed by 0.5 mg/m$^2$ at day 8 and at day 15).
- There will be no dose-capping for obese patients/patients with high BSA (see also Section 4.5)
- InO is administered as an IV infusion over 60 minutes (+/- 15 minutes), on days 1, 8, and 15 (as per ***Table 9***). See section 6.1.5 for detailed administration guidelines;
- A higher dose is given on Day 1 of Cycle 1, as per Section 4.5 and ***Table 15***, and may be repeated in cycle 2 if no CR/CRi/CRp is obtained in cycle 1;
- Liver functions (toal bilirubin and transaminases) should be checked at day 8 prior to InO infusion. In case of a >2.5 x ULN elevation in AST and/or ALT, or >1.5 x ULN for total bilirubin, delay the dose according to Section 4.4.1. (excluding cases where these aberrations are due to hemolysis or Gilbert's syndrome);
- Record vital signs prior to each infusion and 1 hour (±15 minutes) after the end of each infusion. Vital signs will be also recorded 2 hours (±15 minutes) after the end of InO infusion at Cycle 1 Day 1 dose;
- These vital sign measurements should be captured in the eCRF.

***Table 9. Single-agent InO schedule for cycle 1***

| Drug | Day 1 | Day 8 | Day 15 | Day 22 (+/- 2 days) to day 42 |
|---|---|---|---|---|
| Anti-emetics (per institutional guidelines) 15 min prior to InO infusion | • | • | • | *Disease Evaluation* |
| Methylprednisolone 1 mg/kg IV (max 50 mg); 10 min prior to InO infusion | • | • | • | |
| Inotuzumab Ozogamicin; in 1 hour IV (+/- 15 min)* | • | •# | • | |
| Intrathecal Methotrexate** | • | | | |

** Record vital signs prior and post infusion; for dose-levels see **Table 15**.*
*** See below for IT treatment for CNS 2 and 3 patients and patients with other CD22-positive B-cell malignancies. Note that the intrathecal therapy may be given together with the diagnostic lumbar and/or bone marrow puncture rather than on Day 1.*
*# Liver functions (total bilirubin and transminase levels) need to be determined prior to day 8 infusion. In case of a >2.5 ULN elevation in AST and/or ALT, or >1.5 xULN for total bilirubin, delay the dose according to Section 4.4.1 (excluding cases where these aberrations are due to hemolysis or Gilbert's syndrome).*

### 4.3.2. Stratum 1B/1B-ASP Cycle 1

**Premedication**
To avoid infusion-related side-effects it is recommended to premedicate patients directly prior to each infusion of InO. On day 1 and 15 when the dexamethasone pulse is scheduled to begin, administer the first

dose of dexamethasone IV 10 minutes before InO administration (as depicted in ***Table 10***), avoiding the need for methylprednisolone premedication. On day 8, methylprednisolone 1 mg/kg IV (max 50 mg) should be given, as there is no dexamethasone scheduled at day 8. If methylprednisolone or dexamethasone is given, there is no need for pre-treatment with acetaminophen (paracetamol) or anti-histamine drugs such as clemastine.

In case of an InO-related infusion reaction, the infusion should be discontinued and appropriate medical treatment, as needed, should be instituted (eg, glucocorticoids, epinephrine, bronchodilators, or oxygen). Depending on the severity of the infusion reaction and interventions required, the investigator could consider restarting the infusion at a reduced rate. The same may apply to infusion reactions to other drugs such as asparaginase.

**Inotuzumab ozogamicin**

- The InO dose level is assigned at study entry (dose-levels are described in ***Table 15***), and is adjusted to BSA using the Mosteller formula as given in Appendix 4;
- There will be no dose-capping for obese patients (see also Section 4.5)
- InO is administered as an IV infusion over 60 minutes (+/- 15 minutes), on days 1, 8, and 15 (as per ***Table 9***). See Section 6.1.5 for detailed administration guidelines;
- A higher dose is given on Day 1 of Cycle 1, as per Section 4.5 and ***Table 15***
- Liver tests (total bilirubin, ALT and AST) should be checked at day 8 and day 15 prior to each InO infusion. In case of a >5 x ULN elevation in AST and/or ALT, or >1.5 x ULN for total bilirubin attributed to InO, delay the InO dose according to Section 4.4.1. (excluding cases where these aberrations are due to hemolysis or Gilbert's syndrome);
- Record vital signs will be recorded in the eCRF prior to each infusion and 1 hour (±15 minutes) after the end of each infusion, as well as 2 hours (±15 minutes) after the end of first InO infusion for a given patient;

***Table 10. Stratum 1B/1B-ASP combination InO schedule for cycle 1 (See Appendix 5)***

| Drug | Day | | | | | | | |
|---|---|---|---|---|---|---|---|---|
| | 1 | 3 | 8 | 10 | 15 | 17 | 24 (+/- 1 day) | 28- 42 (+/- 2 days) |
| Anti-emetics per institutional guidelines 15 min prior to chemotherapy | • | • | • | • | • | • | • | *Disease Evaluation* |
| Methylprednisolone 1 mg/kg IV (max 50 mg) 10 min prior to InO infusion; only on day 8 when no dexamethasone is administered | | | • | | | | | |
| Inotuzumab ozogamicin over 1 hour IV (+/- 15 min)* | • | | •# | | •# | | | |
| **Dexamethasone 10 $mg/m^2$/day (5 day blocks) orally divided in two daily doses** (max 20 mg/day) ** Administer $1^{st}$ dose IV on days 1 and 15; ~10 min prior to InO infusion (pre-med)** | • days 1-5 | | | | • days 15-19 | | | |
| Vincristine 1.5 $mg/m^2$ (max single dose 2 mg); IV push or 15 min infusion (+/- 5 min)*** | | • | | • | | • | • | |
| Intrathecal therapy**** | • | | •# | | | | | |
| ***For Stratum 1B-ASP only:*** PEG-asparaginase 1000 units/$m^2$ over 2 hours IV (+/- 15 min) ***** | | • | | | | • | | |

** Record vital signs prior and post infusion; for dose-levels see **Table 15**.*
***Dexamethasone 10 mg/m² (maximum 20 mg/d) orally divided into two daily doses on days 1-5 starting at day 1 and day 15. The first dose of day 1 and day 15 should be administered IV 10 minutes prior to InO infusion, avoiding methylprednisolone pre-medication.*
****Vincristine 1.5 mg/m² (maximum single dose 2 mg) as 15 min short infusion or as IV bolus on days 3,10,17,24*
***** See Section 4.3.3 for IT treatment. Note that the IT therapy may be given together with the diagnostic lumbar and/or bone marrow puncture rather than on Day 1.*

****** Pegylated-Asparaginase administration will be added after an initial period of InO dose-escalation combined with Vincristine and dexamethasone only, without asparaginase, and is referred to as Stratum 1B-ASP. The exact regimen (with and without pegylated asparaginase), and the dose of InO will be communicated at enrolment. Pegylated asparaginase is not subject to dose-capping.*
*# Liver functions (total bilirubin and transminase levels) need to be determined prior to day 8 and 15 infusion.*
*In case of a >5 (> grade 2) ULN elevation in AST and/or ALT, or >1.5 xULN for total bilirubin, delay the dose of InO and IT therapy according to Section 4.4.1 (excluding cases where these aberrations are due to hemolysis or Gilbert's syndrome).*

### 4.3.3. Intrathecal prophylaxis and therapy

#### *4.3.3.1 Intrathecal therapy (IT) for BCP-ALL patients with CNS 1 status*

Note that definitions of CNS involvement are given in Appendix 2.1.3
- IT methotrexate prophylaxis is recommended to be given intrathecally to patients with BCP-ALL who are CNS1 at study entry on day 1 of each cycle of single-agent InO per local standard of care, for example using the doses listed below in ***Table 11***.
- In Stratum 1B/1B-ASP, IT prophylaxis is given on day 1 and 8.
- Omit IT methotrexate on Day 1 of cycle 1 if the patient already received IT therapy within 7 days prior to study enrollment as part of a diagnostic lumbar puncture procedure.

***Table 11. Dosages of IT medication for patients with CNS1 status***

| Age (yrs) | Dose of IT Methotrexate (MTX) |
|---|---|
| 1 – 1.99 | 8 mg |
| 2 – 2.99 | 10 mg |
| 3 – 8.99 | 12 mg |
| ≥ 9 | 15 mg |

#### *4.3.3.2 Intrathecal therapy (IT) for BCP-ALL patients with CNS 2 and 3 status*

Note that definitions of CNS involvement are given in Appendix 2.1.3.
- Patients with BCP-ALL who are CNS 2 or 3 prior to enrollment may receive intensified IT therapy with triple IT agents per local standard of care, for example at the doses listed below in ***Table 12***.
- Either prednisolone or hydrocortisone may be used as glucocorticoid per institutional guidelines/local standard of care (depending which steroid is approved for IT use in a given country)

***Table 12: Dosages of IT medication for CNS 2 or 3 status***

| Age | Methotrexate dose (mg) | Cytarabine dose (mg) | Prednisolone dose (mg)* | Hydrocortisone dose (mg)* |
|---|---|---|---|---|
| 1 – 1.99 | 8 | 16 | 8 | 8 |
| 2 – 2.99 | 10 | 20 | 10 | 10 |
| 3 – 8.99 | 12 | 24 | 12 | 12 |
| ≥9 years | 15 | 30 | 12 | 15 |

** use either prednisolone or hydrocortisone according to local standard of care.*

#### *4.3.3.3 Intrathecal (IT) prophylaxis for Stratum 2 patients with other CD22-positive B-cell malignancies*
- Intrathecal therapy for Stratum 2 patients is at the discretion of their treating physician and may be given according to institutional guidelines or as mentioned above.
- All administered IT therapy must be recorded on the CRF (dose and schedule).
- Please note that in Burkitt Lymphoma and Burkitt Leukemia, the definition of CNS1, 2 and 3 status does not apply. In these patients any leukemic cell in the cerebrospinal fluid is considered as CNS-involvement and requires IT treatment until the CSF is cleared. In lymphoblastic lymphoma the same definitions can be applied as in ALL.

### 4.3.4 Disease Evaluations for Cycle 1 (see also schedule of events in Section 7)

#### *4.3.4.1 Stratum 1A, Phase 2 Cohort and Stratum 3 (BCP-ALL patients)*

All patients are to have a bone marrow aspirate/biopsy, lumbar puncture, and CBC with differential and platelet count performed and repeat imaging in case of extramedullary disease (if positive at baseline; using the same method for evaluation – either sonography, CT or MRI-scan), to assess response on Day 22 (+/- 2 days, as needed for scheduling reasons). When clinically indicated, the presence or absence of EMD/lymphomas may need to be confirmed by cytological or histopathological or flow cytometry examination.

Patients with ALL should be classified for response according to the criteria provided in Appendix 2.1.

a. Startum 1A cohort: If the Day 22 (± 2 days) bone marrow aspirate is hypoplastic or M1 but counts have not recovered to ANC ≥ 500/µL and PLTS ≥ 50,000/µL (achieved PLT transfusion independence), obtain a weekly CBC and weekly bone marrow until evidence of count recovery (ANC ≥ 500/µL and PLTS ≥ 50,000/µL) or latest at day **42**; whichever is **first**. Aplasia (ANC<500/µL or platelets <50,000/µL) in absence of persisting leukemia which lasts beyond day 42 and is considered related to InO, will be considered a DLT and the patient needs to be taken off study.

b. Phase 2 cohort AND Stratum 3: If the Day 22 (± 2 days) bone marrow aspirate is hypoplastic or M1 but counts have not recovered to ANC ≥ 500/µL and PLTS ≥ 30,000/µL (achieved PLT transfusion independence), obtain a weekly CBC and weekly bone marrow until evidence of count recovery (ANC ≥ 500/µL and PLTS ≥ 30,000/µL) or latest at day **42**; whichever is **first**.

c. If the patient was M3 at study entry and the Day 22 (± 2 days) marrow is M2 by aspirate (assessed by morphology and/or flow cytometry), then proceed to Cycle 2 regardless of ANC and PLT count after all grade 3 or 4 non-hematologic toxicities have returned to grade 2 or less.

d. If the marrow is M3, if the patient has progression in any extramedullary site, or if the patient is CNS3 at the end of cycle 1 in spite of CNS-directed therapy, discontinue protocol therapy and proceed with alternative treatment.

*4.3.4.2 Stratum 1B/1B-ASP (BCP-ALL patients)*

All patients are to have a bone marrow aspirate/biopsy, lumbar puncture, and CBC with differential and platelet count, and repeat imaging in case extramedullary disease is suspected and/or if positive at baseline using the same method for evaluation – either sonography, CT or MRI-scan should be used to assess response on day 28 (+/- 2 days). When clinically indicated, the presence or absence of EMD may be confirmed by cytological, histopathological or flow cytometry examination.

Response will be classified according to the criteria provided in Appendix 2.1.

a. If the Day 28 (± 2 days) bone marrow is hypoplastic or M1 but counts have not recovered to ANC ≥ 500/µL and PLTS ≥ 30,000/µL (reached PLT transfusion independence), obtain a weekly CBC and weekly bone marrow until evidence of count recovery (ANC ≥ 500/µL and PLTS ≥ 30,000/µL) or latest at day **42**; whichever is **first**. Aplasia (ANC < 500/µL or platelets < 30,000/µL) in absence of persisting leukemia which lasts beyond day 42 and is considered related to InO will be considered a DLT (prolonged cytopenia considered related to impaired hematopoietic reservoir due to previous therapy will not be considered as a DLT)..

b. If the patient was M3 at study entry and the Day 28 (± 2 days) marrow is M2 by aspirate (assessed by morphology and/or flow cytometry) at Day 28 (± 2 days), then proceed to Cycle 2 re-induction regardless of ANC and PLT count after all grade 3 or 4 non-hematologic toxicities have returned to grade 2 or less.

c. If the marrow is M3, if the patient has progression in any extramedullary site, or if the patient is CNS3 at the end of cycle 1 in spite of intensive CNS-directed therapy, discontinue protocol therapy.

*4.3.4.3 Stratum 2 (other CD22 B-cell malignancies)*

All patients should have a CBC with differential and platelet count, bone marrow aspirate or biopsy (if positive at baseline), lumbar puncture (if positive at baseline), and repeated imaging of known sites of disease (if positive at baseline; using the same method for evaluation – either sonography, CT or MRI or PET scan) on day 22 (± 2 days). When clinically indicated the presence or absence of EMD/lymphomas may need to be confirmed by cytological or histopathological or flow cytometry examination.

Note that in case of continued cytopenias, a bone marrow evaluation should be performed, but imaging may be delayed to day 28-42, depending on the bone-marrow evaluation as listed below. Patients with NHL should be classified for response according to the criteria provided in Appendix 2.2.

**Bone marrow evaluation criteria for stratum 2 patients who were bone marrow positive at study entry:**

- If the marrow is hypoplastic or M1, and counts have not recovered to ANC ≥ 500/µL and platelets ≥50,000/µL (reached PLT transfusion independence) obtain a weekly CBC and weekly bone marrow until evidence of count recovery (ANC ≥ 500/µL and PLTS ≥50,000/µL) or latest at day **42**; whichever is **first**. If aplasia lasts longer than day 42 and is considered related to InO, the patient needs to be discontinued from the study.

- If the marrow is M2 by aspirate (assessed by morphology and/or flow cytometry), then proceed to Cycle 2 regardless of ANC and PLT count after all grade 3 or 4 non-hematologic toxicities have returned to grade 2 or less, unless there is progressive disease at other disease sites.

- If the marrow is M3 or has more blasts than the pre-study bone marrow, if the patient demonstrates progression in any other extramedullary site, or if the patient is CNS 3 in spite of CNS-directed therapy at day 22 (± 2 days) discontinue protocol therapy.

4.3.5 Criteria to begin Cycle 2

Height, weight and BSA are to be re-assessed prior to each cycle of therapy, and the dose of InO is to be recalculated at the beginning of each cycle for changes of ≥ 5% in BSA.

*4.3.5.1 Stratum 1A, Phase 2 Cohort and Stratum 3 BCP-ALL patients*

Patients in Stratum 1A and Phase 2 cohort may proceed to Cycle 2 with single-agent InO (***Table 13***) once the following criteria are met:

- Stratum 1A: Patients with an M1 marrow after the first cycle may proceed to Cycle 2 when ANC ≥ 500/µL and platelet count ≥ 50,000/µL (achieved PLT transfusion independence);
- Phase 2 and Stratum 3: Patients with an M1 marrow after the first cycle may proceed to Cycle 2 when ANC ≥ 500/µL and platelet count ≥ 30,000/µL (achieved PLT transfusion independence);
- Patients who were M3 at study entry who are M2 at the end of the first cycle disease evaluation may proceed to Cycle 2 regardless of hematologic criteria;
- Patients who were M2 at study entry must be M1 in order to proceed with cycle 2;
- Patient with an M1 marrow must be CNS1; patients with an M2 marrow may have CNS2;
- Patients with an M1 marrow following the first cycle whose ANC and/or platelet count do not meet criteria to proceed to Cycle 2 by Day 42 will meet criteria for a hematologic DLT if the event is considered to be related to InO (see Section 4.6);
- All grade 3 and 4 non-hematologic toxicities must have resolved to ≤ grade 2 or baseline
- Liver functions (total bilirubin and transaminases) should be checked prior to InO infusion. In case of a >2.5 x ULN elevation in transaminases, or >1.5 x ULN for total bilirubin, delay the dose according to Section 4.4.1. (excluding cases where these aberrations are due to hemolysis or Gilbert's syndrome). There must be no evidence of VOD at the time of starting Cycle 2.

*4.3.5.2 Stratum 1B/1B-ASP BCP-ALL patients*

Patients in Stratum 1B or 1B-ASP may either proceed with a second combination cycle or with further cycle(s) of single-agent InO (see ***Table 13*** for single-agent InO, and ***Table 10*** for combination treatment, and Section 4.3.6.2. for a description), once the following criteria are met:

- Patients with an M1 marrow after the first cycle may proceed to Cycle 2 when ANC ≥ 500/µL and platelet count ≥ 30,000/µL (achieved PLT transfusion independence);
- Patients who were M3 at study entry who are M2 at the end of the first cycle disease evaluation may proceed to Cycle 2 regardless of hematologic criteria;
- Patients who were M2 at study entry must be M1 in order to proceed with cycle 2;
- Patient with an M1 marrow must be CNS1;
- Patients with an M2 marrow may have CNS2;
- Patients with an M1 marrow following the first cycle whose ANC and/or platelet count do not meet criteria to proceed to Cycle 2 by Day 42 will meet criteria for a hematologic DLT if the event is considered to be related to InO (see Section 4.6);
- Liver function tests (total bilirubin and transaminases) should be checked prior to InO infusion. In case an increment of total bilirubin >1.5 x ULN, delay the dose according to ***Table 14*** (excluding cases where these aberrations are due to hemolysis or Gilbert's syndrome) In case of AST and/or ALT increase > 5 x ULN delay the dose according to ***Table 14***. There must be no evidence of VOD at the time of starting Cycle 2.
- All other grade 3 and 4 non-hematologic toxicities must have resolved to ≤ Grade 2 or baseline.

*4.3.5.3 Stratum 2: Other CD22-positive B-cell malignancies*

Patients in stratum 2 may proceed to Cycle 2 (single-agent InO per ***Table 13***) when all of the following criteria are met:

- In the absence of clinical or radiographic progressive disease (see Appendix 2);
- When ANC ≥500/µL and platelet count ≥50,000/µL (achieved PLT transfusion independence);
- All grade 3 or 4 non-hematologic toxicities must have resolved to grade 2 or less;
- Liver functions (total bilirubin and transaminases) should be checked prior to InO infusion. In case of a >2.5 x ULN elevation in transaminases, or >1.5 x ULN for total bilirubin, delay the dose according to Section 4.4.1. (excluding cases where these aberrations are due to hemolysis or Gilbert's syndrome). There must be no evidence of VOD at the time of starting Cycle 2.
- If BM involvement at study entry, they must have a lower BM blast percentage than at baseline;
- Patients must have CNS 1 or 2 status. However, patients with Burkitt lymphoma or Burkitt leukemia should have no evidence of CNS-involvement.

4.3.6 Cycles 2-6

Height, weight and BSA are to be re-assessed prior to each cycle of therapy, and the dose of InO is to be recalculated at the beginning of each cycle for changes of ≥ 5% in BSA.

*4.3.6.1 Cycles 2-6 single-agent InO for Stratum 1A, Phase 2 Cohort, Stratum 2 and Stratum 3*

- The Dose level is assigned at study entry (see ***Table 15*** for dose-levels), and is adjusted to BSA using the Mosteller formula as given in Appendix 4;
- For patients in stratum 3, the RP2D as established in the phase 1 study will be used (1.8 mg/m$^2$ in 3 weekly fractions of 0.8 mg/m$^2$ at day 1, followed by 0.5 mg/m$^2$ at day 8 and at day 15).
- There will be no dose-capping for obese patients/patients with high BSA (see also Section 4.5)
- InO is administered as an IV infusion over 60 minutes (+/-15 min), on Days 1, 8 (±1 day), and 15 (±1 day) as per ***Table 13***. See Section 6.1.5 for further administration guidelines. The Days 8 and 15 doses of InO will remain steady for a given patient throughout study therapy, barring any dose reduction implemented due to toxicity;

- Once a patient achieves CR/CRi/CRp, the dose on day 1 of all subsequent cycles is reduced (per ***Table 15*** in Section 4.5) and the same dose of InO is given every week;
- For patients not yet in CR/CRi/CRp:
  - The same dose of InO is given as in Cycle 1; hence do NOT reduce the dose on day 1
  - From cycle 3 onwards the higher dose on day 1 is omitted and the same dose of InO is given every week in all Cycles as per ***Table 15*** in Section 4.5;
- Record vital signs prior to each infusion and 1 hour (±15 minutes) after the end of each infusion;
- All vital sign measurements should be captured in the eCRF;
- Note that the recommended duration of therapy for those that will be transplanted is 2 cycles, or 3 cycles if the patient is not yet MRD-negative. For patients not proceeding to HSCT, a maximum of 6 cycles of InO can be given.
- Note that there is no specific recommendation for the number of cycles that may be given prior to CAR-T cells therapy. InO is mainly used in such patients as bridging therapy to control disease and cytoreduce patients prior to CAR-T cells therapy. Please note that patients may become MRD negative with InO and that in such patients we recommend to perform a very close watch and wait strategy and to delay CAR-T cells infusion until regeneration of healthy B-cells or minimal residual leukemia, although there are no published guidelines for this.
- It is however suggested to harvest T-cells prior to InO infusion – as there are some reports suggesting that T-cells may be affected by prior InO infusion.
- If the dose of InO is reduced for any reason, it may not be re-escalated.

***Table 13. InO schedule for cycle 2-6 of Single Agent InO***

| **Drug** | **Day 1** | **Day 8 (±1 day)** | **Day 15 (±1 day)** | **Day 28 (± 2 days) to day 42** |
|---|---|---|---|---|
| Anti-emetics (per institutional guidelines) 15 min prior to InO infusion | • | • | • | Disease Evaluation |
| Methylprednisolone 1 mg/kg IV (max 50 mg) 10 min prior to InO infusion | • | • | • | |
| Inotuzumab Ozogamicin in 1 hour (+/- 15 min) IV* | • | • | • | |
| Intrathecal Methotrexate** | • | | | |

** Record vital signs prior and post infusion*
*** Note that the intrathecal therapy may be given together with the disease evaluation lumbar puncture according to the schedule of events rather than on Day 1 of the cycle.*

Intrathecal therapy is given as per section 4.3.3.

*4.3.6.2 Cycles 2-6 for patients in Stratum 1B/1B-ASP*

- Following disease evaluation after Cycle 1, if there are no criteria for discontinuation of treatment (see Section 4.3.5 and 4.3.5.1 for cycle 2, and 4.3.8.1 for subsequent cycles), patients may continue therapy with a 2nd cycle of combination therapy or single-agent InO per the investigator's choice. Alternatively, patients may proceed to off-therapy HSCT or other follow-up therapy.
- When single-agent InO treatment is elected for Cycle 2, patient may receive additional cycles of InO as detailed in ***Table 13***, up to a maximum of 6 cycles total (including 1 or 2 cycle(s) of combination therapy). The dose of single-agent InO will be the RP2D established in 1A Stratum (please discuss this with the Sponsor), with considerations about the loading dose as explained in Section 4.3.6.1. Note that the recommended duration of InO therapy for those that will proceed to HSCT is 2 cycles, or 3 cycles if the patient is not yet MRD-negative after Cycle 2 (to minimize the risk for VOD/SOS).
- When combination therapy is elected for Cycle 2, this will consist of a maximum of one additional cycle of the combination block exactly as given in Cycle 1, as per ***Table 10***. After this 2nd combination block, the patient is allowed to receive additional single-agent cycles of InO but no further combination blocks. The dose of single-agent InO is the RP2D established in Stratum 1A (please discuss with the Sponsor), with considerations about the loading dose as explained in

Section 4.3.6.1. Note that the recommended duration of therapy for those that will be transplanted is 2 cycles of InO, or 3 cycles if the patient is not yet MRD-negative after Cycle 2. For patients not proceeding to HSCT, a maximum of 6 total cycles of InO can be given.

4.3.7 Disease Evaluations for Cycle 2-6 (see also Section 7 schedule of events)

*4.3.7.1 End of cycles 2, 3, 4, 5 and 6 for all BCP-ALL patients*
All patients should have a bone marrow aspirate/biopsy, lumbar puncture, and CBC with differential and platelet count plus radiological evaluation of EMD when present at baseline to assess response between day 28 (+/-2 days)-42 depending on recovery from aplasia.

*4.3.7.2 End of cycles 2, 4 and 6 for other CD22-positive B-cell malignancies (stratum 2) patients*
All patients should have a bone marrow aspirate/biopsy, lumbar puncture, and CBC with differential and platelet count when abnormal at enrollment, and repeated imaging of involved disease sites to assess response between day 28 (+/- 2 days)-42 depending on recovery from aplasia.

For patients with baseline BM-involvement:

- A bone marrow procedure need not be performed if the patient has an absolute blast count greater than or equal to 2,500/$mm^3$ in the peripheral blood **confirmed with morphology AND flow cytometry**, as the patient will be considered a treatment failure and protocol therapy will be discontinued.

  - If the Day 28 (+/- 2 days) bone marrow aspirate is hypoplastic or M1 but counts have not recovered to ANC ≥ 500/µl and PLTS ≥ 50,000/µl (achieved PLT transfusion independence), obtain a weekly CBC with differential and platelets and weekly bone marrow until evidence of count recovery (ANC ≥ 500/µl and PLTS ≥ 50,000/µl) or day **42**; whichever is **first**. In case of persisting aplasia longer than day 42, discontinue study therapy.

- If the marrow is M3, the patient has disease progression or recurrence, new appearance of any extramedullary site or if the patient is CNS 2 or 3 despite intrathecal therapy, discontinue protocol therapy.

4.3.8 Criteria to begin Cycles 3-6

*4.3.8.1 All patients with BCP-ALL*

- Patient with leukemia must have an M1 marrow following the prior cycle, an ANC ≥ 500/µL and platelet count ≥ 30,000/µl (achieved PLT transfusion independence);
- Patients must have CNS 1 status and no evidence of leukemia elsewhere;
- All grade 3 and 4 non-hematologic toxicities must have resolved to grade ≤ 2 or baseline
- Liver functions (total bilirubin and transaminases) should be checked prior to InO infusion. In case of a >2.5 x ULN elevation in AST and/or ALT, or >1.5 x ULN for total bilirubin, delay the dose according to Section 4.4.1. (excluding cases where these aberrations are due to hemolysis or Gilbert's syndrome). There must be no evidence of VOD at the time of starting the cycle.

*4.3.8.2 Patients with other CD22-positive B-cell malignancies*

- Absence of progressive disease;
- ANC ≥ 500/µL and platelet count ≥ 50,000/µl (achieved PLT transfusion independence);
- All grade 3 or 4 non-hematologic toxicities have resolved to ≤ grade 2 or baseline,
- Liver functions (total bilirubin and transaminases) should be checked prior to InO infusion. In case of a >2.5 x ULN elevation in AST and/or ALT, or >1.5 x ULN for total bilirubin, delay the

dose according to Section 4.4.1. (excluding cases where these aberrations are due to hemolysis or Gilbert's syndrome). There must be no evidence of VOD at the time of starting the cycle.
- No evidence of CNS involvement.

## 4.4 Dose discontinuation/delay/modification

### 4.4.1 Cycle 1 in Stratum 1A, 1B/1B-ASP, Stratum 2 and Stratum 3

Patients who experience a DLT in Stratum 1A will discontinue study therapy immediately.

For patients in Stratum 1B or Stratum 2 who experience a DLT, most will discontinue study therapy immediately. However, for those patients who are benefiting from study treatment, a case by case decision will be made by the Sponsor and Steering Committee regarding the possibility to continue the study therapy.

If a patient in Stratum 1A, Stratum 2 and stratum 3 experiences AST/ALT elevation > 2.5 x institutional ULN or bilirubin elevation > 1.5 x institutional ULN, the InO infusion needs to be withheld until the AST/ALT and bilirubin levels have recovered to ≤ 2.5 x ULN or ≤ 1.5 x ULN, respectively, unless hyperbilirubinemia is attributed to hemolysis or Gilbert's disease.

For Stratum 1B, during combination treatment, if a patient experiences AST and/or ALT elevation ≥ Grade 3 or total bilirubin elevation >1.5 x institutional ULN, the InO infusion (day 8 and day 15) and intrathecal methotrexate on day 8 needs to be withheld until the AST and/or ALT and total bilirubin levels have recovered to ≤ Grade 2 or ≤ 1.5 x ULN, respectively, unless hyperbilirubinemia is attributed to hemolysis or Gilbert's disease.

Results for liver tests (bilirubin and AST and/or ALT) at day 8 and day 15 for Stratum 1B/1B-ASP must be reviewed by the treating investigator prior to each InO infusion.

Note that:

- In case Grade 3 or 4 AST and/or ALT elevation lasts longer than 7 days, this is considered a DLT, unless it is considered by the Investigator to be related to leukemic infiltration of the liver or due to another medical complication of therapy such as infection or hepatotoxic concomitant medication(s). In case of a DLT, the patient should come off study therapy and receive no further study drug (see DLT definitions in Section 4.6).

- In case a Grade 3 or 4 AST and/or ALT elevation lasting less than 7 days (and returns to ≤ Grade 2 or baseline), or is considered due to leukemic infiltration, the 2nd and 3rd InO dose may be administered. The interval between the $2^{nd}$ and $3^{rd}$ dose still needs to be a minimum of 6 days.

Although in Stratum 2 and 3, DLTs will not formally be assessed, patients experiencing toxicity which would fulfill the DLT criteria also need to discontinue therapy immediately and need to be reported and discussed with the principle investigator, who will report this to the steering committee for discussion of potential consequences for the dose-escalation in stratum 1A patients.

### 4.4.2 Cycles 2-6 single-agent InO (Stratum 1A, Phase 2, Stratum 2 and Stratum 3)

Criteria to proceed with cycles 2-6 are detailed in Section 4.3.5, 4.3.6 and 4.3.8.

During Cycles 2-6, if the patient experiences severe hematological (not due to persistent/relapsed leukemia) and/or non-hematological toxicities while on treatment, the dose of InO will be delayed for a maximum of 2 weeks and the patient assessed twice weekly until toxicity is resolved to grade 1 or baseline.
If a patient in Stratum 1A, Stratum 2 and Stratum 3 experiences AST/ALT elevation > 2.5 x institutional ULN or bilirubin elevation > 1.5 x institutional ULN, the InO infusion needs to be withheld until the AST/ALT and bilirubin levels have recovered to ≤ 2.5 x ULN or ≤ 1.5 x ULN, respectively, unless hyperbilirubinemia is attributed to hemolysis or Gilbert's disease.

Doses cannot be delayed for more than 42 days after the prior dose without pre-approval from the principle investigator; otherwise the patient must permanently withdraw from the study treatment.

Treatment delay occurring once does not require a change of dose. If delay occurs a second time because of toxicity the dose will be reduced by one dose level following discussion with the PI.

Only one dose reduction is permitted. If toxicity recurs after the dose reduction, treatment will be permanently discontinued.

#### 4.4.3 Cycle 1 Stratum 1B/1B-ASP

Patients who experience a DLT in Stratum 1B will discontinue study therapy immediately. EXCEPTION: For patients who are benefiting from treatment, a case by case decision will be made by the Sponsor and Steering Committee regarding the possibility to continue the study therapy).

In general, in case of excessive toxicity, one or more cytotoxic drugs may need to be dose-reduced. This may include general toxicity such as marrow hypoplasia or infections, but also toxicities that may be attributed to specific drugs, as specified in ***Table 14***. Please, discuss such modifications with the coordinating investigator/sponsor study team.

#### 4.4.4 Cycles 2-6 Stratum 1B/1B-ASP

Treatment may need to be postponed to allow the patient to recover from severe toxicity. The need to delay therapy must be reassessed at least weekly.

Criteria to proceed with cycles 2-6 are detailed in Section 4.3.5.2 and 4.3.8.1. In case modifications are required, please contact the coordinating investigator/sponsor study team.

During Cycle 2-6, if the patient experiences severe toxicities not due to persistent/relapsed leukemia, and are considered at least possibly related to InO, the dose of InO will be delayed for a maximum of 2 weeks. The patient will be assessed frequently until toxicity is resolved to grade 1 or baseline.

In general, in case of excessive toxicity, one or several cytotoxic drugs may need to be dose-reduced, as reported in ***Table 14*** below. Doses cannot be delayed for more than 42 days after the prior dose without pre-approval from the Sponsor principle investigator; otherwise the patient must permanently withdraw from the study treatment.

Treatment delay occurring once does not require a change of dose. If a delay occurs a second time because of InO-related toxicity, the dose will be reduced by one dose level following discussion with coordinating investigator/sponsor study team (see also ***Table 14***).

Only one dose reduction of InO is permitted. If toxicity recurs after the InO dose reduction, protocol treatment will be permanently discontinued and further treatment will be at the discretion of the treating physician.

***Table 14. Dose Modification/Reduction for Toxicity in Stratum 1B/1B-ASP***

| Organ class | Toxicity | InO | Dexamethasone | Vincristine | PEG-Asparaginase | Methotrexate i.t. |
|---|---|---|---|---|---|---|
| **Hepatic toxicity** | **Hyperbilirubinemia grade 2-3** | **Occurrence 1:** Withhold until level ≤1.5xULN[1]<br><br>**Occurrence 2:** reduce by 1 dose level following discussion with PI<br><br>**Note:** In case of confirmed VOD/SOS, no further dosing of InO allowed | No dose interruption or reduction | No dose interruption or reduction | **Occurrence 1:** Withhold until recovery to Grade ≤2 (≤3xULN)<br><br>**Occurrence 2:** Reduce to 2/3 of scheduled dose | No dose interruption or reduction |
| | **Hyperbilirubinemia grade 4** | **Occurrence 1:** Withhold until level ≤1.5xULN[1]<br><br>**Occurrence 2**: permanent discontinuation | **Occurrence 1:** Withhold until recovery to gr ≤2 (<3xULN)<br><br>**Occurrence 2:** Withhold until recovery to gr ≤2 (<3xULN) | **Any occurrence:** Withhold until recovery to gr ≤2 (<3xULN) | **Any occurrence:** Withhold until recovery to Grade ≤2 (<3xULN) | No dose interruption or reduction |
| | **AST and/or ALT increased grade 3** | **Occurrence 1:** Withhold until level ≤Grade 2, unless considered not related[1]<br><br>**Occurrence 2:** Reduce by 1 dose level following discussion with PI, unless considered not related | **Occurrence 1:** Withhold until level ≤5xULN<br><br>**Occurrence 2:** Reduce to 50% of scheduled dose. Resume at 100% dose after recovery to ≤2.5xULN | **Occurrence 1:** No dose interruption or reduction<br><br>**Occurrence 2:** No dose interruption or reduction | **Occurrence 1:** Withhold until level ≤5xULN<br><br>**Occurrence 2:** Reduce to 2/3 of scheduled dose | **Occurrence 1:** Withhold until recovery to Grade ≤2 (≤5xULN)<br><br>**Occurrence 2:** Discuss with PI |
| | **AST and/or ALT increased grade 4** | **Occurrence 1:** Withhold until level ≤ Grade 2 [1]<br><br>**Occurrence 2:** Reduce by 1 dose level following discussion with PI | **Any occurrence:** Withhold until level ≤5xULN . Reduce to 2/3 of scheduled dose.<br><br>Resume at 100% dose after recovery to ≤2.5xULN | **Any occurrence:** Withhold until level 5xULN | **Occurrence 1:** Withhold until level ≤5xULN<br><br>**Occurrence 2:** Reduce to 2/3 of scheduled dose | **Occurrence 1:** Withhold until recovery to Grade ≤2 (≤5xULN)<br><br>**Occurrence 2:** Discuss with PI |
| | **Hy's law cases** | **Any Occurrence:** evaluate pt. as per section 5.6.3; withhold until AST and/or ALT ≤2.5xULN and Total bilirubin ≤1.5xULN | | | | |

| Organ class | Toxicity | InO | Dexamethasone | Vincristine | PEG-Asparaginase | Methotrexate i.t. |
|---|---|---|---|---|---|---|
| **Hematologic toxicity** | **Neutropenia and Thrombocytopenia grade 4** | **Start cycle 2:** refer to Section 4.3.5<br>**Start cycles 3-6:** refer to Section 4.3.8<br>If the start of Cycle 3 or subsequent is delayed later than Day +42, InO will be reduced by 1 dose level following discussion with PI. If hematologic toxicity recurs after the InO dose reduction, protocol treatment will be permanently discontinued.<br><br>Grade 4 hematologic toxicity, lasting longer than Day +42 after Cycle 1 (in the absence of persisting leukemia) is considered a DLT, thus requires permanent discontinuation of treatment. | | | | |
| **Infections** | **Infections grade 3-4** | **Any occurrence:** Withhold until recovery, then dose at 100% after infection is controlled | | | | No dose interruption or reduction |
| **Endocrine** | **Corticosteroid-associated diabetes mellitus** | No dose interruption or reduction, introduce insulin | **Any occurrence:** No dose interruption or reduction, introduce insulin | No dose interruption or reduction, introduce insulin | **Any occurrence:** Withhold until sufficiently controlled with insulin | No dose interruption or reduction |
| **Vascular** | **Thrombosis** | No dose interruption or reduction | Dose reduction/interruption at investigator's discretion | No dose interruption or reduction | Start LMWH, then resume dosing at investigators discretion | No dose interruption or reduction |
| **Electrolytes** | **Hyponatremia grade 4** | No dose interruption or reduction | No dose interruption or reduction | **Any occurrence:** Withhold until electrolytes normalize | No dose interruption or reduction | No dose interruption or reduction |
| **Neurologic** | **Peripheral neuropathy grade 3** | No dose interruption or reduction | No dose interruption or reduction | **Any occurrence:** Consider dose reduction at investigator's discretion | No dose interruption or reduction | No dose interruption or reduction |
| | **Peripheral neuropathy grade 4** | **Any occurrence:** Withhold until recovery; resume dosing at 100% after recovery[1] | **Any occurrence:** Withhold until recovery | No further VCR | **Any occurrence:** Withhold until recovery | No dose interruption or reduction |
| | **CNS disorders (seizures or depressed level of consciousness)** | **Any occurrence:**<br>Withhold until complete recovery, resume dosing at 100% after recovery depending on the identfied cause | | | | |
| **Gastro-intestinal** | **Paralytic ileus** | **Any occurrence:** Withhold until recovery, resume dosing at 100% after recovery[1] | **Any occurrence:** Withhold until recovery, resume dosing at investigator's discretion | No further VCR | **Any occurrence:** Withhold until recovery, resume dosing at investigator's discretion | No dose interruption or reduction |
| | **Pancreatitis mild/severe (PdL criteria)#** | **Any occurrence:** Withhold until complete recovery | | | No further ASP | No dose interruption or reduction |
| **Renal** | **Creatinine increased grade 3-4 and/or signs of tumor lysis syndrome** | Dose reduce/ withhold treatment at investigator's discretion | | | | No dose interruption or reduction |
| **Laboratory other** | **Hypertriglyceridemia grade 3-4** | No dose interruption or reduction | **Any occurrence:** Dose reduction/interruption at investigator's discretion | No dose interruption or reduction | **Any occurrence:** Dose reduction/interruption at investigator's discretion | No dose interruption or reduction |

Abbreviations: C=cycle; conc=concentration; ULN=upper limit of normal; LLN=lower limit of normal; gr=CTCAE grade; VCR=vincristine; ASP=asparaginase;
# Schmiegelow K et al. Consensus definitions of 14 severe acute toxic effects for childhood lymphoblastic leukaemia treatment: a Delphi consensus. Lancet Oncol. 2016;17(6):e231-e239.
[1] Per SPC: reduce by 1 dose level if recovery requires > 14 days; permanent discontinuation if recovery requires > 28 days.

## 4.5 Dose Escalation Schedule

### 4.5.1 Stratum 1A, BCP-ALL patients

Pediatric dose-finding will start in stratum 1A at 80% of the adult RP2D in NHL and ALL patients of 1.8 mg/m$^2$ per cycle, administered in a fractionated fashion. The Cycle 1 Day 1 dose will be higher than subsequent dosages given the high burden of leukemic/lymphoma cells present at the time of relapse and the receptor-mediated clearance in that situation. Hence the pediatric starting dose-level will be 1.4 mg/m$^2$ IV total per cycle, administered as 0.6 mg/m$^2$ on day 1 and 0.4 mg/m$^2$ on days 8 and 15. If dose level 1 is too toxic, the study will de-escalate down to dose level -1 and if needed dose level -2 (refer to ***Table 15*** below).

Dose escalation in stratum 1A will follow a Rolling-6 design to establish the single-agent MTD.[97] The Rolling-6 design is consistent with the rules of the traditional 3+3 escalation design except that enrollment to a dose level can continue until up to 6 patients are treated as long as:

- the observed DLTs among evaluated patients are consistent with further enrollment to that cohort (i.e., 0 or 1 DLTs observed), and
- escalation to the next dose level occurs if at least 3 patients have been treated, and 0 evaluated patients have experienced DLT, and occurrence of DLTs in any patient not yet evaluated for DLT will not violate the design rules of the 3+3.
- In case of 1 DLT in the first 3 patients the cohort will be expanded to a minimum of 6 patients.
- See Section 9.6 for further dose escalation details.

For the starting dose level, a modified version of the Rolling-6 will be used in order to ensure recruitment of at least 2 patients aged ≥ 1 and ≤ 6 years. The first 3 patients enrolled in stratum 1A must be ≥ 6 and ≤ 18 years at the time of study enrollment. After the first 3 patients have been enrolled, at least 2 more patients aged ≥ 1 and ≤ 6 years at the time of study enrolment must be treated at the starting dose level (provided that the rules to de-escalate have not been met).

***Table 15. InO dose-levels for Stratum 1A and 1B Patients (Note that Dose Level 2 is the RP2D for InO single agent only and to be used in Stratum 3)***

| **Inotuzumab Ozogamicin Dose Escalation/De-escalation Schema in mg/m$^2$ IV** | | | | | | | | |
|---|---|---|---|---|---|---|---|---|
| | **Cycle 1*** | | | | **Cycle 2-6#*** | | | |
| **Day** | **1** | **8** | **15** | **Total Dose per Cycle** | **1** | **8** | **15** | **Total Dose per Cycle** |
| **Visit Window in cycle 2-6** | | | | | | **+/- 1 days** | **+/- 1 days** | |
| **Level -2** | 0.4 | 0.2 | 0.2 | **0.8 mg/m$^2$** | 0.2 | 0.2 | 0.2 | **0.6 mg/m$^2$** |
| **Level -1** | 0.5 | 0.3 | 0.3 | **1.1 mg/m$^2$** | 0.3 | 0.3 | 0.3 | **0.9 mg/m$^2$** |
| ***Level 1 (Start)**** | 0.6 | 0.4 | 0.4 | **1.4 mg/m$^2$** | 0.4 | 0.4 | 0.4 | **1.2 mg/m$^2$** |
| **Level 2** | 0.8 | 0.5 | 0.5 | **1.8 mg/m$^2$** | 0.5 | 0.5 | 0.5 | **1.5 mg/m$^2$** |
| **Level 3** | 1.0 | 0.6 | 0.6 | **2.2 mg/m$^2$** | 0.6 | 0.6 | 0.6 | **1.8 mg/m$^2$** |

- Dose de-escalation will not go below Level -2.
- # Following Cycle 1, in patients who have achieved a CR/CRi or CRp, the day 1 dose is decreased slightly due to no loading dose requirement. In patients who have not yet achieved a CR/CRi or CRp after cycle 1, a loading dose similar to cycle 1 will be given in cycle 2, but not in subsequent cycles.

*Note that there will be no dose-capping for obese patients/patients with high BSA.

4.5.2 Stratum 1, Phase 2 Cohort, BCP-ALL patients

During the phase 2 cohort, up to 25 additional evaluable patients will be treated at the MTD/RP2D as identified in Stratum 1A.

4.5.3 Stratum 2 patients (other CD22 positive B-cell malignancies)

Stratum 2 patients will be treated at one dose level below the Stratum 1 dose escalation level, that is, at a dose that has provisionally been determined to be safe. Therefore, stratum 2 enrollment will start only when the first dose-level has been declared safe for BCP-ALL patients. No formal dose-escalation rules will apply. This arm may remain open until accrual for stratum 1 is completed.

4.5.4 Stratum 1B/1B-ASP

The starting dose level in Stratum 1B (InO + modified UKALL-R3 re-induction chemotherapy) will be 2 dose levels below the safe dose established for single agent InO, as detailed in *Table 15*, i.e. 60% of the RP2D of InO monotherapy. The Steering Committee will carefully review the risk-benefit after each cohort before deciding to escalate to the next cohort. The Steering Committee could decide not to escalate to 1.8 mg/m$^2$ or to the MTD, based on the most recent risk-benefit analysis. Therefore, the maximum dose in stratum 1B/1B-ASP might be capped for example at 1.4 mg/m$^2$ in combination with chemotherapy. The InO dose of the combination regimen might not exceed 1.4 mg/m$^2$. Hence dose-escalation steps will include 60, 80 and 100% of single-agent RP2D, in absence of major safety concerns. For example, if DL2 is the RP2D for single-agent InO, we will start Stratum 1B dose-escalation with DL-1 as per *Table 15*. The exact dose level of InO will be communicated to the site at the time of enrollment.

The Cycle 1 Day 1 dose of InO will be higher than subsequent dosages, given the higher burden of leukemic blasts present at the time of relapse, and the receptor-mediated clearance. If the starting dose level is too toxic, the combination regimen may be considered not feasible and enrollment in Stratum 1B may close. Alternatively, an amendement may be necessary implemented testing InO in combination with a modified ALL reinduction chemotherapy regimen.
Protocol amendment v4.0 was implemented to modify the backbone chemotherapy regimen reducing the dexamethasone dose to 10 mg/m$^2$/day. After the amendment, dose escalation will start from DL-2 or DL-1, depending on the safety of dexamethasone 20 mg/m$^2$/day with InO at 0.8 mg/m$^2$/cycle (DL-2) (refer to ***Table 15***).

Initially, InO will be combined with vincristine and dexamethasone only (plus IT chemotherapy) (Stratum 1B). After amendment v4.0 the same combination will be tested with a lower dose of dexamethasone. Once the recommended dose of InO in this combination (1B) is established, a subsequent cohort will be treated with InO combined with vincristine, dexamethasone and PEG-asparaginase to study the safety profile of this combination (Stratum 1B-ASP).

Dose escalation in Stratum 1B will follow a Rolling-6 design, which is consistent with the rules of the traditional 3+3 escalation design except that enrollment to a dose level can continue until up to 6 patients are treated as long as:

- the observed DLTs among evaluated patients are consistent with further enrollment to that cohort (i.e., 0 or 1 DLTs observed), and
- escalation to the next dose level occurs if at least 3 patients have been treated, and 0 evaluated patients have experienced DLT, and occurrence of DLTs in any patient not yet evaluated for DLT will not violate the design rules of the 3+3.
- In case of 1 DLT in the first 3 patients, the cohort will be expanded to a minimum of 6 patients.
- See Section 9.7 for further dose escalation details.
- When pegylated asparaginase is added (Stratum 1B-ASP), this cohort will enroll at least 6 patients as long as the observed DLTs among evaluable patients are consistent with further enrollment (i.e., 0 or 1 DLTs observed).
- In order to establish the RP2D for this cohort (1B-ASP), the proposed RP2D level will be expanded so that a minimum of 10 patients (total) will be evaluable at this dose.

- In order to allow in the study patients who are not eligible for Stratum 1B-ASP, the RP2D level established in Stratum 1B will be expanded by up to 4 patients.

Please note that patients in whom asparaginase levels are monitored per local standard of care, no ASP dose adjustments will be implemented based on these drug levels.

In case of silent inactivation of asparaginase, the patient will be considered non-evaluable for Stratum 1B-ASP and will be replaced.

4.5.5. The InO dosing plan for Stratum 3

Patients will be treated at the RP2D of 1.8 mg/m$^2$ in cycle 1 as identified in Stratum 1A and the Phase 2 single agent cohorts.

## 4.6 Dose-limiting Toxicity (DLT)

Toxicity will be graded using the CTCAE criteria, v4.03. The CTCAE provides descriptive terminology and a grading scale for each adverse event listed. A copy of the CTCAE can be downloaded from the CTEP home page **(**http://ctep.cancer.gov**).**

4.6.1 Definition of Dose-limiting Toxicity for Stratum 1A
DLT will be evaluated during Cycle 1 only and only in stratum 1A patients. All DLTs will be evaluated by the trial steering committee and in regular safety teleconferences with the investigators, including the reporting physician.

Dose-limiting toxicity (DLT) will be defined as the occurrence of the following toxicities anytime during the first cycle of therapy in stratum 1A that is considered related to InO:

- Any Grade 5 toxicity

- Neutropenia (defined as absolute neutrophil count [ANC] < 500/µL) and/or thrombocytopenia (defined as a platelet count < 50,000/µL) will be considered a DLT only when lasting longer than Day +42, but only in the absence of persisting leukemia.

- Grade 3 or 4 Non-Hematologic Toxicities persisting for > 48 hours without resolution to ≤ grade 2 assuming the event is considered related to InO, excluding the following:
  - Alopecia, anorexia or nausea.
  - Grade 3 or 4 mucositis that resolves to ≤ grade 2 within 14 days.
  - Grade 3 or 4 vomiting or diarrhea that resolves to ≤ grade 2 within 7 days.
  - Grade 3 or 4 fever with neutropenia, with or without infection.
  - Grade 3 or 4 infection.
  - Grade 3 or 4 electrolyte abnormalities not associated with clinical sequelae.
  - Grade 3 or 4 hypotension explained by sepsis.
  - Grade 3 injection site reaction.
  - Grade 3 or 4 elevation of hepatic transaminases (ALT/SGPT and/or AST/SGOT) that returns to ≤ grade 2 or baseline within 7 days.
  - Grade 3 or 4 elevation of hepatic transaminases (ALT/SGPT and/or AST/SGOT) if considered by the investigator to be related to: 1) leukemic infiltration of the liver or 2) due to another medical complication of therapy such as infection or hepatotoxic concomitant medication(s).
  - Grade 3 or 4 elevation in amylase or lipase that is asymptomatic and returns to ≤ Grade 2 within 14 days.

- Grade 3 bilirubin elevation that is asymptomatic and returns to ≤ grade 2 within 7 days and is not related to liver pathology (eg mostly indirect bilirubin, Gilbert's syndrome).
- Grade 3 hemorrhage/bleeding.
- Grade 3 or 4 allergic event.
- Any other toxicity considered medically relevant by the investigator may be considered a DLT after agreement by the sponsor.

The dose escalation process to determine the MTD is guided by the safety evaluation during Cycle 1 in BCP-ALL stratum 1A patients only. However, at the end of dose escalation phase, a longitudinal dose-time-toxicity model proposed in Paoletti et al (2015)[98] will be applied to help determine the RP2D by integrating data from all cycles.[98] The model will also be re-run at the end of the single-agent phase 2 cohort to further refine the dose. In addition, toxicities occurring in stratum 2, and cumulative toxicities observed in subsequent cycles could also be considered for the dose escalation process, dose definition and determination of the RP2D, upon agreement between the sponsor and the Investigators.

All DLTs require continued monitoring and follow-up reporting until they resolve to grade 1 or less, until the patient begins another treatment regimen or until the toxicity is determined to be unresolvable or stabilized.

4.6.2 Definition of Dose-limiting Toxicity for Stratum 1B/1B-ASP

DLTs will be evaluated during cycle 1 only. All DLTs will be evaluated by the trial steering committee and in regular safety teleconferences with the investigators, preferably including the reporting physician.

DLT will be defined as the occurrence of the following toxicities in Stratum 1B that are possibly, probably or definitely related to InO:

- Any Grade 5 toxicity, which is not clearly attributable to well-known chemotherapy-related toxicity (either from vincristine, dexamethasone, intrathecal treatment, plus pegylated asparaginase in patients enrolled in Stratum 1B-ASP), and is thought to be related to or worsened by the addition of InO.

- ANC < 500/µL and/or thrombocytopenia (platelet count < 30,000/ µL and/or persistent PLT transfusion dependence) will be considered a DLT only when lasting longer than Day +42, and only in the absence of persisting leukemia. If the prolonged aplasia in not considered related to InO (e.g. related to bone marrow exhaustion due to previous therapy), this event will not be considered a DLT in agreement with the Sponsor and Steering Committee.

- Grade 3 or 4 Non-Hematologic Toxicities persisting for > 48 hours without resolution to ≤ grade 2 assuming the event is considered related to InO, excluding the following:
    - Alopecia, anorexia or nausea.
    - Grade 3 or 4 mucositis that resolves to ≤ grade 2 within 14 days.
    - Grade 3 or 4 vomiting or diarrhea that resolves to ≤ grade 2 within 7 days.
    - Grade 3 or 4 fever with neutropenia, with or without infection.
    - Grade 3 or 4 infection.
    - Grade 3 or 4 electrolyte abnormalities not associated with clinical sequelae.
    - Grade 3 or 4 hypotension explained by sepsis.
    - Grade 3 injection site reaction.
    - Grade 3 or 4 elevation in amylase or lipase that is asymptomatic and returns to ≤ Grade 2 within 14 days
    - Grade 3 bilirubin elevation that is asymptomatic and returns to ≤ grade 2 within 7 days and is not related to liver pathology (e.g. mostly indirect bilirubin, Gilbert's syndrome).
    - Grade 3 or 4 elevation of hepatic transaminases (ALT/SGPT and/or AST/SGOT) that returns to ≤ grade 2 or baseline within 7 days.
    - Grade 3 or 4 elevation of hepatic transaminases (ALT/SGPT and/or AST/SGOT) that are

considered by the investigator to be related to: 1) leukemic infiltration of the liver or 2) due to another medical complication of therapy, such as infection or hepatotoxic concomitant medication(s).

- Grade 3 hemorrhage/bleeding.
- Grade 3 or 4 allergic event.
- Specific toxicities known to be associated with any of the standard reinduction chemotherapy agent, as recently defined by Schmiegelow and colleagues[1], including but not limited to:
    - Asparaginase-induced pancreatitis
    - Allergic reaction/Hypersensitivity to asparaginase
    - Dexamethasone and/or asparaginase-induced thrombosis
    - Dexamethasone and/or asparaginase-induced diabetes mellitus
    - Dexamethasone-induced osteonecrosis
    - Dexamethasone-induced behavorial changes
    - Cushingoid appearance
    - Hyperlipidemia
    - Arterial hypertension and/or posterior reversible encephalopathy syndrome
    - Methotrexate-induced seizures or neurological symptoms
    - Pneumocystis jirovecci pneumonia.
    - Peripheral neuropathy and or constipation thought to be related to vincristine
- Any other toxicity considered medically relevant by the investigator may be considered a DLT after agreement by the sponsor and steering committee.

All DLTs require continued monitoring and follow-up reporting until they resolve to grade 1 or less, until the patient begins another treatment regimen, or until the toxicity is determined to be unresolvable or stabilized.

# 5.0 SUPPORTIVE CARE

Best supportive care and treatment will be given as appropriate to each patient (anti-emetics, antibiotics, transfusions, oxygen therapy, nutritional support, etc.). Patients may experience profound myelosuppression and immune suppression during this time. Caregivers must also be made aware that patients may experience very rapid clinical deterioration. This suggests the need for a supportive care network that can recognize and respond to sudden changes in a patient's condition. Aggressive supportive care improves outcome. The following guidelines are intended to give general heath direction for optimal patient care and to encourage uniformity in the treatment of this patient population.

## 5.1 Blood Products

Investigators should follow institutional guidelines regarding administration of blood products.

## 5.2 Infection Control and Prophylaxis

### 5.2.1 Pneumocystis jirovecci prophylaxis

Pneumocystis jirovecci prophylaxis is required, with agents and dosing provided according to institutional guidelines.

### 5.2.2 Antibacterial prophylaxis

It is recommended that consideration be given to anti-bacterial prophylaxis during periods of neutropenia (ANC <0.75 × $10^9$/L). Treating physicians may follow their institutional guidelines.

5.2.3 Herpes simplex virus (HSV) prophylaxis*:* Patients with history of HSV or positive antibodies may receive prophylaxis according to institutional guidelines.

### 5.2.4 Fever and Neutropenia

All patients with a fever ≥38.5°C on a single occasion, or >38°C on 2 occasions within 12 hours, and an ANC <500×$10^9$/L are to be treated immediately with intravenous broad-spectrum antibiotics after obtaining appropriate cultures. It is strongly recommended that such patients be hospitalized. The specific choice of antibiotics to be used in empiric treatment of febrile neutropenia is dependent on each institution's experience regarding the type of infecting organisms, and their antibiotic sensitivity patterns. Duration of therapy should be determined by site of infection, culture results, and response to treatment. Antifungal treatment is to be strongly considered for the persistence of fever, or emergence of a new fever in neutropenic patients. Surveillance radiographic imaging surveillance for sites of infection should also be performed as clinically indicated or per institutional guidelines. When severe mucositis or a sepsis syndrome is present in patients with febrile neutropenia, or a patient has a history of alpha hemolytic streptococcal infection, consider inclusion of vancomycin in the empiric antibiotic regimen.

### 5.2.5 Anti-fungal prophylaxis

Anti-fungal prophylaxis is recommended, and may be given according to institutional guidelines and for instance include therapy with IV caspofungin, micafungin or amphotericin (1mg/kg IV thrice weekly). The patient should remain on anti-fungal prophylaxis until the ANC has reached a nadir and has subsequently reached at least 500x$10^9$/l and is rising, and the patient is afebrile and clinically stable.

**Note: prophylactic azole antifungals are not preferred until 7 days after the final dose of InO, because of the risk of liver function abnormalities both with InO as well as with azoles.** An alternative may be prophylactic liposomal amphotericin-B at dosages according to institutional guidelines.

### 5.2.6 Mucositis

Mucositis should be managed with IV hydration and hyperalimentation if indicated, effective analgesia, broad-spectrum gram-positive and gram-negative antibiotic therapy, and empiric antiviral and antifungal therapy as clinically indicated.

## 5.3 Anti-emetic Protection

Anti-emetics should be given to all patients as needed and per institutional guidelines.

## 5.4 Tumor Lysis

InO should only be administered under the supervision of physicians who are experienced in the supportive care of patients who may experience tumor lysis syndrome (TLS). TLS events usually occur within 2 days post-infusion, but may cause urgent problems in case of a rapid drop in WBC. Patients should be monitored for the potential risk of tumor lysis syndrome. Patients who experience TLS should receive appropriate treatment including IV hyperhydration, electrolyte correction, and allopurinol or rasburicase to prevent renal dysfunction.

## 5.5 Concurrent Therapy

In general, the use of any concomitant medication/therapies deemed necessary for the care of the patient are allowed, including drugs given prophylactically (e.g. anti-emetics, antibiotics) with the following exceptions:

1. No other investigational therapy should be given to patients.
2. No anticancer agents other than the study medications administered as part of this study protocol should be given to patients. If such agents are required for a patient then the patient must first be withdrawn from the study treatment.
3. Hydroxyurea, steroids or 6-mercaptopurine may be given per institutional guidelines prior to initiating protocol therapy up to 48 hours prior to the first dose of InO to help manage elevated WBC.
4. The use of dexamethasone as an anti-emetic is prohibited, however premedication with methylprednisolone (or dexamethasone in Stratum 1B/1B-ASP) is allowed to prevent infusion-related side-effects, as well as dexamethasone as an anti-leukemic agent in Stratum 1B/1B-ASP patients according to the dose and schedule as provided in the protocol.
5. Leukocyte growth factors (e.g. G-CSF and GM-CSF) are not to be administered routinely but may be used per institutional policy if the patient has neutropenic sepsis or other serious infection. Leukocyte growth factor administration must be documented in the CRFs.
6. Patients in Stratum 1B-ASP who experienced prior asparaginase-related thrombosis must be treated with prophylactic anticoagulants such as low molecular weight heparin, per institutional guidelines.
7. Patients in Stratum 1B/1B-ASP who experience steroid-related diabetes mellitus should be treated with insulin, per institutional guidelines.
8. No dose-adjustments will be made in the asparginase dose based on measuring asparaginase levels/activity.
9. It is suggested to harvest T-cells prior to InO infusion – as there are some reports suggesting that T-cells may be affected by prior InO infusion.[5] Indeed, a small study collected data from 9 patients that received InO as bridging therapy before CAR-T cells infusion, among 67 subjects that in total underwent CAR-T cells therapy. Suboptimal expansion was observed in the InO group, and similar conclusions were obtained in the 15 patients that received prior Blimatumumab.95 Other investigators did not report issues regarding harvesting T cells post InO treatment.[96]. Patients in stratum 3 are allowed to undergo T-cells harvesting during screening for a planned CAR-T cells therapy.
10. Please note that patients may become MRD negative with InO and that in such patients we recommend to perform a very close watch and wait strategy and to delay CAR-T cells infusion until regeneration of healthy B-cells or minimal residual leukemia.

## 5.6 Hepatic Toxicity

- Patients should be carefully monitored for any signs/symptoms of Veno-Occlusive Disease (VOD)/Sinusoidal Obstruction Syndrome (SOS)[99]; these can include rapid weight gain, right upper quadrant pain, hepatomegaly, ascites and elevations in bilirubin, as well as low platelets and refractoriness for platelet transfusions. See Appendix 3 for diagnostic criteria of VOD/SOS. Patients with VOD/SOS are required to permanently discontinue study treatment with InO.

- When evaluating liver toxicity, the radiologist should be informed of the potential for hepatic vascular disease. When VOD is in the differential diagnosis, a right upper quadrant ultrasound with color flow doppler should be performed.

- Defibrotide may be used in the setting of severe VOD, as per SPC and/or institutional guidelines[100,101]

5.6.1 Considerations for patients who proceed to Stem Cell Transplant After Treatment with InO.

- Please note that the HSCT procedure itself is not part of this protocol but performed as standard of care based on investigator discretion after the patient underwent the EOT visit.

- However, we do aim at collecting certain data on the HSCT procedure and especially regarding particular safety concerns such as VOD/SOS, as described in Section 7.12. Because it is known from studies in adults that InO re-induction prior to HSCT may increase the risk of VOD/SOS, some recommendations are provided to reduce that risk, which are given below. The final choice of the conditioning regimen is however left to the discretion of the treating physician.

- For patients planning to receive an allogeneic HSCT, it is recommended that treatment with InO be limited to the fewest number (up to maximum 3) of cycles required to achieve a complete remission (CR)/complete remission with incomplete hematologic recovery (CRi), or incomplete platelet regeneration (CRp), and preferably an MRD level below 1 x $10^{-4}$ (complete MRD response)[102]. Response definitions are given in Appendix 2.1.

- It is recommended that patients are assessed for risk factors for VOD/SOS and that these risk factors are addressed prior to start of conditioning regimen. Healthcare providers should use their clinical judgment to determine the most appropriate cycle of prophylactic treatment before the start of conditioning therapy according to institutional guidelines, including for instance:
    - Prophylactic ursodeoxycholic acid at 12-15 mg/kg/day, beginning 2 weeks before the start of conditioning therapy[103,104]
    - Prophylactic defibrotide as advised in the literature at a dose of 6.25 mg/kg given four times daily for 14 days, for children undergoing allo-HSCT with the following risk factors: pre-existing hepatic disease, second myeloablative transplant, allogeneic transplant for leukemia beyond second relapse, conditioning with busulfan-containing regimens[105].
    - Defibrotide should be instigated promptly if VOD is suspected as treatment for VOD at a dose of 6.25 mg/kg given intravenously over 2 hours four times per day. The optimal duration of defibrotide in the treatment of VOD is unknown. Defibrotide should be continued for a minimum of 7 days and until the liver function is clearly improving.

- The least hepatotoxic conditioning regimen should be used, specifically avoiding regimens that contain 2 alkylating agents (e.g. busulfan, cyclophosphamide, melphalan, thiotepa) and/or that combine an alkylating agent with *higher dose* TBI (defined as >12 Gy).

- The following conditioning regimens were proven to be effective with acceptable liver toxicity in pediatric ALL, and may be considered:
    - TBI 12 Gray with etoposide[106]
    - A treosulfan-based regimen[107]
    - Busulfan (PK targeted) with fludarabine and clofarabine[108]

  If a busulfan-containing conditioning regimen is used, consideration should be given to pharmacokinetically dosed busulfan[109]

- Whenever possible, the concomitant use of hepatotoxic drugs peri-transplant should be avoided.

- If a patient will proceed to HSCT under the care of different physicians, these recommendations should be reviewed with the new treating physicians, and the reporting responsibilities should be discussed and agreed with the transplant center.

5.6.2 Considerations for patients who proceed to CAR-T cells therapy after treatment with InO.

- Should be noted that the CAR-T cells therapy procedure itself is not part of this protocol but performed as standard/experimental care based on investigator discretion after the patient underwent the EOT visit.
- There is no specific recommendation for the number of cycles that may be given prior to CAR-T cells therapy. InO is mainly used in such patients as bridging therapy to control disease and cytoreduce patients prior to CAR-T cells therapy. The final choice of the timing to perform the CAR-T cells therapy procedure is however left to the discretion of the treating physician.
- Please note that patients may become MRD negative with InO and that in such patients we recommend to perform a very close watch and wait strategy and to delay CAR-T cells infusion until regeneration of healthy B-cells or minimal residual leukemia, although there are no published guidelines for this, apart from anecdotal evidence. It is however advised to harvest T-cells prior to InO infusion – as there are some reports suggesting that T-cells may be affected by prior InO infusion and suboptimal expansion might occur as reported in section 4.1.[5,95,96]
- However, we do aim at collecting certain data on the CAR-T cells therapy after InO and especially regarding the absolute number of peripheral blood CD19+ B-Cells, CD4+/CD8+ T-cells and serum immunoglobulin levels (IgM and IgG) after EOT, and the percentage of patients responding to InO (ORR) without adequate recovery of CD19-positive B-cells or immunoglobulins (both below LLN for age).

5.6.3 Potential Hy’s Law Cases (drug-induced liver injury)

Abnormal values in aspartate transaminase (AST) and/or alanine transaminase (ALT) concurrent with abnormal elevations in total bilirubin that meet the criteria outlined below in the absence of other causes of liver injury are considered potential cases of drug-induced liver injury (potential Hy’s Law cases) and should always be considered important medical events.

The threshold of laboratory abnormalities for a potential case of drug-induced liver injury depends on the patient’s individual baseline values and underlying conditions. Patients who present with the following laboratory abnormalities should be evaluated further to definitively determine the etiology of the abnormal laboratory values:

- Patients with AST or ALT and total bilirubin baseline values within the normal range, who subsequently present with AST or ALT >3 x ULN concurrent with a total bilirubin >2 x ULN, with no evidence of hemolysis or cholestasis, and an alkaline phosphatase <2 x ULN or not available.
- For patients with pre-existing ALT **OR** AST **OR** total total bilirubin values above the ULN, the following threshold values are used in the definition mentioned above:
    - Pre-existing AST or ALT baseline values above the normal range: AST or ALT values >2 x the baseline values **AND** >3 × ULN **OR** >8 × ULN (whichever is smaller).
    - Pre-existing values of total bilirubin above the normal range: total bilirubin level increased from baseline value by an amount of at least 1 × ULN **OR** if the value reaches >3 × ULN (whichever is smaller).

The patient should return to the investigational site and be evaluated as soon as possible, preferably within 48 hours from awareness of the abnormal results. This evaluation should include laboratory tests, detailed history and physical assessment. The possibility of hepatic neoplasia (primary or secondary) should be considered. In addition to repeating measurements of AST and ALT, laboratory tests should include albumin, creatine kinase, total bilirubin, direct and indirect bilirubin, gamma-glutamyl transferase,

prothrombin time (PT) international normalized ratio (INR), and alkaline phosphatase. A detailed history, including relevant information, such as review of ethanol, acetaminophen, recreational drug and supplement consumption, family history, occupational exposure, travel history, history of contact with a jaundiced patient, surgery, blood transfusion, history of liver or allergic disease, and work exposure, should be collected. Further testing for acute hepatitis A, B, or C infection and liver imaging (eg, biliary tract) may be warranted. The cases confirmed on repeat testing as meeting the laboratory criteria defined above, with no other cause for liver function test (LFT) abnormalities identified at the time should be considered potential Hy's Law cases irrespective of availability of all the results of the investigations performed to determine etiology of the abnormal LFTs. Rises in AST and/or ALT and TBili separated by more than a few weeks should be assessed individually based on clinical judgment; any case where uncertainty remains as to whether it represents a potential Hy's law case should be reviewed with the Principle Investigator, representing the Sponsor. **Such potential Hy's Law cases should be reported as SAEs.**

## 6.0 DRUG INFORMATION

### 6.1 Inotuzumab Ozogamicin

Please refer to the Summary of Product Characteristics for full details.

6.1.1 Nomenclature and Molecular Structure
Chemical Name – Conjugate of humanized anti-CD22 monoclonal IgG4 antibody G544 with N acetyl-gamma calicheamicin dimethlyhydrazide (via AcBut linker).

6.1.2 Physical and Chemical Properties
InO (PF-05208773; CMC-544) is an intravenous (IV) chemotherapy agent composed of an anti-CD22 antibody linked to calicheamicin, a potent cytotoxic antitumor antibiotic. The targeting agent in InO is a humanized monoclonal antibody (G544) which is an immunoglobulin type G, subtype 4 that specifically recognizes human CD22. As an IgG4 isotype antibody G544 is not expected to mediate effector functions such as complement-dependent cytotoxicity or antibody-dependent cellular cytotoxicity. CD22 is expressed on both normal and malignant cells of the mature B-lymphocyte lineage but not on lymphocyte precursor cells or memory B cells. Consequently, the impact of treatment with InO on long-term immune function is expected to be minimal. Antibody-targeted chemotherapy is predicated on the specific binding of a monoclonal antibody-drug (mAb-drug) conjugate to the relevant tumor antigen followed by internalization of the antigen-mAb-drug complex allowing delivery of the cytotoxic drug within the tumor cell. Calicheamicins are DNA minor-groove-binding natural cytotoxic products that cause cell death by inducing double-strand DNA breaks. They are significantly more potent than conventional cytotoxic chemotherapeutic agents. Targeted delivery of cytotoxic agents such as calicheamicin is expected to not only maximize antitumor efficacy, but also to significantly reduce exposure of normal tissues to calicheamicin, thereby improving the therapeutic index of the antibody-targeted chemotherapy agent.

6.1.3 Toxicity
As of 01 September 2014, a total of 845 cancer patients had been treated with InO during the Pfizer sponsored development program. 173 patients were treated with single-agent InO, 368 patients were treated with InO in combination with rituximab, and 103 patients were treated with InO in combination with rituximab plus chemotherapy. In studies in patients with relapsed or refractory ALL, 201 patients were treated with single-agent InO.

The table below summarizes the adverse drug reactions (ADRs) reasonably determined to be associated with therapy occurring in NHL and ALL patients receiving InO as monotherapy, AE data from patients with relapsed or refractory ALL treated with single agent InO in Study B1931022 are not presented since this an ongoing pivotal study.

***Table 16. Side-effects related to single agent InO in other studies***

Percentage of adult patients with relapsed or refractory ALL reporting adverse drug reactions (all grades) after receiving single-agent InO, all causality.

| System Organ Class | Very Common<br>≥1/10 | Common<br>≥1/100 to <1/10 |
|---|---|---|
| Infections and infestations | Infection[a*] (48%) | |
| Blood and lymphatic system disorders | Febrile neutropenia (26%)<br>Neutropenia[b] (49%)<br>Thrombocytopenia[c] (51%)<br>Leukopenia[d] (35%)<br>Lymphopenia[e] (18%)<br>Anaemia[f] (36%) | Pancytopenia[g] (2%) |
| Metabolism and | Decreased appetite[‡] (12%) | Tumour lysis syndrome (2%) |

| | | |
|---|---|---|
| Nutrition Disorders | | Hyperuricaemia‡ (4%) |
| Nervous system disorders | Headache[h] (28%) | |
| Vascular disorders | Haemorrhage[i*] (33%) | |
| Gastrointestinal disorders | Abdominal pain[j] (23%)<br>Vomiting (15%)<br>Diarrhoea‡ (17%)<br>Nausea (31%)<br>Stomatitis[k] (13%)<br>Constipation‡ (17%) | Ascites (4%)<br>Abdominal distention‡‡ (6%) |
| Hepatobiliary disorders | Hyperbilirubinaemia‡ (21%) | Venoocclusive liver disease (sinusoidal obstruction syndrome (3% [pre-HSCT][l]) |
| General disorders and administration site conditions | Pyrexia (32%)<br>Fatigue[m] (35%)<br>Chills‡ (11%) | |
| Investigations | Transaminases increased[n] (26%)<br>Blood alkaline phosphatase increased‡ (13%)<br>Gamma-glutamyltransferase increased‡ (21%) | Lipase increased‡ (9%)<br>Electrocardiogram QT prolonged‡ (1%)<br>Amylase increased‡ (5%) |
| Injury, poisoning and procedural complications | | Infusion-related reaction (2%) |

Data cutoff: 01 September 2016
Adverse reactions included treatment-emergent, all-causality events that commenced on or after Cycle 1 Day 1 but within 42 days after the last dose of Inotuzumab Ozogamicin but prior to the start of a new anti-cancer treatment (including HSCT).

a. Infection includes any reported preferred terms for Inotuzumab Ozogamicin retrieved in the System Organ Class Infections and infestations, and includes fatal events.
b. Neutropenia includes the following reported preferred terms: Neutropenia and Neutrophil count decreased†‡.
c. Thrombocytopenia includes the following reported preferred terms: Platelet count decreased‡ and Thrombocytopenia.
d. Leukopenia includes the following reported preferred terms: Leukopenia, Monocytopenia†‡, and White blood cell count decreased‡.
e. Lymphopenia includes the following reported preferred terms: B-lymphocyte count decreased‡, Lymphopenia‡ and Lymphocyte count decreased‡.
f. Anaemia includes the following reported preferred terms: Anaemia and Haemoglobin decreased‡.
g. Pancytopenia includes the following reported preferred terms: Bone marrow failure‡, Febrile bone marrow aplasia†‡, and Pancytopenia‡.
h. Headache includes the following preferred terms: Headache, Migraine†‡, and Sinus headache‡.
i. Haemorrhage includes any reported preferred terms for Inotuzumab Ozogamicin retrieved in the Standard Medical Dictionary for Regulatory Activities (MedDRA) Query (narrow) for Haemorrhage terms (excluding laboratory terms).
j. Abdominal pain includes the following reported preferred terms: Abdominal pain, Abdominal pain lower, Abdominal pain upper, Abdominal tenderness‡, Oesophageal pain†‡, and Hepatic pain†‡.
k. Stomatitis includes the following reported preferred terms: Aphthous ulcer‡, Mucosal inflammation‡, Mouth ulceration†‡, Oral pain‡, Oropharyngeal pain‡, and Stomatitis.
l. Includes 1 additional patient with Venoocclusive liver disease that occurred at Day 56 with no intervening HSCT
m. Fatigue includes the following reported preferred terms: Asthenia and Fatigue.
n. Transaminase increased includes the following reported preferred terms: Aspartate aminotransferase increased‡, Alanine aminotransferase increased‡, Hepatocellular injury†‡, and Hypertransaminasaemia†‡.
o. Infusion-related reaction includes the following reported preferred terms: Infusion-related reaction‡ and Hypersensitivity†‡.

* Events with fatal outcomes.
† Singular event
‡ Nonserious events.

6.1.4 Formulation and Packaging

InO is provided as a lyophilized, unpreserved white to off-white powder for solution for injection in an amber vial at 1 mg/vial for intravenous injection. For information regarding formulation, admixture, and administration of InO, refer to Section 6.1.5 and to the Dosage and Administration Instructions (DAI), provided separately. For information regarding formulation, admixture, and administration of the other study drugs, refer to the most recent version of the local product labeling (product package insert).

6.1.5 Guidelines for Administration

InO Powder for Solution, 1 mg/vial for Intravenous Administration is supplied as a sterile white lyophile, essentially free of visible foreign matter, packaged in a 20 mL amber glass vial with a coated rubber lyo stopper and aluminum overseal.

Each vial must be reconstituted with 4 mL sterile water for injection. Prior to reconstitution, drug vials should be allowed to warm to room temperature over 5 minutes. InO is light sensitive and must be protected from light during preparation and infusion. The final concentration of InO in the reconstituted drug product is 0.25 mg/mL. Reconstituted drug must be a clear to slightly cloudy, colorless solution, essentially free of visible foreign matter. Use reconstituted solution immediately or after being stored in a refrigerator (2°C-8°C) for up to 4 hours. Do not freeze.

Reconstituted InO is diluted in 0.9% Sodium Chloride Injection (Normal Saline) prior to IV infusion. Use diluted solution immediately or after being stored at room temperature (20°C-25°C) or in a refrigerator (2°C-8°C). If the diluted solution is stored in a refrigerator (2°C-8°C), bring it to room temperature (20°C-25°C) for approximately 1 hour prior to administration. Protect from light.

The maximum time from reconstitution through the end of administration should be ≤ 8 hours, with ≤ 4 hours between reconstitution and dilution. During dilution please protect from light. An infusion container made of polyvinyl chloride (PVC) (di(2-ethylhexyl)phthalate [DEHP]- or non-DEHP-64 containing), polyolefin (polypropylene and/or polyethylene), or ethylene vinyl acetate (EVA) is recommended. Do not freeze.

The concentrations verified for administration by syringe are 0.025 mg/mL to 0.1 mg/mL with volume limits of 2 mL to 50 mL, concentrations verified for administration in 50 mL IV bags are 0.01 mg/mL to 0.1 mg/mL.

The dose will be administered intravenously over the cycle of approximately 1 hour (60 min +/- 15 minutes) utilizing an infusion pump or syringe pump. InO for injection should never be administered as an IV push or bolus. Prior to reconstitution, drug vials should be allowed to warm to room temperature over 5 minutes. InO is light sensitive and must be protected from light during preparation and infusion. IV lines may only be exposed to light for the approximate 1 hour slated for infusion; if delays in start of administration occur, or if infusions may take longer than the suggested 1 hour, lines must be protected from light. Infusion lines made of PVC (DEHP or non-DEHP-containing), polyolefin (polypropylene and/or polyethylene), or polybutadiene are recommended.

**Special attention for infusion of low volume doses:**

- Please note that the volume of the line between where the syringe is connected and the patient should be known;
- The line with the IMP should be flushed initially with this volume to make sure that the subsequent actual drug administration to the patient is within 60 min (+/- 15 minutes), including a flush of the line volume at the end of the infusion as explained below.
- To ensure that all of the intended dose is administered, with no residual drug volume remaining in the syringe or infusion line upon the completion of administration, a flush of the infusion line at the end of the infusion is required. This flush should be performed at the same rate as the InO infusion to prevent bolusing of the drug product. Sites should follow their normal standard of care process to perform this flush. An example administration and flush process is provided in Appendix 6.
- We suggest to use a 2-way adapter with one syringe containing the IMP and the other containing saline for flushing – to minimize 'bed-side' handling by nursing staff during administration.

Prior to InO treatment, patients should receive pretreatment medications supplied by the site to reduce the incidence and severity of possible infusion syndrome characterized by fever and chills, and less commonly hypotension, as defined in Section 4.0, Tables 9 and 10. Premedication before InO may also include antiemetics. Pretreatment with methylprednisolone (or other corticosteroid) is recommended, especially in the first cycle. Also acetaminophen/paracetamol and diphenhydramine (or other antihistamine) approximately 0.5 to 2 hours before each InO administration may be used, although they are usually not needed when methylprednisolone is administered.

In cases of infusion reactions, the infusion should be discontinued and appropriate medical treatment, as needed, should be instituted (eg, glucocorticoids, epinephrine, bronchodilators, or oxygen). Depending on the severity of the infusion reaction and interventions required, the investigator could consider restarting the infusion at a reduced rate.

6.1.6 Medication Errors

Medication errors may result, in this study, from the administration or consumption of the wrong drug, by the wrong patient, at the wrong time, or at the wrong dosage strength. Such medication errors occurring to a study participant are to be captured on the Medication page of the CRFs and on the SAE form when appropriate. In the event of medication dosing error, the sponsor should be notified immediately.

Medication errors are reportable irrespective of the presence of an associated AE/SAE, including:

- Medication errors involving patient exposure to the investigational product.
- Potential medication errors or uses outside of what is foreseen in the protocol that do or do not involve the participating subject.

Medication errors that involve the participating subject will be captured on the Medication CRF. In case the error is accompanied by an AE, as determined by the Investigator, the associated adverse event(s) is captured on the adverse event (AE) CRF page (see Section 12.3 for further details).

6.1.7 Drug Storage and Accountability

InO should be stored refrigerated 2-8° Celsius and protected from light as detailed in the IP manual. In brief, InO must be protected from light during preparation and administration of the infusion using an ultraviolet protective covering.

Each investigative site must maintain adequate records documenting the inventory and disposition of all InO received, used and unused during the cycle of this clinical trial. The InO supplied for this trial is for investigational use only and to be used only within the context of treating patients formally enrolled onto this clinical trial by authorized personnel experienced in handling cytotoxic therapies. The Investigator, or an approved representative (e.g. pharmacist), will ensure that all study drug is stored in a secured area, under recommended storage conditions and in accordance with applicable regulatory requirements.

To ensure adequate records, all study drugs must be accounted for on the drug accountability inventory forms. Used or partially used vials can be destroyed at the site and accurate records must be kept and made available to trial personnel on request and during monitoring visits. Unless otherwise authorized by trial personnel, at the end of the clinical study all InO supplies unallocated or unused by the patients must be destroyed according to the centers own practice, and accountability logs completed accordingly.

IMP traceability must be employed at sites starting at the point of dispensing to the patient.

## 6.2 Methotrexate (MTX)

Please refer to the Summary of Product Characteristics for full details.

### 6.2.1 Source and Pharmacology

Methotrexate is a folate analogue that inhibits the enzyme dihydrofolate reductase, halting DNA, RNA, and protein synthesis. Initial IV half-life is about 1.2 hours, with a second phase of 10.4 hours. About 50% is bound to protein. Transport into the cell is carrier-mediated. Once in the cell, MTX (Glu)n are formed, the number of which are related to the cytocidal effect. Once MTX (Glu)n are formed, they do not pass back out of the cell unless converted back to MTX. The elimination of MTX from the CSF after an intrathecal dose is characterized by a biphasic curve with half-lives of 4.5 and 14 hours. After intrathecal administration of 12 mg/m², the lumbar concentration of MTX is ~100 times higher than in plasma. (Ventricular concentration is ~ 10% of lumbar concentration).

### 6.2.2 Toxicity

***Table 17. Toxicities of intrathecal methotrexate***

| | **Common**<br>Happens to 21-100 children out of every 100 | **Occasional**<br>Happens to 5-20 children out of every 100 | **Rare**<br>Happens to <5 children out of every 100 |
|---|---|---|---|
| **Immediate:**<br>Within 1-2 days of receiving drug | Nausea, headache | Arachnoditis: (headache, fever, vomiting,meningismus, nuchal rigidity, and pleocytosis) | Anaphylaxis, vomiting, seizures, confusion, back pain, rash, bleeding into subarachnoid or subdural space (risk > with platelet counts < 20,000), |
| **Prompt:**<br>Within 2-3 weeks, prior to the next cycle | | | Myelosuppression, ataxia, somnolence, cranial nerve palsy, subacute myelopathy (paraparesis/paraplegia), speech disorders, pain in the legs, bladder dysfunction |
| **Delayed:**<br>Any time later during therapy, excluding the above conditions | | Learning disability (L), Cognitive disturbance | Leukoencephalopathy (L) |
| **Late:**<br>Any time after completion of treatment | | | Progressive CNS Deterioration* |
| **Unknown Frequency and Timing:** Fetal and teratogenic toxicities | | | |

* *May be enhanced by HDMTX and/or cranial irradiation.*
*(L) Toxicity may also occur later.*

### 6.2.3 Formulation and Stability

Intact vials may be stored at room temperature (22°-25°C) and are stable for at least 2 years or until date of expiration. IT MTX: Available in various dosages in preservative-free liquid, or as a lyophilized powder. Reconstitute the powder with buffered saline solution. The methotrexate solutions may be further diluted with buffered saline or the patient's own CSF to an appropriate volume for administration. After mixing it should be used within 24 hours, since MTX contains no antibacterial preservative.

### 6.2.4 Guidelines for Administration

See Treatment section of the protocol in Section 4.

### 6.2.5 Supplier

All forms of methotrexate are commercially available and will be used from commercial stock. See package insert for further information.

### 6.2.6 Drug accountability

The lot number and expiration date should be documented at a minimum in the pharmacy dispensing system.

## 6.3 Cytarabine (Ara-C)

Please refer to the Summary of Product Characteristics for full details.

### 6.3.1 Source and Pharmacology

Cytarabine appears to act through the inhibition of DNA polymerase. A limited, but significant, incorporation of cytarabine into both DNA and RNA has also been reported. It exhibits cell phase specificity, primarily killing cells undergoing DNA synthesis (S-phase) and under certain conditions blocking the progression of cells from the G1 phase to the S-phase. Cytarabine is metabolized by deoxycytidine kinase and other nucleotide kinases to the nucleotide triphosphate (Ara-CTP), an effective inhibitor of DNA polymerase. Ara-CTP is inactivated by a pyrimidine nucleoside deaminase, which converts it to the nontoxic uracil derivative (Ara-U). It appears that the balance of kinase and deaminase levels may be an important factor in determining sensitivity or resistance of the cell to cytarabine. It has an initial distributive phase t½ of about 10 minutes, with a secondary elimination phase t½ of about 1 to 3 hours. Peak levels after intramuscular or subcutaneous administration of cytarabine occur about 20 to 60 minutes after injection and are lower than IV administration. Intrathecally administered doses are metabolized and eliminated more slowly with a t½ of about 2 hours.

### 6.3.2 Toxicity / Adverse Events

***Table 18. Toxicities of intrathecal cytarabine***

| | **Common**<br>Happens to 21-100 children out of every 100 | **Occasional**<br>Happens to 5-20 children out of every 100 | **Rare**<br>Happens to < 5 children out of every 100 |
|---|---|---|---|
| **Immediate:**<br>Within 1-2 days of receiving drug | Nausea, vomiting, fever,<br>headache | Arachnoiditis | Rash, somnolence, meningismus, convulsions, paresis |
| **Prompt:**<br>Within 2-3 weeks, prior to the next cycle | | | Myelosuppression, ataxia |
| **Delayed:**<br>Any time later during therapy, excluding the above condition | | | Necrotizing leukoencephalopathy, paraplegia, blindness (in combination with XRT & systemic therapy) |

### 6.3.3 Formulation and stability

Cytarabine for Injection is available in vials with different content containing a sterile powder for reconstitution. For intrathecal administration it is essential that a preservative free solution is used.
Stability: Intrathecal cytarabine is stable for 24 hours at 25ºC, but contains no preservative and should be administered as soon as possible after preparation.

### 6.3.4 Guidelines for Administration

Intrathecal: For intrathecal administration, dilute with 5-10 mL (or volume per institutional practice) preservative free 0.9% sodium chloride injection

### 6.3.5 Supplier

Cytarabine is commercially available and will be used from commercial stock. See package insert and prescriber information for further information.

### 6.3.6 Drug accountability

The lot number and expiration date should be documented at a minimum in the pharmacy dispensing system.

## 6.4 Prednisolone for IT use

Please refer to the Summary of Product Characteristics for full details

### 6.4.1 Source and Pharmacology

Prednisolone is a synthetic compound closely related to cortisol and the active metabolite of prednisone. The elimination of prednisolone from the CNS is prolonged. Note that a formulation should be used that is registered for intrathecal use, which should not contain potentially neurotoxic excipients. An example is Di-Adreson F aquosum.

### 6.4.2 Toxicity / Adverse Events

Toxicity related to IT steroids are mainly related to the association with cytarabine and methotrexate for triple intrathecal therapy. Most authors do agree that the inclusion of IT corticosteroid has a beneficial effect on the profile of adverse effects, given that it decreases the risk of chemical arachnoiditis. An increased risk of infection may potentially occur with IT steroids.

### 6.4.3 Formulation and Stability

For intrathecal administration, use prednisolone for injection **WITHOUT** preservative. Do not reconstitute vial with bacteriostatic water for injection.

### 6.4.4 Supplier

Prednisolone is commercially available and will be used from commercial stock. See package insert for further information.

### 6.4.5 Drug accountability

The lot number and expiration date should be documented at a minimum in the pharmacy dispensing system.

## 6.5 Hydrocortisone for IT use

Please refer to the Summary of Product Characteristics for full details

### 6.5.1 Source and Pharmacology

Hydrocortisone is a glucocorticoid. Glucocorticoids are adrenocortical steroids, both naturally-occurring and synthetic. Hydrocortisone is believed to be the principal corticosteroid secreted by the adrenal cortex. Naturally-occurring glucocorticosteroids (hydrocortisone and cortisone), which also have salt-retaining properties, are used as replacement therapy in adrenocortical deficiency states. They are also used for their potent anti-inflammatory effects in disorders of many organ systems.

### 6.5.2 Toxicity / Adverse Events

Toxicity related to intrathecal steroids are mainly related to the association with cytarabine and methotrexate for triple intrathecal therapy. Most authors do agree that the inclusion of IT corticosteroid has a beneficial effect on the profile of adverse effects, given that it decreases the risk of chemical arachnoiditis. An increased risk of infection may rappresent an additional toxicity with intrathecal steroids.

### 6.5.3 Formulation and Stability

For intrathecal administration, use hydrocortisone sodium succinate (Solu-Cortef) for injection WITHOUT preservative. Do not reconstitute vial with bacteriostatic water for injection.

### 6.5.4 Supplier

Hydrocortisone is commercially available and will be used from commercial stock. See package insert for further information.

#### 6.5.5 Drug accountability

The lot number and expiration date should be documented at a minimum in the pharmacy dispensing system.

### 6.6 Dexamethasone for systemic administration

Please refer to the Summary of Product Characteristics for full details.

#### 6.6.1 Source and Pharmacology

Dexamethasone possesses the effects of other glucocorticoids and is among the most active members of its class. Glucocorticoids are adrenocortical steroids, both naturally occurring and synthetic, which are readily absorbed from the gastrointestinal tract. They cause profound and varied metabolic effects and in addition, they modify the body's immune responses to diverse stimuli. Naturally-occurring glucocorticoids (hydrocortisone and cortisone), which also have salt-retaining properties, are used primarily for their potent anti-inflammatory effects in disorders of many organ systems. Dexamethasone has predominant glucocorticoid activity with little propensity to promote renal retention of sodium and water. Glucocorticoids produce widespread and diverse physiologic effects on carbohydrate, protein, and lipid metabolism, electrolyte and water balance, functions of the cardiovascular system, kidney, skeletal muscle, and the nervous systems. Glucocorticoids reduce the concentration of thymus-dependent lymphocytes (T-lymphocytes), monocytes, and eosinophils. Glucocorticoids selectively bind to the cortisol receptors on human lymphoid cells which are found in larger numbers on leukemic lymphoblasts. They also decrease binding of immunoglobulin to cell surface receptors and inhibit the synthesis and/or release of interleukins, thereby decreasing T lymphocyte blastogenesis and reducing expansion of the primary immune response. The specific cellular mechanisms that act to halt DNA synthesis are thought to be related to inhibition of glucose transport or phosphorylation, retardation of mitosis, and inhibition of protein synthesis. The biological half-life of dexamethasone in plasma is about 190 minutes. Binding of dexamethasone to plasma proteins is less than for most other corticosteroids and is estimated to be about 77%. Up to 65% of a dose is excreted in the urine in 24 hours, the rate of excretion being increased following concomitant administration of phenytoin.

#### 6.6.2 Toxicity / Adverse Events

***Table 19*. *Toxicities for systemically administered dexamethasone***

| | |
|---|---|
| Infections and infestations | Increased susceptibility to, or exacerbation of, (latent) infections with masking of clinical symptoms, opportunistic infections |
| Blood and lymphatic system disorders | Leukocytosis, lymphopenia, eosinopenia, polycythemia, abnormal coagulation |
| Endocrine disorders | Suppression of the hypothalamic-pituitary-adrenal axis and induction of Cushing's syndrome (typical symptoms: full-moon face, plethora, truncal obesity), secondary adrenal and pituitary insufficiency (especially in stress such as trauma or surgery), growth suppression in infancy, childhood and adolescence, menstrual irregularity and amenorrhoea, hirsutism |
| Metabolism and nutrition disorders | Weight gain, negative protein and calcium balance, increased appetite, sodium and water retention, potassium loss, hypokalemic alkalosis, manifestation of latent diabetes mellitus, impaired carbohydrate tolerance with increased dose requirements of antidiabetic therapy, hypercholesterolemia, hypertriglyceridaemia |
| Psychiatric disorders | Psychological dependence, depression, insomnia, aggravated schizophrenia, mental illness, from euphoria to manifest psychosis |
| Nervous system disorders | Increased intracranial pressure with papilloedema in children (pseudotumor cerebri) usually following discontinuation of treatment |
| Eye disorders | Elevated intraocular pressure, glaucoma, papilloedema, cataract, |
| Vascular disorders | Hypertension, vasculitis, increased risk of thrombosis/thromboembolism (increase in coagulability of blood may lead to thromboembolic complications) |
| Gastrointestinal disorders | Dyspepsia, abdominal distension, gastric ulcers with perforation and bleeding, acute pancreatitis, ulcerative esophagitis, oesophageal candidiasis, flatulence, nausea, vomiting, elevation in serum liver enzyme levels (usually reversible upon discontinuation). |

| | |
|---|---|
| Skin and subcutaneous tissue disorders | Hypertrichosis, skin atrophy, telangiectasia, striae, erythema, steroid acne, petechiae, ecchymosis, |
| Musculoskeletal and connective tissue disorders | Premature epiphyseal closure, osteoporosis, fractures of the spine and long bones, aseptic necrosis of the femoral and the humeral bones, proximal myopathy, muscle weakness, loss of muscle mass |
| General disorders and administration site conditions | Reduced response to vaccination and skin tests.<br>Malaise, steroid withdrawal syndrome: a too rapid reduction in corticosteroid dose after prolonged treatment can lead to acute adrenal insufficiency, hypotension, and death. A withdrawal syndrome may present with fever, myalgia, arthralgia, rhinitis, conjunctivitis, painful itchy skin nodules and weight loss. |

6.6.3 Formulation and Stability

Oral formulation: Tablets, store below 25°C protected from light. IV formulation: From a microbiological point of view, the product should be used immediately after opening. If not used immediately, in-use storage times and conditions prior to use are the responsibility of the user and would normally not be longer than 24 h at 2 to 8°C, unless dilution has taken place in controlled and validated aseptic conditions. Any unused portion of the product should be discarded immediately after use. Chemical and physical in-use stability of dilutions has been demonstrated for 24 h at 25°C. Dilutions should be used within 24 hours and discarded after use.

6.6.4 Supplier

Dexamethasone is commercially available and will be used from commercial stock. See package insert for further information.

6.6.5 Drug accountability

The lot number and expiration date should be documented at a minimum in the pharmacy dispensing system.

## 6.7 Vincristine

Please refer to the Summary of Product Characteristics for full details.

6.7.1 Source and Pharmacology

Pharmacotherapeutic group: Antineoplastic agent - vinca alkaloid
Vinca alkaloids are classical “spindle poisons”, that bind to the microtubular protein tubulin and block cells during metaphase by both preventing polymerisation of tubulin and subsequent formation of microtubules and by inducing depolymerisation of existing microtubules. Vincristine can also affect other cellular systems such as RNA and DNA synthesis, cyclic AMP, lipid biosynthesis and calmodulin-dependent Ca2+ transport ATPase. After the intravenous injection vincristine is rapidly cleared from the serum. Within 15-30 minutes more than 90% of the medicinal product is distributed from the serum to the tissues and other blood components. The distribution volume is 8.4 ± 3.2 l/kg during steadystate conditions. Twenty minutes after the intravenous administration, more than 50% of vincristine is bound to blood components, particularly to platelets, which contain high concentrations of tubulin. Analysis of plasma particulars shows that the plasma elimination of vincristine after a rapid intravenous administration can best be described as a triphasic model. The initial, mean and final half-lives are respectively 5 minutes, 2.3 hours and 85 hours (range 19-155 hours). Penetration into the cerebrospinal fluid after an intravenous bolus injection appears to be very low. Vincristine appears to be largely metabolised, probably in the liver by the microsomal enzyme system cytochrome P450, amongst which CYP3A.

6.7.2 Toxicity / Adverse Events

***Table 20. Toxicities of Vincristine***

| | **Common**<br>Happens to 21-100 children out of every 100 | **Occasional**<br>Happens to 5-20 children out of every 100 | **Rare**<br>Happens to < 5 children out of every 100 |
|---|---|---|---|
| **Immediate:**<br>Within 1-2 days of receiving drug | Nausea, vomiting, abdominal pain. Constipation | Convulsions, frequently associated with hypertension. Paralytic ileus. Injection site irritation. | Syndrome of inappropriate antidiuretic hormone |

| | | | |
|---|---|---|---|
| **Prompt:**<br>Within 2-3 weeks, prior to the next cycle | peripheral neuropathy (mixed sensorimotor)<br><br>Alopecia | Severe bone marrow depression, anaemia, leukopenia and thrombocytopenia | |
| **Delayed:**<br>Any time later during therapy, excluding the above condition | | Azoospermia | |

6.7.3 Formulation and stability

After dilution chemical and physical in-use stability of the solution prepared for injection or infusion has been demonstrated for 24 hours at 2-8 °C and also at 15 to 25 °C when diluted to a concentration range of 0.01 mg/ml to 0.1 mg/ml in 9 mg/ml (0.9%) sodium chloride solution for infusion or in 50 mg/ml (5%) glucose solution for infusion. From a microbiological point of view, the diluted solution should be used immediately. If not used immediately, in-use storage times and conditions prior to use are the responsibility of the user and would normally not be longer than 24 hours at 2 to 8 °C, unless dilution has taken place in controlled and validated aseptic conditions. Store and transport refrigerated (2-8°C). Keep vial in the outer carton in order to protect from light.

6.7.4 Guidelines for Administration

It is recommended to infuse vincristine sulphate as bolus injection diluted to a volume of 15 ml with Sodium Chloride 9 mg/ml (0.9%) Solution for Injection. After administration the vein must be flushed through thoroughly. Care should be taken to avoid extravasation as this may cause local ulceration. Vincristine is for intravenous use only, fatal if given by any other route (e.g. IT administration).

6.7.5 Supplier

Vincristine is commercially available and will be used from commercial stock. See package insert and prescriber information for further information.

6.7.6 Drug accountability

The lot number and expiration date should be documented at a minimum in the pharmacy dispensing system.

## 6.8 Pegylated (PEG-) asparaginase

Please refer to the Summary of Product Characteristics for full details.

6.8.1 Source and Pharmacology

The mechanism of action of L-asparaginase is the enzymatic cleavage of the amino acid L-asparagine into aspartic acid and ammonia. Depletion of L-asparagine in blood serum results in inhibition of protein-synthesis, DNA-synthesis and RNA-synthesis, especially in leukaemic blasts which are not able to synthetise L-asparagine, thus undergoing apoptosis. Normal cells, in contrast, are capable of synthesising L-asparagine and are less affected by its rapid withdrawal during treatment with the enzyme L-asparaginase. The PEGylation does not change the enzymatic properties of L-asparaginase, but it influences the pharmacokinetics and immunogenicity of the enzyme. In adults with leukaemia, the initial enzymatic activity after intravenous adminstration was proportional to the dose. The elimination half-life from the plasma was between 1 and 6 days and appeared to be unaffected by the dose. It was also independent of age, sex, body surface area, renal and hepatic function, diagnosis and severity of the illness. However, terminal half-life was shorter in hypersensitive patients than in non-hypersensitive patients, and may be decreased due to the formation of high levels of anti-drug antibodies.
The distribution volume was in the range of the estimated plasma volume. After a one-hour intravenous infusion, asparaginase activity was detected for at least 15 days after the first treatment.

### 6.8.2 Toxicity / Adverse Events

***Table 21. Toxicities of PEG-asparaginase***

| | **Common**<br>Happens to 21-100 children out of every 100 | **Occasional**<br>Happens to 5-20 children out of every 100 | **Rare**<br>Happens to < 5 children out of every 100 |
|---|---|---|---|
| **Immediate:**<br>Within 1-2 days of receiving drug | Hypersensitivity, Urticaria, Rash, Anaphylactic reactions | Hypertriglyceridaemia, Hyperlipidaemia, Amylase increased, Alanine aminotransferase increase, bilirubin increase, vomiting, stomatitis | |
| **Prompt:**<br>Within 2-3 weeks, prior to the next cycle | Hyperglycaemia Pancreatitis, Diarrhoea, Abdominal pain | Febrile neutropenia, Infections, Anaemia, Neutrophil count decreased, Platelet count decreased, Thrombosis Convulsion, Peripheral Motor Neuropathy, Syncope | Hyperammonaemia, hepatic failure with potentially fatal outcome, hepatic necrosis, cholestasis, jaundice |
| **Delayed:**<br>Any time later during therapy, excluding the above condition | | | Late effects of pancreatitis, neurological damage due to thrombosis and infarction or bleeding |

### 6.8.3 Formulation and stability

Chemical and physical in-use stability has been demonstrated for 2 days at 2°C – 8°C. From a microbiological point of view, the product should be used immediately. If not used immediately, in-use storage times and conditions prior to use are the responsibility of the user and would normally not be longer than 24 hours at 2°C – 8°C unless reconstitution/dilution has taken place in controlled and validated aseptic conditions.

### 6.8.4 Guidelines for Administration

The daily amount of PEG-asparaginase needed per patient can be diluted in a final volume of 50 - 250 ml sodium chloride 9 mg/ml (0.9 %) solution for injection. The diluted solution of asparaginase may be infused over 0.5 to 2 hours. Asparaginase must not be administered as a bolus dose.

### 6.8.5 Supplier

PEG-asparaginase is commercially available and will be used from commercial stock. See package insert and prescriber information for further information.

### 6.8.6 Drug accountability

The lot number and expiration date should be documented at a minimum in the pharmacy dispensing system.

## 7.0 REQUIRED OBSERVATIONS/MATERIAL AND DATA TO BE ACCESSIONED

All protocol-specified clinical hematology, blood chemistries, and bone marrow aspirations and/or biopsies are to be performed under the auspices of the local laboratory at each investigational site, unless the material needs to be sent to a central laboratory as detailed below. Additional assessments may be obtained as needed for good patient care. Response evaluation by radiology will be assessed locally and not centrally reviewed. All screening tests must be done within 10 days of enrollment.

### 7.1 Schedule of events for BCP-ALL patients in Stratum 1A and Phase 2 cohort

**Table 22** ***Schedule of events for BCP-ALL patients in Stratum 1A and Phase 2 cohort.*** *Further follow-up is described in* ***Table 33.***

| Parameter | Screening | Course 1 | | | | Course 2 | | | | Course 3-6 | | | | End of Therapy | Safety follow-up |
|---|---|---|---|---|---|---|---|---|---|---|---|---|---|---|---|
| **Day** | **-10 to 0** | **1** | **8** | **15** | **22 (±2 day) –day 42** | **1 (±1 day)** | **8 (±1 day)** | **15 (±1 day)** | **28 (±2 day) – day 42** | **1 (±1 day)** | **8 (±1 day)** | **15 (±1 day)** | **28 (±2 day) – day 42** | **4 w. post last InO dose (±1 week)[20]** | **10 w. post last-InO dose (±1 week)[21]** |
| **Inform. consent /assent [1]** | X | | | | | | | | | | | | | | |
| **Med. History[2]** | X | | | | | | | | | | | | | | |
| **Full Clinical Examination** | X | X | X | X | X | X | | | | X | | | | X | X |
| **Height, weight, BSA[3]** | X | X | | | | X | | | | X | | | | | |
| **Vital signs[4]** | X | X | X | X | X | X | X | X | | X | X | X | | X | |
| **Performance Status[5]** | X | X | | | | X | | | | X | | | | X | X |
| **Menarchal status** | X | X | | | | X | | | | X | | | | X | X |
| **CBC and differential** | X | X (and day 4) | X | X | X | X | X | X | X | X | X | X | X | X | X |
| **Chemistry Panel[6]** | X | X | X[#] | X | X | X | X | X | X | X | X[#] | X | X | X | X |
| **Amylase, Lipase** | X | X | | | | X | | | | X | | | | X | X |
| **Urine or Serum Pregnancy and contraception check[7]** | X | X | | | | X | | | | X | | | | X | X |
| **Echocardiogram or MUGA** | X | | | | | | | | | | | | | X | |
| **ECG[8]** | X | X (Pre and Post InO) | | | | X (Pre and Post InO) | | | | X (Pre and Post InO) | | | | X | |

| Parameter | Screening | Course 1 | | | | Course 2 | | | | Course 3-6 | | | | End of Therapy | Safety follow-up |
|---|---|---|---|---|---|---|---|---|---|---|---|---|---|---|---|
| Day | -10 to 0 | 1 | 8 | 15 | 22 (±2 day) –day 42 | 1 (±1 day) | 8 (±1 day) | 15 (±1 day) | 28 (±2 day) – day 42 | 1 (±1 day) | 8 (±1 day) | 15 (±1 day) | 28 (±2 day) – day 42 | 4 w. post last InO dose (±1 week)[20] | 10 w. post last-InO dose (±1 week)[21] |
| Chest X-ray[9] | X | | | | | | | | | | | | | | |
| Eligibility and registration[10] | X | | | | | | | | | | | | | | |
| LP with cell count + diff & it therapy[11] | X | | | | X | | | | X | | | | X | X | |
| BM for flow cytometry & morph (local) | X | | | | X | | | | X | | | | X | X | |
| BM cytogen.[12] | X | | | | | | | | | | | | | | |
| PB and BM for MRD (sect. 7.6)[13] | X | | | | X | | | | X | | | | X | X | |
| CD22 expression levels[13, 14] | X | | | | X[14] | | | | X[14] | | | | X[14] | X[14] | |
| PB CD22 Saturation Kinetics[13, 15] | | X (prior to and at the end of the infusion[15]) | X (prior to and end of the infusion[15]) | | | | | | | | | | | | |
| PK[13, 16] | | X | X | X | X | X | X | X | X | X[16] (course 3 only) | X[16] (course 3 only) | X[16] (course 3 only) | X[16] (course 3 only) | | |
| Immunogenicity samples[13,17] | X | | | | | X | | | | X | | | | X | |
| PB and BM Calicheamicin Sens.[13,18] | X | | | | | | | | | | | | | | |
| Number of CD19+ B-Cells and serum Ig levels[19] | X | | | | X | | | | | X (course 3 & 6 only) | | | | X | |
| Concomitant medication | X | X | X | X | X | X | X | X | X | X | X | X | X | X | X |
| Safety evaluation adverse events | X | X | X | X | X | X | X | X | X | X | X | X | X | X | X |
| InO infusion (see tables 9, 10 & 11) | | X | X | X | | X | X | X | | X | X | X | | | |

| Parameter | Screening | Course 1 | | | | Course 2 | | | | Course 3-6 | | | | End of Therapy | Safety follow-up |
|---|---|---|---|---|---|---|---|---|---|---|---|---|---|---|---|
| Day | -10 to 0 | 1 | 8 | 15 | 22 (±2 day) -42 | 1 (±1 day) | 8 (±1 day) | 15 (±1 day) | 28 (±2 day) – day 42 | 1 (±1 day) | 8 (±1 day) | 15 (±1 day) | 28 (±2 day) – day 42 | 4 w. post last InO dose (± 1 week)[20] | 10 w. post last-InO dose (± 1 week)[21] |
| Follow-up for Relapse, events, survival & start of new therapy | | | | | | | | | | | | | | X | X |

BM=bone marrow, BSA=body surface area, CBC=complete blood count, ECG=electrocardiography LP=lumbar puncture, MRD=minimal residual disease, PB=peripheral blood, PK=pharmacokinetics, it=intrathecal

[1] Informed consent/assent must be obtained before any study specific investigations are performed
[2] Medical history including review of cancer diagnosis and previous cancer treatment (screening/pre-study visit only), current medications and any current medical conditions or abnormalities.
[3] Calculate BSA according to the Mosteller formula as given in Appendix 4
[4] Vital signs include pulse, respirations, blood pressure and temperature
[5] Performance status, see Appendix 1
[6] Chemistry Panel (including sodium, potassium, calcium, phosphate, uric acid, creatinine, urea, total protein, albumin, Total and direct bilirubin, AST, ALT, alkaline phosphatase and gamma-glutamyltranferase (GGT).
[7] Serum/Urine Pregnancy Test and Contraception Check: for women of child bearing potential, a serum or urine pregnancy test, with sensitivity of at least 25 mIU/mL, will be performed as indicated and from then on monthly until 8 months after the last InO dose, following the CTFG recommendations. A pregnancy test should also be done whenever 1 menstrual cycle is missed during treatment (or potential pregnancy is suspected). Additionally, for male and female patients who are of childbearing potential, investigators must also document method of contraception For male patients, the method of contraception should be documented monthly until 5 months after the last dose of InO, following the CTFG recommendations. See also section 7.12
[8] Pre-dose ECG should be done after pre-medications are given but before InO infusion begins. Post dose ECG should be done within 15 minutes after the end of the InO infusion. The QT interval for each exam will be recorded on the CRF.
[9] In case of suspected subclinical fungal infections perform CT-scan
[10] Registration: after confirmation of eligibility, patient number and dose level allocation will be assigned by the Sponsor.
[11] Intrathecal therapy will be given as described in Section 4.3.3.
[12] Cytogenetic analysis will be performed locally according to local standard of care guidelines, but results will be centrally reviewed by an expert cytogeneticist.
[13] Send to central laboratory (see Section 7 and procedure manual). Please note: for patients <18 Kg collect reduced blood volumes for MRD analysis.
[14] CD22-expression should be repeated in case of loss of response/progression to study clonal evolution and should be sent to the central laboratory for analysis in such cases. See Section 7.7
[15] CD22 saturation is to be determined in peripheral blood at day 1 and 8 prior to and at the end of the infusion
Please note: for patients of 12-18 Kg at day 1 CD22 saturation assessment will be performed with a reduced amount of blood, and at day 8 it will be skipped. For subjects <12 kg the CD22 saturation assessment will be excluded. See Section 7.7 for details.
[16] PK samples should be collected in course 1, 2 and 3. Please note: for patients of 12-18 Kg and <12 kg specific samples will be skipped as detailed in Section 7.8
[17] Immunogenicity samples should be taken as described in Section 7.9.
[18] In-vitro calicheamicin sensitivity samples will be taken at screening as described in section 7.10, and require both PB and BM, and can be sent together with the MRD samples. Please note: for subjects < 12 kg calicheamicin sensitivity will not be performed (see Section 7.10 In-vitro Calicheamicin Resistance).
[19] The absolute number of peripheral blood $CD19^+$ B-Cells and serum immunoglobulin levels (IgM and IgG) should be measured every 3 months after the end of therapy until disease progression or HSCT/CAR-T cell therapy or maximum for one year whichever occurs first. For patients <12Kg the immune-activity analysis (CD19+, IgG and IgM) will be excluded.
[20.] The end-of-treatment (EOT) visit should occur approximately 4 weeks after the last dose of InO. However, in the event that a patient requires initiation of a new anti-cancer therapy, the EOT visit should be performed before the initiation of the new anti-cancer therapy as close as possible to 4 weeks after the last dose of study drug.
[21] Note that adverse events, safety laboratory tests and associated concomitant medications must continue to be collected in the CRF through at least 10 weeks after the last dose of study therapy unless a new anti-cancer therapy is given.
*# Liver tests (bilirubin and ALT/AST levels) need to be determined prior to each day 8 infusion of InO. In case of a >2.5 ULN elevation in ALT/AST, or >1.5 xULN for total bilirubin,*

*delay the InO dose according to Section 4.4.1 (excluding cases where these aberrations are due to hemolysis or Gilbert's syndrome).*

## 7.2 Schedule of events for BCP-ALL in Stratum 1B/1B-ASP patients – combination therapy cycle 1 and 2

***Table 23. Schedule of events for BCP-ALL in Stratum 1B/1B-ASP patients – combination therapy cycle 1 and 2.*** *Further follow-up is described in* ***Table 33****. For those patients proceeding with InO single agent refer to* ***Table 24****.*

| Parameter | Screening | Cycle 1 (and potentially Cycle 2) | | | | | | | | End of Therapy | Safety follow-up |
|---|---|---|---|---|---|---|---|---|---|---|---|
| **Day** | **-10 to 0** | **1(±1 day)** | **3** | **8(±1 day)** | **10** | **15** | **17** | **24 (±1 day)** | **28[19] (±2 day) – day 42** | **4 w. post last InO dose (±1 week)[20]** | **10 w. post last-InO dose (±1 week)[21]** |
| **Inform. consent /assent [1]** | X | | | | | | | | | | |
| **Med. History[2]** | X | | | | | | | | | | |
| **Full Clinical Examination** | X | X | X | X | X | X | X | X | X | X | X |
| **Height, weight, BSA[3]** | X | X | | | | | | | X | | |
| **Vital signs[4]** | X | X | | X | | X | | X | X | X | |
| **Performance Status[5]** | X | X | | | | | | | X | X | X |
| **Menarchal status** | X | X | | | | | | | | X | X |
| **CBC and differential** | X | X | X | X | X | X | X | X | X | X | X |
| **Chemistry Panel[6]** | X | X | | X[#] | | X[#] | | X | X | X | X |
| **Coagulation [7]** | X | X | | | | | | | x | | |
| **Amylase, Lipase** | X | X | | X | | X | | X | X | X | X |
| **Urine or Serum Pregnancy and contraception check[8]** | X | X | | | | | | | | X | X |
| **Echocardiogram or MUGA** | X | | | | | | | | | X | |
| **ECG[9]** | X | X (Pre and Post InO) | | | | | | | | X | |
| **Chest X-ray[10]** | X | | | | | | | | | | |
| **Eligibility and registration[11]** | X | | | | | | | | | | |

| Parameter | Screening | Cycle 1 (and potentially Cycle 2) | | | | | | | | End of Therapy | Safety follow-up |
|---|---|---|---|---|---|---|---|---|---|---|---|
| **Parameter** | **Screening** | | | | | | | | | **End of Therapy** | **Safety follow-up** |
| **Day** | **-10 to 0** | **1** | **3** | **8** | **10** | **15** | **17** | **24 (±1 day)** | **28[19] (±2 day)-day 42** | **4 w. post last InO dose (±1 week)[20]** | **10 w. post last-InO dose (±1 week)[21]** |
| **LP with cell count + diff & it therapy[12]** | X | | | X | | | | | X | X | |
| **BM for flow cytometry & morph (local)** | X | | | | | | | | X | X | |
| **BM cytogenetics[13]** | X | | | | | | | | | | |
| **PB and BM for MRD (sect. 7.6)[14]** | X | | | | | | | | X | X | |
| **CD22 expression levels[14, 15]** | X | | | | | | | | X[15] | X[15] | |
| **PK[14, 16]** | | X | | X | | X | | X | | | |
| **Concomitant medication** | X | X | | X | | X | | X | X | X | X |
| **Adverse events** | X | X | | X | | X | | X | X | X | X |
| **InO infusion (see Table 10)** | | X | | X | | X | | | | | |
| **Steroid premedication[17]** | | Dex IV | | Methylpred. | | Dex IV | | | | | |
| **Other chemotherapy[17]** | | Start 5-day pulse Dex | VCR | | VCR | Start 5-day pulse Dex | VCR | VCR | | | |
| **Stratum 1B-ASP[18]** | | | PEG-ASP | | | | PEG-ASP | | | | |
| **Follow-up for Relapse, AEs, survival & start of new ALL therapy** | | | | | | | | | | X | X |

BM=bone marrow, BSA=body surface area, CBC=complete blood count, ECG=electrocardiography LP=lumbar puncture, MRD=minimal residual disease, MUGA=multigated acquisition scan, PB=peripheral blood, PEG-ASP=pegylated asparaginase, PK=pharmacokinetics, it=intrathecal; Methylpred.=methylprednisolone; Dex=Dexamethasone; VCR=Vincristine.

[1] Informed consent/assent must be obtained before any study specific investigations are performed
[2] Medical History including review of cancer diagnosis and previous cancer treatment (screening/pre-study visit only), current medications and any current medical conditions or

abnormalities.
[3] Calculate BSA according to the Mosteller formula, see Appendix 4
[4] Vital signs include pulse, respirations, blood pressure and temperature
[5] Performance status, see Appendix 1.
[6] Chemistry Panel (including sodium, potassium, calcium, phosphate, uric acid, creatinine, urea, total protein, albumin, Total and direct bilirubin, AST, ALT, glucose, alkaline phosphatase and gamma-glutamyltranferase (GGT)
[7] Screening for clotting parameters (PT, aPTT, fibrinogen)
[8] Serum/Urine Pregnancy Test and Contraception Check: for women of child bearing potential, a serum or urine pregnancy test, with sensitivity of at least 25 mIU/mL, will be performed as indicated and from then on monthly until 8 months after the last InO dose, following the CTFG recommendations. A pregnancy test should also be done whenever 1 menstrual cycle is missed during treatment (or potential pregnancy is suspected). Additionally, for male and female patients who are of childbearing potential, investigators must also document method of contraception. For male patients, the method of contraception should be documented monthly until 5 months after the last dose of InO, following the CTFG recommendations. See also section 7.12
[9] Pre-dose ECG should be done after pre-medications are given but before InO infusion begins. Post dose ECG should be done within 15 minutes after the end of the InO infusion. The QT interval for each exam will be recorded on the CRF.
[10] In case of suspected subclinical fungal infections perform CT-scan.
[11] Registration: after confirmation of eligibility, patient number and dose level allocation will be assigned by the Sponsor, as well as wheather the patient is enrolled during the phase of treatment with or without PEG-asparaginase.
[12] Intrathecal therapy will be given as described in Section 4.3.3. The it injection may be combined with the diagnostic LP, in which case the LP on day 1 can be omitted.
[13] Cytogenetic analysis will be performed locally according to local standard of care guidelines, but results will be centrally reviewed by an expert cytogeneticist.
[14] Send to central laboratory (see section 7 and procedure manual). Please note: for patients <18 Kg reduced volumes are requested for MRD analysis and CD22 expression.
[15] CD22-expression should be repeated in case of loss of response/progression to study clonal evolution and should be sent to the central laboratory for analysis in such cases. See Section 7.7
[16] PK samples should be taken as detailed in Section 7.8. Please note: for patients of 12-18 Kg and <12 kg specific samples will be skipped as detailed in Section 7.8.
[17] Premedication therapy will be given as described in Section 4.3.2, ***Table 10***.
[18] Only patients enrolled in stratum 1B-ASP, as communicated at enrollment to sites, will receive PEG-ASP.
[19] Day 1 of subsequent cycles may be combined with day 28 of the previous cycle, if there are no contraindications to re-start therapy.
[20] The end-of-treatment (EOT) visit should occur approximately 4 weeks after the last dose of InO. However, in the event that a patient requires initiation of a new anti-cancer therapy, the EOT visit should be performed before the initiation of the new anti-cancer therapy as close as possible to 4 weeks after the last dose of study drug.
[21] Note that adverse events, safety laboratory tests and associated concomitant medications must continue to be collected in the CRF through at least 10 weeks after the last dose of study therapy unless a new anti-cancer therapy is given.
*# Liver tests (total bilirubin, AST and/or ALT) need to be determined prior to day 8 and 15 InO infusion. In case of a >5 ULN elevation in transaminases (> grade 2), or >1.5 xULN for total bilirubin, delay the dose of InO and IT therapy according to Section 4.4.1 (excluding cases where these aberrations are due to hemolysis or Gilbert's syndrome).*

## 7.3 Schedule of Events for Stratum 1B/1B-ASP, cycle 2 and/or beyond – for patients continuing therapy with single-agent InO.

***Table 24: Schedule of Events for Stratum 1B/1B-ASP, cycle 2 and/or beyond - for patients continuing therapy with single-agent InO.***

Further follow-up is described in ***Table 33***.

| **Parameter** | **Cycle 2** | | | | **Cycle 3-6[12]** | | | | **End of Therapy** | **Safety follow-up** |
|---|---|---|---|---|---|---|---|---|---|---|
| **Day** | **1 (±1 day)** | **8 (±1 day)** | **15 (±1 day)** | **28[12] (±2 day) – day 42** | **1[12] (±1 day)** | **8 (±1 day)** | **15 (±1 day)** | **28 (±2 day) – day 42** | **4 w. post last InO dose (±1 week)[13]** | **10 w. post last-InO dose (±1 week)[14]** |
| **Med. History[1]** | | | | | | | | | | |
| **Full Clinical Examination** | X | | | | X | | | | X | X |
| **Height, weight, BSA[2]** | X | | | | X | | | | | |
| **Vital signs[3]** | X | | | | X | | | | X | |
| **Performance Status[4]** | X | | | | X | | | | X | X |
| **Menarchal status** | X | | | | X | | | | X | X |
| **CBC and differential** | X | X | X | X | X | X | X | X | X | X |
| **Chemistry Panel[5]** | X | X | X | X | X | X | X | X | X | X |
| **Amylase, Lipase** | X | | | | X | | | | X | X |
| **Urine or Serum Pregnancy & contraception check[6]** | X | | | | X | | | | X | X |
| **Echocardio-gram or MUGA** | | | | | | | | | X | |
| **ECG[7]** | X (Pre and Post InO) | | | | X (Pre and Post InO) | | | | X | |

| Parameter | Cycle 2 | | | | Cycle [12] 3-6 | | | | End of Therapy | Safety follow-up |
|---|---|---|---|---|---|---|---|---|---|---|
| Day | 1 (±1 day) | 8 (±1 day) | 15 (±1 day) | 28 (±2 day) – day 42 | 1 (±1 day) | 8 (±1 day) | 15 (±1 day) | 28 (±2 day) – day 42 | 4 w. post last InO dose (±1 week)[13] | 10 w. post last-InO dose (±1 week)[14] |
| LP (cell count + diff); IT therapy[8] | X | | | X | | | | X | X | |
| BM for flow cytometry & morph (local) | | | | X | | | | X | X | |
| PB and BM for MRD (sect. 7.5)[9] | | | | X | | | | X | X | |
| CD22 expression levels[9, 10] | | | | X[10] | | | | X[10] | X[10] | |
| PK[9,11] | X | X | X | X | X (cycle 3 only) | X (cycle 3 only) | X (cycle 3 only) | X (cycle 3 only) | | |
| Concomitant medication | X | X | X | X | X | X | X | X | X | |
| Adverse events | X | X | X | X | X | X | X | X | X | X |
| InO infusion (see *Table 10*) | X | X | X | | X | X | X | | | |
| Follow-up (Relapse, AEs, survival & start of new ALL therapy) | | | | | | | | | X | X |

BM=bone marrow, BSA=body surface area, CBC=complete blood count, ECG=electrocardiography LP=lumbar puncture, MRD=minimal residual disease, PB=peripheral blood, PK=pharmacokinetics, it=intrathecal

[1] Medical History including current medications and any current medical conditions or abnormalities.
[2] Calculate BSA according to the Mosteller formula as given in Appendix 4
[3] Vital signs include pulse, respirations, blood pressure and temperature
[4] Performance status, see Appendix 1.
[5] Chemistry Panel (including sodium, potassium, calcium, phosphate, uric acid, creatinine, urea, total protein, albumin, Total and direct bilirubin, AST, ALT, glucose, alkaline phosphatase and gamma-glutamyltranferase (GGT).
[6] Serum/Urine Pregnancy Test and Contraception Check: for women of child bearing potential, a serum or urine pregnancy test, with sensitivity of at least 25 mIU/mL, will be performed as indicated and from then on monthly until 8 months after the last InO dose, following the CTFG recommendations. A pregnancy test should also be done whenever 1 menstrual cycle is missed during treatment (or potential pregnancy is suspected). Additionally, for male and female patients who are of childbearing potential, investigators must also document method of contraception For male patients, the method of contraception should be documented monthly until 5 months after the last dose of InO, following the CTFG recommendations. See also section 7.12
[7] Pre-dose ECG should be done after pre-medications are given but before InO infusion begins. Post dose ECG should be done within 15 minutes after the end of the InO infusion. The corrected QT interval (QTcF) for each exam will be recorded on the CRF.
[8] Intrathecal therapy will be given as described in Section 4.3.3. The IT injection may be combined with the diagnostic procedure, in which case the LP on day 1 can be omitted.
[9] Send to central laboratory (see section 7.8 and procedure manual). Please note: for patients <18 Kg reduced blood volumes are requested for MRD analysis and CD22 expression.
[10] CD22-expression should be repeated in case of loss of response/progression to study clonal evolution and should be sent to the central laboratory for analysis in such cases. See Section 7.7
[11] PK samples should be taken as detailed in Section 7.8. Please note: for patients of 12-18 Kg and <12 kg specific samples will be skipped as detailed in Section 7.8.
[12] Day 1 of subsequent cycles may be combined with day 28 of the previous cycle, if there are not contraindications to re-start therapy. Maximum 3 cycle are allowed before proceeding to HSCT.
[13] The end-of-treatment (EOT) visit should occur approximately 4 weeks after the last dose of InO. However, in the event that a patient requires initiation of a new anti-cancer therapy, the EOT visit should be performed before the initiation of the new anti-cancer therapy as close as possible to 4 weeks after the last dose of study drug.
[14] Note that adverse events, safety laboratory tests and associated concomitant medications must continue to be collected in the CRF through at least 10 weeks after the last dose of study therapy unless a new anti-cancer therapy is given.

### 7.4 Schedule of events for BCP-ALL patients in Stratum 3

**Table 25.** ***Schedule of events for BCP-ALL patients in Stratum 3 (VHR).*** *Further follow-up is described in* ***Table 34***.

| **Parameter** | **Screening** | **Cycle 1** | | | | **Cycle 2** | | | | **Cycle 3-6** | | | | **End of Therapy** | **Follow up after EOT until CAR-T/HSCT or disease progression** | **Safety follow-up** |
|---|---|---|---|---|---|---|---|---|---|---|---|---|---|---|---|---|
| **Day** | **-10 to 0** | **1** | **8** | **15** | **22 (±2 day) –day 42** | **1 (±1 day)** | **8 (±1 day)** | **15 (±1 day)** | **28 (±2 day) – day 42** | **1 (±1 day)** | **8 (±1 day)** | **15 (±1 day)** | **28 (±2 day) – day 42** | **4 w. post last InO dose (±1 week)[18]** | **Refer to Table 34 for the frequency of assess-ment** | **10 w. post last-InO dose (±1 week)[19]** |
| **Inform. consent /assent [1]** | X | | | | | | | | | | | | | | | |
| **Med. History[2]** | X | | | | | | | | | | | | | | | |
| **Full Clinical Examination** | X | X | X | X | X | X | | | | X | | | | X | | X |
| **Height, weight, BSA[3]** | X | X | | | | X | | | | X | | | | | | |
| **Vital signs[4]** | X | X | X | X | X | X | X | X | | X | X | X | | X | | |
| **Performance Status[5]** | X | X | | | | X | | | | X | | | | X | | X |
| **Menarchal status** | X | X | | | | X | | | | X | | | | X | | X |
| **CBC and differential** | X | X (and day 4) | X | X | X | X | X | X | X | X | X | X | X | X | | X |
| **Chemistry Panel[6]** | X | X | X# | X | X | X | X | X | X | X | X | X | X | X | | X |
| **Amylase, Lipase** | X | X | | | | X | | | | X | | | | X | | X |
| **Urine or Serum Pregnancy and contraception check[7]** | X | X | | | | X | | | | X | | | | X | | X |
| **Echocardiogram or MUGA** | X | | | | | | | | | | | | | X | | |
| **ECG[8]** | X | X (Pre and Post InO) | | | | X (Pre and Post InO) | | | | X (Pre and Post InO) | | | | X | | |

| Parameter | Screening | Cycle 1 | | | | Cycle 2 | | | | Cycle 3-6 | | | | End of Therapy | Follow up after EOT until CAR-T/ HSCT or disease progression | Safety follow-up |
|---|---|---|---|---|---|---|---|---|---|---|---|---|---|---|---|---|
| Day | -10 to 0 | 1 | 8 | 15 | 22 (±2 d) –day 42 | 1 | 8 (±1 day) | 15 (±1 day) | 28 (±2 day) – day 42 | 1 (±1 day) | 8 (±1 day) | 15 (±1 day) | 28 (±2 day) – day 42 | 4 w. post last InO dose (±1 week)[18] | Refer to *Table 34* for frequency of assess-ment | 10 w. post last-InO dose (±1 week)[19] |
| Chest X-ray[9] | X | | | | | | | | | | | | | | | |
| Eligibility and registration[10] | X | | | | | | | | | | | | | | | |
| LP with cell count + diff & it therapy[11] | X | | | | X | | | | X | | | | X | X | | |
| BM for flow cytometry & morph (local) | X | | | | X | | | | X | | | | X | X | | |
| BM cytogen.[12] | X | | | | | | | | | | | | | | | |
| PB and BM for MRD (sect. 7.5)[13] | X | | | | X | | | | X | | | | X | X | | |
| CD22 expression levels[13, 14] | X | | | | X[14] | | | | X[14] | | | | X[14] | X[14] | | |
| PB and BM Calicheamicin Sens.[13,15] | X | | | | | | | | | | | | | | | |
| Number of peripheral CD19+B-Cells and CD4+/CD8+ T cells[16] | X | | | | X | | | | | X (cycle 3 & 6 only) | | | | X | X | X |
| Serum IgG and IgM levels[17] | X | | | | X | | | | | X (cycle 3 & 6 only) | | | | X | X | X |
| BM (local) with MRD levels and B-cells CD19+ [20] | | | | | | | | | | | | | | | X | |

| Parameter | Screening | Cycle 1 | | | | Cycle 2 | | | | Cycle 3-6 | | | | End of Therapy | Follow up after EOT until CAR-T/ HSCT or disease progression | Safety follow-up |
|---|---|---|---|---|---|---|---|---|---|---|---|---|---|---|---|---|
| **Concomitant medication** | X | X | X | X | X | X | X | X | X | X | X | X | X | X | | X |
| **Safety evaluation adverse events** | X | X | X | X | X | X | X | X | X | X | X | X | X | X | | X |
| **InO infusion (see tables 9,10 &11)** | | X | X | X | | X | X | X | | X | X | X | | | | |
| **Follow-up for Relapse, events, survival & start of new therapy** | | | | | | | | | | | | | | X | | X |

BM=bone marrow, BSA=body surface area, CBC=complete blood count, ECG=electrocardiography LP=lumbar puncture, MRD=minimal residual disease, PB=peripheral blood, PK=pharmacokinetics, it=intrathecal

[1] Informed consent/assent must be obtained before any study specific investigations are performed

[2] Medical history including review of cancer diagnosis and previous cancer treatment (screening/pre-study visit only), current medications and any current medical conditions or abnormalities.

[3] Calculate BSA according to the Mosteller formula as given in Appendix 4

[4] Vital signs include pulse, respirations, blood pressure and temperature

[5] Performance status, see Appendix 1

[6] Chemistry Panel (including sodium, potassium, calcium, phosphate, uric acid, creatinine, urea, total protein, albumin, Total and direct bilirubin, AST, ALT, alkaline phosphatase and gamma-glutamyltranferase (GGT).

[7] Serum/Urine Pregnancy Test and Contraception Check: for women of child bearing potential, a serum or urine pregnancy test, with sensitivity of at least 25 mIU/mL, will be performed as indicated and from then on monthly until 8 months after the last InO dose, following the CTFG recommendations. A pregnancy test should also be done whenever 1 menstrual cycle is missed during treatment (or potential pregnancy is suspected). Additionally, for male and female patients who are of childbearing potential, investigators must also document method of contraception For male patients, the method of contraception should be documented monthly until 5 months after the last dose of InO, following the CTFG recommendations. See also section 7.12

[8] Pre-dose ECG should be done after pre-medications are given but before InO infusion begins. Post dose ECG should be done within 15 minutes after the end of the InO infusion. The QT interval for each exam will be recorded on the CRF.

[9] In case of suspected subclinical fungal infections perform CT-scan

[10] Registration: after confirmation of eligibility, patient number and dose level allocation will be assigned by the Sponsor.

[11] Intrathecal therapy will be given as described in Section 4.3.3 Treatment Cycles

[12] Cytogenetic analysis will be performed locally according to local standard of care guidelines, but results will be centrally reviewed by an expert cytogeneticist.

[13] Send to central laboratory (see Section 7 and procedure manual). Please note: for patients <18 Kg collect reduced blood volumes for MRD analysis.

[14] CD22-expression should be repeated in case of loss of response/progression to study clonal evolution and should be sent to the central laboratory for analysis in such cases. See Section 7.7

[15] In-vitro calicheamicin sensitivity samples will be taken at screening as described in section 7.10 and require both PB and BM, and can be sent together with the MRD samples. Please note: for subjects < 12 kg calicheamicin sensitivity will not be performed (see Section 7.10).

[16] The absolute number of peripheral blood $CD19^{+}$ B-Cells and CD4+/CD8+ T-cells should be measured every 2 weeks in Stratum 3 patients after the end of therapy and until disease progression or HSCT/CAR-T cell therapy as detailed in as detailed in ***Table 34***..Lymphocytes are selected on the basis of CD45/side scatter dot plot. T and B lymphocytes are selected by CD3 and CD19 expression respectively from CD45-gated lymphocytes. CD4 and CD8 are then analyzed from CD3+ lymphocytes. Central Memory T cells are to be gated depending

on the CD62L and CD45RA expression from CD4+ or CD8+ T cells. (CD45RA- CD62L+). Further details for monitoring during follow-up are provided in ***Table 34.*** For patients <12Kg the immune-activity analysis (CD19+, IgG and IgM) will be excluded.

[17] Serum IgG and IgM should be measured monthly in Stratum 3 patients, as detailed in ***Table 34***. For patients <12 Kg the immune-activity analysis (CD19+, IgG and IgM) will be excluded.

[18] The end-of-treatment (EOT) visit should occur approximately 4 weeks after the last dose of InO. However, in the event that a patient requires initiation of a new anti-cancer therapy, the EOT visit should be performed before the initiation of the new anti-cancer therapy as close as possible to 4 weeks after the last dose of study drug.

[19] Note that adverse events, safety laboratory tests and associated concomitant medications must continue to be collected in the CRF through at least 10 weeks after the last dose of study therapy unless a new anti-cancer therapy is given.[20.] After EOT and prior to CAR-T cell infusion or HSCT with frequency at investigator discretion as detailed in **Table 34**.

*# Liver tests (total bilirubin and AST and/or ALT levels) need to be determined prior to day 8 infusion of InO. In case of a >2.5 ULN elevation in AST and/or ALT, or >1.5 xULN for total bilirubin, delay the InO dose according to Section 4.4.1 (excluding cases where these aberrations are due to hemolysis or Gilbert's syndrome).*

## 7.5 Schedule of Events for Stratum 2 patients with other CD22-positive B-cell malignancies.

***Table 26: Schedule of Events for Stratum 2 patients with other CD22-positive B-cell malignancies.***
*Further follow-up is described in* ***Table 33.***

| **Parameter** | **Screening** | **Cycle 1** | | | | **Cycle 2** | | | | **Cycle 3-6** | | | | **End of Therapy** | **Safety follow-up** |
|---|---|---|---|---|---|---|---|---|---|---|---|---|---|---|---|
| **Day** | **-10 to 0** | **1** | **8** | **15** | **22 (±2 day)-42** | **1 (±1 day)** | **8 (±1 day)** | **15 (±1 day)** | **28 (±1 day) – day 42** | **1 (± 1 day)** | **8 (± 1 day)** | **15 (± 1 day)** | **28 (± 2 day) – day 42** | **4 w. post last InO dose (± 1 week)[18]** | **10 w. post last-InO dose (± 1 week)[19]** |
| **Inform. consent /assent [1]** | X | | | | | | | | | | | | | | |
| **Med. History[2]** | X | | | | | | | | | | | | | | |
| **Full Clinical Examination** | X | X | X | X | X | X | | | | X | | | | X | X |
| **Height, weight, BSA[3]** | X | X | | | | X | | | | X | | | | | |
| **Vital signs[4]** | X | X | X | X | X | X | X | X | | X | X | X | | X | X |
| **Performance Status[5]** | X | X | | | | X | | | | X | | | | X | X |
| **Menarchal status** | X | X | | | | X | | | | X | | | | X | X |
| **CBC and differential** | X | X (and day 4) | X | X | X | X | X | X | X | X | X | X | X | X | X |
| **Chemistry Panel[6]** | X | X | X[#] | X | X | X | X | X | X | X | X | X | X | X | X |
| **Amylase, Lipase** | X | X | | | | X | | | | X | | | | X | X |
| **Urine or Serum Pregnancy and contraception check[7]** | X | X | | | | X | | | | X | | | | X | X |

| Parameter | Screening | Cycle 1 | | | | Cycle 2 | | | | Cycle 3-6 | | | | End of Therapy | Safety follow-up |
|---|---|---|---|---|---|---|---|---|---|---|---|---|---|---|---|
| **Day** | **-10 to 0** | **1** | **8** | **15** | **22 (±2 day)-42** | **1 (±1 day)** | **8 (±1 day)** | **15 (±1 day)** | **28 (±1 day) – day 42** | **1 (± 1 day)** | **8 (± 1 day)** | **15 (± 1 day)** | **28 (± 2 day) – day 42** | **4 w. post last InO dose (± 1 week)[18]** | **10 w. post last-InO dose (± 1 week)[19]** |
| **Echocardiogram or MUGA** | X | | | | | | | | | | | | | X | |
| **ECG[8]** | X | X (Pre and Post InO) | | | | X (Pre and Post InO) | | | | X (Pre and Post InO) | | | | X | |
| **Chest X-ray[9]** | X | | | | | | | | | | | | | | |
| **Radiological investigations[10]** | X | | | | X | | | | X | | | | X (cycle 4 and 6 only) | X | |
| **Eligibility and registration[11]** | X | | | | | | | | | | | | | | |
| **LP with cell count + diff & it therapy[12]** | X | X | | | X | | | | X | | | | X | X | |
| **BM for flow & morphology** | X | | | | X | | | | X | | | | X (cycle 4 and 6 only) | X | |
| **BM cytogen.[13]** | X | | | | | | | | | | | | | | |
| **CD22 expression levels[14,15]** | X | | | | X[15] | | | | X[15] | | | | X[15] (cycle 4 and 6 only) | X[15] | |
| **PK[14,16]** | | X | X | X | X | X | X | X | X | X[16] (cycle 3 only) | X[16] (cycle 3 only) | X[16] (cycle 3 only) | X[16] (cycle 3 only) | | |

| Parameter | Screening | Cycle 1 | | | | Cycle 2 | | | | Cycle 3-6 | | | | End of Therapy | Safety follow-up |
|---|---|---|---|---|---|---|---|---|---|---|---|---|---|---|---|
| **Day** | **-10 to 0** | **1** | **8** | **15** | **22 (±2 day)-42** | **1 (±1 day)** | **8 (±1 day)** | **15 (±1 day)** | **28 (±1 day) – day 42** | **1 (± 1 day)** | **8 (± 1 day)** | **15 (± 1 day)** | **28 (± 2 day) – day 42** | **4 w. post last InO dose (± 1 week)[18]** | **10 w. post last-InO dose (± 1 week)[19]** |
| **Immunogenicity samples[14,17] ADA (NOT valid for patients enrolled after Amendment 4)** | X | | | | | X | | | | X | | | | X | |
| **Number of CD19+ B-cells and serum Ig levels[18]** | X | | | | X | | | | | X (cycle 3 & 6 only) | | | | X | |
| **Concomitant medication** | X | X | X | X | X | X | X | X | X | X | X | X | X | X | X |
| **Safety evaluation adverse events** | X | X | X | X | X | X | X | X | X | X | X | X | X | X | X |
| **InO infusion (see Tables 6 & 9)** | | X | X | X | | X | X | X | | X | X | X | | | |
| **Follow-up for Relapse, events, survival & start of new therapy[19]** | | | | | | | | | | | | | | X | X |

BM=bone marrow, BSA=body surface area, CBC=complete blood count, ECG=electrocardiography LP=lumbar puncture, MRD=minimal residual disease, PB=peripheral blood, PK=pharmacokinetics, it=intrathecal

[1] Informed consent/assent must be obtained before any study specific investigations are performed

[2] Medical History including review of cancer diagnosis and previous cancer treatment (screening/pre-study visit only), current medications and any current medical conditions or abnormalities.

[3] Calculate BSA according to the Mosteller formula as given in Appendix 4

[4] Vital signs include pulse, respirations, blood pressure and temperature

[5] Performance status, see Appendix 1.

[6] Chemistry Panel (including sodium, potassium, calcium, phosphate, uric acid, creatinine, urea, total protein, albumin, Total and direct bilirubin, AST, ALT, alkaline phosphatase and

gamma-glutamyltranferase (GGT).

[7] Serum/Urine Pregnancy Test and Contraception Check: For women of child bearing potential, a serum or urine pregnancy test, with sensitivity of at least 25 mIU/mL, will be performed as indicated and from then on monthly until 8 months after the last InO dose, following the CTFG recommendations. A pregnancy test should also be done whenever 1 menstrual cycle is missed during treatment (or potential pregnancy is suspected). Additionally, for male and female patients who are of childbearing potential, investigators must also document method of contraception. For male patients, the method of contraception should be documented monthly until 5 months after the last dose of InO, following the CTFG recommendations. See also section 7.12

[8] Pre-dose ECG should be done after pre-medications are given but before InO infusion begins. Post dose ECG should be done within 15 minutes after the end of the InO infusion. The QT interval (QTcF) for each exam will be recorded on the CRF.

[9] In case of suspected subclinical fungal infections perform CT-scan

[10] Radiological investigations:

- Chest x-ray (PA and lateral) and abdominal ultrasound
- Chest and Abdominal CT are recommended according to the tumor localization
- CT scan and/or MRI in case of head and neck tumors
- Bone scan if clinically applicable (or PET(-CT) when available), and x-ray or MRI of the suspected areas
- Cranial and/or spinal MRI if clinically applicable

During follow-up only repeat radiological investigations which are abnormal at baseline. Please use the same method of assessment for each evaluation.

[11] Registration: after confirmation of eligibility, patient number and dose level allocation will be assigned by the Sponsor.

[12] Intrathecal therapy will be given as described in Section 4.3.3.

[13] Only in case of PB/BM-involvement at the discretion of the investigator. Cytogenetic analysis will be performed locally according to local standard of care guidelines, but results will be centrally reviewed by an expert cytogeneticist.

[14] Send to central laboratory (see Section 7 and procedure manual). This can either be performed in case of a liquid tumor on bone-marrow and peripheral blood or a tumor biopsy.

[15] CD22-expression should be repeated in case of loss of response/progression to study clonal evolution and should be sent to the central laboratory for analysis in such cases. See Section 7.7, for details on liquid or tumor biopsies.

[16] Send to central laboratory (see Section 7 and procedure manual). PK samples should be collected in course 1, 2 and 3. For patients <12 Kg see the specific PK schedule, as detailed in Section 7.8

[17] Immunogenicity samples should be taken as described in Section 7.9

[18] The absolute number of peripheral blood $CD19^+$ B-Cells and serum immunoglobulin levels (IgG and IgM) should be measured every 3 months after the end of therapy until disease progression or HSCT/CAR-T cell therapy or maximum of one year whichever occurs first. This will not be done in patients below 12 kg of body weight to reduce blood volume.

[18.] The end-of-treatment (EOT) visit should occur approximately 4 weeks after the last dose of InO. However, in the event that a patient requires initiation of a new anti-cancer therapy, the EOT visit should be performed before the initiation of the new anti-cancer therapy as close as possible to 4 weeks after the last dose of study drug.

[19] Note that adverse events, safety laboratory tests and associated concomitant medications must continue to be collected in the CRF through at least 10 weeks after the last dose of study therapy unless a new anti-cancer therapy is given.

*# Liver tests (total bilirubin, AST and/or ALT levels) need to be determined prior to day 8 infusion. In case of a >2.5 ULN elevation in AST and/or ALT, or >1.5 xULN for total bilirubin, delay the dose according to Section 4.4.1 (excluding cases where these aberrations are due to hemolysis or Gilbert's syndrome).*

## 7.6 Samples for Minimal Residual Disease (MRD) testing (BCP-ALL patients only)

### 7.6.1 MRD Instructions for BCP-ALL patients (stratum 1A, 1B/1B-ASP and Phase 2 and Stratum 3 (VHR) patients)

Participation in these procedures is required.

MRD assessment is considered standard of care in Europe and will be performed centrally for all European sites at the Department of Immunology, Erasmus MC, and coordinated by Dr. Vincent van der Velden. Sample shipment towards the Erasmus MC will be organised by the Central Laboratory for Clinical Trials of the Princess Maxima Center. Tubes and shipment boxes will also be provided by the Central lab of the Princess Maxima Center. MRD results will be forwarded to the sites for clinical management.

Both multi-parameter flow cytometry and RQ-PCR will be performed as methods for MRD detection. The first to study patterns of clonal evolution under CD22-directed treatment, and the latter as gold standard for MRD-based clinical decision-making in Europe.

After cycle 1, MRD will be analyzed in BM and PB using multiparameter flow cytometry only. Results of RQ-PCR (BM only) will become available after cycle 2 (including the RQ-PCR MRD level after cycle 1 and cycle 2).

***Table 27: Instructions to perform MRD monitoring in BCP-ALL patients***

| | |
|---|---|
| **Samples requested for MRD testing:** | **Bone Marrow and Peripheral Blood**<br>• Pre-study for stratum 1A, 1B/1B ASP, Phase 2 Cohort and for Stratum 3 (VHR) (all ALL patients)<br>• End of cycle 1, 2, 3, 4 and 5, 6 and EOT for stratum 1A, 1B/1BASP;Phase 2 Cohort and for Stratum 3 (VHR) |
| **MRD results** | After cycle 1: BM and PB flow MRD<br>After cycle 2: BM and PB flow MRD + RQ-PCR BM MRD levels after cycle 1 and cycle 2<br>After each subsequent cycle: BM flow and RQ-PCR levels. |
| **Bone marrow Collection procedure:** | Please use the tubes in the packages that have been provided to you by the Princess Máxima Center.<br><br>Collect minimum of 2 ml of marrow into a syringe and transfer the specimen immediately in a preservative free sodium heparin vacutainer (orange top). Use multiple syringes and tubes as needed. Reposition marrow aspirate needle as least once during procedure to ensure the maximum quality of marrow. Mix sample well.<br><br>If at the end of the cycle the patient has a peripheral absolute blast count of at least 2500/uL the site can send 5 ml of peripheral blood in a green top heparin tube in place of bone marrow. Please note: for patients <18 Kg take 50% of blood volume requested for MRD analysis. |
| **Peripheral Blood collection procedure:** | Please use the tubes in the packages that have been provided to you by the Princess Maxima Center.<br><br>Collect minimum of 5 ml of peripheral blood into a syringe and transfer the specimen immediately in a preservative free sodium heparin vacutainer (orange top). Mix sample well. Please note: for patients <18 Kg take 50% of blood volume requested for MRD analysis.<br><br>If at the end of the cycle the patient has a peripheral absolute blast count of at least 2500/uL the site can send 5 ml of peripheral blood in a green top heparin tube in place of bone marrow. Please note: for patients <18 Kg take 50% of blood volume requested for MRD analysis. |
| **Specimen Labeling:** | Each tube must be labeled with the study ID number, date of birth and the date the sample was obtained. |

| | |
|---|---|
| **Specimen Packaging and Shipping:** | For specimen shipping, the Central Lab of the Princess Maxima Center will arrange the transport logistics and provide you with details on the courier pick up time. Please use the shipping boxes and "specimen shipping forms" that have been provided to the participating centers. Samples need to be sent at room temperature.<br><br>Preferably send samples from Mondays to Thursdays, so that they arrive at Erasmus MC on Friday before noon.<br><br>Please send the completed "Specimen Shipping Form" by E-mail to: trials.researchlab@prinsesmaximacentrum.nl. A confirmation will be sent to you by e-mail. In case you need to clarify details: please call the laboratory at +31-88-9725071.<br><br>The delivery address on the box should read:<br>Erasmus MC Department of Immunology<br>Wytemaweg 80<br>3015 CN Rotterdam, The Netherlands<br>Att: Dr. V.H.J. van der Velden / LLD<br>Room no. Nb-1218<br>Phone: +31-10-7044090 / +31-10 704 4084 |

## 7.7 Biology samples: CD22 Expression and Saturation Kinetics

### 7.7.1 CD22 expression analysis (all patients)

Participation in these procedures is required.

At baseline, immunophenotyping (applying 8 or 10 color staining) will be performed in order to confirm the presence of CD22 expression levels on the blast cells using flow cytometry, applying the original antibody used for the design of CMC-544. A G5/44-PE-conjugated antibody will be used, and data will be quantified using bead standards. Leukaemic blast cells present **in the bone marrow** sample will be identified by 8- or 10-color immunophenotyping using a combination of antibodies, including CD22, CD45, CD19, CD10, CD34 and CD20.

Separate sampling is *not* necessary, since the BM and PB samples obtained at screening for MRD (see Section 7.6) will also be used for evaluation of CD22 expression levels.

**Note that** in case of NHL patients a biopsy may be sent; please put the biopsy material in a wet gauze in a closed container (the biopsy should not dry out during transport).

### 7.7.2 CD22 saturation analysis (stratum 1A, and Phase 2 cohort only)

Participation in these procedures is required, however **NOT** for Stratum 1B/1B-ASP and Stratum 3 patients or patients <12 kg (to reduce sample volume in small children; see table below), and with some samples skipped for patients <18 kg body weight.

CD22 saturation will be evaluated using a flow cytometric assay applying a biotinylated anti-human IgG4 antibody, comparable to the one used for Mylotarg.[90,92] In order to determine the most effective dosing schedule of InO, saturation analysis should be performed after drug infusions on day 1

and day 8 of cycle 1 in **PB**. Later time points and cycles may not be informative due to the lack of circulating ALL cells in the responding cases. The day 8 t = 0 h sample can be used to determine whether CD22 saturation remaining from the previous infusion is still present.

For these analyses, peripheral blood samples will be taken just prior to the first and second infusions of InO, and 1 hour after start of each infusion, hence at the end of the infusion (a total of 4 samples).

***Table 28: Instructions to perform CD22 saturation analysis***

| | |
|---|---|
| **Samples requested for CD22 saturation analysis** | **<u>Peripheral Blood Samples</u>**<br><br>**<u>Cycle 1, Day 1</u>** (These samples will be omitted for patients <12 Kg)<br>• Prior to the start of the InO infusion<br>• At the end of the infusion (max + 15 min)<br><br>**<u>Cycle 1, Day 8</u>** (These sample will be omitted for patients <18 Kg)<br>• Prior to the start of the InO infusion<br>• At the end of the infusion (max + 15 min) |
| **Peripheral blood collection procedure** | Collect a minimum of 3-5 ml (max 1.5 ml for patients <18 Kg) of whole blood in a preservative free sodium heparin vacutainer (to be taken from hospital stock). Store the tube after sampling immediately at 4°C, do NOT freeze.<br><br>For patients of 12-18 Kg: at day 1 collect 1.5 ml per assessment; at day 8, skip the sample collection<br>For patients <12 kg: the CD22 saturation assessment is excluded. |
| **Specimen Labeling** | Each tube must be labeled with the study ID number, date of birth, and the date the sample was obtained. |

| Specimen Packaging and Shipping: | Ship samples (prior to infusion and at end of infusion sample together) at 4°C, using cool packs. Please be sure NOT to use frozen ice packs since samples may freeze. On the other hand cold transport is required in order to avoid ongoing InO metabolism.<br>Samples obtained after cycle 1 day 1 should be shipped immediately after obtaining the second sample; samples obtained after cycle 1 day 8 should be shipped immediately after obtaining the second sample.<br><br>For specimen shipping, the Central Lab of the Princess Maxima Center will arrange the transport logistics and provide you with details on the courier pick up time. Please use the “specimen shipping forms” that have been provided to the participating centers. Samples need to be sent at 4°C, do NOT freeze.<br><br>Preferably send samples from Monday to Thursday, so that they arrive at Erasmus MC on Friday before noon.<br><br>Please send the completed “Specimen Shipping Form” by E-mail to: trials.researchlab@prinsesmaximacentrum.nl.<br>A confirmation will be sent to you by e-mail. In case you need to clarify details: please call the laboratory at +31-88-9725071.<br><br>The delivery address is:<br>Erasmus MC<br>Department of Immunology<br>Wytemaweg 80<br>3015 CN Rotterdam<br>The Netherlands<br><br>Att: Dr. V.H.J. van der Velden / LLD<br>Room no. Nb-1218<br>Phone: +31-10-7044090 / +31-10 704 4084 |
|---|---|

## 7.8 Pharmacokinetic Samples (Stratum 3 patients excluded)

Participation in these procedures is required (except for patients enrolled in Stratum 3 VHR cohort)

Concentrations of InO (as parent drug PF-05208773) and unconjugated calicheamicin (as NAc-gamma calicheamicin DMH) will be determined in serum (not plasma) by validated, high sensitivity LCMS assays. Bioanalytical analyses will be performed at a central laboratory designated by Pfizer.

Bioanalytical measures will be combined with respective individual patient dosing history and demographic information. Pharmacometric analysis will be performed by Pfizer and supplemental analysis (for instance additional PK-PD modelling) by Dr. Alwin Huitema (pharmacist at the Dutch Cancer Institute), using an appropriate model-based approach. Pharmacokinetic parameters may serve as input for correlation to biomarker measures, and other endpoints, as appropriate. Data in adults have shown that InO exhibits both linear- and time-dependent components of clearance. By cycle 4, steady state is achieved and the linear component of clearance predominates. Therefore, PK samples will be taken during cycle 1, 2 and 3 to also characterize the linear- and time-dependent component of clearance.

In order to correctly utilize the pharmacokinetic data, it is critical that the actual time and date of InO administration and of pharmacokinetic sample collection is recorded on the case report form.

For blood collection procedure, specimen labeling and specimen packaging and shipping, please refer to the procedure manual.

***Table 29. Cycle 1. Sampling schedule for PK of InO***

| **Study Visit** | **Cycle 1 Day 1** | | **Cycle 1 Day 8** | **Cycle 1 Day 15** | | **Cycle 1 Day 22 [6] (± 2 days)** |
|---|---|---|---|---|---|---|
| **Hour (post-dose)** | **Pre-Dose** | **1 hr (± 15 min)*** | **Pre-Dose** | **Pre-Dose** | **1 hr (± 15 min)*** | **Trough sample** |
| Whole blood collection for InO PK[2] | X[1,4] | X | X[1] | X[1] | X | X[5, 6] |
| Whole blood collection for Unconjugated Calicheamicin PK[3] | X[1,4] | | | | X | |

[1] Prior to the start of the InO infusion.
[2] For InO assay, 1.5 ml of whole blood/timepoint is required (total 9 ml)
[3] For calicheamicin assay, 1 ml of whole blood / timepoint is required (total 2 ml)
[4] These samples will be skipped for patients <12 Kg body weight to reduce blood volume withdrawn from the patient
[5] For patients <18 Kg, trough PK samples for INO on day 22 Cycle 1 will be skipped to reduce blood volume.
[6] For Stratum 1B/1B-ASP this sample can be collected during the visit scheduled on day 24.
* 1 hr sample is collected right after the end of the infusion

***Table 30. Cycle 2. Sampling schedule for PK of InO***

| **Study Visit** | **Cycle 2 Day 1** | **Cycle 2 Day 8 (± 1 day)** | **Cycle 2 Day 15 (± 1 day)** | | **Cycle 2 Day 28 (± 2 days)** |
|---|---|---|---|---|---|
| **Hour (post-dose)** | **1 hr (± 15 min)*** | **Pre-Dose** | **Pre-Dose** | **1 hr (± 15 min)*** | **Trough sample** |
| Whole blood collection for InO PK[2] | X[4] | X[1] | X[1] | X | X |
| Whole blood collection for Unconjugated Calicheamicin PK[3] | | | | X | |

[1] Prior to the start of the InO infusion
[2] For InO assay, 1.5 ml of whole blood / timepoint is required (total 7.5 ml)
[3] For unconjugated calicheamicin assay, 1 ml of whole blood / timepoint is required (total 1 ml)
[4] For BCP-ALL patients of <18 Kg: C2D1 PK sample 1 hours after the end of the infusion will be skipped to reduce the blood volume withdrawn.
* 1 hr sample is collected right after the end of the infusion

***Table 31. Cycle 3. Sampling schedule for PK of InO***

| **Study Visit** | **Cycle 3 Day 1** | **Cycle 3 Day 8 (± 1 day)** | **Cycle 3 Day 15 (± 1 day)** | **Cycle 3 Day 28 (± 2 days)** |
|---|---|---|---|---|
| **Hour (post-dose)** | **1 hr (± 15 min)*** | **Pre-Dose** | **Pre-Dose** | **Trough sample** |
| Whole blood collection for InO PK[2,3] | X[4] | X[1] | X[1] | X |

[1] Prior to the start of the InO infusion
[2] For InO assay, 1.5 ml of whole blood/timepoint is required (total 6 ml)
[3] Unconjugated calicheamicin will not be tested during cycle 3
[4] For BCP-ALL patients of <18 Kg body weight, the C3D1 PK sample 1 hours after the end of the infusion will be skipped.
* 1 hr sample is collected right after the end of the infusion

### 7.9 Immunogenicity (for patients treated with single-agent InO only)

Participation in these procedures is required for Stratum 1A, and the Phase 2 cohort, however not in Stratum 1B/1B-ASP, and Stratum 3 patients. Stratum 2 patients will not participate anymore in these procedures starting from Protocol amendment v 4.0, due to the fact that the analysis on collected samples will be already performed while the enrollment of Stratum 2 patients will continue.

An electrochemiluminescent (ECL) bridging assay supporting analysis of immunogenicity samples for clinical studies in patients with relapsed/refractory ALL (Phase 1/2 Study B1931010 and Phase 3 Study B1931022) was developed and validated by Questpharma (Newark, Delaware, US). Using the ECL methodology, a positive ADA response at any time was observed in 6/219 (3%) patients. No patients who tested positive for ADA were positive for neutralizing anti-InO antibodies. In patients who tested positive for ADA, InO clearance did not appear to be affected.

For the present study, a 2 ml sample of blood for serum will be collected during the Screening visit, prior to each cycle of treatment from cycle 2 to 6, and at the End of Treatment study visit. Upon harvesting the serum, samples will be stored at -70 °C until they are shipped to the sponsor-designated analytical laboratory.

Samples for immunogenicity will be tested using a 3 tier strategy according to current industry practices and regulatory guidelines. The samples will be first tested in a screening assay. Samples that are ADA-positive at screening will undergo confirmatory testing and subsequent titer determination if confirmed positive. Characterization of the specificity of the immunogenic responses to the native antibody (G544) and calicheamicin payload will be conducted using the competitive ECL assay.

If the sample is confirmed positive for ADAs, the neutralizing capacity of the ADAs will be tested using a CellTiter-Glo® Luminescent Cell Viability Assay.

For blood collection procedure, specimen labeling and specimen packaging and shipping, please refer to the procedure manual.

### 7.10 In-vitro Calicheamicin Resistance

Participation in these procedures is required for all BCP-ALL patients receiving single-agent InO (not those in Stratum 1B).
For BCP-ALL subjects weighing <12 kg, calicheamicin sensitivity will not be performed.

Cellular sensitivity to the cytotoxic compound calicheamicin may influence response to treatment with InO. In an earlier publication we reported on differential sensitivity of AML and ALL cells to this compound, with ALL cells being relatively sensitive to calichemicin [62]. However, some ALL samples displayed resistance for which the clinical relevance is yet unknown. The present study set-up enables us to determine the predictive value of ex-vivo sensitivity/resistance to calicheamicin for the clinical response to InO. To do so, a leukemic cell sample taken prior to the start of the InO infusion needs to be sent to the Erasmus MC laboratory. In principle, this can be the same bone marrow tube as used for MRD detection. MRD studies will have highest priority and left-over material will be used for this calicheamicin cytotoxicity testing (by MTT-assay). In addition, we will collect DNA and RNA out of these samples for optional molecular profiling to elucidate causes of cellular resistance to calicheamicin.

***Table 32. In-vitro Calicheamicin Resistance***

| | |
|---|---|
| **Samples requested:** | **<u>Pre-study Bone Marrow and Peripheral Blood (see section 7.6.1)</u>**<br>Heparin tubes (orange top), 1 ml bone marrow and 5 ml of peripheral blood.<br><br>This can be combined with PB and BM for MRD and CD22 expression.<br><br>This is NOT done in patients below 12 Kg body weight to reduce sampling volume. |
| **Specimen Labeling:** | See MRD Section 7.6. |
| **Specimen Packaging and Shipping:** | See MRD Section 7.6. |

### 7.11 End of Treatment and Week 10 post last InO dose safety visit

The end-of-treatment (EOT) visit is required for all patients. It should occur approximately 4 weeks (+/- 1 week) after the last dose of InO . However, in the event that a patient requires initiation of a new anti-cancer therapy, the end-of-treatment visit, including procedures, should be performed before the initiation of the new anti-cancer therapy as close as possible to 4 weeks after the last dose of study drug. Adverse events, safety laboratory tests and associated concomitant medications must continue to be collected in the CRF through at least **10 weeks** after the last dose of study therapy unless a new anti-cancer therapy is given. These data will be collected at the week 10 (+/-1week) post last InO-dose safety visit.

### 7.12 Follow-up for all patients

All patients will be followed for a maximum of 3 years after enrollment.

Patients will have follow-up data collected in the eCRF as detailed in ***Table 33*** and ***Table 34***. The purpose is to assess InO-related adverse events, remission status, administration of HSCT and/or other therapies, and survival.

Follow-up data will be collected monthly until 12 months after last InO dose. The following data will be collected:

- Disease status information.
- Anti-cancer therapy received after completion of study therapy.
- Information about possible stem cell transplant: conditioning regimen (dosages of chemotherapy, antibodies and/or TBI), GVHD prophylaxis, date of HSCT, stem-cell source, donor type, date of hematopoetic regeneration (defined as ANC >500 x 3 consecutive days).
- Information about VOD/SOS regardless of attribution, including prophylaxis and treatment (see Appendix 3 for a clinical definition) and whether VOD occurred concurrent with serious infection (i.e. sepsis, viral or fungal infection). Note that VOD/SOS cases will be recorded in the CRF and reported as an SAE for up to 1 year after enrollment regardless if new anti-cancer therapy is given.
- Date and cause of death.

If there are any ongoing adverse events (SAEs) that are attributed to the study drug, the patient will be followed monthly after the week 10 post-InO safety visit (as detailed in Schedule of Events, Section 7.1-7.5), until either the start of subsequent anti-leukemic therapy including conditioning for HSCT, or disease progression, or for 1 year post-last InO dose, whichever occurs first (**see *Table 33* and *Table 34*)**. In the event that the patient starts another anti-tumor treatment the date the subsequent therapy started should be recorded.

Peripheral blood $CD19^+$ B-Cells and immunoglobulin (IgM and IgG) levels should be measured every 3 months until HSCT or CAR-T cells therapy, or maximum 1 year after InO, except for Stratum 3 patients (VHR) due to a specific more frequent follow-up is planned. See ***Table 33*** and ***Table 34***.

***Table 33. Follow up for all patients except patients in Stratum 3 (see Table 34 )***

| All patients | Follow-up<br>Until 12 months post last InO dose<br>(starting after week 10 safety visit) | Follow-up[3]<br>Year 2 and 3 after end of treatment |
|---|---|---|
| **Safety evaluation adverse events** | Related SAEs &.<br>VOD/SOS **monthly** (±7 days)[1] | Every 3 months (±14 days) |
| **Number of peripheral B-cells** [2] | Every 3 months (not for Stratum 1B/1B-ASP)[2] | |
| **Serum Ig A, G and M levels** | Every 3 months (not for Stratum 1B/1B-ASP)[2] | |
| **Follow-up for Relapse, events, survival & start of new therapy** | X[1] | X[3] |
| **Serum/Urine Pregnancy Test and Contraception Check** | X[4] | |

1 The following data will be collected monthly during the first 12 months after last InO dose:
• Disease status information.
• Anti-cancer therapy received after completion of study therapy.
• Information about possible stem cell transplant: conditioning regimen (dosages of chemotherapy, antibodies and/or TBI), GVHD prophylaxis, date of HSCT, stem-cell source, donor type, date of hematopoetic regeneration (defined as ANC >500 x 3 consecutive days)
• Information about VOD/SOS regardless of attribution, including prophylaxis and treatment (see Appendix 3 for a clinical definition) and whether VOD occurred concurrent with serious infection (i.e. sepsis, viral or fungal infection). Note that VOD/SOS cases will be recorded in the CRF and reported as an SAE for up to 1 year after enrollment regardless if new anti-cancer therapy is given.
• All adverse events thought to be related treatment on this protocol.
• Date and cause of death.

2 The absolute number of peripheral blood $CD19^+$ B-Cells and serum immunoglobulin levels (IgG and IgM) should be measured every 3 months after the end of therapy until disease progression or HSCT/CAR-T cell therapy or maximum for one year, whichever occurs first. This is not done in patients below 3 years of age and for patients treated with InO in combination with chemotherapy (Stratum 1B/1B-ASP).

3 Follow-up will continue for a maximum of 3 years after end of treatment. Patients will have follow-up data collected in the eCRF every 3 months. The purpose is to assess occurrence of relapse, administration of alternative therapies, and survival and date and cause of death. In case the patient does not visit the hospital, follow-up can also be done by telephone or E-mail.

4 Serum/Urine Pregnancy Test and Contraception Check: For women of child bearing potential, a serum or urine pregnancy test, with sensitivity of at least 25 mIU/ml, will be performed monthly until 8 months after the last InO dose, following the CTFG recommendations. Additionally, for male and female patients who are of childbearing potential, investigators must also document method of contraception. For male patients, the method of contraception should be documented monthly until 5 months after the last dose of InO, following the CTFG recommendations.

*Table 34 Specific follow up for patients in Stratum 3*

| VHR patients | Follow up starting after EOT until disease progression or HSCT/CAR-T cell therapy | Follow-up Until 12 months post last InO dose (starting after week 10 safety visit) | Follow-up[3] Year 2 and 3 after end of treatment |
|---|---|---|---|
| **Safety evaluation adverse events** | | Related SAEs &. VOD/SOS **monthly** (±7 days)[1] | Every 3 months (±14 days) |
| **Number of B-cells and T-cells** [2] | Every 2 weeks | | |
| **Serum IgG and M levels** | Monthly | | |
| **BM[5] with MRD levels and B-cell CD19+** | With frequency at investigator discretion | | With frequency at investigator discretion |
| **Follow-up for Relapse, events, survival & start of new therapy** | | X[1] | X[3] |
| **Serum/Urine Pregnancy Test and Contraception Check** | | X[4] | |

[1] The following data will be collected monthly during first 12 months after last InO dose:

- Disease status information.
- Anti-cancer therapy received after completion of study therapy.
- Information about possible stem cell transplant: conditioning regimen (dosages of chemotherapy, antibodies and/or TBI), GVHD prophylaxis, date of HSCT, stem-cell source, donor type, date of hematopoetic regeneration (defined as ANC >500 x 3 consecutive days)
- Information about VOD/SOS regardless of attribution, including prophylaxis and treatment (see Appendix 3 for a clinical definition) and whether VOD occurred concurrent with serious infection (i.e. sepsis, viral or fungal infection). Note that VOD/SOS cases will be recorded in the CRF and reported as an SAE for up to 1 year after enrollment regardless if new anti-cancer therapy is given.
- All adverse events thought to be related treatment on this protocol.
- Date and cause of death.

[2] The absolute number of peripheral blood $CD19^+$ B-Cells and CD4+/CD8+ T-cells should be measured every 2 weeks in Stratum 3 patients after the end of therapy until disease progression or HSCT/CAR-T cells therapy or maximum for one year, whichever occurs first. Lymphocytes are selected on the basis of CD45/side scatter dot plot. T and B lymphocytes are selected by CD3 and CD19 expression respectively from CD45-gated lymphocytes. CD4 and CD8 are then analysed from CD3+ lymphocytes. Central Memory T cells are to be gated depending on the CD62L and CD45RA expression from CD4+ or CD8+ T cells (CD45RA- CD62L+). Serum immunoglobulin levels (IgG, IgM) should be measured monthly after the end of therapy until disease progression or HSCT/CAR-T cells therapy or maximum for one year, whichever occurs first.This is not done in patients below 3 years of age.

[3] Follow-up will continue for a maximum of 3 years after end of treatment. Patients will have follow-up data collected in the eCRF every 3 months. The purpose is to assess occurrence of relapse, administration of alternative therapies, and survival and date and cause of death. In case the patient does not visit the hospital, follow-up can also be done by telephone or E-mail.

**[4]** Serum/Urine Pregnancy Test and Contraception Check: For women of child bearing potential, a serum or urine pregnancy test, with sensitivity of at least 25 mIU/ml, will be performed monthly until 8 months after the last InO dose, following the CTFG recommendations. Additionally, for male and female patients who are of childbearing potential, investigators must also document method of contraception. For male patients, the method of contraception should be documented monthly until 5 months after the last dose of InO, following the CTFG recommendations.

**[5]** BM samples collected after the EOT will be analysed locally and not sent to the central lab, all available data will be collected.

# 8.0 CRITERIA FOR REMOVAL FROM PROTOCOL THERAPY, OFF STUDY CRITERIA AND STUDY TERMINATION

## 8.1 Criteria for removal of subjects from protocol therapy (End of treatment)

- Completion of 6 cycles of protocol therapy or 3 cycles if proceeding to allogeneic HSCT
- Dose Limiting Toxicity (for patients who are benefiting from study treatment, a case by case decision will be made by the Sponsor and Steering Committee regarding the possibility to continue the study therapy)
- M3 marrow any time after the completion of Cycle 1 of therapy
- Patient is not M1 after the completion of cycle 2
- Patient develops CNS involvement despite CNS directed therapy or fails clearance of CSF
- Progressive disease as defined in Appendix 2
- Relapse in any site following remission
- Patient/parent withdrawal or refusal
- Non-compliance with protocol regimen and procedures
- Investigator determination
- Female patient planning pregnancy, becomes pregnant or begins breast-feeding
- Study drug is no longer available

## 8.2 Off study criteria (End of study)

- Death
- Patient withdraws consent, refuses follow-up
- Patient has completed protocol treatment and the required follow-up period
- Patient Lost to follow-up

### 8.2.1 Patient withdrawal

Patients may withdraw from study treatment at any time at their own request or at the request of their legal guardian. Patients may be withdrawn from study treatment at any time at the discretion of the treating Investigator for safety or compliance reasons. If a patient does not return for a scheduled visit, every effort should be made to contact the patient. Every effort should be made to document patient outcome where possible.

If a patient expresses interested in withdrawing from the study the Investigator should:

- Request the patient return for a final visit, if applicable
- Follow up with the patient regarding any unresolved adverse events
- Perform a physical exam on the patient (including height, weight and blood pressure/pulse)
- Arrange for disease assessment and all safety labs (full blood count, biochemistry) if the patient agrees to this
- Ask for consent for study personnel to collect important safety data (i.e. occurrence of VOD) and survival follow-up. Such results could be obtained by the treating physician.

### 8.2.2 Withdrawal of consent

Patients/parents/legal guardians may withdraw consent at any time during the trial. The details of the withdrawal should be clearly documented and communicated to the DCOG-ECTC Trials Office.

There are three types of withdrawal as detailed below:

- Patient or their parent(s)/legal guardian would like to withdraw the patient from the trial, but is willing for the patient to be followed-up according to the trial schedule (follow-up data can be collected and used in the trial analysis)

- Patient or their parent(s)/legal guardian does not wish for the patient to attend trial follow-up visits but is willing for the patient to be followed-up at standard clinic visits (follow-up data can be collected at standard clinic visits and used in the trial analysis)
- Patient or their parent(s)/legal guardian is not willing for the patient to be followed up for trial purposes at any further visits (any data collected prior to the withdrawal of consent can be used in the trial analysis).

The following should be clearly documented in the medical notes:

- The date the patient or their parent(s)/legal guardian withdraw consent.
- The reason, if given (e.g. toxicity to drug).
- Type of withdrawal

### 8.3 Termination of the Study by ITCC

The ITCC Consortium may terminate this study prematurely, either in its entirety or at an investigative site, for reasonable cause provided that written notice is submitted in advance of the intended termination. Advance notice is not required if the study is stopped due to significant safety concerns.

## 9.0 STATISTICAL CONSIDERATIONS

This is a Phase I/II dose escalation study for patients enrolled in stratum 1 with CD22-positive BCP-ALL to establish the maximum tolerated dose and preliminary activity of InO as monotherapy or as add-on to modified UKALL-R3 reinduction chemotherapy regimen.

The dose escalation phase (Stratum 1A and 1B) will follow a Rolling-6 dose escalation design[100].

In Stratum 1B, initially InO will be combined with vincristine and dexamethasone only. Once the RP2D of InO, dexamethasone and vincristine is established, asparaginase will be added to the next cohort (referred to as Stratum 1B-ASP). Stratum 1B-ASP cohort will initially enroll 6 patients, in absence of major safety concerns. Assuming preliminary safety is confirmed, at least 4 additional patients will be enrolled to this cohort (minimum of 10 patients at the RP2D for the combination) to better characterize the safety profile of this modified UKALL-R3 regimen before proceeding with the planned randomized study (separate protocol).

The single-agent phase II cohort will follow a single stage design.

Note: Patients participating in Stratum 2 (other CD22-positive hematological malignancies) will not formally participate in the dose finding or the Phase 2 part of the study, as this is an explorative cohort only. These patients are rare and heterogeneous and numbers preclude designing a formal dose-finding study for this stratum.

### 9.1 Stratum 1A

9.1.1. Primary endpoints for patients enrolled in Stratum 1A

Dose-limiting toxicities (DLTs) during the first cycle of therapy.

9.1.2. Secondary endpoints for patients enrolled in Stratum 1A

1. Safety and tolerability:
    - AEs, as characterized by type, frequency, severity (as graded using CTCAE version, v4.03, timing, seriousness, and relation to study therapy, during the first and subsequent cycles of therapy.
    - Occurrence of toxic death; i.e., death attributable to InO therapy.
    - Occurrence of hepatic veno-occlusive disease (VOD)/sinusoidal obstruction syndrome (SOS) during or after therapy with InO.
    - Laboratory abnormalities as characterized by type, frequency, severity and timing.
    - The cumulative incidence of non-relapse mortality, defined as the cumulative probability of non-relapse mortality, with time calculated between start of study treatment and death due to other causes than relapsed or refractory leukemia or lymphoma, accounting for competing events.

2. Measures of anti-leukemic activity:
    - ORR, defined as CR, CRi, or CRp both after cycle 1 as well as the best response over multiple cycles of InO therapy (see Appendix 2 for definitions).
    - MRD levels, including the percentage of patients who become MRD-negative (complete MRD response defined as an MRD-level < $1x10^{-4}$), after cycle 1, as well as the overall best response (MRD-negativity) over multiple cycles.
    - Duration of response, defined as the time between achieving response (CR, CRi or CRp) after starting study treatment and documented relapse or death.
    - Number and percentage of patients being transplanted and those who received CAR-T cells therapy after treatment with InO.

- EFS, defined as the time between start of study treatment and first event including failure to achieve CR/CRp/CRi (calculated as an event on day 0), relapse, death of any cause and second malignancies.
- Overall survival, defined as time to death following start of study treatment.
- The cumulative incidence of non-response or relapse, defined as the cumulative probability of non-response or relapse, with time calculated between start of study treatment and relapse and with non-responders included as an event on day 0. Non-relapse death is considered a competing event.

3. Serum pharmacokinetic parameters of InO and unconjugated calicheamicin.

4. Pharmacodynamic parameters
   - Relationship between response (ORR) and CD22 expression levels and WBC.
   - Relationship between response (ORR) and CD22 saturation kinetics.
   - Relationship between response (ORR) and calicheamicin sensitivity.
   - Clonal evolution (CD22-negativity) and relation to loss of response.

5. Other endpoints
   - The percentage of patients responding to InO (ORR) without adequate recovery of CD19-positive B-cells (below lower limit of normal (LLN) for age) or immunoglobulins (below LLN for age). following 4 weeks, 10 weeks, 3, 6 and 12 months after treatment with InO, excluding patients who have been transplanted from the date of HSCT or have received CAR-T cells therapy.
   - Percentage of patients who exhibit anti-drug antibodies (ADA).

## 9.2 Phase 2 cohort

### 9.2.1 Primary endpoints for patients enrolled in Phase 2 cohort

ORR, defined as the percentage of patients with CR, CRi, CRp, measured as best response during InO treatment (see Appendix 2 for definitions).

### 9.2.2. Secondary endpoints for patients enrolled in Phase 2 cohort

1. Safety:
   - AEs, as characterized by type, frequency, severity (as graded using CTCAE v4.03, timing, seriousness, and relation to study therapy, during the first and subsequent cycles of therapy.
   - Occurrence of toxic death; i.e., death attributable to InO therapy.
   - Occurrence of VOD/SOS during or after therapy with InO.
   - Laboratory abnormalities as characterized by type, frequency, severity and timing.
   - The cumulative incidence of non-relapse mortality, defined as the cumulative probability of non-relapse mortality, with time calculated between start of study treatment and death due to other causes than relapsed or refractory leukemia or lymphoma, accounting for competing events.

2. Other measures of anti-leukemic activity:
   - ORR after cycle 1.

- Minimal residual disease levels, including the percentage of patients who become MRD-negative (complete MRD response defined as an MRD-level < $1x10^{-4}$), after cycle 1, as well as the best response (MRD-negativity) over multiple cycles.
- Duration of response, defined as the time between achieving response (CR, CRi or CRp) after starting study treatment and documented relapse or death.
- Number and percentage of patients being transplanted and those receiving CAR T-cell therapy after treatment with InO.
- EFS, defined as the time between start of study treatment and first event including failure to achieve CR/CRp/CRi (calculated as an event on day 0), relapse, death of any cause and second malignancies.
- Survival, defined as time to death following start of study treatment.
- The cumulative incidence of non-response or relapse, defined as the cumulative probability of non-response or relapse, with time calculated between start of study treatment and relapse and with non-responders included as an event on day 0. Non-relapse death is considered a competing event.

3. Serum pharmacokinetic parameters of InO and unconjugated calicheamicin.

4. Pharmacodynamic parameters
    - Relationship between response (ORR) and CD22 expression levels and WBC.
    - Relationship between response (ORR) and CD22 saturation kinetics.
    - Relationship between response (ORR) and calicheamicin sensitivity.
    - Clonal evolution (CD22-negativity) and relation to loss of response.

5. Other endpoints
    - The percentage of patients responding to InO (ORR) without adequate recovery of CD19-positive B-cells (below LLN for age) or immunoglobulins (below LLN for age) following 4 weeks, 10 weeks, 3, 6 and 12 months after treatment with InO, excluding patients who have been transplanted from the date of HSCT or have received CAR-T cells therapy.
        - Percentage of patients who exhibit ADA.

## 9.3 Stratum 1B and 1B-ASP

### 9.3.1 Primary endpoint

Dose-limiting toxicities (DLTs) during the first cycle of InO when added to a modified UKALL-R3 re-induction chemotherapy regimen without or with ASP.

### 9.3.2 Secondary endpoints

1. Safety:
    - AEs
    - Occurrence of toxic death; i.e., death attributable to InO therapy.
    - Occurrence of hepatic VOD/SOS during or after therapy with InO.
    - Laboratory abnormalities

- Cumulative incidence of non-relapse mortality

2. Other measures of anti-leukemic activity:
   - ORR
   - MRD levels
   - Duration of response
   - Number and percentage of patients who undergo HSCT and those receiving CAR T-cell therapy after treatment with InO.
   - EFS
   - Overall survival
   - Cumulative incidence of non-response or relapse

3. Serum pharmacokinetic parameters of InO and unconjugated calicheamicin during treatment combined with modified UKALL-R3 re-induction regimen both with and without pegylated asparaginase.

4. Pharmacodynamic parameters
   - Relationship between response (ORR) and CD22 expression levels
   - Clonal evolution (CD22-negativity) and relation to loss of response .

## 9.4 Stratum 2

9.4.1 Primary endpoint

Safety and tolerability:

- AEs, as characterized by type, frequency, severity (as graded using CTCAE v4.03, timing, seriousness, and relation to study therapy, during the first and subsequent cycles of therapy.
- Occurrence of toxic death; i.e., death attributable to InO therapy.
- Occurrence of hepatic VOD/SOS during or after therapy with InO.
- Laboratory abnormalities as characterized by type, frequency, severity and timing.
- The cumulative incidence of non-relapse mortality, defined as the cumulative probability of non-relapse mortality, with time calculated between start of study treatment and death due to other causes than relapsed or refractory leukemia or lymphoma, accounting for competing events.

9.4.2 Secondary endpoints for patients enrolled in Stratum 2

1. Measures of anti-tumor activity:
   - Overall remission rate (CR and PR) both after cycle 1 as well as overall best response in patients receiving multiple cycles of InO therapy (see Appendix 2 for definitions).
   - Duration of response, defined as the time between achieving response (CR and PR) after starting study treatment and documented relapse or death.
   - Number and percentage of patients being transplanted and those receiving CAR T-cell therapy after treatment with InO.

- EFS, defined as the time between start of study treatment and first event including failure to achieve CR/PR (calculated as an event on day 0), relapse, death of any cause and second malignancies.
- Overall survival, defined as time to death following start of study treatment.
- The cumulative incidence of non-response or relapse, defined as the cumulative probability of non-response or relapse, with time calculated between start of study treatment and relapse and with non-responders included as an event on day 0. Non-relapse death and is considered a competing event.

2. Serum pharmacokinetic parameters of InO and unconjugated calicheamicin.

3. Other endpoints:

- The percentage of patients responding to InO (ORR) without adequate recovery of CD19-positive B-cells (below LLN for age) or immunoglobulins (below LLN for age) following 4 weeks, 10 weeks, 3, 6 and 12 months after treatment with InO, excluding patients who have been transplanted from the date of HSCT or have received CAR-T cells therapy.
- Percentage of patients who exhibit ADA. (NOT valid for patients enrolled after Amendment 4)

## 9.5 Stratum 3

9.5.1 Primary endpoints for patients enrolled in the VHR cohort

ORR, defined as the percentage of patients with CR, CRi, CRp, measured as best response to InO treatment (see Appendix 2 for definitions) as a single agent in CD22-positive VHR 1st relapse BCP ALL patients.

9.5.2 Secondary endpoints for patients enrolled in the VHR cohort

1. Safety:
    - AEs, as characterized by type, frequency, severity (as graded using CTCAE v4.03), timing, seriousness, and relation to study therapy, during the first and subsequent cycles of therapy.
    - Occurrence of toxic death; i.e., death attributable to InO therapy.
    - Occurrence of VOD/SOS during or after therapy with InO.
    - Laboratory abnormalities as characterized by type, frequency, severity and timing.
    - The cumulative incidence of non-relapse mortality, defined as the cumulative probability of non-relapse mortality, with time calculated between start of study treatment and death due to other causes than relapsed or refractory leukemia or lymphoma, accounting for competing events.

2. Other measures of anti-leukemic activity:
    - ORR after cycle 1.
    - Minimal residual disease levels, including the percentage of patients who become MRD-negative (complete MRD response defined as an MRD-level < $1x10^{-4}$), after cycle 1, as well as the best response (MRD-negativity) over multiple cycles.
    - Duration of response, defined as the time between achieving response (CR, CRi or CRp) after starting study treatment and documented relapse or death.
    - Number and percentage of patients being transplanted and those receiving CAR-T-cells therapy after treatment with InO.

- EFS, defined as the time between start of study treatment and first event including failure to achieve CR/CRp/CRi (calculated as an event on day 0), relapse, death of any cause and second malignancies.
- Survival, defined as time to death following start of study treatment.
- The cumulative incidence of non-response or relapse, defined as the cumulative probability of non-response or relapse, with time calculated between start of study treatment and relapse and with non-responders included as an event on day 0. Non-relapse death is considered a competing event.
- To study the interval between InO re-induction and CAR-T cells therapy based on MRD negativity and B cell aplasia after InO re-induction.

3. Pharmacodynamics parameters

- Relationship between response (ORR) and CD22 expression levels and WBC.
- Relationship between response (ORR) and calicheamicin sensitivity.
- Clonal evolution (CD22-negativity) and relation to loss of response.

4. Other endpoints

- The percentage of patients responding to InO (ORR) without adequate recovery of CD19-positive B-cells and CD4+/CD8+ T-cells(below LLN for age) or immunoglobulins (below LLN for age) following 4,6,8 and 10 weeks, 3, 6 and 12 months after treatment with InO, excluding patients who have been transplanted from the date of HSCT or CAR T-cell infusion.

## 9.6 Statistical design for dose escalation phase (Stratum 1A)

9.6.1 Rolling-6 design

A Rolling-6 escalation design will be used [97,110]. Escalation/de-escalation decisions will be based on the DLTs that occur during the first cycle of treatment for patients in Stratum 1A. The starting dose level is dose level 1, as described in ***Table 15***. Dose level -2 will be the minimum dose level considered during the InO monotherapy escalation phase.

The MTD is the highest dose level tested at which 0/6 or 1/6 patients experiences DLT during cycle 1 with at least 2 patients experiencing DLT at the next higher dose. If the highest specified dose level (dose Level 3) in this study is reached with 0/6 or 1/6 patients experiencing DLT during cycle 1 – i.e., the MTD has not been reached – this dose level will be referred to as the highest tested dose (HTD), and this dose will be taken forward as the RP2D, unless modelling for cumulative toxicity (see below) suggests otherwise.

Enrollment restrictions during the dose escalation phase (Stratum 1A): enrollment during the dose escalation phase will be restricted as follows:

a) The first 3 patients enrolled at the starting dose level will be age 6 years or older.
b) At least 2 of the next 3 patients at the starting dose levels will be < 6 years of age.
c) The first 6 patients enrolled during the escalation phase must have M3 marrow.

If de-escalation occurs from the starting dose level, restrictions a and b above will remain in place for each subsequent dose level until an MTD is established or until the study is halted

If the dose of InO is escalated above the starting dose level (i.e., 5 evaluable patients are treated with 0 DLT or 6 evaluable patients are treated with at most 1 DLT), restriction a and b will be relaxed for the remainder of the study.

Note that although the Rolling-6 design allows escalation after 3 to 5 evaluable patients are treated with no DLTs, up to six patients will be enrolled if necessary to ensure that if restriction b above has been satisfied.

Based upon the initial experience with dose-escalation from DL1 to DL2 the protocol was amended to allow enrollment of additional patients in DL1 and DL2, based on the following rationale:

- Given the observed DLTs at DL2, of which at least the one with transaminase elevation was considered atypical and the patient tolerated the dose clinically very well, the dose was temporarily de-escalated to DL1 while awaiting approval of this amendment. Six additional DLT-evaluable patients will be recruited in DL1 to better characterize the safety of InO in the pediatric population. This dose level will again be considered safe if a maximum of 1 out of these additional 6 evaluable patients experiences a DLT, (i.e., ≤2 DLTs out of the total of 12 DLT-evaluable patients at this dose level, thus less than 33%).
- When DL1 is once more considered safe, the dose will be re-escalated to DL2, which is the approved adult dose, and again 6 DLT-evaulable patients will be recruited to confirm safety in this dose-level. We will consider this a new cohort of 3-6 patients, and apply the Rolling-6 design characteristics as described above. Prior to re-escalation to DL2 IRB aproval for this amendment needs to be obtained.

Given the Rolling-6 design only considers toxicity in cycle 1, in order to determine the optimal dose for further development, a longitudinal dose-time-toxicity model proposed in Paoletti et al (2015) will be applied at the end of dose escalation phase in Stratum 1A to help evaluate the impact of cumulative toxicities across all cycles and more accurately estimate the toxicity probability. The model will also be re-run at the end of dose expansion cohort to further refine the dose.

9.6.2 Patient evaluability during dose escalation phase (Stratum 1A)

A patient is considered ***evaluable*** for the dose escalation phase of the study if any of the following apply:

- The patient receives at least one dose of InO and experiences a DLT at any time during the first cycle of protocol therapy.
- The patient does not experience DLT during the first cycle of therapy, and receives at least 2 out of 3 doses of the prescribed dose (67%) of InO during that cycle.

A patient will be considered ***not evaluable*** for the dose escalation phase of the study if any of the following apply:

- The patient receives ≤1 out of 3 of the prescribed doses of InO during the first cycle for reasons not related to toxicity or intolerability (e.g. early progressive disease/logistical reasons/non-compliance, etc), or for reasons possibly related to toxicity or intolerability not fulfilling the definition of a DLT as defined in Section 4.6. (e.g. considered related to intrathecal therapy).

Note that patients who are not evaluable will be replaced.

## 9.7 Statistical design for dose escalation phase (Stratum 1B/1B-ASP)

9.7.1 Rolling-6 design

A Rolling-6 escalation design will be used [97,110]. Escalation/de-escalation decisions will be based on the DLTs that occur during the first cycle of treatment for patients in Stratum 1B. The starting dose level is 2 dose levels below the RP2D of InO as single agent, hence 60% of the InO dose. Subsequent dose levels are 80% and 100% of the InO dose. See schedules in ***Table 15***. The dose-escalation will thus not exceed the single-agent MTD of InO.

The MTD of InO combined with reinduction chemotherapy is the highest dose level tested at which 0/6 or 1/6 patients experiences DLT during cycle 1 with at least 2 patients experiencing DLT at the next higher dose. If

the highest specified dose level in this study is reached with 0/6 or 1/6 patients experiencing DLT during cycle 1 – i.e. the MTD has not been reached – this dose level will be referred to as the highest tested dose (HTD).

Initially InO will be combined with vincristine and dexamethasone only.

Once the RP2D of InO in combination with dexamethasone and vincristine is established, asparaginase will be added (referred to as Stratum 1B-ASP). This latter cohort will initially enroll 6 patients, in absence of major safety concerns. Once this dose is cleared, at least 4 additional patients will be enrolled (minimum of 10 patients) to this modified UKALL-R3 cohort to better characterize safety.

Enrollment restrictions during the dose escalation phase (Stratum 1B) include the following:

a) The first 3 patients enrolled at the starting dose level will be age 6 years or older.
b) At least 2 of the next 3 patients at the starting dose levels will be aged 1 year up to 6 years of age.

If de-escalation occurs from the starting dose level, restrictions a and b above will remain in place for each subsequent dose level until an MTD is established or until the study is halted.

If the dose of InO is escalated above the starting dose level, restriction a and b will be relaxed for the remainder of the study.

Note that although the Rolling-6 design allows escalation after 3 to 5 evaluable patients are treated with no DLTs, up to six patients will be enrolled if necessary to ensure that restriction b above has been satisfied.

9.7.2 Patient evaluability during dose escalation phase (Stratum 1B)

A patient is considered ***evaluable*** for the dose escalation phase of the study if any of the following apply:

- The patient receives at least one dose of the planned dose of InO (together with the first dose of dexamethasone) and experiences a DLT at any time during the first cycle of combined study therapy.
- The patient does not experience DLT during the study therapy, and receives at least 2 out of 3 doses of the planned dose of InO during the first cycle and at least 3 days of dexamethasone, 1 dose of vincristine and 1 dose of intrathecal treatment.

A patient will be considered ***not evaluable*** for the dose escalation phase of the study if any of the following apply:

- The patient receives ≤1 dose of the prescribed dose of InO, < 3 days of dexamethasone, or no dose of vincristine or intrathecal treatment during the first cycle for reasons not related to toxicity or intolerability (e.g. early progressive disease/logistical reasons/non-compliance, etc), or for reasons possibly related to toxicity or intolerability not fulfilling the definition of a DLT as defined in section 4.6. (e.g. considered related to intrathecal therapy or specific ALL chemotherapy toxicities precluding ongoing treatment).
- Silent inactivation of asparaginase in a patient enrolled in Stratum 1B-ASP, in which case asparaginase is considered ineffective.

Note that patients who are not evaluable will be replaced.

Even though no dose-escalation takes place for the Stratum 1B-ASP (sub)cohort, evaluability criteria for this cohort will be the same as for the Stratum 1B.

## 9.8 Statistical design for the Phase 2 cohort

The statistical design for the Phase 2 cohort will consist of a single-stage design asumming binomial distribution using exact method. Assuming that an ORR of 30% or less ($H_0$) is not promising and an ORR of over 55% (Ha) is expected. A total of 25 patients evaluable for response will provide 80% power to reject

$H_0$ at a significance level of 0.05 (1-sided) when the true ORR is ≥55% (Ha). The drug will be considered promising if there are ≥12 responders out of 25 response evaluable patients.

9.8.1 Patient evaluability for Phase 2 Cohort

- *The Intent-to-treat Population* (ITT) includes all subjects who are enrolled and treated in the study. The ITT population (patients who received at least 1 dose of study therapy) will be used for primary analysis.

- *The Response Evaluable Population* includes all subjects who received at least one dose of InO and have completed a baseline disease assessment and at least one post-baseline disease assessment. The Response Evaluable Population will be used to evaluate the endpoints for the efficacy profile.

- The *Safety Population* includes all subjects who are enrolled and received at least 1 dose of InO. This patient population will exclude only patients who never receive any InO.

### 9.9 Statistical design for Stratum 2

Due to the exploratory nature of this cohort, only descriptive statistical analysis will be provided for both efficacy and safety endpoints.

### 9.10 Statistical design for Stratum 3

The Stratum 3 VHR cohort is designed as a Optimal Simon 2-stage design based on the exact binomial distribution. Assume that an ORR of 55% or less is not promising (based on historical data from IntReALL), ie. the null hypothesis $H_0$ is ORR $\leq$ 55%, and an ORR of ≥75% is expected with InO treatment, a total of 43 patients will provide 80% power to reject $H_0$ at a significance level of 0.05 (1-sided) when the true ORR is ≥75%.

In the first stage, 15 patients will be enrolled: if 10 patients or more have clinical response (CR, CRp or CRi), the results will appear as promising and the enrollment will continue. A total of 43 patients will be enrolled and the results will be considered promising if at least 29 patients have shown a CR, CRp or CRi.

9.10.1 Patient evaluability for Stratum 3

- The full analysis set includes all subjects who are enrolled and treated in the study. The full analysis set (patients who received at least 1 dose of study therapy) will be used for efficacy and safety analysis.

### 9.11 Patient Accrual and Study Duration

The proposed single agent, dose escalation study Stratum 1A will accrue a minimum of 6 evaluable patients and a maximum of approximately 36 evaluable patients.

The phase 2 cohort requires 25 response-evaluable patients.

The maximum number of patients in Stratum 1B/1B-ASP with the addition of the dose-escalation cohort treated with the lower dose of dexamethasone is estimated to be 42 (34 in the dose finding part, with 4 additional patients in the expansion cohort at the RP2D of 1B-ASP and up to 4 additional patients who are not eligible to receive ASP enrolled in Stratum 1B after initiation of 1B-ASP).. At the time of protocol amendment v4.0 writing, 4 patients were treated at DL-1 (2 DLTs occurred) and recruitment in DL-2 is ongoing with the expectation of recruiting 6 patients. We assume that 2-3 dose levels will be evaluated in Stratum 1B after protocol amendment v4.0 with lower dexamethasone dose. When the recommended dose of InO in combination with vincristine and dexamethasone 10 $mg/m^2$ is reached, 6 patients will be treated

in the 1B-ASP cohort. Once the RP2D is determined for Stratum 1B-ASP, 4 additional patients will be enrolled as an expansion cohort (10 patients to be treated in total in Stratum 1B-ASP), if the treatment is considered safe after the first 6 patients are treated. In addition, up to 4 patients who are not eligible to receive ASP may be enrolled in Stratum 1B after initiation of Stratum 1B-ASP. Non-evaluable patients will be replaced. Therefore, the maximum number of evaluable patients is expected to be 42 (34 in the dose finding part, with 4 additional patients in the expansion cohort at the RP2D of 1B-ASP and up to 4 additional patients who are not eligible to receive ASP enrolled in Stratum 1B after initiation of 1B-ASP).

Therefore the total enrollment of BCP-ALL patients in this study will comprise approximately 103 patients for Stratum 1A, Phase 2 and Stratum 1B/1B-ASP, although less patients may be needed, depending on the number of dose levels tested in stratum 1A and 1B.

Stratum 2 may remain open for the duration of the study, and it is expected that approximately 5-10 patients may be enrolled.

The Stratum 3 (VHR) requires approximately 43 patients. It is expected to last approximately 3 years to recruit approximately 43 evaluable patients.

Across the entire study, the total number of patients to be enrolled is a maximum of approximately 156 patients.

The last patient EOT visit for Stratum 1A is expected in June 2019, for Phase 2 in April 2020, for Stratum 1B/1B-ASP in July 2022 and for Stratum 3 in February 2024. Hence, the last patient last visit (LPLV), including 3 year follow-up is projected for June 2026. The estimated accrual is 2-3 patients per month, including all stratum 1 dose-finding cohorts (1A, 1B and 1B-ASP), and the Phase 2 single agent expansion cohort.

### 9.12 Interim Monitoring of Toxic Death

The occurrence of toxic death (TD) at any time will be a trigger for safety evaluation. When a patient expires while undergoing treatment, within 30 days of receiving protocol therapy, or during follow-up where the death is directly attributed to toxicity experienced on the protocol, the trial steering committee including the statistician will be notified to review the event. No formal statistical rule will be employed. Rather, if the crude proportion of patients experiencing a toxic death exceeds 5% at any time, the cause and circumstances of these deaths will be reviewed with the trial steering committee to determine whether modifications to or termination of the study is warranted.

### 9.13 Analysis of primary endpoints

Statistical summaries of primary endpoints will be reported for the entire study as appropriate by stratum and phase. Note that for Stratum 1B and Stratum 1B-ASP, the primary endpoint will be analyzed separately. These will include:

- For Stratum 1A: DLTs during the first cycle of therapy.
- For Stratum 1B: DLTs during the first cycle of therapy with InO as add-on to a modified UKALL-R3 re-induction regimen.
- For Stratum 1B-ASP: DLTs during the first cycle of therapy with InO as add-on to a modified UKALL-R3 re-induction regimen with asparaginase added.
- For Phase 2 Cohort: Number and percentage of patients who responded to InO (ORR defined as CR, CRi, CRp), with exact 2-sided 95% confidence intervals.
- For Stratum 2: Descriptive summary of safety and tolerability
- For Stratum 3: Number and percentage of patients who responded to InO (ORR defined as CR, CRi, CRp), with exact 2-sided 95% confidence intervals

### 9.14 Analysis of secondary toxicity, efficacy and correlative endpoints.

Statistical summaries of secondary endpoints will be reported for the entire study as appropriate by stratum and phase. In addition strata 1B and 1B-ASP will be analyzed both separately and pooled. These will include:

- The per-cycle and per patient incidence and the cumulative incidence of grade ≥3 hematologic and non-hematologic toxicities
- The cumulative incidence of relapse considering non-relapse mortality as a competing event
- The number and percentage of patients with specific MRD-levels ($<10^{-2}$, $10^{-3}$, $10^{-4}$ and $10^{-5}$) and of negative MRD after cycle 1 as well as best response during subsequent therapy
- The number and percentage of patients who proceed to HSCT or CAR-T cells therapy as consolidation after treatment with InO
- Product-limit estimates of the time to progression following remission (i.e., duration of response), EFS and survival. For time to event variables, patients will be followed until disease progression (relapse), death, loss of follow up, withdrawal of consent, a minimum of one year, even after subsequent treatment, or all patients have been followed for a maximum of 3 years after the protocol completes accrual.
- The analysis of PK parameters including InO and unconjugated calicheamicin using a nonlinear, mixed-effects population modeling approach to estimate clearance and volume of distribution.
- The number and percentage of patients who responded to InO (defined as CR, CRi, CRp) who lack recovery from peripheral blood $CD19^+$ B-cells or immunoglobulins (IgG and IgM) after treatment with InO, censored at time of HSCT or CAR-T cells therapy, at 4 weeks, 10 weeks, 3, 6 and 9 months post last-InO dose
- Percentage of patients who develop anti-drug antibodies (ADA) (NOT valid for patients enrolled after Amendment 4)
- To (preliminarily) assess the relationship between CD22 receptor density, white blood cell count (WBC) at start of treatment, CD22 saturation kinetics, cytogenetics, and in-vitro calicheamicin resistance to clinical response to InO

### 9.15 Definitions

- Response criteria for ALL and NHL are defined in Section 11 and Appendix 2.1 and 2.2. EFS is defined as the time between start of study treatment and first event including failure to achieve CR/CRp/CRi (calculated as an event on day 0), relapse, death of any cause and second malignancies.
- Overall survival (OS) is defined as the time between start of study treatment and death.
- The cumulative incidence of non-response or relapse (CIR) is defined as the cumulative probability of non-response or relapse, with time calculated between start of study treatment and relapse and with non-responders included as an event on day 0. Non-relapse death is considered a competing event.
- The cumulative incidence of non-relapse mortality is defined as the cumulative probability of non-relapse mortality, with time calculated between start of study treatment and death due to other causes than relapsed or refractory leukemia or lymphoma. Relapsed or refractory leukemia or lymphoma is considered a competing event.
- Duration of response is defined as the time between achieving response (CR, CRi or CRp) after starting study treatment and documented relapse or death.

## 10.0 ASSESSMENTS

All study procedures are detailed in the Schedule of Activities (**Table 22,Table 23, Table 24, Table 25, Table 26, Table 33 and Table 34 in Section 7**), which may differ for patients with BCP-ALL and other CD22 positive hematological malignancies. The trial-related blood volume as specified in the protocol will not exceed 3% of the patient's blood volume during a 4-week period, especially during the first induction cycle when sampling is most intensive.

Every effort should be made to ensure that the protocol required tests and procedures are completed as described. However, it is anticipated that from time to time there may be circumstances, outside of the control of the Investigator, that may make it unfeasible to perform the test. In these cases, the Investigator will take all steps necessary to ensure the safety and well-being of the patient. When a protocol required test cannot be performed, the Investigator will document the reason for this and any corrective and preventive actions which he/she has taken to ensure that normal processes are adhered to as soon as possible. The sponsor will need to be informed of these incidents in a timely fashion.

### 10.1 Safety Assessments

Safety assessments will include collection of AEs, SAEs, vital signs and physical examination, echocardiogram, laboratory assessments including pregnancy tests, and verification of concomitant treatments as described in the sections below.

### 10.2 Pregnancy Testing and Contraception Check

For all female patients with childbearing potential, a serum or urine pregnancy test, with sensitivity of at least 25 mIU/ml, will be performed prior to starting study therapy. Following a negative pregnancy result at screening, appropriate contraception for patients of childbearing potential must be commenced. Pregnancy tests will also be routinely repeated as per the schedule of events. A pregnancy test will also be performed additionally whenever one menstrual cycle is missed (for females with childbearing potential) or when potential pregnancy is otherwise suspected. In the case of a positive confirmed hCG test/pregnancy, the patient will be withdrawn from study medication but may remain in the study. In the case of a positive pregnancy test, investigators should follow local regulations/IRB/EC requirements regarding whether or the patient's parents/legal guardians are to be informed.

### 10.3 Adverse Events

Adverse events should be documented and recorded at each visit using the National Cancer Institute Common Terminology Criteria for Adverse Events (NCI CTCAE) version 4.03.
AEs and SAEs should be recorded on the eCRF from the time the patient provided informed consent.

Patients must be followed for AEs and SAEs for 10 weeks after the last InO dose or until all drug related toxicities have resolved, whichever is later. If a patient begins a new anticancer therapy, the reporting period for non-serious AEs and SAEs ends at the time the new treatment is started unless an SAE is considered at least possibly related to InO by the Investigator. All cases of VOD should be reported as an SAE regardless of causality and severity for 1 year after enrolment, even if the event occurs after follow-up therapy.

After the active reporting period has ended new events need to be reported to the Sponsor only if the event is classified as 'serious' and if there is at least a reasonable possibility of the event being related to InO (i.e. VOD post-HSCT). Details about AE and SAE reporting are provided in Section 12.0.

### 10.4 Laboratory Safety Assessments

Hematology and blood chemistry will be drawn at the time points described in the Schedule of Activities (Section 7) and analyzed at local laboratories.

Blood tests include:

- Hematology: Hemoglobin, platelet count, WBC count and differential (percentage neutrophils, lymphocytes, eosinophils, basophils, monocytes, blasts, myelocytes, promyelocytes, metamyelocytes), including absolute neutrophil count (ANC).

- Blood chemistry: sodium, potassium, calcium, phosphate, uric acid, creatinine, urea or blood urea nitrogen (BUN), total protein, albumin, Total and direct bilirubin, AST, ALT, alkaline phosphatase, GGT, glucose, lipase and amylase.
- Coagulation: aPTT, PT, fibrinogen.

Investigators may order additional blood/urine tests for planning treatment administration, dose modification, or further evaluation of adverse events.

### 10.5 Vital signs and Physical Examination

Patients will have a physical exam to include height, weight, vital signs, assessment of Lansky or Karnofsky performance status (Appendix 1), at the time points described in the Schedule of Activities (Section 7). All treatment-emergent abnormalities will be recorded on the Adverse Events eCRF page.

Blood pressure and pulse rate should be recorded in either the sitting or supine position (after patient has been in the supine position for at least two minutes).

In children 3 years of age or greater, height should be measured using a wall-mounted stadiometer by appropriately trained personnel. Length should be measured in children <3 years of age. If possible, the same person should perform height measurement at each visit.

### 10.6 Echocardiograms

Echocardiograms will be performed at the time points described in the Schedule of Activities (Section 7) and as clinically indicated. Left ventricular function (shortening fraction and/or ejection fraction) as well as evaluation for pericardial effusion should be performed.

### 10.7 Menarchal Status Assessments

Assessments of menarchal status in female patients will be performed at the time points described in the Schedule of Events. Age at menarche will be recorded in the eCRF.

### 10.8 Disease Response Assessments

Disease response assessments will include all known or suspected disease sites and will include evaluation of the peripheral blood, bone marrow, physical exam (liver and spleen size), and/or (when applicable) CSF.

Anti-cancer activity will be assessed at baseline, during treatment as specified in the Schedule of Events/Activities, whenever disease progression is suspected (e.g., symptomatic deterioration), and at the time of withdrawal from treatment.

Disease response assessments will be based upon disease specific response criteria (Appendix 2), and will in part be analyzed at a central laboratory (Minimal Residual Disease and potential clonal evolution). All patients' files and radiologic images and pathology samples must be available for source verification and for potential peer review.

- **Hematologic Response Assessments**

  Complete blood counts and differentials, bone marrow differential, and extra-medullary disease assessment (including the liver and spleen) from the physical examination and CSF-evaluation will be used to determine response to treatment, as outlined in Appendix 2.

- **Molecular Response Assessments**

  Samples (bone marrow and/or peripheral blood samples) will be collected at each efficacy time point for independent central laboratory mRNA quantitative RQ-PCR analysis of Ig-rearrangements. In addition, we will perform multi-color flow cytometry based MRD as this may show patterns of clonal evolution such as CD22-negative leukemia. See Schedule of Events (Section 7), and Appendix 2 for response definitions. Central molecular assessments and flow MRD will be carried out at central laboratory at Erasmus MC.

# 11.0 RESPONSE CRITERIA

## 11.1 Response criteria in BCP-ALL patients

Response criteria for patients treated for BCP-ALL are detailed in Appendix 2.1, and follows standard procedures in leukemia, as designed by the National Comprehensive Cancer Network with slight modifications.

## 11.2 Response criteria in patients with other CD22-positive malignancies

Response and progression will be evaluated in this study using the recently published International pediatric NHL Response Criteria summarized in Appendix 2.2.

Key points are that a maximum of 6 target lesions are identified, and that changes in the sum of the products of the largest diameter and the perpendicular diameter (SPD) for each tumor mass, as measured by CT or MRI, are used as the measure of tumor size change.

# 12.0 ADVERSE EVENTS

## 12.1 Definition of an Adverse Event

An adverse event (AE) is any untoward medical occurrence in a patient or clinical investigation subject administered a pharmaceutical product and which does not necessarily have to have a causal relationship with the treatment.

An AE can therefore be any unfavorable and unintended sign (including an abnormal laboratory finding, for example), symptom, or disease temporally associated with the use of a medicinal product, whether or not considered related to the medicinal product.

## 12.2 Definition of Serious Adverse Events

A Serious Adverse Event (SAE) is any untoward medical occurrence that at any dose:

- Results in death;
- Is life threatening (patient was at risk of death at the time of the event);
- Requires inpatient hospitalization or prolongation of existing hospitalization;
- Results in persistent or significant disability/incapacity (substantial disruption of the ability to conduct normal life functions);
- Is a congenital anomaly/birth defect;
- Any other important medical event requiring medical or surgical intervention to prevent serious outcome):

  An important medical event that may not be immediately life-threatening or result in death or hospitalization, but based on medical judgment may jeopardize the patient and may require medical or surgical intervention to prevent any of the outcomes listed above (*i.e.,* death of patient, life-threatening, hospitalization, prolongation of hospitalization, congenital anomaly, or persistent or significant disability/incapacity). Examples of such events include allergic bronchospasm requiring intensive treatment in an emergency room or at home, blood dyscrasias or convulsions that do not result in inpatient hospitalization, or the development of drug dependency or drug abuse, or Hy's Law cases.

Progression of the malignancy under study (including signs and symptoms of progression) should not be reported as an SAE unless the outcome is fatal and unless it is considered drug-related within the safety reporting period.

Hospitalization due to signs and symptoms of disease progression should not be reported as an SAE. Hospitalisation for social or logistical reasons (such as a blood transfusion or central line placement or a bone marrow puncture) does not need to be reported as an SAE, unless hospitalization is due to unforeseen complications.

If the malignancy has a fatal outcome during the study or within the safety reporting period, then the event leading to death must be recorded as an AE and as an SAE with Common Terminology Criteria (CTC) grade 5.

Medical and scientific judgement is exercised in determining whether an event is an important medical event. An important medical event may not be immediately life threatening and/or result in death or hospitalization. However, if it is determined that the event may jeopardize the patient or may require intervention to prevent one of the other AE outcomes, the important medical event should be reported as serious. Examples of such events are intensive treatment in an emergency room or at home for allergic bronchospasm; blood dyscrasias or convulsions that do not result in hospitalization; or development of drug dependency or drug abuse.

AEs of Special Interest that require immediate reporting, should also be reported as 'other medically important condition'. This includes potential Hy's Law cases, VOD / SOS.

### 12.3 Adverse Event Reporting

All observed AEs regardless of suspected causal relationship to the investigational product(s) will be reported on the electronic CRFs beginning following signing of informed consent until 10 weeks following the last dose of study therapy, unless a new treatment starts earlier.

For all AEs, the Investigator must pursue and obtain information adequate both to determine the outcome of the AE and to assess whether it meets the criteria for classification as an SAE requiring immediate notification to the sponsor. For all AEs, sufficient information should be obtained by the Investigator to determine the causality of the AE. The Investigator is required to assess causality. Follow up by the Investigator may be required until the event or its sequelae resolve or stabilize at a level acceptable to the Investigator, and sponsor concurs with that assessment.

All AEs will be reported on the AE page(s) of the eCRF. It should be noted that the form for collection of SAE information is not the same as the AE eCRF. Where the same data are collected, the forms must be completed in a consistent manner. For example, the same AE term should be used on both forms. AEs should be reported using concise medical terminology on the eCRFs as well as on the form for collection of SAE information.

As part of ongoing safety reviews conducted by the Sponsor, any non-serious adverse event that is determined by the Sponsor to be serious will be reported by the Sponsor as a SAE. To assist in the determination of case seriousness further information may be requested from the Investigator to provide clarity and understanding of the event in the context of the clinical study.

### 12.4 Serious Adverse Event Reporting

For SAEs, the active reporting period to the sponsor or its designated representative begins from the time that the patient provides informed consent, which is obtained prior to the patient’s participation in the study, i.e., prior to undergoing any study related procedure and/or receiving investigational product, through and including the 10-week safety visit after the last administration of the investigational product, unless a new treatment starts earlier.

After the active reporting period has ended, new events need to be reported to the Sponsor as SAE only if the event classifies as ‘serious’ and if there is at least a reasonable possibility of being related to study drug. An exception to this is that all cases of VOD/SOS should be reported as an SAE up to 1 year after enrolment, regardless of causality to InO.

If an SAE occurs, the sponsor Erasmus MC is to be notified within 24 hours of Investigator awareness of the event. In particular, if the SAE is fatal or life threatening, notification to the sponsor must be made immediately, irrespective of the extent of available AE information.

This timeframe also applies to additional new information (follow up) on previously forwarded SAE reports as well as to the initial and follow up reporting of pregnancy and occupational exposure cases.

In the rare event that the Investigator does not become aware of the occurrence of an SAE immediately (e.g., if an outpatient study patient initially seeks treatment elsewhere), the Investigator is to report the event within 24 hours after learning of it and document the time of his/her first awareness of the AE.

For all SAEs, the Investigator is obligated to pursue and provide information to the sponsor in accordance with the timeframes for reporting specified above. In addition, an Investigator may be requested by the sponsor to obtain specific additional follow up information in an expedited fashion. This information collected for SAEs is more detailed than that captured on the AE eCRF. In general, this will include a description of the AE in sufficient detail to allow for a complete medical assessment of the case and independent determination of possible causality. Information on other possible causes of the event, such as concomitant medications, vaccines, and/or illnesses must be provided.

## 12.5 AEs of Special Interest

Adverse Events of Special Interest (AESI) constitute AEs (serious or non-serious) of scientific and medical concern in the evaluation of InO, for which in addition to ongoing monitoring, rapid communication by the Investigator to the Sponsor is considered appropriate. Such events may require further investigation in order to characterize and understand them. AESI may be added or removed during a study by Protocol Amendment.

### 12.5.1 Reporting of AESI **WITH** immediate notification

Investigator should notify the sponsor immediately (i.e. within 24 hours) of the following events, as per SAE notification and / or using the pregnancy form (whichever is applicable):

a. Hy's Law cases

Abnormal values in aspartate transaminase (AST) and/or alanine transaminase (ALT) concurrent with abnormal elevations in total bilirubin that meet the criteria as defined in Section 5.6.2 in the absence of other causes of liver injury are considered potential cases of drug-induced liver injury (potential Hy's Law cases) and should always be considered an important medical event, to be reported as an SAE.

b. VOD/SOS

Suspected cases of hepatic VOD/SOS for up to 1 year from enrollment, irrespective of causality, will be reported in the CRF and need to be reported as an SAE. Serious adverse event reports will include a complete list of anti-leukemic therapy, including pre- and post-study transplant conditioning regimen(s) including GVHD prophylaxis and concomitant medications used for prevention of hepatic toxicity (for example ursodiol, defibrotide, low molecular weight heparin) or treatment of VOD (defibrotide).

c. Exposure during Pregnancy

An exposure during pregnancy occurs if:

- A female becomes, or is found to be, pregnant either while receiving or having been exposed (e.g., because of treatment or environmental exposure) to the investigational product; or the female becomes, or is found to be pregnant after discontinuing and/or being exposed to the investigational product;

If a study patient or study patient's partner becomes or is found to be pregnant during the study patient's treatment with the investigational product, the Investigator must submit this information to the sponsor. In addition, the Investigator must submit information regarding environmental exposure to the IMP in a pregnant woman (e.g., a patient reports that she is pregnant and has been exposed to a cytotoxic product by inhalation or spillage). This must be done irrespective of whether an AE has occurred and within 24 hours of awareness of the exposure. The information submitted should include the anticipated date of delivery (see below for information related to termination of pregnancy). Additionally, investigators should follow local regulations/IRB/EC requirements regarding whether or the patient's parents/legal guardians are to be informed.

Follow up is conducted to obtain general information on the pregnancy and its outcome for all reports with an unknown outcome. The Investigator will follow the pregnancy until completion or until pregnancy termination and notify sponsor. In the case of a live birth, the structural integrity of the neonate can be assessed at the time of birth. In the event of a termination, the reason(s) for the termination should be specified and, if clinically possible, the structural integrity of the terminated fetus should be assessed by gross visual inspection (unless pre procedure test findings are conclusive for a congenital anomaly and the findings are reported).

If the outcome of the pregnancy meets the criteria for an SAE (i.e., ectopic pregnancy, spontaneous abortion, intrauterine fetal demise, neonatal death, or congenital anomaly [in a live born, a terminated fetus, an intrauterine fetal demise or a neonatal death]), the Investigator should follow the procedures for reporting SAE.

Additional information about pregnancy outcomes that are reported as SAEs follows:
- Spontaneous abortion includes miscarriage and missed abortion;
- Neonatal deaths that occur within 1 month of birth should be reported, without regard to causality, as SAEs. In addition, infant deaths after 1 month should be reported as SAEs when the Investigator assesses the infant death as related or possibly related to exposure to investigational product.

Additional information regarding the exposure during pregnancy may be requested by the Investigator.

d. Symptomatic overdose with IMP/NIMP
- An overdose (accidental or intentional) with the IMP/non investigational medicinal product (NIMP) is an event suspected by the Investigator or spontaneously notified by the patient (not based on systematic pills count) and defined as at least twice of the intended dose within the intended therapeutic interval, adjusted according to the tested drug.

e. Second primary malignancy

12.5.2 Reporting of AESI **without** immediate notification
The following events require reporting, but do not required immediate notification:
- Asymptomatic overdose with IMP/NIMP.
- Neurotoxicity, nephrotoxicity, QT abnormalities, interstitial lung disease and pancreatitis

These events will be collected in the eCRF.

### 12.6 Sponsor Reporting Requirements to Regulatory Authorities
Adverse event reporting, including suspected unexpected serious adverse reactions, will be carried out in accordance with applicable local regulations.

### 12.7 Reporting to Pfizer
Reportable SAEs/SUSARs that occur after the 1st InO dose and other safety information will be forwarded by Erasmus MC to the Pfizer Drug Safety Unit within 1 business day of becoming aware of the SAE.

### 12.8 Safety Oversight
Adverse events will be reviewed by the sponsor on an ongoing basis. Regular teleconferences (depending on accrual) will be set up with the Trial Steering Committee to discuss potential DLTs, SAEs and other relevant safety information, including AEs of special interest. During the phase I part of the study regular TCs with the sites will be set up to capture safety information and discuss the enrolled patients at an early stage.

### 12.9 SUSARs
A Suspected Unexpected Serious Adverse Reaction (SUSAR) is defined as a suspected Adverse Reaction that occurs in the trial that is both unexpected and serious.

Adverse reactions (AR) are those AEs of which a reasonable causal relationship to any dose administered of the investigational medicinal product and the event is suspected.
Unexpected adverse reactions are adverse reactions, of which the nature, or severity, is not consistent with the applicable product information (e.g. Investigator's Brochure for an unapproved IMP or SmPC for an authorized medicinal product). For this study the IB of Inotuzumab Ozogamicin is used to assign expectedness. If a previously reported non-serious adverse reaction occurs as a serious reaction, the reaction will be considered to be unexpected and reported as a SUSAR. Each fatal reaction will also be reported as a SUSAR if not reported as fatal in the current InO IB.

Timelines for SUSAR reporting are as follows:

- Initial fatal or life-threatening SUSARs will be reported to the competent authorities as soon as possible but no later than 7 calendar days from initial receipt of SAE by Sponsor. A completed follow-up will be submitted within an additional 8 calendar days;
- All other SUSARs will be reported to the competent authorities as soon as possible but no later than 15 calendar days from initial receipt of SAE by Sponsor;
- SUSAR's will also be reported to the ethics committees in accordance with applicable local regulations.

### 12.10 Development Safety Update Reports

The Sponsor Erasmus MC will prepare a DSUR (Development Safety Update Report) each year; this DSUR will be distributed to the national coordinating centers for submission to the competent authorities and ethics committees.

# 13.0 DATA HANDLING AND RECORD KEEPING

## 13.1 Case Report Forms / Electronic Data Record

As used in this protocol, the term Case Report Form (CRF) should be understood to refer to either a paper form or an electronic data record or both, depending on the data collection method(s) used in this study. An eCRF is required and should be completed for each included patient. The completed original eCRFs are the sole property of Erasmus MC and should not be made available in any form to third parties, except for authorized representatives of Erasmus MC or appropriate regulatory authorities, without written permission from Erasmus MC.

The Investigator has ultimate responsibility for the collection and reporting of all clinical, safety and laboratory data entered on the eCRFs and any other data collection forms (source documents) and ensuring that they are accurate, authentic/original, attributable, complete, consistent, legible, timely (contemporaneous), enduring and available when required. The eCRFs must be signed by the Investigator or by an authorized staff member to attest that the data contained on the eCRFs is true. Any corrections to entries made in the eCRFs or source documents must be dated, initialed and explained (if necessary) and should not obscure the original entry.

In most cases, the source documents are the hospital's, the physician's patient chart, the patient’s diaries, and the completed questionnaires. In these cases data collected on the eCRFs must match the data in those charts and documents.

In some cases, the eCRF, or part of the eCRF, may also serve as source documents. In these cases, a document should be available at the Investigator’s site as well as at Erasmus MC and clearly identify those data that will be recorded in the eCRF, and for which the eCRF will stand as the source document.

## 13.2 Record retention / Archiving

To enable evaluations and/or audits from regulatory authorities or Erasmus MC, the Investigator agrees to keep records, including the identity of all participating patients (sufficient information to link records, e.g., eCRFs and hospital records), all original signed informed consent/assent documents, copies of all CRFs, safety reporting forms, source documents, and detailed records of treatment disposition, and adequate documentation of relevant correspondence (e.g., letters, meeting minutes, telephone calls reports). The records should be retained by the Investigator according to International Conference on Harmonisation (ICH), according to local regulations, or as specified in the Clinical Study Agreement (CSA), whichever is longer.

If the Investigator becomes unable for any reason to continue to retain study records for the required period (e.g., retirement, relocation), Erasmus MC should be prospectively notified. The study records must be transferred to a designee acceptable to Erasmus MC, such as another Investigator, another institution, or to an independent third party arranged by Erasmus MC.

Investigator records must be kept for a minimum of 15 years after completion or discontinuation of the study or for longer if required by applicable local regulations.

The Investigator must obtain Erasmus MC’s written permission before disposing of any records, even if retention requirements have been met.

## 14.0 QUALITY MANAGEMENT

During study conduct and/or after study completion, the study site may be subject to review by the institutional review board (IRB) / ethics committee (EC), and/or to quality assurance audits performed by Erasmus/ITCC, or companies working with or on behalf of Erasmus/ITCC, and/or to inspection by appropriate regulatory authorities.

During study conduct, Erasmus MC or its collaborative partner will conduct periodic monitoring visits to ensure that the protocol and Good Clinical Practices (GCPs) are being followed. The monitors will review source documents to confirm that the data recorded on (e)CRFs is accurate. The investigator and institution will allow Erasmus MC or Pfizer monitors/auditors or its agents and appropriate regulatory authorities direct access to source documents to perform this verification. This verification may also occur after study completion.

It is important that the Investigator(s) and their relevant personnel are available during the monitoring visits and possible audits or inspections and that sufficient time is devoted to the process.

### 14.1 Site Set-up and Initiation

All sites will be required to sign a Clinical Study Site Agreement prior to participation. In addition, all participating Investigators will be asked to sign the necessary confidentiality agreement and supply a current, dated, and signed CV to the ECTC Trial Office, including evidence of GCP-training and expertise in running early clinical trials.

All members of the site research/study team will also be required to sign the ‘Site Signature and Delegation of Authority Log’*,* which should be returned to the ECTC Trial Office. Prior to commencing recruitment all sites will undergo a process of initiation. Key members of the site research team will be required to attend either a meeting and/or a teleconference covering aspects of the trial design, protocol procedures, Adverse Event reporting, collection and reporting of data and record keeping.

Sites will be provided with an Investigator File and a Pharmacy File containing essential documentation, instructions, and other documentation required for the conduct of the trial. The ECTC Trial Office must be informed immediately of any change in the site research team.

### 14.2 On-site Monitoring

Monitoring will be carried out as required following a risk assessment and as documented in the InO Quality Management Plan. Additional on-site monitoring visits may either be triggered for example by poor (E)CRF return, poor data quality, low SAE reporting rates, excessive number of patient withdrawals or deviations or for instance by patient recruitment figures. If a monitoring visit is required the monitor of the CRO will contact the site to arrange a date for the proposed visit and will provide the site with written confirmation. Investigators will allow the InO trial staff and/or CRO access to source documents as requested.

### 14.3 Central Monitoring

Sponsor trial staff and/or the CRO will be in regular contact with the site research team to check on progress and address any queries that they may have. Sponsor’s trial staff will check incoming Case Report Forms for compliance with the protocol, data consistency, missing data and timing. Sites will be sent Data Clarification Forms requesting missing data or clarification of inconsistencies or discrepancies.

Sites may be suspended from further recruitment in the event of serious and persistent non-compliance with the protocol and/or GCP, and/or poor recruitment. Any major problems identified during monitoring may be reported to the Trial Steering Committee and the relevant regulatory bodies. This includes reporting serious breaches of GCP and/or the trial protocol to the main Ethics Committee (EC) and the Competent Authorities.

### 14.4 Audit and Inspection

The Investigator will permit trial-related monitoring, audits, ethical review, and regulatory inspection(s) at their site, providing direct access to source data/documents, either by the sponsor, a third party vendor designated for this task by the sponsor and/or Pfizer. Sites are requested to notify the sponsor/ ECTC Trial Office immediately of any inspection (planned or unplanned) by regulatory bodies in their own country.

### 14.5 Notification of Serious Breaches (deviations/violations)

The Sponsor of the trial is responsible for reporting of any serious breach of:

- The conditions and principles of GCP in connection with the trial or;
- The protocol relating to the trial, within 7 days of becoming aware of that breach.

For this purpose, a “serious breach” is a breach which is likely to effect to a significant degree:

- The safety or physical or mental integrity of the subjects of the trial; or
- The scientific value of the trial.

Sites are therefore requested to notify the ECTC Trial Office of a suspected trial-related serious breach of GCP and/or the trial protocol. Where the ECTC Trial Office is investigating whether or not a serious breach has occurred sites are also requested to cooperate with the ECTC Trial Office in providing sufficient information to report the breach where required and in undertaking any corrective and/or preventive action.

## 15.0 ETHICAL CONSIDERATIONS

The study will be conducted in accordance with legal and regulatory requirements, as well as the general principles set forth in the International Ethical Guidelines for Biomedical Research Involving Human Subjects (Council for International Organizations of Medical Sciences 2002), Guidelines for GCP (ICH 1996), and the Declaration of Helsinki (World Medical Association 1996 and 2008 versions).

In addition, the study will be conducted in accordance with the protocol, the ICH guideline on GCP, and applicable local regulatory requirements and laws.

### 15.1 Institutional Review Board (IRB)/Ethics Committee (EC)

It is the responsibility of the Sponsor to have prospective approval of the study protocol, protocol amendments, informed consent documents, and other relevant documents, e.g., recruitment advertisements, if applicable, from the IRB/EC. All correspondence with the IRB/EC should be retained in the Investigator File. Copies of IRB/EC approvals should be forwarded to Erasmus MC.

It is the responsibility of the Sponsor to ensure that all subsequent amendments gain the necessary local approval. This does not affect the individual clinicians' responsibility to take immediate action if thought necessary to protect the health and interest of individual patients.

Certain Sponsor-related duties are delegated to the national coordinating centers and the Investigator as detailed in the contract.

The only circumstance in which an amendment may be initiated prior to IRB/EC approval is where the change is necessary to eliminate apparent immediate hazards to a patient. In that event, the Investigator must notify the IRB/EC and Erasmus MC in writing immediately after the implementation. Whenever possible it is advised to consult or notify the PI before implementation, and get approval from the sponsor.

### 15.2 Patient Information Sheet and Consent Form

The Patient Information Sheet and Consent Form must be in compliance with ICH GCP, local regulatory requirements, and legal requirements, including applicable privacy laws.
The informed consent document(s) used during the informed consent process must be reviewed by the Sponsor, approved by the IRB/EC before use, and available for inspection.

### 15.3 Patient Recruitment

Advertisements approved by ethics committees, Investigator outreach through cooperative groups, patient advocacy groups, and posting of the study outline on websites may be used as recruitment procedures.

### 15.4 Reporting of Safety Issues and Serious Breaches of the Protocol or ICH GCP

In the event of any prohibition or restriction imposed (i.e., clinical hold) by an applicable Competent Authority in any area of the World, or if the Investigator is aware of any new information which might influence the evaluation of the benefits and risks of the investigational product, Erasmus MC should be informed immediately.
In addition, the Investigator will inform Erasmus MC immediately of any urgent safety measures taken by the Investigator to protect the study patients against any immediate hazard, and of any serious breaches of this protocol or of ICH GCP that the Investigator becomes aware of.

### 15.5 Sponsor discontinuation criteria

Premature termination of this study may occur because of a regulatory authority decision, change in opinion of the IRB/EC, or significant safety of efficacy concerns. The conditions to discontinue may not conflict with the clinical study agreement.

If a study is prematurely terminated or discontinued, the sponsor will promptly notify the Investigators. After notification, the Investigators must contact all participating patients and the hospital pharmacy (if applicable) within 14 days. As directed by the sponsor, all study materials must be collected and all (E)CRFs completed to the greatest extent possible.

### 15.6 Risk-benefit analysis

The most important risks with regard to this study are standard risks for children treated with chemotherapy for ALL, such as bone marrow suppression and/or infections, and the side-effects of procedures such as repeated bone marrow aspiration and intrathecal chemotherapy administration.

Additional risks particularly concern the adverse events of InO, including possible unknown adverse events and an increased risk of VOD/SOS. In a randomized study with 326 adults with relapsed ALL, it was shown that about 80% of the patients had complete remission after treatment with InO compared to only 30% for patients treated with chemotherapy, with much higher rated of MRD-negativity in the InO arm, which translated in a significant increase in progression free and overall survival (Kantarijian et al., 2016).[83] However, patients treated with InO also clearly had a higher risk of SOS, especially following HSCT after InO-based reinduction chemotherapy.

Considering the activity of InO in adults it seems justified to study the activity of InO in children, as the prognosis for children with ALL who experience a 2$^{nd}$ or greater relapse, or who relapse after HSCT, or who are refractory to front-line therapy remains dismal, and there is no standardized treatment approach for these children.

In this study increasing dose levels of InO are used starting at a dose of 80% of the adult dose, therefore there will be no underdosing in the phase I part of this trial. Furthermore, only a limited number of dose-levels will be tested to prevent overdosing. The highest escalation dose-level is slightly higher than in adults but the recommended phase 2 dose in adults is not the maximum tolerated dose.

From the studies with InO in adults there is sufficient rationale to believe that included patients may have the chance to benefit from the study treatment. This is estimated to be in reasonable proportion compared to the side effects that can be expected from participating in the study. To reduce the risk of SOS mainly associated with HSCT following InO-based reinduction treatment the protocol summarizes some measures to mitigate this risk.

For patients with pediatric subtypes of NHL very few salvage options exist even at 1$^{st}$ relapse, apart from patients with lymphoblastic lymphoma, who are usually treated on ALL relapse protocols. Given the dismal prognosis treatment with InO may be justified to study whether this shows hints of activity in these patients.

As ALL therapy usually consists of combination chemotherapy to avoid resistance the combination of InO with UKALL-R3 based chemotherapy will be studied, to develop a new reinduction block that may be compared with the mitoxantrone-based UKALL-R3 reinduction schedule in a later randomized study as part of the PIP. Given the activity observed in adults, it is anticipated that InO may be more effective and potentially safer (depending on findings on liver toxicity) than mitoxantrone for reinduction of relapsed/refractory ALL.

## 16.0 CONFIDENTIALITY AND DATA PROTECTION

Information about study subjects will be kept confidential and managed under the applicable laws and regulations. Personal data recorded on all documents will be regarded as strictly confidential and will be handled and stored accordingly.
Patients will be identified using only their unique Study Subject Identification number, on the Case Report Form and correspondence between the ECTC Trial Office and the participating site.

The Investigator must maintain documents not for submission to the ECTC Trial Office (e.g. Patient Identification Logs) in strict confidence. In the case of specific issues and/or queries from the regulatory authorities, it will be necessary to have access to the complete trial records, provided that patient confidentiality is protected.

The ECTC Trial Office will maintain the confidentiality of all patients' data and will not disclose information by which patients may be identified to any third party. Representatives of the InO trial team may be required to have access to patient's notes for quality assurance purposes but patients should be reassured that their confidentiality will be respected at all times.

## 17.0 INSURANCE AND INDEMNITY

The sponsor as well as the study sites need a liability insurance and an clinical trial insurance to cover for potential damage to research subjects which is in accordance with local law and regulations.

## 18.0 PUBLICATION POLICY

The results of the clinical trial will be published after complete data collection and evaluation, but can be published if data collection is completed in a given stratum or cohort. Partial or preliminary results may not be published. Publications and/or presentations are to be initiated and/or authorized by the coordinating principal investigator together with the steering committee.

The following persons will be considered as co-authors:

- Members of the trial steering committee
- Investigators who have recruited at least 2 patients into the trial. In case the journal we want to publish the paper in does not allow a large number of co-authors this may have to be restricted to investigators enrolling at least 3 patients.

The complete study report (CSR) should be prepared within six months after last patient last visit, and will be reported to the EUDRACT database upon completion.

A manuscript will be prepared within one year after the last patient last visit (LPLV). The co-authors must notify the main author in writing concerning their approval or proposed changes to the manuscript within four weeks after receiving the publication draft. When failing to do this, their approval will be assumed.

Any publication in the form of a lecture, poster or publication of data must be approved by the coordinating investigator. Such publication should generally not occur before the joint publication of the study group. Enquiries from the press and general public concerning study results may only be answered by the coordinating investigator of the clinical trial after consultation with the sponsor.

## 19.0 REFERENCES


1. Brivio E, Lopez-Yurda M, Ownes C, et al. A Phase I Study of Single-Agent Inotuzumab Ozogamicin in Pediatric CD22-Positive Relapsed/Refractory Acute Lymphoblastic Leukemia: Preliminary Results of the ITCC-059 Study. *Blood.* 2019;134(Supplement_1):2629-2629.
2. Jabbour E, Sasaki K, Ravandi F, et al. Chemoimmunotherapy with inotuzumab ozogamicin combined with mini-hyper-CVD, with or without blinatumomab, is highly effective in patients with Philadelphia chromosome-negative acute lymphoblastic leukemia in first salvage. *Cancer.* 2018;124(20):4044-4055.
3. Advani AS, Moseley A, Liedtke M, et al. SWOG 1312 Final Results: A Phase 1 Trial of Inotuzumab in Combination with CVP (Cyclophosphamide, Vincristine, Prednisone) for Relapsed/ Refractory CD22+ Acute Leukemia. *Blood.* 2019;134(Supplement_1):227-227.
4. Meikle AW, Tyler FH. Potency and duration of action of glucocorticoids. Effects of hydrocortisone, prednisone and dexamethasone on human pituitary-adrenal function. *Am J Med.* 1977;63(2):200-207.
5. Brown Patrick. Personal communication. In:2020.
6. Von Stackelberg Arend. Personal communication. In:2020.
7. Krentz S, Hof J, Mendioroz A, et al. Prognostic value of genetic alterations in children with first bone marrow relapse of childhood B-cell precursor acute lymphoblastic leukemia. *Leukemia.* 2013;27(2):295-304.
8. Eckert C, Groeneveld-Krentz S, Kirschner-Schwabe R, et al. Improving Stratification for Children With Late Bone Marrow B-Cell Acute Lymphoblastic Leukemia Relapses With Refined Response Classification and Integration of Genetics. *Journal of Clinical Oncology.* 2019;37(36):3493-3506.
9. Parker C, Krishnan S, Hamadeh L, et al. Outcomes of patients with childhood B-cell precursor acute lymphoblastic leukaemia with late bone marrow relapses: long-term follow-up of the ALLR3 open-label randomised trial. *Lancet Haematol.* 2019;6(4):e204-e216.
10. Eckert C, von Stackelberg A, Seeger K, et al. Minimal residual disease after induction is the strongest predictor of prognosis in intermediate risk relapsed acute lymphoblastic leukaemia - long-term results of trial ALL-REZ BFM P95/96. *Eur J Cancer.* 2013;49(6):1346-1355.
11. Brown PA, Ji L, Xu X, et al. A Randomized Phase 3 Trial of Blinatumomab Vs. Chemotherapy As Post-Reinduction Therapy in High and Intermediate Risk (HR/IR) First Relapse of B-Acute Lymphoblastic Leukemia (B-ALL) in Children and Adolescents/Young Adults (AYAs) Demonstrates Superior Efficacy and Tolerability of Blinatumomab: A Report from Children's Oncology Group Study AALL1331. *Blood.* 2019;134(Supplement_2):LBA-1-LBA-1.
12. Schmiegelow K, Attarbaschi A, Barzilai S, et al. Consensus definitions of 14 severe acute toxic effects for childhood lymphoblastic leukaemia treatment: a Delphi consensus. *Lancet Oncol.* 2016;17(6):e231-e239.
13. McNeil DE, Cote TR, Clegg L, Mauer A. SEER update of incidence and trends in pediatric malignancies: acute lymphoblastic leukemia. *Med Pediatr Oncol.* 2002;39(6):554-557; discussion 552-553.
14. Mitchell C, Richards S, Harrison CJ, Eden T. Long-term follow-up of the United Kingdom medical research council protocols for childhood acute lymphoblastic leukaemia, 1980-2001. *Leukemia.* 2010;24(2):406-418.
15. Moricke A, Zimmermann M, Reiter A, et al. Long-term results of five consecutive trials in childhood acute lymphoblastic leukemia performed by the ALL-BFM study group from 1981 to 2000. *Leukemia.* 2010;24(2):265-284.
16. Pui CH, Sandlund JT, Pei D, et al. Improved outcome for children with acute lymphoblastic leukemia: results of Total Therapy Study XIIIB at St Jude Children's Research Hospital. *Blood.* 2004;104(9):2690-2696.
17. Gaynon PS. Childhood acute lymphoblastic leukaemia and relapse. *Br J Haematol.* 2005;131(5):579-587.
18. Einsiedel HG, von Stackelberg A, Hartmann R, et al. Long-term outcome in children with relapsed ALL by risk-stratified salvage therapy: results of trial acute lymphoblastic leukemia-relapse study of the Berlin-Frankfurt-Munster Group 87. *J Clin Oncol.* 2005;23(31):7942-7950.

19. Henze G, Fengler R, Hartmann R, et al. Six-year experience with a comprehensive approach to the treatment of recurrent childhood acute lymphoblastic leukemia (ALL-REZ BFM 85). A relapse study of the BFM group. *Blood.* 1991;78(5):1166-1172.
20. Tallen G, Ratei R, Mann G, et al. Long-term outcome in children with relapsed acute lymphoblastic leukemia after time-point and site-of-relapse stratification and intensified short-cycle multidrug chemotherapy: results of trial ALL-REZ BFM 90. *J Clin Oncol.* 2010;28(14):2339-2347.
21. Maude SL, Frey N, Shaw PA, et al. Chimeric antigen receptor T cells for sustained remissions in leukemia. *N Engl J Med.* 2014;371(16):1507-1517.
22. Roy A, Cargill A, Love S, et al. Outcome after first relapse in childhood acute lymphoblastic leukaemia - lessons from the United Kingdom R2 trial. *Br J Haematol.* 2005;130(1):67-75.
23. Rizzari C, Valsecchi MG, Arico M, et al. Outcome of very late relapse in children with acute lymphoblastic leukemia. *Haematologica.* 2004;89(4):427-434.
24. Raetz EA, Borowitz MJ, Devidas M, et al. Reinduction platform for children with first marrow relapse of acute lymphoblastic Leukemia: A Children's Oncology Group Study[corrected]. *J Clin Oncol.* 2008;26(24):3971-3978.
25. Paganin M, Zecca M, Fabbri G, et al. Minimal residual disease is an important predictive factor of outcome in children with relapsed 'high-risk' acute lymphoblastic leukemia. *Leukemia.* 2008;22(12):2193-2200.
26. Hagedorn N, Acquaviva C, Fronkova E, et al. Submicroscopic bone marrow involvement in isolated extramedullary relapses in childhood acute lymphoblastic leukemia: a more precise definition of "isolated" and its possible clinical implications, a collaborative study of the Resistant Disease Committee of the International BFM study group. *Blood.* 2007;110(12):4022-4029.
27. von Stackelberg A, Völzke E, Kühl JS, et al. Outcome of children and adolescents with relapsed acute lymphoblastic leukaemia and non-response to salvage protocol therapy: a retrospective analysis of the ALL-REZ BFM Study Group. *Eur J Cancer.* 2011;47(1):90-97.
28. A vS. Conventional Reinduction/Consolidation-Type Therapy Versus Short Cycle High Intensity Combination Chemotherapy As Post-Induction Treatment for Children with Relapsed Acute Lymphoblastic Leukemia. Early Results of Study ALL-REZ BFM 2002. In. Vol 118:871-: Blood; 2011.
29. Parker C, Waters R, Leighton C, et al. Effect of mitoxantrone on outcome of children with first relapse of acute lymphoblastic leukaemia (ALL R3): an open-label randomised trial. *Lancet.* 2010;376(9757):2009-2017.
30. Sun W, Orgel E, Malvar J, et al. Treatment-related adverse events associated with a modified UK ALLR3 induction chemotherapy backbone for childhood relapsed/refractory acute lymphoblastic leukemia. *Pediatr Blood Cancer.* 2016;63(11):1943-1948.
31. Jeha S, Gandhi V, Chan KW, et al. Clofarabine, a novel nucleoside analog, is active in pediatric patients with advanced leukemia. *Blood.* 2004;103(3):784-789.
32. Raetz EA, Cairo MS, Borowitz MJ, et al. Chemoimmunotherapy reinduction with epratuzumab in children with acute lymphoblastic leukemia in marrow relapse: a Children's Oncology Group Pilot Study. *J Clin Oncol.* 2008;26(22):3756-3762.
33. Messinger YH, Gaynon PS, Sposto R, et al. Bortezomib with chemotherapy is highly active in advanced B-precursor acute lymphoblastic leukemia: Therapeutic Advances in Childhood Leukemia & Lymphoma (TACL) Study. *Blood.* 2012;120(2):285-290.
34. Bhojwani D, Pui CH. Relapsed childhood acute lymphoblastic leukaemia. *Lancet Oncol.* 2013;14(6):e205-217.
35. Chessells JM, Veys P, Kempski H, et al. Long-term follow-up of relapsed childhood acute lymphoblastic leukaemia. *Br J Haematol.* 2003;123(3):396-405.
36. Saarinen-Pihkala UM, Heilmann C, Winiarski J, et al. Pathways through relapses and deaths of children with acute lymphoblastic leukemia: role of allogeneic stem-cell transplantation in Nordic data. *J Clin Oncol.* 2006;24(36):5750-5762.
37. Ko RH, Ji L, Barnette P, et al. Outcome of patients treated for relapsed or refractory acute lymphoblastic leukemia: a Therapeutic Advances in Childhood Leukemia Consortium study. *J Clin Oncol.* 2010;28(4):648-654.
38. Jeha S, Gaynon PS, Razzouk BI, et al. Phase II study of clofarabine in pediatric patients with refractory or relapsed acute lymphoblastic leukemia. *J Clin Oncol.* 2006;24(12):1917-1923.

39. Hijiya N, Thomson B, Isakoff MS, et al. Phase 2 trial of clofarabine in combination with etoposide and cyclophosphamide in pediatric patients with refractory or relapsed acute lymphoblastic leukemia. *Blood.* 2011;118(23):6043-6049.
40. von Stackelberg A, Locatelli F, Zugmaier G, et al. Phase I/Phase II Study of Blinatumomab in Pediatric Patients With Relapsed/Refractory Acute Lymphoblastic Leukemia. *J Clin Oncol.* 2016;34(36):4381-4389.
41. Messinger Y, Gaynon P, Raetz E, et al. Phase I study of bortezomib combined with chemotherapy in children with relapsed childhood acute lymphoblastic leukemia (ALL): a report from the therapeutic advances in childhood leukemia (TACL) consortium. *Pediatr Blood Cancer.* 2010;55(2):254-259.
42. Nitschke L, Floyd H, Crocker PR. New functions for the sialic acid-binding adhesion molecule CD22, a member of the growing family of Siglecs. *Scand J Immunol.* 2001;53(3):227-234.
43. Tedder TF, Tuscano J, Sato S, Kehrl JH. CD22, a B lymphocyte-specific adhesion molecule that regulates antigen receptor signaling. *Annu Rev Immunol.* 1997;15:481-504.
44. Shan D, Press OW. Constitutive endocytosis and degradation of CD22 by human B cells. *J Immunol.* 1995;154(9):4466-4475.
45. Tedder TF, Poe JC, Haas KM. CD22: a multifunctional receptor that regulates B lymphocyte survival and signal transduction. *Adv Immunol.* 2005;88:1-50.
46. Doody GM, Justement LB, Delibrias CC, et al. A role in B cell activation for CD22 and the protein tyrosine phosphatase SHP. *Science.* 1995;269(5221):242-244.
47. Nitschke L, Carsetti R, Ocker B, Kohler G, Lamers MC. CD22 is a negative regulator of B-cell receptor signalling. *Curr Biol.* 1997;7(2):133-143.
48. Boue DR, LeBien TW. Expression and structure of CD22 in acute leukemia. *Blood.* 1988;71(5):1480-1486.
49. Moyron-Quiroz JE, Partida-Sanchez S, Donis-Hernandez R, Sandoval-Montes C, Santos-Argumedo L. Expression and function of CD22, a B-cell restricted molecule. *Scand J Immunol.* 2002;55(4):343-351.
50. Childhood Non-Hodgkin Lymphoma Treatment (PDQ®)–Health Professional Version p*ublished by the National Cancer Institute*. In.
51. Burkhardt B, Lenz G. XI. Management of paediatric and adult non-Hodgkin lymphoma: what lessons can each teach the other? *Hematol Oncol.* 2015;33 Suppl 1:62-66.
52. Minard-Colin V, Brugieres L, Reiter A, et al. Non-Hodgkin Lymphoma in Children and Adolescents: Progress Through Effective Collaboration, Current Knowledge, and Challenges Ahead. *J Clin Oncol.* 2015;33(27):2963-2974.
53. Trail PA, Bianchi AB. Monoclonal antibody drug conjugates in the treatment of cancer. *Curr Opin Immunol.* 1999;11(5):584-588.
54. Hinman LM, Hamann PR, Wallace R, Menendez AT, Durr FE, Upeslacis J. Preparation and characterization of monoclonal antibody conjugates of the calicheamicins: a novel and potent family of antitumor antibiotics. *Cancer Res.* 1993;53(14):3336-3342.
55. Hanna R, Ong GL, Mattes MJ. Processing of antibodies bound to B-cell lymphomas and other hematological malignancies. *Cancer Res.* 1996;56(13):3062-3068.
56. Li JL, Shen GL, Ghetie MA, et al. The epitope specificity and tissue reactivity of four murine monoclonal anti-CD22 antibodies. *Cell Immunol.* 1989;118(1):85-99.
57. Zein N, Sinha AM, McGahren WJ, Ellestad GA. Calicheamicin gamma 1I: an antitumor antibiotic that cleaves double-stranded DNA site specifically. *Science.* 1988;240(4856):1198-1201.
58. Lee MD, Manning JK, Williams DR, Kuck NA, Testa RT, Borders DB. Calicheamicins, a novel family of antitumor antibiotics. 3. Isolation, purification and characterization of calicheamicins beta 1Br, gamma 1Br, alpha 2I, alpha 3I, beta 1I, gamma 1I and delta 1I. *J Antibiot (Tokyo).* 1989;42(7):1070-1087.
59. Bross PF, Beitz J, Chen G, et al. Approval summary: gemtuzumab ozogamicin in relapsed acute myeloid leukemia. *Clin Cancer Res.* 2001;7(6):1490-1496.
60. Sievers EL. Efficacy and safety of gemtuzumab ozogamicin in patients with CD33-positive acute myeloid leukaemia in first relapse. *Expert Opin Biol Ther.* 2001;1(5):893-901.
61. Hamann PR, Hinman LM, Hollander I, et al. Gemtuzumab ozogamicin, a potent and selective anti-CD33 antibody-calicheamicin conjugate for treatment of acute myeloid leukemia. *Bioconjug Chem.* 2002;13(1):47-58.

62. Zwaan CM, Reinhardt D, Jurgens H, et al. Gemtuzumab ozogamicin in pediatric CD33-positive acute lymphoblastic leukemia: first clinical experiences and relation with cellular sensitivity to single agent calicheamicin. *Leukemia.* 2003;17(2):468-470.
63. Shor B, Gerber HP, Sapra P. Preclinical and clinical development of inotuzumab-ozogamicin in hematological malignancies. *Mol Immunol.* 2015;67(2 Pt A):107-116.
64. DiJoseph JF, Armellino DC, Boghaert ER, et al. Antibody-targeted chemotherapy with CMC-544: a CD22-targeted immunoconjugate of calicheamicin for the treatment of B-lymphoid malignancies. *Blood.* 2004;103(5):1807-1814.
65. DiJoseph JF, Goad ME, Dougher MM, et al. Potent and specific antitumor efficacy of CMC-544, a CD22-targeted immunoconjugate of calicheamicin, against systemically disseminated B-cell lymphoma. *Clin Cancer Res.* 2004;10(24):8620-8629.
66. de Vries JF, Zwaan CM, De Bie M, et al. The novel calicheamicin-conjugated CD22 antibody inotuzumab ozogamicin (CMC-544) effectively kills primary pediatric acute lymphoblastic leukemia cells. *Leukemia.* 2012;26(2):255-264.
67. DiJoseph JF, Dougher MM, Evans DY, Zhou BB, Damle NK. Preclinical anti-tumor activity of antibody-targeted chemotherapy with CMC-544 (inotuzumab ozogamicin), a CD22-specific immunoconjugate of calicheamicin, compared with non-targeted combination chemotherapy with CVP or CHOP. *Cancer Chemother Pharmacol.* 2011;67(4):741-749.
68. DiJoseph JF, Popplewell A, Tickle S, et al. Antibody-targeted chemotherapy of B-cell lymphoma using calicheamicin conjugated to murine or humanized antibody against CD22. *Cancer Immunol Immunother.* 2005;54(1):11-24.
69. Dijoseph JF, Dougher MM, Armellino DC, Evans DY, Damle NK. Therapeutic potential of CD22-specific antibody-targeted chemotherapy using inotuzumab ozogamicin (CMC-544) for the treatment of acute lymphoblastic leukemia. *Leukemia.* 2007;21(11):2240-2245.
70. Goy A, Forero A, Wagner-Johnston N, et al. A phase 2 study of inotuzumab ozogamicin in patients with indolent B-cell non-Hodgkin lymphoma refractory to rituximab alone, rituximab and chemotherapy, or radioimmunotherapy. *Br J Haematol.* 2016;174(4):571-581.
71. Fayad L, Offner F, Smith MR, et al. Safety and clinical activity of a combination therapy comprising two antibody-based targeting agents for the treatment of non-Hodgkin lymphoma: results of a phase I/II study evaluating the immunoconjugate inotuzumab ozogamicin with rituximab. *J Clin Oncol.* 2013;31(5):573-583.
72. Wagner-Johnston ND, Goy A, Rodriguez MA, et al. A phase 2 study of inotuzumab ozogamicin and rituximab, followed by autologous stem cell transplant in patients with relapsed/refractory diffuse large B-cell lymphoma. *Leuk Lymphoma.* 2015;56(10):2863-2869.
73. Ogura M, Tobinai K, Hatake K, et al. Phase I Study of Inotuzumab Ozogamicin Combined with R-CVP for Relapsed/Refractory CD22+ B-cell Non-Hodgkin Lymphoma. *Clin Cancer Res.* 2016;22(19):4807-4816.
74. Sangha R, Davies A, Dang NH, et al. Phase 1 study of inotuzumab ozogamicin combined with R-GDP for the treatment of patients with relapsed/refractory CD22+ B-cell non-Hodgkin lymphoma. *J Drug Assess.* 2017;6(1):10-17.
75. Dang NH, Ogura M, Castaigne S, et al. Randomized, phase 3 trial of inotuzumab ozogamicin plus rituximab versus chemotherapy plus rituximab for relapsed/refractory aggressive B-cell non-Hodgkin lymphoma. *Br J Haematol.* 2018;182(4):583-586.
76. Kantarjian H, Thomas D, Jorgensen J, et al. Inotuzumab ozogamicin, an anti-CD22-calecheamicin conjugate, for refractory and relapsed acute lymphocytic leukaemia: a phase 2 study. *Lancet Oncol.* 2012;13(4):403-411.
77. Kantarjian H, Thomas D, Jorgensen J, et al. Results of inotuzumab ozogamicin, a CD22 monoclonal antibody, in refractory and relapsed acute lymphocytic leukemia. *Cancer.* 2013;119(15):2728-2736.
78. Rytting M, Triche L, Thomas D, O'Brien S, Kantarjian H. Initial experience with CMC-544 (inotuzumab ozogamicin) in pediatric patients with relapsed B-cell acute lymphoblastic leukemia. *Pediatr Blood Cancer.* 2014;61(2):369-372.
79. DeAngelo DJ, Stock W, Stein AS, et al. Inotuzumab ozogamicin in adults with relapsed or refractory CD22-positive acute lymphoblastic leukemia: a phase 1/2 study. *Blood Adv.* 2017;1(15):1167-1180.

80. al. DDe. Weekly Inotuzumab Ozogamicin in Adult Patients with Relapsed or Refractory CD22-Positive Acute Lymphoblastic Leukemia. In:Blood 2012 120:2612.
81. DeAngelo D. Efficacy and safety of inotuzumab ozogamicin (INO) vs standard of care (SOC) in salvage 1 or 2 patients with acute lymphoblastic leukemia (ALL): An ongoing global phase 3 study. In:EHA Learning Center. Jun 14, 2015; 103387.
82. Kantarjian HM, DeAngelo DJ, Advani AS, et al. Hepatic adverse event profile of inotuzumab ozogamicin in adult patients with relapsed or refractory acute lymphoblastic leukaemia: results from the open-label, randomised, phase 3 INO-VATE study. *Lancet Haematol.* 2017;4(8):e387-e398.
83. Kantarjian HM, DeAngelo DJ, Stelljes M, et al. Inotuzumab Ozogamicin versus Standard Therapy for Acute Lymphoblastic Leukemia. *N Engl J Med.* 2016;375(8):740-753.
84. Bhojwani D, Sposto R, Shah NN, et al. Inotuzumab ozogamicin in pediatric patients with relapsed/refractory acute lymphoblastic leukemia. *Leukemia.* 2018.
85. Guffroy M, Falahatpisheh H, Biddle K, et al. Liver Microvascular Injury and Thrombocytopenia of Antibody-Calicheamicin Conjugates in Cynomolgus Monkeys-Mechanism and Monitoring. *Clin Cancer Res.* 2017;23(7):1760-1770.
86. K. S, al. e. Inotuzumab Ozogamicin in Combination with Low-Intensity Chemotherapy (mini-hyper-CVD) As Frontline Therapy for Older Patients with Acute Lymphoblastic Leukemia (ALL): Interim Result of a Phase II Clinical Trial. In:Blood 2016 128:588;.
87. Kantarjian H, Ravandi F, Short NJ, et al. Inotuzumab ozogamicin in combination with low-intensity chemotherapy for older patients with Philadelphia chromosome-negative acute lymphoblastic leukaemia: a single-arm, phase 2 study. *Lancet Oncol.* 2018;19(2):240-248.
88. Gamis AS, Alonzo TA, Meshinchi S, et al. Gemtuzumab ozogamicin in children and adolescents with de novo acute myeloid leukemia improves event-free survival by reducing relapse risk: results from the randomized phase III Children's Oncology Group trial AAML0531. *J Clin Oncol.* 2014;32(27):3021-3032.
89. Walter RB, Gooley TA, van der Velden VH, et al. CD33 expression and P-glycoprotein-mediated drug efflux inversely correlate and predict clinical outcome in patients with acute myeloid leukemia treated with gemtuzumab ozogamicin monotherapy. *Blood.* 2007;109(10):4168-4170.
90. Jedema I, Barge RM, van der Velden VH, et al. Internalization and cell cycle-dependent killing of leukemic cells by Gemtuzumab Ozogamicin: rationale for efficacy in CD33-negative malignancies with endocytic capacity. *Leukemia.* 2004;18(2):316-325.
91. van der Velden VH, Boeckx N, Jedema I, et al. High CD33-antigen loads in peripheral blood limit the efficacy of gemtuzumab ozogamicin (Mylotarg) treatment in acute myeloid leukemia patients. *Leukemia.* 2004;18(5):983-988.
92. van Der Velden VH, te Marvelde JG, Hoogeveen PG, et al. Targeting of the CD33-calicheamicin immunoconjugate Mylotarg (CMA-676) in acute myeloid leukemia: in vivo and in vitro saturation and internalization by leukemic and normal myeloid cells. *Blood.* 2001;97(10):3197-3204.
93. Raetz EA, Salzer WL. Tolerability and efficacy of L-asparaginase therapy in pediatric patients with acute lymphoblastic leukemia. *J Pediatr Hematol Oncol.* 2010;32(7):554-563.
94. Graham ML. Pegaspargase: a review of clinical studies. *Adv Drug Deliv Rev.* 2003;55(10):1293-1302.
95. Andre Baruchel ea. TISAGENLECLEUCEL FOR PEDIATRIC/YOUNG ADULT PATIENTS WITH RELAPSED/REFRACTORY B-CELL ACUTE LYMPHOBLASTIC LEUKEMIA: PRELIMINARY REPORT OF B2001X FOCUSING ON PRIOR EXPOSURE TO BLINATUMOMAB AND INOTUZUMAB. European Hematology Association 25th; 06-12-2020.
96. Personal Communication [press release].
97. Skolnik JM, Barrett JS, Jayaraman B, Patel D, Adamson PC. Shortening the timeline of pediatric phase I trials: the rolling six design. *J Clin Oncol.* 2008;26(2):190-195.
98. Paoletti X, Doussau A, Ezzalfani M, Rizzo E, Thiebaut R. Dose finding with longitudinal data: simpler models, richer outcomes. *Stat Med.* 2015;34(22):2983-2998.
99. McDonald GB, Hinds MS, Fisher LD, et al. Veno-occlusive disease of the liver and multiorgan failure after bone marrow transplantation: a cohort study of 355 patients. *Ann Intern Med.* 1993;118(4):255-267.

100. Richardson PG, Soiffer RJ, Antin JH, et al. Defibrotide for the treatment of severe hepatic veno-occlusive disease and multiorgan failure after stem cell transplantation: a multicenter, randomized, dose-finding trial. *Biol Blood Marrow Transplant.* 2010;16(7):1005-1017.
101. Corbacioglu S, Cesaro S, Faraci M, et al. Defibrotide for prophylaxis of hepatic veno-occlusive disease in paediatric haemopoietic stem-cell transplantation: an open-label, phase 3, randomised controlled trial. *Lancet.* 2012;379(9823):1301-1309.
102. Bader P, Kreyenberg H, Henze GH, et al. Prognostic value of minimal residual disease quantification before allogeneic stem-cell transplantation in relapsed childhood acute lymphoblastic leukemia: the ALL-REZ BFM Study Group. *J Clin Oncol.* 2009;27(3):377-384.
103. Ruutu T, Eriksson B, Remes K, et al. Ursodeoxycholic acid for the prevention of hepatic complications in allogeneic stem cell transplantation. *Blood.* 2002;100(6):1977-1983.
104. Ruutu T, Gratwohl A, de Witte T, et al. Prophylaxis and treatment of GVHD: EBMT-ELN working group recommendations for a standardized practice. *Bone Marrow Transplant.* 2014;49(2):168-173.
105. Dignan FL, Wynn RF, Hadzic N, et al. BCSH/BSBMT guideline: diagnosis and management of veno-occlusive disease (sinusoidal obstruction syndrome) following haematopoietic stem cell transplantation. *Br J Haematol.* 2013;163(4):444-457.
106. Peters C, Schrappe M, von Stackelberg A, et al. Stem-cell transplantation in children with acute lymphoblastic leukemia: A prospective international multicenter trial comparing sibling donors with matched unrelated donors-The ALL-SCT-BFM-2003 trial. *J Clin Oncol.* 2015;33(11):1265-1274.
107. Boztug H, Zecca M, Sykora KW, et al. Treosulfan-based conditioning regimens for allogeneic HSCT in children with acute lymphoblastic leukaemia. *Ann Hematol.* 2015;94(2):297-306.
108. Boelens J, al. ZMe. Combining Clofarabine/Fludarabine with Exposure Targeted Busulfan for Pediatric Leukemia: An Effective, Low Toxic TBI-Free Conditioning Regimen. In:Biol Blood Marrow Tr 2015 Volume 21, Issue 2, Supplement, Pages S93–S94.
109. Hoelzer D, Gokbuget N. Chemoimmunotherapy in acute lymphoblastic leukemia. *Blood Rev.* 2012;26(1):25-32.
110. Le Tourneau C, Lee JJ, Siu LL. Dose escalation methods in phase I cancer clinical trials. *J Natl Cancer Inst.* 2009;101(10):708-720.
111. National Comprehensive Cancer Network Criteria. In:at http://www.nccn.org/.
112. Sandlund JT, Guillerman RP, Perkins SL, et al. International Pediatric Non-Hodgkin Lymphoma Response Criteria. *J Clin Oncol.* 2015;33(18):2106-2111.
113. Jones RJ, Lee KS, Beschorner WE, et al. Venoocclusive disease of the liver following bone marrow transplantation. *Transplantation.* 1987;44(6):778-783.
114. Carreras E, Diaz-Beya M, Rosinol L, Martinez C, Fernandez-Aviles F, Rovira M. The incidence of veno-occlusive disease following allogeneic hematopoietic stem cell transplantation has diminished and the outcome improved over the last decade. *Biol Blood Marrow Transplant.* 2011;17(11):1713-1720.
115. Mosteller RD. Simplified calculation of body-surface area. *N Engl J Med.* 1987;317(17):1098.

## APPENDIX 1: PERFORMANCE STATUS SCALES / SCORES

| **Karnofsky** | | **Lansky** | |
|---|---|---|---|
| Score | Description | Score | Description |
| 100 | Normal, no complaints, no evidence of disease | 100 | Fully active, normal. |
| 90 | Able to carry on normal activity, minor signs or symptoms of disease. | 90 | Minor restrictions in physically strenuous activity. |
| 80 | Normal activity with effort; some signs or symptoms of disease. | 80 | Active, but tires more quickly |
| 70 | Cares for self, unable to carry on normal activity or do active work. | 70 | Both greater restriction of and less time spent in play activity. |
| 60 | Required occasional assistance, but is able to care for most of his/her needs. | 60 | Up and around, but minimal active play; keeps busy with quieter activities. |
| 50 | Requires considerable assistance and frequent medical care. | 50 | Gets dressed, but lies around much of the day; no active play, able to participate in all quiet play and activities. |
| 40 | Disabled, requires special care and assistance. | 40 | Mostly in bed; participates in quiet activities. |
| 30 | Severely disabled, hospitalization indicated. Death not imminent. | 30 | In bed; needs assistance even for quiet play. |
| 20 | Very sick, hospitalization indicated. Death not imminent. | 20 | Often sleeping; play entirely limited to very passive activities. |
| 10 | Moribund, fatal processes progressing rapidly. | 10 | No play; does not get out of bed. |

## APPENDIX 2: RESPONSE CRITERIA

### APPENDIX 2.1. Definitions and Response Criteria for BCP-ALL patients

#### 2.1.1. Definitions Bone Marrow Morphology Status

- **M1 Marrow**
  Less than 5% blasts in a bone marrow aspirate and at least 200 cells counted.

- **M2 Marrow**
  5-25% blasts in a bone marrow aspirate with at least 200 cells counted.

- **M3 Marrow**
  Greater than 25% blasts in a bone marrow aspirate with at least 200 cells counted.

#### 2.1.2. Bone Marrow Response Criteria

These criteria were modified from the National Comprehensive Cancer Network Criteria especially with respect for hematopoietic regeneration (platelets 50,000/µL and not 100,000/µL and ANC >500/µL and not 1000/µL), taking into account that most patients will have been transplanted and that hematopoietic regeneration may be hampered despite remission of leukemia. Since this mostly affects platelet regeneration, we distinguish between CRp and CRi, in which respectively platelet regeneration or both platelet and ANC regeneration are below the level of recovery required for Cr designation. Moreover, the categories of PR and SD were defined.

**Definitions of response:**

- **Complete Response (CR)**
  - No evidence of circulating blasts or extramedullary disease; including CN1 status; absence of splenomegaly, lymphadenopathy, skin/gum infiltration, testicular mass
  - A bone marrow with <5% blasts (M1 marrow); and
  - Recovery of peripheral counts (platelets >50,000/µL and transfusion independent, and ANC >500/µL)

- **Complete Response with insufficient platelet recovery (CRp)**
  - No evidence of circulating blasts or extramedullary disease;
  - A bone marrow with <5% blasts (M1 marrow); and
  - An ANC > 500/µL but Platelets ≤ 50,000/µL

- **Complete Response without recovery of counts (CRi)**
  - No evidence of circulating blasts or extramedullary disease;
  - A bone marrow with <5% blasts (M1 marrow); and
  - An ANC ≤ 500/µL and / or Platelets ≤50,000/µL

- **Partial Response (PR)**
  Greater than 50% relative reduction (with a minimum of 10% absolute reduction) in the bone marrow aspirate leukemic cell count, irrespective of recovery of the peripheral blood counts.

- **Non-Response or Stable Disease (SD)**
  Stable disease is present when the patient fails to qualify for CR, CRi, PR, or PD.

- **Progressive Disease (PD)**
  Progressive disease is defined as an increase of at least 25% of the absolute number of bone marrow or circulating leukemic blasts, development of extramedullary disease, or other laboratory or clinical evidence of PD, with or without recovery of ANC or platelets.

- **Induction Death (ID)**
  Any patient who dies after receiving therapy on this protocol during the first cycle before a final bone-marrow evaluation for response was performed.

**Definition of refractory disease:**

Any patient not achieving CR, CPp or CRi after induction therapy (cycle 1 and/or cycle 2).

**Definition of Relapse:**

After documentation of remission, a bone marrow aspirate and/or biopsy showing ≥5% leukemic blasts using morphology with flow cytometric confirmation, and/or pathological/radiological evidence of extramedullary disease, including development of CNS3 status or clinical CNS-involvement with radiological confirmation (MRI).

**Definition of Overall Response Rate**

The number of patients with CR, CRi, CRp.

**Definition of Minimal Residual Disease Complete Response**

Bone marrow is considered as 'MRD complete response' if the result is less than 0.01% by flow cytometry or < $1x10^{-4}$ by PCR.

**Definitions of survival estimates:**

- **Event-free survival (EFS)**
  The time between start of study treatment and first event including failure to achieve CR/CRp/CRi (calculated as an event on day 0), relapse, death of any cause and second malignancies.
- **Overall survival (OS)**
  The time of start of study treatment and death.
- **The cumulative incidence of non-response or relapse (CIR)**
  The cumulative probability of non-response or relapse, with time calculated between start of study treatment and relapse, and with non-responders included as an event on day 0. Non-relapse death is considered a competing event.
- **The cumulative incidence of non-relapse mortality**
  The cumulative probability of non-relapse mortality, with time calculated between start of study treatment and death due to other causes than relapse or resistant leukemia. Relapsed or refractory leukemia or lymphoma is considered a competing event.
- **Duration of response**
  The time between achieving response (CR, CRi or CRp) after starting study treatment and documented relapse or death.

### 2.1.3. CNS Status Definitions

**CNS 1**: In cerebral spinal fluid (CSF), presence < 5/μL WBCs and absence of blasts on cytospin preparation

**CNS 2:** In CSF, presence < 5/μL WBCs and cytospin positive for blasts

**CNS 3**: In CSF, presence of ≥ 5/μL WBCs and cytospin positive for blasts **and/or** clinical signs of CNS leukemia

## APPENDIX 2.2 Response criteria for pediatric NHL patients [112]

Table 1 below summarizes the response criteria. In cases with multiple masses, up to 6 of the most representative nodal or extranodal masses should be selected for measurement.

To grade FDG uptake for metabolic response assessment on PET, the Deauville criteria 5-point scale should be used.

### Definition of response

### CR (Complete response)

The CR designation will be used to indicate the disappearance of all disease; however, there will be sub-classification of this designation to indicate how this designation was determined.

- **CR.** The CR designation indicates the complete disappearance of all disease, as confirmed by physical examination, CT, or MRI and examination of CSF and BM. Specifically, the CT or MRI should be free of residual mass or evidence of new disease. FDG-PET should be negative (Deauville criteria score of 1, 2, or 3 [unless score of 3 is considered as inadequate response to avoid under-treatment, such as in de-escalation trial]). If a residual or new mass is present but has been completely resected and is negative for disease by pathologic evaluation of morphology, a CR designation is still assigned. Evaluation of BM and CSF should be negative for morphologic evidence of disease as well for the CR designation. Detection of disease using more sensitive techniques, such as immunophenotyping or molecular techniques should be indicated as supporting data, as summarized in Table 2. There should be no new and/or progressive disease (PD) elsewhere[112].
- **CR biopsy negative.** The CR biopsy negative (CRb) designation is for patients who otherwise meet the CR designation but have a residual mass on CT or MRI that is biopsied (not resected) and found to be negative for disease based on pathologic evaluation of morphology. If disease is detected by more sensitive tools (eg, molecular techniques, flow cytometry, IHC, cytogenetics), this should be indicated as supporting data, as summarized in Table 2. Although a biopsy of a residual mass that is negative for viable tumor provides some reassurance, there is always a possibility of sampling error. Thus, a CRb designation is included until there are more data showing that a negative biopsy is equivalent to a negative morphologic examination of a completely resected residual mass and/or until there are adequate PET data to confidently exclude the need for biopsy of a PET-negative residual mass. The decision to biopsy an FDG-PET–positive bone lesion is a clinical judgment based on symptoms and level of concern about risk associated with biopsy of a weight-bearing long bone.
- **CR unconfirmed.** The CR unconfirmed designation is applied in otherwise CR cases in which a residual mass on CT or MRI is negative by FDG-PET imaging. BM and CSF must be morphologically negative for tumor. There should be no new and/or PD elsewhere.

### PR (partial response)

The PR designation is assigned when there has been ≥ 50% decrease in the SPD by CT or MRI. The FDG-PET imaging results may be positive (Deauville score of 4 or 5 with reduced lesional uptake compared with baseline). Morphologic detection of disease in a biopsy sample of the mass may be present. There may also be persistent morphologic detection of disease in the BM and CSF if this finding was present at diagnosis; however, there should be a 50% reduction in the percentage of lymphoma cells. There should be no new and/or PD elsewhere.

**MR (Minor response)**

The minor response designation is assigned in cases where the decrease in the SPD is > 25% but < 50%. Morphologic detection of disease in a biopsy sample of the mass may be present. There may also be persistent morphologic detection of disease in the BM and CSF, if this finding was present at diagnosis; however, there should be a 25% to 50% reduction in the percentage of lymphoma cells. There should be no new and/or PD elsewhere.

**No response**

The no response designation will be applied for those patients whose residual lesions do not meet the criteria for CR, PR, minor response, or PD.

**PD (Progressive disease)**

The PD designation is applied for any patient with > 25% increase in the SPD of residual lesions, Deauville score 4 or 5 on FDG-PET with an increase in lesional uptake from baseline, or documentation of new lesions. PD also applies to any patient who develops new morphologic evidence of BM or CNS disease.

**Appendix 2.2 TABLE 1. International Pediatric NHL Response Criteria**

**Table 1.** International Pediatric NHL Response Criteria

| Criterion | Definition |
|---|---|
| CR | Disappearance of all disease (three designations) |
| CR | CT or MRI reveals no residual disease or new lesions |
| | Resected residual mass that is pathologically (morphologically) negative for disease (detection of disease with more sensitive techniques described as supporting data [Table 2]) |
| | BM and CSF morphologically free of disease (detection of disease with more sensitive techniques described as supporting data [Table 2]) |
| CRb | Residual mass has no morphologic evidence of disease from limited or core biopsy (detection of disease with more sensitive techniques described as supporting data [ Table 2]), with no new lesions by imaging examination |
| | BM and CSF morphologically free of disease (detection of disease with more sensitive techniques described as supporting data [ Table 2]) |
| | No new and/or progressive disease elsewhere |
| CRu | Residual mass is negative by FDG-PET; no new lesions by imaging examination |
| | BM and CSF morphologically free of disease (detection of disease with more sensitive techniques described as supporting data [Table 2]) |
| | No new and/or progressive disease elsewhere |
| PR | 50% decrease in SPD on CT or MRI; FDG-PET may be positive (Deauville score or 4 or 5 with reduced lesional uptake compared with baseline); no new and/or PD; morphologic evidence of disease may be present in BM or CSF if present at diagnosis (detection of disease with more sensitive techniques described as supporting data [Table 2]); however, there should be 50% reduction in percentage of lymphoma cells |
| MR | Decrease in SPD $> 25\%$ but $< 50\%$ on CT or MRI; no new and/or PD; morphologic evidence of disease may be present in BM or CSF if present at diagnosis (detection of disease with more sensitive techniques described as supporting data [Table 2]); however, there should be 25% to 50% reduction in percentage of lymphoma cells |
| NR | For those who do not meet CR, PR, MR, or PD criteria |
| PD | For those with $> 25\%$ increase in SPD on CT or MRI, Deauville score 4 or 5 on FDG-PET with increase in lesional uptake from baseline, or development of new morphologic evidence of disease in BM or CSF |

Abbreviations: BM, bone marrow; CR, complete response; CRb, complete response biopsy negative; CRu, complete response unconfirmed; CT, computed tomography; FDG, [$^{18}$F]fluorodeoxyglucose; MR, minor response; MRI, magnetic resonance imaging; NHL, non-Hodgkin lymphoma; NR, no response; PD, progressive disease; PET, positron emission tomography; PR, partial response; SPD, sum of product of greatest perpendicular diameters.

**Appendix 2.2 TABLE 2. Supporting International Pediatric NHL Response Criteria Data**

**Table 2.** Supporting International Pediatric NHL Response Criteria Data

| Supporting Information | Description |
|---|---|
| BM involvement | Currently defined by morphologic evidence of lymphoma cells; this applies to any histologic subtype; type and degree of BM involvement should be specified* |
| BMm | BM positive by morphology (specify percentage of lymphoma cells) |
| BMi | BM positive by immunophenotypic methods (histochemical or flow cytometric analysis; specify percentage of lymphoma cells) |
| BMc | BM positive by cytogenetic or FISH analysis (specify percentage of lymphoma cells) |
| BMmol | BM positive by molecular techniques |
| CNS involvement | |
| CSF status | CSF positivity is based on morphologic evidence of lymphoma cells; CSF should be considered positive when any number of blasts is detected; CSF may be unknown; as with BM, type of CSF involvement should be described whenever possible |
| CSFm | CSF positive by morphology (specify No. of blasts/μL) |
| CSFi | CSF positive by immunophenotype methods (histochemical or flow cytometric analysis; specify percentage of lymphoma cells) |
| CSFc | CSF positive by cytogenetic or FISH analysis (specify percentage of lymphoma cells) |
| CSFmol | CSF positive by molecular techniques |
| RM | |
| RMm | Tumor detected by standard morphologic evaluation |
| RMi | Tumor detected by immunophenotypic methods (immunohistochemical or flow cytometric analysis) |
| RMc | Tumor detected by cytogenetic or FISH analysis |
| RMmol | Tumor detected by molecular techniques |

Abbreviations: BM, bone marrow; FISH, fluorescent in situ hybridization; NHL, non-Hodgkin lymphoma; PB, peripheral blood; RM, residual mass.
*Same approach should be used for PB involvement (ie, PBm, PBi, PBc, PBmol).

## APPENDIX 3: MODIFIED SEATTLE CRITERIA FOR THE DIAGNOSIS OF VOD/SOS

Hepatic VOD/SOS is a syndrome comprising weight gain, ascites, painful hepatomegaly and jaundice, and occurs frequently in patients following allogeneic stem cell transplantation. Risk factors include the use of myeloablative conditioning, a stem cell source other than a matched sibling donor, pre-existing liver disease, and poor performance status. This has also been described following treatment with Mylotarg and with InO.

Two diagnostic systems are in common use, and are shown here: the modified Seattle criteria[99], and the Baltimore criteria[113]. The Baltimore criteria are more stringent, with an absolute requirement for hyperbilirubinemia. In this protocol we will use the Modified Seattle Criteria to define VOD/SOS. Formally these criteria describe VOD/SOS within 20 days post-HSCT, but since VOD/SOS may also occur post-InO and/or at a later time-point, for this study we will consider all occurrences of VOD/SOS per the definition below:

| **Modified Seattle Criteria [99]** |
|---|
| Two of the following criteria must be present:<br>- Total bilirubin > 34.2 µmol/l (2mg/dL)<br>- Hepatomegaly or right upper quadrant pain<br>- Weight gain (> 2% from pre-transplant weight) |

Other factors that may point at VOD/SOS include:

- ascites,
- thrombocytopenia with refractoriness to platelet transfusion,
- changes in the flow of v. portae.

Therefore, when evaluating liver toxicity, the radiologist should be informed of the potential for hepatic vascular disease. When VOD is in the differential diagnosis, a right upper quadrant ultrasound with color flow doppler (including indices to hepatic artery flow and evaluation of hepatic venous outflow) should be performed. In addition, the radiology report should describe common bile duct, the degree of gall bladder wall thickening in millimeters, and the volume of ascites should be estimated as closely as possible (ie, small and localized, moderate and generalized, or large and generalized).

The severity of VOD/SOS can be graded by (Seattle criteria)[114]:

| | |
|---|---|
| Mild | No adverse effects of liver disease, AND<br>No medications required for diuresis or hepatic pain, AND<br>All symptoms, signs and laboratory features reversible |
| Moderate | Adverse effects of liver disease present, AND<br>Sodium restriction or diuretics required, OR<br>Medication for hepatic pain required, AND<br>All symptoms, signs and laboratory features reversible |
| Severe | Adverse effects of liver disease present, AND<br>Symptoms, signs or laboratory features not resolved by day +100, OR<br>Death |

**APPENDIX 4: FORMULAE**

Mosteller BSA Formula [115]

$$\text{BSA (m}^2\text{)} = \sqrt{\frac{\text{Height (cm)} \times \text{Weight (kg)}}{3600}}$$

## APPENDIX 5: REINDUCTION UKALL-R3 THERAPY COMBINED WITH INO

Time-table UKALL-R3 block without mitoxantrone combined with InO

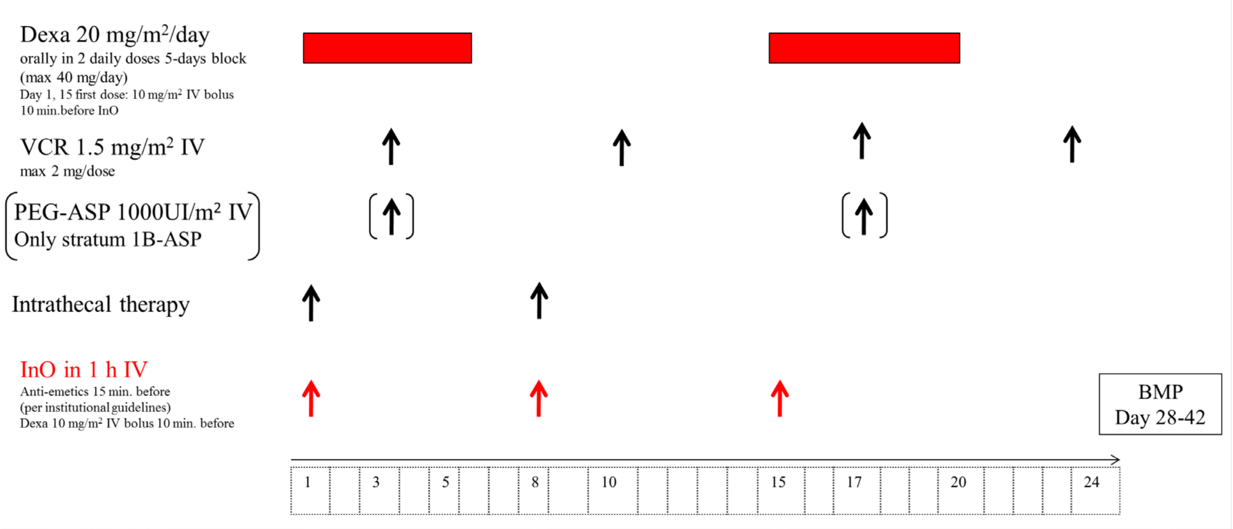


* Intrathecal therapy will be given as described in ***Table 10***; ***Table 11*** *and* ***Table 12*** in Section 4.3.3. The intrathecal injection may be combined with the diagnostic procedure, in which case the lumbar puncture of day 1 can be omitted.
**For premedication refer also to ***Table 10***, for dosage level refer to ***Table 15***

## APPENDIX 6: DRUG ADMINISTRATION SETUP AND PROCESS FOR LOW VOLUME DOSES

A syringe with a stopcock may be used for low volume administration to maintain a closed system during the infusion line flush process.

The set up for this process is shown in the figure below. The syringe containing the IMP dose is connected to one arm of the stopcock, a saline flush syringe is to be connected to a second arm of the stopcock, and an acceptable small volume infusion line (with or without filter) is to be connected to the longest arm of the stopcock. The infusion line connected to the stopcock may be primed with IMP solution or with normal saline (depending on site process), however if priming with normal saline, it should be noted that the initial portion of the infusion will only contain the saline prime volume and therefor the true start time of IMP infusion should be back calculated based on the infusion rate used and the volume of the saline prime.

The IMP infusion line must be connected to a primary infusion line running with NS at KVO rate (per institutional standard) or greater to maintain line patency. Program the infusion pump to administer the contents of the IMP syringe over the required time. Once the IMP syringe is empty, close the IMP arm of the stopcock and open the saline flush arm. Insert the saline flush syringe into the syringe pump and begin the infusion of the saline flush at the same rate as the IMP infusion to prevent accidental bolus of IMP to the patient during the flush process. The flush syringe must contain sufficient volume to ensure complete administration of IMP dose to the patient. This should take the hold volume of the stopcock and the infusion line into consideration.

**Infusion Setup**

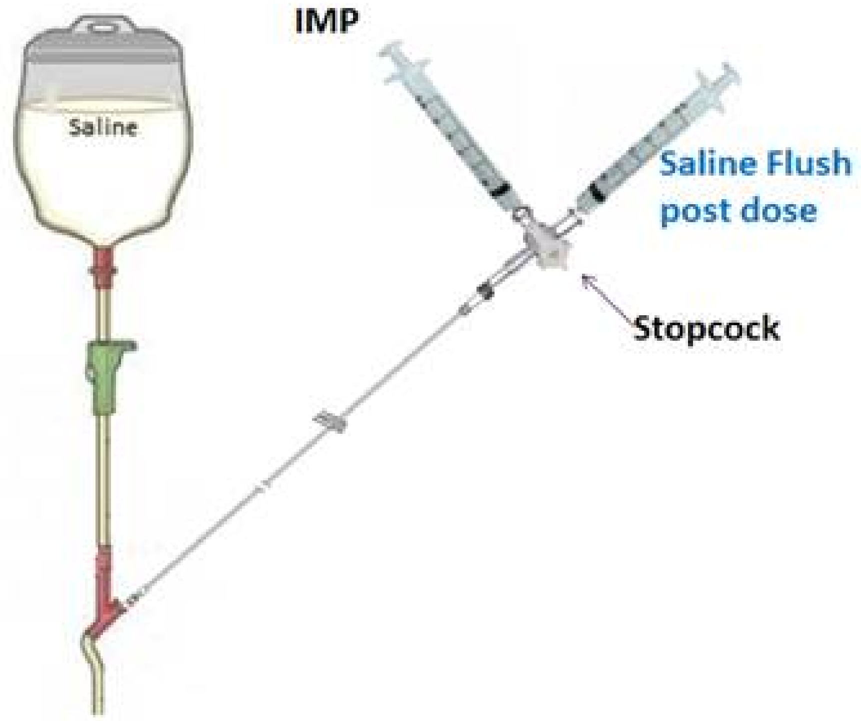

Supplement: Supplementary file 2 — Study ITCC-059 Protocol [file 41375_2022_1576_MOESM2_ESM.pdf]
